# Development of a Rapid Adeno-Associated Virus (AAV) Identity Testing Platform through Comprehensive Intact Mass Analysis of Full-Length AAV Capsid Proteins

**DOI:** 10.1021/acs.jproteome.3c00513

**Published:** 2023-12-20

**Authors:** Josh Smith, Felipe Guapo, Lisa Strasser, Silvia Millán-Martín, Steven G. Milian, Richard O. Snyder, Jonathan Bones

**Affiliations:** † Characterisation and Comparability Laboratory, The National Institute for Bioprocessing Research and Training, Foster Avenue, Mount Merrion, Dublin A94 X099, Ireland; ‡ Patheon Viral Vector Services, 13859 Progress Blvd, Alachua, Florida 32615, United States; § School of Chemical and Bioprocess Engineering, University College Dublin, Belfield, Dublin D04 V1W8.F, Ireland

**Keywords:** adeno-associated virus, cell and gene therapy, hydrophilic interaction liquid chromatography−mass spectrometry, AAV intact viral capsid protein screening, good manufacturing practices, rapid identity testing

## Abstract

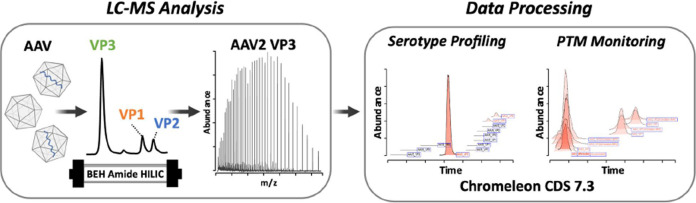

Adeno-associated viruses (AAVs) are commonly used as vectors for the delivery of gene therapy targets. Characterization of AAV capsid proteins (VPs) and their post-translational modifications (PTMs) have become a critical attribute monitored to evaluate product quality. Liquid chromatography–mass spectrometry (LC–MS) analysis of intact AAV VPs provides both quick and reliable serotype identification as well as proteoform information on each VP. Incorporating these analytical strategies into rapid good manufacturing practice (GMP)-compliant workflows containing robust, but simplified, data processing methods is necessary to ensure effective product quality control (QC) during production. Here, we present a GMP-compliant LC–MS workflow for the rapid identification and in-depth characterization of AAVs. Hydrophilic interaction liquid chromatography (HILIC) MS with difluoroacetic acid as a mobile phase modifier is utilized to achieve the intact separation and identification of AAV VPs and their potential proteoforms. Peptide mapping is performed to confirm PTMs identified during intact VP analysis and for in-depth PTM characterization. The intact separations platform is then incorporated into a data processing workflow developed using GMP-compliant software capable of rapid AAV serotype identification and, if desired, specific serotype PTM monitoring and characterization. Such a platform provides product QC capabilities that are easily accessible in a regulatory setting.

## 1 Introduction

Adeno-associated viruses (AAVs) are increasingly being utilized as vectors for gene therapies because of their low immunogenicity and cytotoxicity, high delivery efficacy, and broad tropism due to the variety of serotypes available.^1−4^ Structurally, AAVs consist of an icosahedral, 60-mer capsid composed of the three viral proteins (VPs) VP1, VP2, and VP3 in an approximate 1:1:10 ratio, respectively.^5^ However, recent studies have shown that this stoichiometric ratio may be more varied than once thought and that alterations in this VP ratio during manufacturing can impact product potency.^6,7^ Although each serotype has its own unique VP1, VP2, and VP3 proteins, within a single serotype, all the VPs have the same C-terminal region, with the sequence of VP3 contained within that of VP2 and the sequence of VP2 contained within that of VP1.^4^ These capsid proteins have been found to inherit post-translational modifications (PTMs) during production and storage, some of which are reported to impact transduction efficiency and infectivity.^4,8−10^ Thus, evaluating such critical quality attributes (CQAs) is crucial to ensuring product quality.

Liquid chromatography–mass spectrometry (LC–MS) is increasingly utilized for the characterization of AAV VPs with hydrophilic interaction liquid chromatography (HILIC) and reversed-phase (RP) chromatography being the two LC separation phases most used.^4,9,11−15^ Complete separation of VP1, VP2, and VP3 is desired for comprehensive intact VP characterization and effective determination of VP ratios, with multiple recent intact VP separation studies illustrating an ability to achieve complete VP separation of multiple serotypes.^4,12,15^ However, separation of VP2 and VP1 of AAV2 has been shown to be difficult to achieve and those that succeed in full VP separation of AAV2 suffer from MS ion suppression effects.^4^ Additionally, VP separation is only part of what is required if it is desired to apply such strategies within a quality control (QC) environment, something increasingly necessitated by the increased use of AAV vectors in cell and gene therapies. In a QC environment, it is imperative that comprehensive, good manufacturing practice (GMP) compliant workflows incorporate both AAV VP LC–MS-based separation strategies and simplified data processing methods for rapid QC control during production.

Here, we present a GMP-compliant LC–MS workflow developed for the rapid identification and in-depth characterization of AAVs. Using AAV2 as a test case, separation and identification of the intact VPs were performed using HILIC-MS with difluoroacetic acid (DFA) utilized as a mobile phase additive. Peptide mapping was performed for in-depth PTM analysis and confirmation of PTMs detected during intact VP analysis. The HILIC-MS platform was then incorporated into the development of a workflow using GMP-compliant software capable of rapidly performing AAV serotype identification and then, if desired, performing PTM characterization on a selected serotype. Such a platform provides quick and reliable serotype identification using a single universal processing method and enables PTMs monitoring for product quality, all while being capable of being performed within a regulatory setting.

## 2 Materials and Methods

### 2.1 Chemicals and Solvents

All reagents and solvents used were ACS reagent grade or better. Research-grade AAV2 was produced by Patheon by transient transfection in HEK293 cells. AAV2, AAV5, AAV8, and AAV9 serotypes produced using baculovirus infection of Sf9 cells were purchased from Virovek (Hayward, CA, USA). The NanoOrange Protein Quantitation Kit was obtained from Biosciences (Dublin, Ireland). IonHance DFA was purchased from Waters Corporation (Milford, MA, USA). The Thermo Scientific SMART Digest pepsin kit was obtained from Thermo Fisher Scientific (Sunnyvale, CA, USA). Optima LC–MS grade acetonitrile (ACN), Thermo Scientific UHPLC-MS-grade water, formic acid (FA), and tris(2-carboxyethyl)phosphine hydrochloride (TCEP) were sourced from Fisher Scientific (Dublin, Ireland). All other chemicals or solvents were obtained from Merck Sigma (Wicklow, Ireland). Purified water was obtained from an Arium pro Ultrapure Water System (Sartorius, Göttingen, Germany).

### 2.2 Analytical Instrumentation

Full-length AAV capsid protein separation was performed on a Vanquish Horizon ultrahigh pressure liquid chromatography (UHPLC) instrument consisting of a Binary Pump H (VH-P10-A-02), Split Sampler HT with 25 μL autosampler loop (VH-A10-A-02), Column Compartment H (VH-C10-A-03), and Fluorescence Detector F (VF-D50-A), coupled to an Orbitrap Exploris 240 Mass Spectrometer (MS) through a standard flow Ion MAX Source containing a heated electrospray ionization (H-ESI) probe (Thermo Fisher Scientific, Bremen, Germany). UHPLC instrument module settings used are as follows: autosampler temperature set to 5 °C; column compartment temperature set to 25, 45, or 60 °C; fluorescence detector excitation wavelength (λ_ex_) set to 280 nm, emission wavelength (λ_em_) set to 348 nm, and detector sensitivity set to 4. AAV peptide mapping was performed on a Dionex UltiMate 3000 RSLCnano system consisting of an NCS-3500RS nanoLC/ProFlow flow meter, loading pump, column compartment, and autosampler coupled to a Q-Exactive Plus Hybrid quadrupole-Orbitrap MS using an EASY-Spray source (Thermo Scientific, Bremen, Germany). RSLCnano module settings used are as follows: autosampler temperature set to 5 °C and column compartment temperature set to 45 °C. Instrument modules were controlled with, and capsid protein separation and peptide mapping data was acquired using Thermo Scientific Chromeleon^TM^ Chromatography Data System software (referred to as Chromeleon from now on) versions 7.2.10 ES and 7.2.10 respectively (Thermo Scientific, Germering, Germany).

### 2.3 AAV Protein Concentration Determination

The concentration of the AAV2 sample was determined using a NanoOrange Protein Quantitation Kit composed of a 500× NanoOrange protein quantitation reagent (Component A), a bovine serum albumin (BSA) standard (Component B), and a NanoOrange protein quantitation diluent (10× concentrated). This fluorescence-based assay was utilized due to its high sensitivity, enabling the detection of low protein concentrations. Following the instructions provided, a 1× NanoOrange working solution was prepared by performing a 10× dilution of NanoOrange protein quantitation diluent (10×) with purified water producing a 1× protein quantitation diluent subsequently used to perform a 500× dilution of the 500× NanoOrange protein quantitation reagent. BSA samples (for generation of protein standard curve) in concentrations of 10, 6, 3, 1, 0.6, 0.3, and 0.1 μg·mL^–1^ and 2% v·v^–1^ AAV samples were prepared in 1× NanoOrange working solution (total volume 250 μL per sample). All samples were prepared in triplicate. Prepared samples were incubated at 95 °C for 10 min in the dark and then allowed to cool to room temperature in the dark for 30 min; 200 μL of each sample was transferred to a 96-well plate and read using a fluorescence plate reader with excitation and emission wavelengths of 485 and 590 nm, respectively. The concentration was calculated from the resulting assay readings.

### 2.4 AAV Capsid Protein Separation

Full-length capsid protein separation of AAV2 was performed using an Acquity UPLC Glycoprotein BEH Amide Column, 300 Å, 1.7 μm, 2.1 × 150 mm (Waters Corporation, Milford, MA, USA). One microgram (1 μg, 8.75 μL) of the AAV2 sample was injected neat (storage buffer was PBS containing 0.001% Pluronic F68), with triplicate injections performed. AAV capsid separation was performed following a modified version of the LC parameters outlined by Liu et al. for VP separation.^4^ They were as follows: at a flow rate of 0.1 mL·min^–1^, an isocratic hold at 85% mobile phase B (85% B) was performed for 3.5 min, followed by a decrease to 64.5% B over 0.1 min. An isocratic hold was performed for 7.1 min followed by a linear decrease in mobile phase B from 64.5 to 58.5% over 21.3 min. A column wash was then performed by decreasing mobile phase B to 5% over 1.0 min while simultaneously increasing the flow rate from 0.1 to 0.3 mL·min^–1^. A hold at 5% B with a solvent flow rate of 0.3 mL·min^–1^ was performed for 3.5 min. Column re-equilibration followed by increasing mobile phase B to 85% over 0.5 min at 0.3 mL·min^–1^ and holding for 2.0 min at 85% B before increasing the flow rate to 0.4 mL·min^–1^ over 0.0 min. The flow rate was held for 2 min at 0.4 mL·min^–1^ before being decreased to 0.1 mL·min^–1^ over 0.0 min. The flow rate was held for 4.0 min at 85% B to ensure complete equilibration before the next injection. The column temperature was kept constant at 25, 45, or 60 °C, depending on which column temperature was being tested during a run. Mobile phase A was UHPLC-MS grade water containing 0.1% (v·v^–1^) DFA and mobile phase B was Optima LC–MS grade ACN containing 0.1% (v·v^–1^) DFA.

Global MS data parameters utilized on the Orbitrap Exploris 240 instrument were as follows: full-length protein was selected for application mode, low pressure was selected for pressure mode, liquid chromatography was selected for the infusion mode, the expected LC peak width was 30 s, advanced peak determination was selected, the default charge state was 35, and internal mass calibration was off. The ion source properties consisted of using an H-ESI ion source with a static spray voltage and a positive ion capillary voltage of 2700 V. A static gas mode was utilized with the sheath gas at 20, aux gas at 5, and sweep gas at 0. The ion transfer tube temp and vaporizer temp were set at 320 and 150 °C, respectively.

The MS scan parameters used are also as follows: full scan MS1 analysis was performed and was run in positive ion mode with a scan range of *m*/*z* 867–2400. Samples were analyzed with an Orbitrap resolution of 15,000 (15k) or 45,000 (45k) at *m*/*z* 200 to see if higher resolving power could improve PTM identification. At both resolution settings, the RF lens was set at 125%, the normalized AGC target was 50%, the maximum injection time was 200 ms, and the number of microscans was set to 10. Data were collected in profile mode. To assist in desolvation, the in-source CID was set to 35 V.

### 2.5 Peptide Mapping Sample Preparation

Sample preparation of AAV2 for peptide mapping was performed using a SMART Digest magnetic bead bulk pepsin kit and following the protocol previously described by Guapo et al.,^14^ with sample digestion performed in triplicate. Briefly, for each digestion, 4 μg (35 μL) of the AAV sample were denatured and reduced by bringing the AAV sample to a total volume of 50 μL with LC–MS grade water, before adding 148 μL of SMART Digest buffer and 2 μL of TCEP (0.5 M), producing a 200 μL solution with a final TCEP concentration of 5 mM. Pepsin beads were prepared by mixing 15 μL of pepsin beads with 100 μL SMART Digest buffer and a wash solution for the digest was prepared by mixing 50 μL of SMART Digest buffer with 150 μL of LC–MS-grade water; 200 μL of the denatured AAV sample, 100 μL of the pepsin bead solution, and 200 μL of the wash solution were then added to different wells of a 96-deepwell plate (Thermo Fisher Scientific, Vantaa, Finland). Sample digestion on the pepsin beads was performed using a Thermo Scientific KingFisher Duo Prime purification system under the control of Thermo Scientific BindIt software, version 4.0. The sample was incubated on the pepsin beads for 40 min at 70 °C and set to medium mixing speed to prevent sedimentation of the beads. After incubation, the beads were removed, with the remaining sample volume transferred to 1.5 mL Eppendorf protein LoBind tubes (Eppendorf, Dublin). Samples were then acidified with the addition of trifluoroacetic acid (TFA) at a final concentration of 0.1% (v·v^–1^) for 5 min followed by centrifugation at 14,000 × *g* for 5 min to pellet any pepsin beads that might not have been removed. Subsequently, samples were transferred to clean 1.5 mL Eppendorf protein LoBind tubes and evaporated to dryness by using vacuum centrifugation.

### 2.6 Peptide Mapping LC–MS Analysis

Peptide mapping LC-MS analysis was performed following the procedure described in Guapo et al.^14^ The resulting peptides from the section outlining peptide mapping sample preparation were separated using a Thermo Scientific Easy-Spray PepMap RSLC C18 column, 2 μm, 75 μm × 50 cm (Thermo Fisher, Sunnyvale, CA, USA), Milford, MA, USA). Dried digested samples were reconstituted in 0.1% FA in water to a final concentration of 100 ng μL^–1^, with 200 ng (2 μL) of digested AAV2 sample then injected into the column. Injections were performed in technical triplicate. Peptide separation was performed using a linear gradient from 2% B to 40% B over 105 min, followed by an increase to 60% B over 5 min. The column was then washed by increasing the percentage of B to 80% over 0.1 min followed by an isocratic hold for 9.9 min. Mobile phase B was then decreased to 2% over 0.1 min and then increased back to 80% over 4.9 min, followed by a 5 min isocratic hold. The column was then re-equilibrated by decreasing mobile phase B to 2% over 0.1 min and then performing an isocratic hold for 19.9 min. The flow rate was kept constant at 250 nL·min^–1^ and the column temperature was maintained at 45 °C. Mobile phase A was UHPLC-MS grade water containing 0.1% (v v^–1^) FA and mobile phase B was Optima LC-MS grade ACN containing 0.1% (v v^–1^) FA.

Data-dependent acquisition (DDA)-MS analysis was performed in the positive ion mode. Full scans were acquired at a resolution of 70,000 between a mass range of *m*/*z* 200–2000. The AGC target was set to 1.0 × 10^6^ with a maximum injection time of 100 ms and 1 microscan. MS–MS (MS^2^) fragment scans were acquired using a resolution setting of 17,500 with an AGC target of 1.0 × 10^5^, a maximum injection time of 200 ms, an isolation window of *m*/*z* 2.0, and a signal intensity threshold of 1.0 × 10^4^. Fragmentation of the ten most abundant precursor ions was performed using a normalized collision energy set to 28 with a dynamic exclusion set for 45 s and charge exclusion set to unassigned and >8. The MS tune parameters were as follows: spray voltage was set to 1.7 kV; capillary temperature was set to 310 °C; S-lens RF voltage was set to 50.

### 2.7 AAV Peptide Mapping Data Processing

Peptide identification and relative PTM quantitation were performed as outlined by Guapo et al.^14^ Briefly, a peptide mapping analysis experiment was created in BioPharma Finder (BPF) version 4.1 (Thermo Scientific, San Jose, CA, USA) to process the raw data files generated during peptide mapping LC–MS analysis using the parameters summarized in Table S1. Only peptides with *a* ≥ 95% confidence score, ≥1 × 10^5^ average MS area, and within ±5 ppm mass accuracy were included for sequence coverage evaluation. Peptides with adducts, unknown modifications, gas phase ions, and nonspecific generated peptides fitting these parameters were filtered from the results and thus not utilized for sequence coverage determination.

For PTM quantitation, the same parameters for the peptide sequence coverage were applied. PTMs were identified automatically in BPF by their mass differences compared with unmodified peptides generated from pepsin digestion. Relative PTM abundance was expressed in BPF as a percentage of PTM presence on a peptide to all forms of the said peptide present. Manual validation of peptides selected for relative PTM quantitation was performed in addition to automatic software assignment to ensure accurate quantitation.

### 2.8 Full-Length AAV Capsid Protein Data Processing

A mass analysis experiment was created in BPF to perform the identification of full-length AAV viral proteins and their isoforms using the parameters summarized in Tables S2 and S3. Multiconsensus analysis was performed to analyze the triplicate sample injections together. The resulting list of identified protein features was filtered to include only identifications found in all three triplicate samples, with a score ≥60. This high score was selected to ensure only the highest quality identifications in runs performed at all column temperatures, as components with scores below 60 were found to have poor deconvoluted spectra or could not be identified based on the PTMs found during peptide mapping. Once processed, full-length workbook files (intact .wbpf file format) of the identifications found were generated. These intact workbooks were imported into Chromeleon as part of the MS processing method used in the following section, discussing the development of a rapid AAV identity testing and PTM monitoring platform in Chromeleon.

### 2.9 Rapid AAV Identity Testing and PTM Monitoring Platform Development in Chromeleon

For rapid AAV identity testing method development, triplicate injections of baculovirus/Sf9 derived AAV2, AAV5, AAV8, and AAV9 serotypes (Virovek, Hayward, CA, USA) were separated using the HILIC-MS parameters described in the section describing AAV capsid protein separation. Separations were performed at a column temperature of 45 °C and with a 15k MS resolution. The scan range was *m*/*z* 867–2500. Identification of the VPs for each AAV serotype was performed in BPF using the method parameters outlined in the section describing full-length AAV capsid protein data processing. An “MS Quantitative” processing method was created within Chromeleon so that full-length protein deconvolution of all injections run in Chromeleon could be performed. Workbooks containing the identifications were generated as described in the section describing full-length AAV capsid protein data processing and imported into the Chromeleon MS Quantitative method. All parameters were left as is except for the following: in the chromatogram parameters, the time limit was set from 13.00 to 28.50 min. In the source spectra parameters, the retention time (RT) range was set from 13.0 to 28.5 min, the merge tolerance was set to 10 ppm, and the minimum number of detected intervals was set to 10. Data processing parameters in Chromeleon were then optimized to enable the correct identification of each serotype without other serotypes generating false identifications (see Table S4 for optimized data processing parameters). Method validation was performed by applying the developed processing method to triplicate injections of HEK293 cell-derived AAV2 samples run in the same sequence as the Sf9-derived AAVs as well as the HEK293-derived AAV2 samples used for VP separation in the section describing AAV capsid protein separation, which were run in a later sequence.

The HEK293-derived AAV2 samples separated at 25 °C and analyzed with a resolving power of 45k were used for the development of the PTM monitoring method. This method was created following the same methodology as the development of the rapid identity testing method but optimized for the characterization of the selected serotype (Table S5). Here, the component target list contained VP PTM modifications specific to the serotype being studied rather than a list of VPs from different serotypes.

## 3 Results and Discussion

### 3.1 AAV PTM Characterization

**Figure 1:**
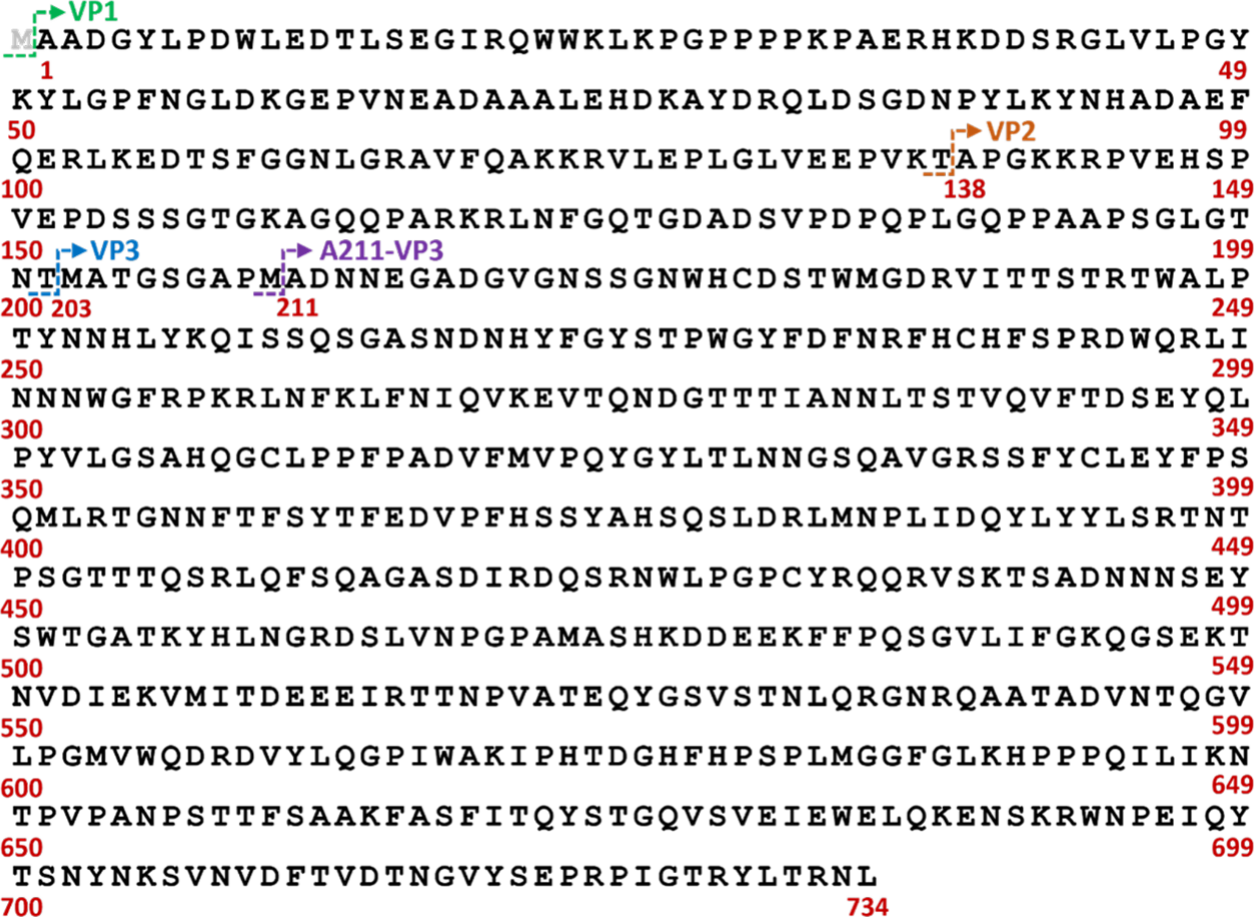
AAV2 capsid protein sequences searched when peptide mapping data were processed by LC-MS using pepsin digestion. The green arrow signifies the start of VP1, the orange arrow the start of VP2, the blue arrow the start of VP3, and the purple arrow the start of the VP3 variant A211-VP3. The red numbers convey the position of the amino acid residue above them in the VP1 protein sequence. Here, the amino acid residue considered to be the start of VP1 is the alanine residue with the red 1 under it as the N-terminal methionine (gray M) is cleaved off in the cell. Thus, here, the VP1 sequence would be considered A1-L734. Please see the web version of this article for the interpretation of color references if necessary.

To develop a QC-compliant workflow capable of both rapid serotype AAV identification and PTM characterization, it is important to understand the PTMs present on the AAV VPs. PTMs on biotherapeutic proteins are monitored as potential CQAs to inform process development, batch consistency, product stability, and more, as their presence can influence product quality, efficacy, and potentially patient safety.^8,16−20^ Comprehensive PTM analysis is crucial for in-depth characterization of full-length AAV VPs, as it aids in the proper identification of different VP proteoforms present and any potential consequences their presence might cause. For PTM determination, peptide mapping and relative PTM quantitation of AAV2 using on-bead pepsin digestion and nano-LC separation were performed in parallel to full-length VP analysis, following the procedure described by Guapo et al.^14^ One hundred percent sequence coverage was obtained after filtering identified peptides in BPF as described in the section describing AAV peptide mapping data processing for improved peptide mapping confidence (Figure S1). Here, it should be noted the VP1 sequence searched for peptide mapping was A2-735 as it is well-known that for AAV2 the N-terminal methionine (M, Met) residue is cleaved within the cell^9,11,21,22^ (Figure 1). Eighteen PTMs were identified with relative abundances greater than 1% including 2 acetylations on alanine (A, Ala) residues, 3 deamidations on asparagine (N, Asn) residues, 5 oxidations on Met residues, 1 oxidation-to-kynurenine on a tryptophan (W, Trp) residue, 3 succinimides formed from aspartic acid (D, Asp) residues (from now on referred to as succinimide D), 1 phosphorylation on a serine (S, Ser) residue, and loss of ammonia (NH_3_) from 3 glutamine (Q, Gln) residues (Table 1).

**Table 1:** Summary of PTMs Identified during Peptide Mapping of AAV2 with a Relative Abundance ≥1%

modification	peptide sequence^a^	confidence	recovery	average % abundance	SD
A1 + acetylation	AADGYLPDWLED	100.00	2.50	100.00	0.00
A203 + acetylation	ATGSGAPMADNNEGADGVGNSSGNWHCDSTWMGDRVITTSTRT	100.00	43.03	97.10	0.07
Q100 + NH3 loss^b^	QERLKEDTSFGGNLGRAVF	100.00	46.94	74.64	1.52
Q119 + NH3 loss^b^	QAKKRVLEPLGL	100.00	50.20	73.38	2.70
N56 + deamidation^b^	LVLPGYKYLGPFNGLDKGEPVNE	100.00	5.64	63.25	3.15
M633 + oxidation	AKIPHTDGHFHPSPLMGGFGLKHPPPQIL	99.99	35.20	10.24	1.14
D624 + succinimide D	AKIPHTDGHFHPSPLMGGF	100.00	49.60	5.79	0.11
Q606 + NH3 loss^b^	QDRDVYLQGPIW	100.00	38.19	5.44	0.25
N702 + deamidation^b^	IEWELQKENSKRWNPEIQYTSN	100.00	84.25	4.70	0.16
S148 + phosphorylation	VEEPVKTAPGKKRPVEHSPVEPD	100.00	5.22	4.43	0.85
D326 + succinimide D	FNIQVKEVTQNDGTTTIANNLTST	100.00	32.30	4.26	0.22
M557 + oxidation	KVMITDE	100.00	134.22	3.49	0.62
D344 + succinimide D	VQVFTDSE	100.00	24.81	2.77	0.36
M433 + oxidation	DRLMNPLIDQYL	100.00	94.54	2.23	0.02
M401 + oxidation	EYFPSQM	100.00	137.46	2.11	0.21
W617 + oxidation to kynurenine	LQGPIW	100.00	31.14	1.50	0.05
N93 + deamidation^b^	YDRQLDSGDNPYLKYNHADAEF	100.00	41.24	1.32	0.02
M603 + oxidation	EEIRTTNPVATEQYGSVSTNLQRGNRQAATADVNTQGVLPGMVWQDRD	100.00	16.39	1.13	0.05

a The peptide sequence shown is that of the most abundant peptide to contain its associated modification.

b It could not be fully determined if the modifications detected were process-related or a result of the digestion conditions used for peptide mapping. All PTMs above the dashed line have a relative abundance >10%.

A common PTM of AAVs is N-terminal acetylation. It is known that in nature over 80% of human proteins are cotranslationally N-term acetylated.^23^ Proteins with N-term Met are often cleaved by Met-aminopeptidases when the amino acid at position 2 is Ala, valine (V, Val), Ser, threonine (T, Thr), cystine (C, Cys), glycine (G, Gly), or proline (P, Pro), due to their small side chains.^23,24^ When the N-term Met is acetylated before cleavage, the result of Met cleavage is almost always the acetylation of the resulting N-term amino acid. For multiple AAV serotypes, VP1 and VP3 contain N-term Met which is cleaved, resulting in the acetylation of the subsequent amino acid residue.^9,11,21,22^ For AAV2, this results in acetylated A1 (A2 if the Met residue is included) and acetylated A203, the N-term amino acids for VP1 and VP3, respectively, after Met cleavage. As expected, we saw near 100% acetylation present at both A1 (100%) and A203 (97.1%). Additionally, the presence of multiple peptides starting with acetylated A211 signified the presence of a VP3 variant A211(Ac)–L735 (A211(Ac)-VP3) previously detected in AAV2 by Oyama et al.^22^ (see Table S6 and Figure S2). Its presence was confirmed during the full-length VP separation analysis of AAV2 discussed in the section on full-length AAV VP proteoform identification. Although its existence is well-known, the biological significance of N-term acetylation is less so, it has been shown to be a potential signal for the degradation of cellular proteins or a site for ubiquitination.^23^ For AAVs, it is suggested that N-term acetylation is linked to viral capsid degradation and uncoating^11^ and might influence AAV transduction.^21^ However, further study is needed.

Deamidation of Asn to Asp or isoaspartic acid (Iso-D, Iso-Asp) is often considered to impact the function of biotherapeutics including their potency, efficacy, and safety, and thus is monitored as a CQA.^8^ While digestion was performed at low pH, which is shown to reduce deamidation formation,^25^ deamidation at N56 (N57 when including Met) was found to be present 63.25% of the time, over 13 times more abundant than the next incidence of deamidation. Given that the presence of deamidation at N56 in AAV2 has been shown to reduce transduction efficiency^8,21^ it is important to understand the high level of deamidation at this site. Previous studies have shown that the structure of amino acid residue following Asn (*N* + 1) will influence the rate of deamidation, as its size and charge impact the local flexibility of the peptide backbone.^8,26,27^ Gly as the *N* + 1 residue has been shown to increase the presence of deamidation the most with at least one AAV study showing >75% deamidation occurring at Asn residues when Gly is the *N* + 1 residue.^8,28^ When the *N* + 1 residues were examined for all deamidated Asn detected during AAV2 peptide mapping, Gly was only present as the *N* + 1 residue for N56, which would help explain its high relative abundance compared to other deamidation features present. Additionally, a recent study has shown that sample preparation conditions including denaturation temperature, digestion temperature, and digestion duration can generate deamidation artifacts during peptide mapping that exaggerate the levels of deamidation present.^29^ Given that pepsin digestion for peptide mapping is performed under acetic conditions (≈pH 2) and at an elevated temperature (70 °C), it is possible that the sample preparation conditions are generating deamidation artifacts that are contributing to the high levels of deamidation seen at N56 and inflating the levels of deamidation seen at lower abundances. These findings indicate that more work is needed to accurately determine how much of the detected deamidation is process-related and to what extent it is an artifact of sample preparation, as the current digestion conditions of this method do not differentiate between the two and thus cannot reliably quantify process related deamidation. These conditions were used because it has previously been reported that more traditional enzymes like trypsin achieved limited sequence coverage when utilized for peptide mapping of AAVs.^14^ However, if separation of the VPs is performed before peptide mapping, then enzymes like trypsin might be more effective as they would only be digesting the VP proteins without needing to dissociate the AAV capsids, as that would have occurred during VP separation. Given that optimum trypsin digestion occurs at a neutral pH (≈pH 7.8) and is usually performed at 37 °C, the digestion conditions are unlikely to impact deamidation, and thus, any deamidation detected would be representative of process-related deamidation. Although such an investigation is beyond the scope of this study it warrants further investigation.

Oxidation is a PTM monitored as a CQA during production, purification, or long-term storage since its presence can adversely impact biotherapeutic stability, efficacy, and product safety.^18,19,30^ A prominent oxidation observed in biotherapeutics is at the sulfur on the Met side chain to create Met sulfoxide.^17,31^ Here, the only Met oxidation with a relative abundance of >10% was found at M633 (10.24%). Additionally, four Met oxidations were detected at M401 (2.11%), M433 (2.23%), M557 (3.49%), and M603 (1.13%). While less common, oxidation can also occur on tryptophan forming a variety of oxidized Trp products.^17^ The occurrence of Trp oxidation to kynurenine (+4 Da mass shift) was observed on residue W617 at low levels (1.50%—Figures S6 and S7).

The loss of NH_3_ on Gln amino acid residues is also readily abundant, with this modification detected at Q100 (74.64%—Figure S3), Q119 (73.38%—Figure S4), and Q606 (5.44%—Figure S5). Gln can naturally slowly undergo deamidation and it is suggested that mechanistically the deamidation process is similar to that of Asn, including the possible formation of a glutarimide-like intermediate.^32^ However, the abundance of such an intermediate compared with either Gln or its deamidated product glutamic acid (E, Glu) is generally minimal. Alternatively, it has been shown that peptides containing N-terminal Gln can undergo partial deamidation in solution^33^ and thus it is possible some of the glutamine NH_3_ loss occurs during the peptide digestion. Additionally, it has been shown that during collision-induced dissociation (CID) MS analysis, NH_3_ loss readily occurs on peptides containing Gln to form a variety of ringed structures.^18,19,30^ Furthermore, deamidation is the predominant neutral mass loss on N-terminal Gln when a peptide has no “mobile protons”, meaning the charge state of the peptide is less than or equal to the number of basic amino acids (Arg, Lys, His) contained within it.^34^ Upon examination of the peptides containing glutamine NH_3_ loss, all peptides contained N-terminal Gln. The two with a high abundance of this modification (>70%) have no mobile protons while the peptide with a relatively low abundance (<6%) has mobile protons (Table 2). Therefore, it is possible that some, if not most, of the detected Gln NH_3_ loss, especially at Q100 and Q119, is produced during sample preparation and MS analysis instead of being a process-induced PTM. Like with Asn deamidation, it is currently not possible to differentiate potential process-related glutamine NH_3_ loss and NH_3_ loss that is an artifact of sample preparation under the digestion conditions currently used. Once again, this might be remedied by separation of the VPs before peptide mapping and then using trypsin for digestion, as both the digestion conditions and the specificity of trypsin should minimize the presence of glutamine NH_3_ loss generated by the digestion process. Still, further studies are necessary to determine what portion of the detected NH_3_ loss is process-related and is beyond the scope of this study.

**Table 2:** Peptides Containing NH_3_ Loss on Gln and Their Mobile Protons

peptide sequence	positions	modification	site	confidence	charge state	neutral amino acids	mobile protons	average % abundance^a^
QERLKEDTSFGGNLGRAVF	100–118	NH_3_ loss	Q100	100	3	R102, K104, R115	0	74.64%
QERLKEDTSF	100–109	NH_3_ loss	Q100	100	2	R102, K104	0	
QAKKRVLEPLGL	119–130	NH_3_ loss	Q119	100	3	K121, K122, R123	0	73.38%
QAKKRVLEPLGL	119–130	NH_3_ loss	Q119	100	2	K121, K122, R123	0	
QDRDVYLQGPIW	606–617	NH_3_ loss	Q606	100	2	R608	1	5.44%
QDRDVYL	606–612	NH_3_ loss	Q606	100	2	R608	1	

a The average % abundance is that of the modification present for all peptides containing the amino acid where the modification occurs. It is calculated in Biopharma Finder for each file processed and then the values for each file were averaged.

Other PTMs of note identified above 1% relative abundance were the presence of succinimide on Asp residues D326 (4.26%), D344 (2.77%), and D624 (5.79%), as well as phosphorylation at Ser residue S148 (4.43%). Succinimide is the ringed intermediate formed during the deamidation of Asn to Asp through the loss of NH_3_ (from now on referred to as succinimide N) or through the loss of water from Asp residues (succinimide D) and can negatively influence the efficacy of biotherapeutics.^17,31^ While unstable at physiological conditions, succinimide is stable at low pH^17^ and thus its detection is not surprising. Phosphorylation is known to play a critical role in a variety of cellular processes with preliminary research, suggesting that it might play a role in AAV transduction efficiency.^9,35−37^

### 3.2 AAV VP Separation

Establishing an effective VP separations platform is crucial to the development of a comprehensive data processing workflow capable of AAV serotype identification and PTM characterization. However, for certain serotypes such as AAV2, the complete separation of VP2 and VP1 proteins can be difficult to achieve on LC-MS platforms. Liu et al. recently demonstrated the separation of AAV2 VP2 and VP1 viral proteins using HILIC combined with mobile phases containing TFA.^4^ However, TFA as a mobile phase additive is generally not recommended for use with MS due to its ion-suppressing effects^38,39^ and necessitated desolvation gas modification to improve MS signal intensity. Alternatively, both Zhang et al. and Wu et al. found success in AAV VP separation utilizing DFA as a mobile phase additive for RP chromatography, although without achieving separation of AAV2 VP1 and VP2 proteins.^12,15^ Given that DFA has a similar effect on analyte separation as TFA without negatively impacting MS signal intensity during MS analysis,^40^ pairing it with HILIC has the potential to provide separation of AAV2 VP2 and VP1 proteins without impacting the quality of the MS spectra.

**Figure 2:**
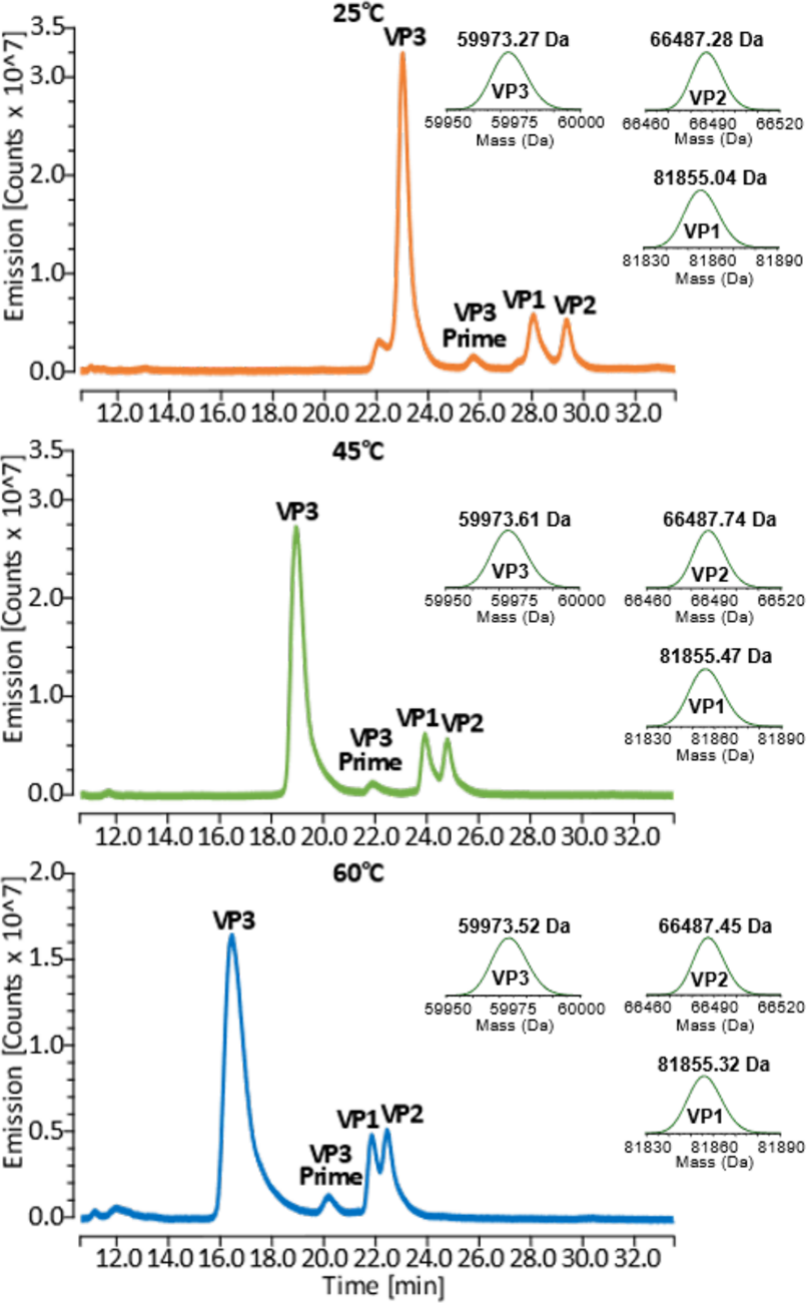
A comparison of HEK293-derived full-length AAV2 viral capsid protein (VP) separation profiles when separation is performed at column temperatures of 25 °C (top), 45 °C (middle), and 60 °C (bottom) during HILIC-FLR-MS using DFA as an ion pairing agent. Deconvoluted MS spectra of the unmodified VPs are shown on the right of each FLR trace. All samples were run in technical triplicate. Separation was performed on an Acquity UPLC glycoprotein BEH amide column, 300 Å, 1.7 μm, 2.1 × 150 mm with a gradient of 64.5–58.5% B. FLR traces were monitored by using λ_em_ = 280 and λ_ex_ = 348 nm. Clear separation of all three viral proteins was seen at all column temperatures with better separation between VP2 and VP1 observed as column temperature decreased. An additional peak labeled VP3 Prime was also detected.

**Figure 3:**
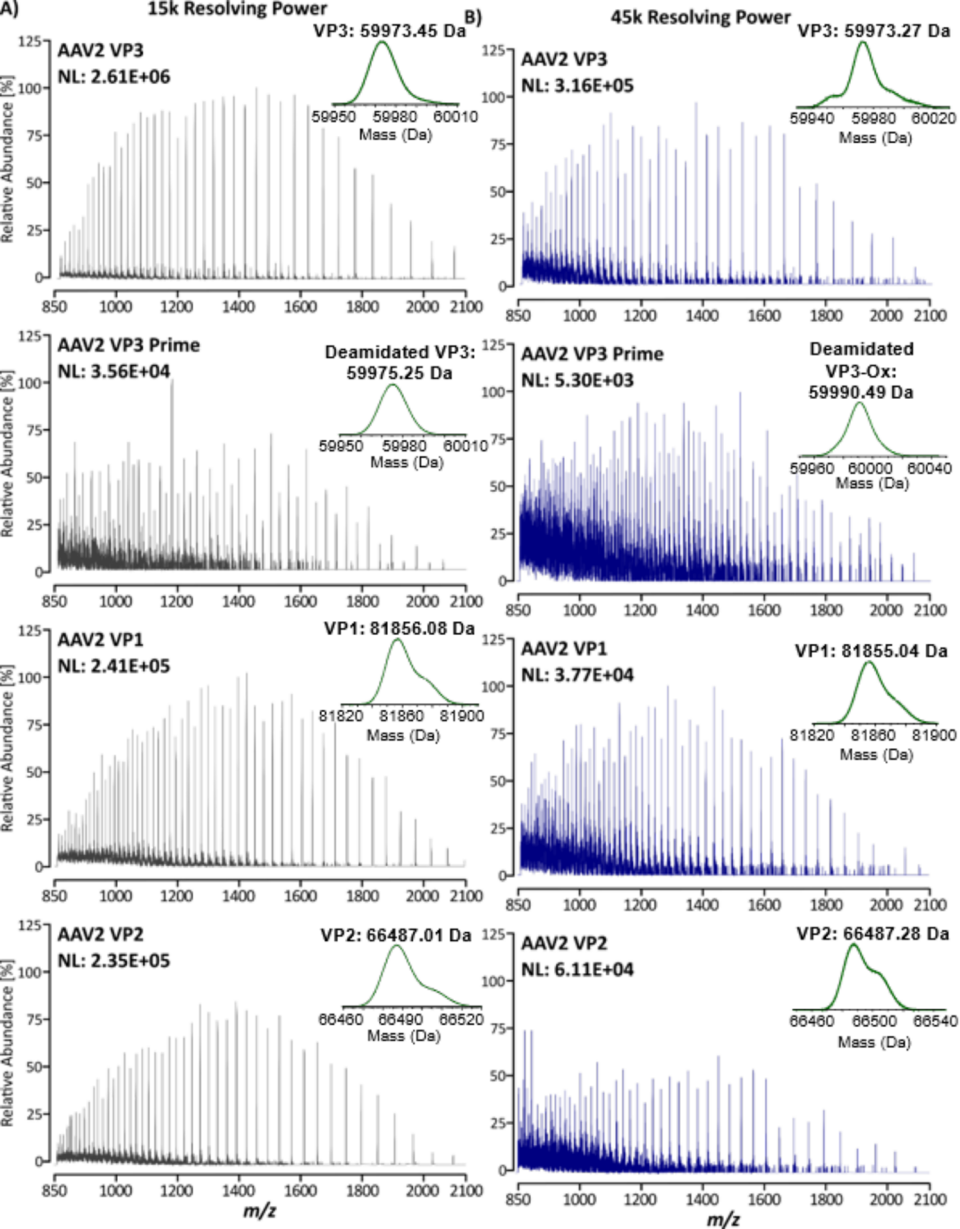
Raw MS spectra of AAV2 VPs analyzed at 15k resolving power (A) and 45k resolving power (B) illustrating the ability of this platform to generate high-quality MS spectra for full-length VP characterization. Top—VP3; middle top—VP3 prime; middle bottom—VP1; bottom—VP2. Deconvoluted MS spectra of the most abundant proteoform identified for each VP are shown above each spectrum on the upper right.

To test the effectiveness of DFA on VP separation using HILIC and as an ion pairing agent for MS analysis, full-length AAV2 VP separation was performed using HILIC-MS with 0.1% DFA added to the mobile phases. LC separation with in-line fluorescence (FLR) and MS detection was utilized for VP stoichiometric calculations. Separations were performed at 25, 45, and 60 °C to observe the impact of column temperature on VP separation, with triplicate injections performed at each temperature. As demonstrated in Figure 2, separation of the three VPs was achieved at all temperatures tested, and high-quality MS spectra were obtained for each VP peak (Figure 3). VP elution was found to be consistent with previous VP separation on HILIC,^4^ with VP3 eluting first, followed by VP1 and then VP2. Additionally, a small peak between VP3 and VP1 (herein termed VP3 prime) was detected in all runs. As column temperature increased, VP retention decreased, resulting in VPs eluting earlier in the gradient as expected. More crucially, the separation of VP1 and VP2 improved as the column temperature decreased, with significant VP1 and VP2 peak overlap occurring when separation was performed at 60 °C and near baseline separation achieved when separation was performed at 25 °C. The improved separation of VP2 and VP1 as the column temperature decreases is a result of the increased analyte phase partitioning that occurs between the ACN-rich mobile phase and the water-rich layer surrounding the stationary phase during HILIC analysis. The phase partitioning is considered to be an exothermic process and thus it is favored at lower column temperatures.^41^ This improved interaction results in greater VP retention to the HILIC column and thus both later elution and improved analyte separation. Determination of VP ratios is important as studies have shown that different VP stoichiometry during manufacturing can impact product potency.^6,7^ VP ratios were calculated using two methods, one FLR-based and one MS-based. For the FLR-based method, VP ratios were calculated by using the integrated VP peak areas from the FLR traces. With the MS-based method, VP stoichiometry was determined using the area under the extracted ion chromatograms (XICs) of the proteoforms identified in the section about full-length AAV VP proteoform identification. As Table 3 illustrates, column temperature had a direct influence on the calculated VP stoichiometry in the FLR method. Improved intact VP separation, particularly between VP1 and VP2, at lower temperatures enables improved peak integration as it minimizes the coelution of the VP2 and VP1 peaks seen at higher temperatures. This coelution can misconstrue the amount of each VP identified, as the integration of either VP1 or VP2 will inevitably contain some amount of the other VP because of the lack of complete separation. In this study, the changes in column temperature resulted in the detected copy numbers of VP1 increasing and the detected copy numbers of VP2 decreasing as the column temperature was lowered, despite the copy numbers of VP3 only minimally changing. It has been suggested that varying ratios of VP1 to VP2 could have an impact on AAV potency,^7^ so the discrepancies seen at the different column temperatures illustrate a limitation to using FLR-based quantitation methods. However, with the MS deconvolution method, consistent VP copy numbers and similar VP ratios were detected at all column temperatures, suggesting that using MS spectra can be a more robust method for VP stoichiometric calculations when only partial separation of VP2 and VP1 is achieved (Table 3).

**Table 3:** AAV2 Viral Protein Ratios Determined by HILIC-FLR-MS at Column Temperatures of 25, 45, and 60 °C

column temp (°C)	FLR^a^					MS^b^			
	viral protein (VP)	average area (counts × min)	area CV (%)	VP copies	ratio	viral protein (VP)	average area (counts × min)	VP copies	ratio
25	VP1	3541041.67	1.28	7.99	1.60	VP1	81,572	6.79	1.36
	VP2	2557869.33	4.39	5.77	1.15	VP2	57,274	4.77	0.95
	VP3	19850718.67	0.80	44.77	8.95	VP3	582,232	48.45	9.69
	VP3 prime	656673.33	9.80	1.48	0.30	VP3 prime			
45	VP1	2801034.67	8.01	6.75	1.35	VP1	105,131	7.07	1.41
	VP2	2762226.00	4.24	6.65	1.33	VP2	52,038	3.50	0.70
	VP3	18581684.00	1.35	44.77	8.95	VP3	735,638	49.44	9.89
	VP3 prime	760355.00	11.57	1.83	0.37	VP3 prime			
60	VP1	1885809.00	3.26	5.08	1.02	VP1	79,079	6.99	1.40
	VP2	2907691.67	2.49	7.84	1.57	VP2	35,040	3.10	0.62
	VP3	17013525.67	2.70	45.85	9.17	VP3	565,115	49.92	9.98
	VP3 prime	459231.67	28.61	1.24	0.25	VP3 prime			

a The average areas were calculated using FLR traces with values calculated as an average of the three runs performed at each column temperature. Given that an AAV capsid contains a total of 60 VP copies, the number of VP copies per VP was determined by dividing the total number of VPs by the summed integrated area of VP1, VP2, VP3, and VP3 Prime and then multiplying the result by the area of each respective VP. VP ratios were calculated based on the theoretical ratio of VP1:VP2:VP3 being 1:1:10 as described in the literature. The summed total of 12 theoretical copies was divided by the summed integrated area of VP1, VP2, VP3, and VP3 Prime and then multiplied by the area of each respective VP. Complete calculations can be found in Table S7 of the Supporting Information.

b The average areas were calculated using the area under the XICs of each proteoform identified during MS deconvolution. The areas of all proteoforms of a single VP were summed to represent the VP. An average of these sums across the three runs performed at each column temperature was used to calculate the total average area of each VP. VP copies and VP ratios were then calculated as described for the FLR method. Components in VP3 prime were minimal and thus were not included in this calculation. Complete calculations can be found in Table S8 of the Supporting Information.

#### 3.2.1 Full-Length AAV VP Proteoform Identification

A key facet of a workflow capable of both serotype identification and PTM characterization on an intact level is the ability of the separations platform to also enable VP proteoform identification. Not only does this allow for serotype identification but also the ability to monitor prominent PTMs present as those could impact product efficacy and safety. Initially we tested an MS resolving power of 15k, but after noticing the strong MS signal intensity (Figure 3A) we additionally tested a MS resolving power of 45k to see if the additional resolving power could increase the number of proteoforms identified. Both MS resolving powers were tested at all three column temperatures to see whether the number of proteoforms identified might be influenced by VP separation. Data deconvolution was performed using BioPharma Finder (BPF) Version 4.1, where the three replicate injections at each condition (column temperature and MS resolving power) were processed together (see Table S2 for the BPF processing parameters). Results were filtered as described in the section about full-length AAV capsid protein data processing to ensure that all identifications were valid. Proteoform identifications were assigned using the PTMs identified in the section discussing AAV PTM characterization.

Using an MS resolving power of 15k, unmodified VP1, VP2, and VP3 along with oxidized VP1 (VP1-Ox), oxidized VP2 (VP2-Ox), oxidized VP3 (VP3-Ox), and the A211(Ac)-VP3 variant were identified in separations performed at all three column temperatures (Table 4). Additionally, a feature with a mass of 60173.24 Da was detected during deconvolution analysis of the separations performed at 60 °C, although identification could not be determined based on peptide mapping results. Deconvolution analysis revealed this feature to elute at approximately 19.41 min (mean apex retention time) with a relative abundance of 1.35%.

**Table 4:** AAV2 Viral Protein PTM Features Identified at 45K and 15K MS Resolution Analyzed by HILIC-FLR-MS

AAV capsid variant identity	AAV observed mass (Da)			AAV theoretical mass (Da)	Δmass (ppm)		
	25 °C	45 °C	60 °C^b^		25 °C	45 °C	60 °C^b^
45k MS resolution							
VP1 (1–734)Ac + 1x Ox	81873.42	81873.77	81874.70	81870.89	30.9	35.1	46.4
VP1 (1–734)Ac	81855.10	81855.44	81855.34	81854.90	2.5	6.6	5.4
VP2 (138–734) + 1x Ox	66504.57	66505.84	—c	66503.90	10.0	29.1	—c
VP2 (138–734)	66487.25	66487.49	66487.55	66487.90	9.9	6.2	5.3
VP3 (203–734)Ac + 2x Ox	60006.63	60007.33	60007.61	60005.78	14.2	25.9	30.4
VP3 (203–734)Ac + 1x Ox	59991.52	59992.56	59992.27	59989.78	29.0	46.4	41.4
VP3 (203–734)Ac	59973.40	59973.62	59973.54	59973.78	6.3	2.7	4.1
VP3 (203–734)Ac- ≈ 19.5 Da	59953.83	59953.88	59954.11	59955.77	32.3	31.4	27.6
VP3 (211–734)Ac	59300.16	—c	—c	59301.03	14.7	—c	—c
15k MS resolution							
VP1 (1–734)Ac + 1x Ox^a^	81877.35	81875.45	81873.63	81870.89	78.9	55.7	33.4
VP1 (1–734)Ac	81856.81	81855.91	81856.11	81854.90	23.4	12.4	14.9
VP2 (138–734) + 1x Ox^a^	66507.47	66506.85	66507.42	66503.90	53.6	44.3	52.9
VP2 (138–734)	66487.53	66487.39	66487.82	66487.90	5.6	7.7	1.3
unknown feature	—c	—c	60173.24	—c	—c	—c	—c
VP3 (203–734)Ac + 1x Ox	59991.30	59991.47	59991.78	59989.78	25.2	28.1	33.3
VP3 (203–734)Ac	59972.86	59973.47	59973.49	59973.78	15.4	5.3	4.9
VP3 (211–734)Ac	59301.00	59301.03	59301.56	59301.03	0.5	0.6	9.0

a Poor identification due to high background interference from mobile phase.

b Identifications were found in a minimum of two of the replicates instead of all three replicates.

c — feature was not identified during analysis.

Interestingly, at 15k resolving power, the accuracy of the proteoform identifications varied depending on the column temperature, with the matched mass error (mme) generally smaller at higher temperatures (Table 4). The accuracy of the unmodified VPs was good at all column temperatures with a mme generally ranging from 1 ppm to <20 ppm. Still, the mme of VP3 and VP1 was more accurate at separations performed at 45 °C (VP3: 5.3 ppm and VP1: 12.4 ppm) and 60 °C (VP3: 4.9 ppm and VP1: 14.9 ppm, respectively) compared to separations performed at 25 °C (VP3: 15.4 ppm and VP1: 23.4 ppm), However, the A211(Ac)-VP3 variant was identified more accurately at separations performed at 25 and 45 °C (0.5 and 0.6 ppm, respectively), compared to separations performed at 60 °C (9.0 ppm). Oxidized variants of VP3, VP2, and VP1 were also detected, with separations performed at 45 °C providing the most accurate results. However, the mme for all oxidized variants was high (>20 ppm for all).

Given the strong MS signal intensity observed with an MS resolution of 15k (Figure 3A), we decided to see if a greater MS resolving power could increase the number of proteoforms identified. Keeping in mind that increasing resolving power reduces MS signal intensity, we decided to test an MS resolving power of 45k as the low abundant VP2 and VP1 peaks still showed reasonably strong MS spectra at this resolution (Figure 3B). With a 45k MS resolution, separation performed at 25 °C resulted in the detection of unmodified VP1, VP2, and VP3 as well as VP1-Ox, VP2-Ox, VP3-Ox, VP3 containing 2 oxidations (VP3–2Ox), and the A211(Ac)-VP3 variant. Additionally, a mass of VP3 minus ≈19.5 Da (VP3 – ≈19.5 Da) was also clearly detected. For separations performed at 45 °C, the same proteoforms as 25 °C were identified except for A211(Ac)-VP3, and at 60 °C, all proteoforms except A211(Ac)-VP3 and oxidized VP2 were identified. The reduction of identifications for separations performed at 45 and 60 °C is the result of the reduced signal intensity due to increased MS resolution combined with reduced chromatographic resolution that occurs when separation is performed at higher column temperatures.

**Figure 4:**
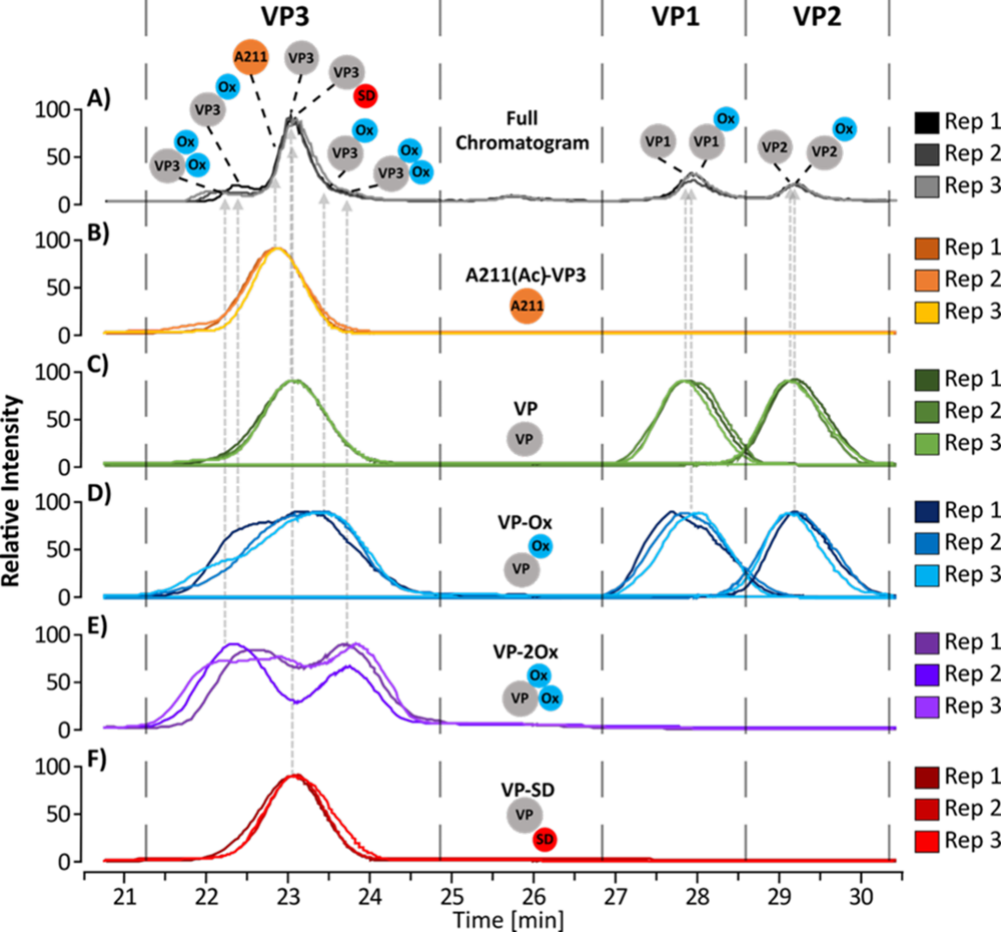
BPF generated deconvolution chromatograms of full-length VP proteoforms identified during data processing of samples separated at 25 °C with a 45k MS resolving power. Multiconsensus processing was performed in BPF to analyze the triplicate injections together. Displayed here are chromatographic overlays of each injection containing (A) Full chromatograms containing all proteoforms (gray); (B) chromatograms of the A211-VP3 variant of VP3 (orange); (C) chromatograms of unmodified VPs (green); (D) chromatograms of VPs containing single oxidation (blue); (E) chromatograms of VPs containing two oxidations (purple); (F) chromatograms of VPs containing a potential succinimide D modification (red). Injection one is represented by the darkest shade of each color, injection two the middle shade, and injection three the lightest shade. Please see the web version of this article for interpretation of color references if necessary.

At 45k MS resolution, the unmodified VP1, VP2, and VP3 proteoforms were detected with a mme below 10 ppm (Table 4), and the A211(Ac)-VP3 variant detected when separations were performed at 25 °C was identified with a mme below 15 ppm (14.7 ppm). The oxidized variants were often detected with a mme >20 ppm, but for separations performed at 25 °C, VP3–2Ox and VP2-Ox had a mme of 14.2 and 10.0 ppm, respectively. The high mme of oxidized VP proteoforms identified at 45k MS resolution as well as at 15k MS resolution is potentially a result of oxidation occurring at both Met and Trp residues, as revealed by peptide mapping. When Met oxidation occurs, the oxidized form is expected to elute after the unmodified form when separation is performed on HILIC.^42^ However, as shown in Figure 4, deconvolution in BPF shows oxidized proteoforms of VP3 (VP3-Ox) eluting over a broad range, including before and after unmodified VP3. This suggests that we are seeing oxidation at different sites on VP3 that cannot be differentiated by the HILIC separation and that some of these oxidized sites are potentially at Trp instead of Met residues.

As previously mentioned, deconvolution analysis of samples analyzed with an MS resolving power of 45k showed the presence of a feature with a mass of VP3- ≈ 19.5 Da, corresponding to a mass of 59953.83, 59953.88, and 59954.11 in analyses performed at 25, 45, and 60 °C, respectively (Table 4). The two most realistic suggestions from the BPF deconvolution results, based on peptide mapping, are the presence of 1× oxidation and 2× succinimide D modifications or the presence of a single succinimide D modification. As discussed earlier, the presence of oxidation would generate a distinct retention time shift from that of unmodified VP3 as well as most likely generate a broad chromatographic peak. However, here, we see a comparatively sharp chromatographic peak with no discernible retention time shift between this mass and unmodified VP3 (Figure 4). There is potential that the ringed formation of the succinimide modifications can counteract the retention time shift that occurs due to the oxidation PTM, as literature has shown that similarly ringed structures have a similar, but opposite retention time shift from that of oxidized PTMs.^43^ However, it is beyond the scope of this study to determine whether this is the case here. The minimal retention time shift can also be indicative of the presence of only succinimide, as previous studies have illustrated that succinimide products only minimally separate from their unmodified counterparts.^17,44^ Only succinimide D was detected during peptide mapping above 1%, so if this is the case, that is, the succinimide is present. Here the 1× succinimide D PTM has a mme of 32.3 ppm. This high mme is a result of the reduced spectral intensity created by the increased MS resolution and its low relative intensity to that of unmodified VP3. Peptide mapping revealed 3 peptides with a Succinimide D modification with an abundance ≥1% (Table 1), all within the VP3 region, though no individual peptide containing the Succinimide D PTM had a relative percent abundance above 6%. Full-length mass analysis indicated that this PTM had a relative abundance of 16–19% unmodified VP3, depending on the column temperature used during LC separation. The exact reason for this discrepancy is not clear. A potential explanation is that the presence of succinimide PTMs below 1% was excluded by our threshold during peptide mapping and that their abundances could generate a greater presence of succinimide than reported. Additionally, the highly acidic nature of the mobile phases due to the presence of DFA might be initiated on column sample deamidation, but the highly acidic environment is more favorable to the succinimide intermediate than the deamidation product. However, further investigation of this is required.

As mentioned during the discussion on AAV VP separation, a small peak, named VP3 prime, was detected between VP3 and VP1. Processing parameters in BPF were modified to try and identify this feature, as it is not detected within the parameters currently utilized (Table S3). Here, the samples separated at 25 °C with an MS resolving power of 45k and the samples separated at 45 °C with an MS resolving power of 15k were reprocessed, as these samples provided the best identifications at their respective MS resolutions. The reprocessed data suggest that this feature contains a mixture of multiple VP3 proteoforms, predominately deamidated forms. At a resolving power of 15k, the prominent feature detected was a deamidated VP3. At a resolving power of 45k, the highest scoring features detected were a deamidated VP3-Ox, a VP3-2Ox, and a deamidated VP3-oxidation to kynurenine. These findings suggest that on-column oxidation or deamidation might be occurring but alone, which is not sufficient to conclude this. Further investigation is required to determine the exact nature of this peak.

When comparing the results of the VP proteoforms identified at 15k MS resolution and 45k MS resolution, the advantages of each method can be seen depending on one’s desired goal. If solely trying to achieve rapid serotype identification, then performing separations at 60 °C using either MS resolution would be the preferred option. Not only are all three VPs detected very accurately, with mmes predominantly below 10 ppm (Table 4), but the early elution of the VPs at 60 °C allows for method optimization to reduce the total run time of each injection. However, if one’s desired goal is to comprehensively characterize a serotype and monitor PTMs identified on intact VPs, then separation performed at 25 °C using a 45k MS resolution is the best option as most VP proteoforms were identified under those analysis conditions. As the desired goal of our work is the development of a workflow that can perform both rapid serotype identity testing and comprehensive characterization of AAV VPs and their PTMs, we used the results from the separation performed at 25 °C using a 45k MS resolution, to develop our data processing workflow.

#### 3.2.2 Rapid AAV Identity Testing and PTM Characterization

**Figure 5:**
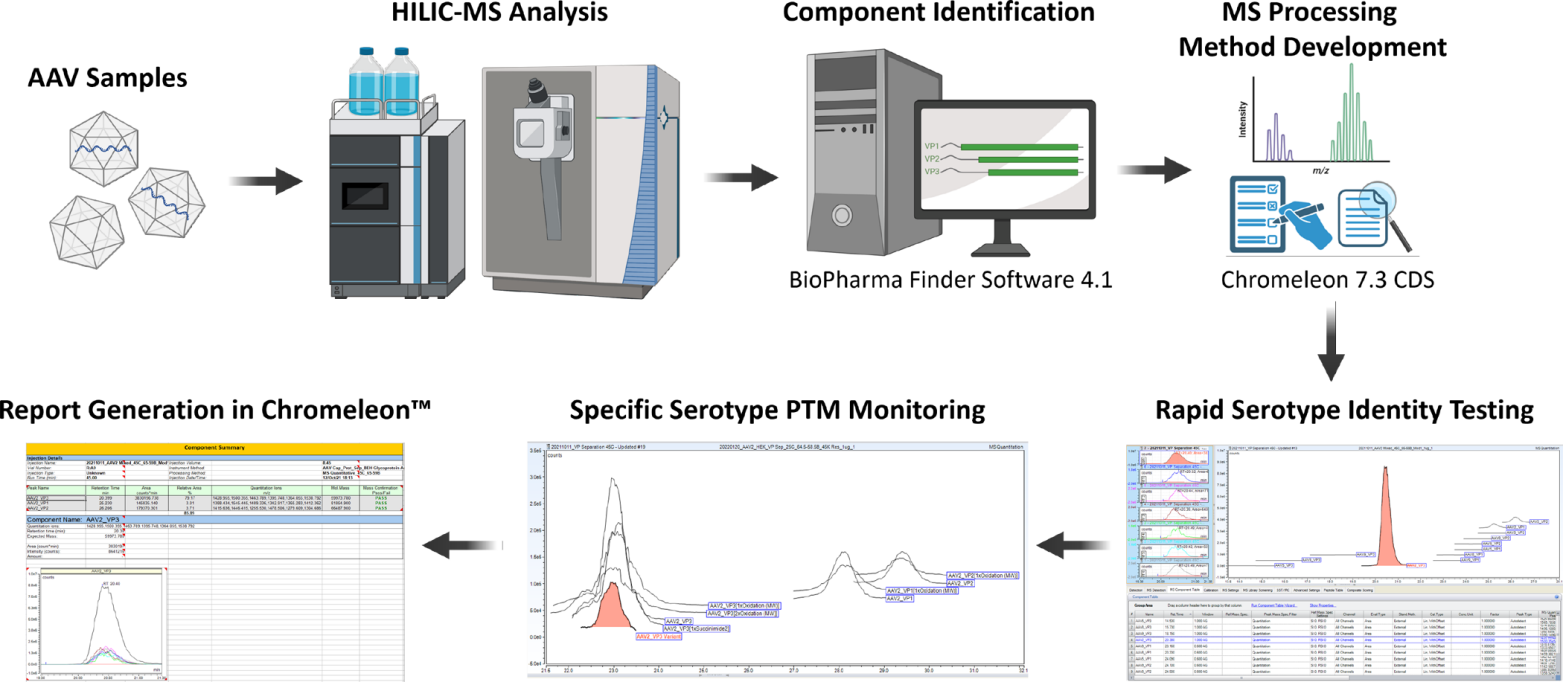
Overview of the developmental process of the platform was created for rapid serotype identification and PTM monitoring. Serotypes are analyzed using HILIC-MS instrumentation controlled by Chromeleon. Components are identified using BioPharma Finder where component workbooks are generated. These workbooks are imported back into Chromeleon where methods for rapid serotype identification and specific serotype PTM monitoring were created and tested. Methods can then be used for release testing and quality control where results can easily be generated within the software.

The selection of a platform capable of VP separations and proteoform identifications is a key first step in the development of a workflow capable of rapid AAV serotype identification and PTM characterization within a QC-compliant setting. With the use of AAV vectors increasing, the importance of such a workflow is imperative to ensuring product quality before release. A workflow that differentiates between different AAV serotypes can enable rapid product identification and verification. A workflow that can additionally perform rapid PTM characterization can help ensure product quality and consistency by monitoring potential changes in VP PTMs. Within Chromeleon, a CFR Part 11 compliant chromatography data system, a dynamic workflow was developed that can be utilized for both rapid serotype identification and VP PTM monitoring (Figure 5). For the development of the serotype identification method, baculovirus/Sf9-derived AAV2, AAV5, AAV8, and AAV9 vector preparations were used (Figure S8). By optimizing data processing, a single universal MS processing method was created that, when applied to all injections, would only detect full-length VPs corresponding to the serotype analyzed in the selected run. This is achieved by using both VP retention times and the generated VP MS spectra to correctly identify each serotype.

While many parameters were optimized, including those within the MS detection, MS settings, and composite scoring tabs in the data processing window (Table S4), optimization of the MS component table was crucial for AAV serotype identification. When the list of VP components is imported from a BPF workbook, Chromeleon imports the observed mass as the molecular mass in the MS component table instead of the theoretical mass. To ensure accurate full-length VP component detection, all molecular masses were changed to the theoretical masses of their respective VPs. The average apex retention time of each VP was then calculated from the three injections of each serotype and used as their respective retention times. For all serotypes, the retention time window of VP3 was set to detect a peak within ±1.00 min of the retention time, while for VP2 and VP1 the retention time window was set to detect a peak at ±0.60 min of the retention time. Matching components within the raw MS data to the features listed in the MS Component table were performed using an algorithm within Chromeleon that considers both the retention time and peak height of a component MS spectrum in the raw data for detection and assigns the highest (most intense) MS component spectrum peak within the retention time window as a component. Only after optimization of the parameters of the components within the MS component table could the other processing parameters be optimized to achieve accurate serotype identification.

Method validation was performed by applying the developed MS quantitative processing method to triplicate injections of a HEK293 cell-derived AAV2 sample, whose VP separation was performed in the same sequence as the baculovirus/Sf9-derived AAVs. All three full-length AAV2 VPs were correctly identified without false positive identifications from other serotypes. To illustrate its adaptability, this method was then applied to select HEK293-derived AAV2 samples analyzed in the section discussing full-length AAV VP proteoform identification. Here, the samples analyzed at 45 °C column temp and 15k MS resolution as well as the samples analyzed at 25 °C and 45k MS resolution were selected. By simply adjusting the expected retention time to correspond with the new retention times obtained under the new experimental conditions, correct identification of the AAV2 VPs was obtained, demonstrating that this method can be quickly and easily modified to accommodate changes in the LC-MS method parameters.

The process described above was then also applied to create a PTM monitoring method allowing further investigation into VP PTMs of a specific serotype (see Table S5 for parameters). Using the HEK293-derived AAV2 samples analyzed at 25 °C and with an MS resolving power of 45k as a proof of concept, we developed and optimized a method for the identification and characterization of full-length VP proteoforms. When put to the test, all VP proteoforms discussed in the section on full-length AAV VP proteoform identification were successfully detected. The ability to characterize the VP variants and proteoforms under each VP peak has the potential to ensure product quality and consistency when used for lot or batch comparisons. It has already been shown that there can be lot-to-lot variation of VP PTMs during production;^4^ therefore, having a QC-ready workflow that can detect such differences is of the utmost importance for product quality control. Once created, the workflow containing both the rapid identity testing method and the PTM monitoring method can be easily applied to sample analysis in Chromeleon without the need to rerun samples. A sample selected during rapid identity testing is simply reprocessed using the PTM monitoring method. Reports detailing the results of either the AAV rapid identity testing method (Supplementary Report 1) or the PTM monitoring method (Supplementary Report 2) can then be generated within Chromeleon for the necessary QC documentation.

## 4 Conclusions

As the utilization of AAV vector-based gene therapies grows, there is a need for regulatory-compliant analytical platforms for quality control and product release assays. Using AAV2 as a test case, we illustrate how performing HILIC-MS with DFA as a mobile phase modifier provides a clear separation of all three full-length AAV VPs while also providing high-quality MS data for AAV characterization. Understanding this, we developed a platform, using the CFR Part 11 compliant software Chromeleon, capable of both rapid serotype identification and serotype-specific PTM monitoring for product quality. Analyzing serotypes AAV2, AAV5, AAV8, and AAV9, we demonstrated how our rapid serotype identification method can be universally applied to accurately determine the serotype in each VP separation analysis, even for runs with modified MS and LC parameters. Using AAV2 as a proof of concept, we then developed a method that can be used to characterize PTMs in different AAV serotypes. Such a method can then be used to monitor PTMs across various production batches to ensure product quality. This method can easily be adopted to other serotypes for broad use in analyzing AAV products. Within Chromeleon both methods can be applied to the same chromatographic runs, because of the separation capabilities and improved spectral quality that DFA as an ion pairing agent provides, allowing both rapid identity testing and PTM characterization within a single analysis. Additionally, being designed within QC-compliant software, this platform enables the necessary easy generation of quality control reports, making it suitable for use within a controlled regulatory environment.

## Supporting Information Available

Detailed AAV SMART digest peptide mapping protocol; BPF 4.1 software parameter settings for peptide mapping analysis; BPF 4.1 software parameter settings for intact mass analysis; BPF 4.1 software parameters for detection of components under VP3 prime peak; rapid AAV identity testing method data processing parameters; AAV PTM monitoring method data processing parameters; acetylated peptides illustrating the presence of the VP3 variant A211(Ac)-L735; sequence coverage map for AAV2 capsid protein peptides; peptide ADNNEGADGVGNSSGNWHCDSTWMGDRVITTSTRT with acetylation at A211; peptide QERLKEDTSFGGNLGRAVF with NH_3_ loss at Q100; peptide QAKKRVLEPLGL with NH_3_ loss at Q119; peptide QDRDVYLQGPIW with NH_3_ loss at Q606; peptide VYLQGPIW with oxidation to kynurenine at W617; peptide LQGPIW with oxidation to kynurenine at W617; FLR traces of VP separations performed using baculovirus/Sf9 derived AAV2, AAV5, AAV8, and AAV9 serotypes; report of AAV rapid identity test generated in Chromeleon; and report of the PTM monitoring method applied to AAV2 generated in Chromeleon (PDF)
Calculations of FLR VP ratios using FLR emission traces; calculations of MS VP ratios using extracted ion chromatograms (XICs) (XLSX)
