# Supplementary material for: Development of a Rapid Adeno-Associated Virus (AAV) Identity Testing Platform through Comprehensive Intact Mass Analysis of Full-Length AAV Capsid Proteins
Source: J Proteome Res. 2023 Dec 20;23(1):161–74. doi: 10.1021/acs.jproteome.3c00513 (PMC10775144; doi:10.1021/acs.jproteome.3c00513)
Supplement: Supplementary file 1 — pr3c00513_si_001.pdf [file pr3c00513_si_001.pdf]

Supporting Information

Development of a Rapid Adeno-Associated Virus (AAV) Identity Testing Platform through Comprehensive Intact Mass Analysis of Full-length AAV Capsid Proteins

Josh Smith[1], Felipe Guapo[1], Lisa Strasser[1], Silvia Millán-Martín[1], Steven Milian[2], Richard Snyder[2], & Jonathan Bones[1,4*]

[1]Characterisation and Comparability Laboratory, The National Institute for Bioprocessing Research and Training, Foster Avenue, Mount Merrion, Co. Dublin, Ireland, A94 X099.

[2]Patheon Viral Vector Services, 13859 Progress Blvd, Alachua, FL 32615, United States.

[3]School of Chemical and Bioprocess Engineering, University College Dublin, Belfield, Dublin 4, Ireland, D04 V1W8.

**Corresponding author*: Jonathan Bones, email: jonathan.bones@nibrt.ie, tel: +353 1215 1800, fax: +353 1215 8116.

TABLE OF CONTENTS

**Supplementary Information 1**	Detailed AAV SMART Digest peptide mapping protocol	S3
**Table S1**	BPF 4.1 software parameter settings for peptide mapping analysis	S4
**Table S2**	BPF 4.1 software parameter settings for intact mass analysis	S5
**Table S3**	BPF 4.1 software parameters for detection of components under VP3 Prime peak	S6
**Table S4**	Rapid AAV identity testing method data processing parameters	S7
**Table S5**	AAV PTM monitoring method data processing parameters	S11
**Table S6**	Acetylated peptides illustrating the presence of the VP3 variant A211(Ac) – L735	S14
**Table S7**	Calculations of FLR VP ratios using FLR emission traces	.XLSX
**Table S8**	Calculations of MS VP ratios using extracted ion chromatograms (XICs)	.XLSX
**Figure S1**	Sequence coverage map for AAV2 capsid protein peptides	S15
**Figure S2**	Peptide *ADNNEGADGVGNSSGNWHCDSTWMGDRVITTSTRT* with acetylation at A211	S21
**Figure S3**	Peptide *QERLKEDTSFGGNLGRAVF* with NH_3 loss at Q100	S22
**Figure S4**	Peptide *QAKKRVLEPLGL* with NH_3 loss at Q119	S23
**Figure S5**	Peptide *QDRDVYLQGPIW* with NH_3 loss at Q606	S24
**Figure S6**	Peptide *VYLQGPIW* with oxidation to kynurenine at W617	S25
**Figure S7**	Peptide *LQGPIW* with oxidation to kynurenine at W617	S26
**Figure S8**	FLR traces of VP separations performed using baculovirus/Sf9 derived AAV2, AAV5, AAV8, and AAV9 serotypes	S27
**Supplementary Report 1**	Report of AAV rapid identity test generated in Chromeleon	S28
**Supplementary Report 2**	Report of PTM monitoring method applied to AAV2 generated in Chromeleon	S239

Supplementary Information 1: Detailed AAV SMART Digest peptide mapping protocol

AAV sample preparation for pepsin digestion on a KingFisher Duo prime

1. For a single digest, 4 µg (35 µL) of AAV sample was transferred to a 500 µL Eppendorf tube and brought to a final volume of 50 µL with LC-MS grade water.
2. For AAV sample denaturation and reduction, 148 µL of SMART Digest™ buffer and 2 µL of TCEP (Bond-Breaker™ solution, 0.5M) were added to the Eppendorf tube containing the AAV sample so that the final solution volume was 200 µL with a final TCEP concentration of 5 mM.
3. In a separate 500 µL tube, 15 µL of SMART Digest™ Pepsin beads and then 100 µL of SMART Digest Buffer™ were added and mixed to create a pepsin bead mixture for digestion
4. In a third 500 µL tube a pepsin bead wash solution was prepared by mixing 50 µL of SMART Digest™ Buffer with 150 µL of LC-MS grade water.
5. All 200 µL of the final AAV sample from Step 2 was transferred into well A1 of a KingFisher™ deepwell 96 plate (Thermo Fisher Scientific, Vantaa, Finland) with subsequently prepared AAV samples transferred into wells A2 and A3. 100 µL of the pepsin bead mixture prepared in Step 3 was added to well D1 of the same deepwell plate, with subsequently prepared pepsin bead mixtures transferred into wells D2 and D3. All 200 µL of the pepsin bead wash solution prepared in Step 4 was then added to well E1 of the aforementioned deepwell plate, with subsequently prepared wash solutions transferred into wells E2 and E3. Finally, a 12-tip comb for microtiter 96 deepwell plate was placed in Row B to complete plate setup for AAV pepsin digestion on a KingFisher™ Duo Prime purification system under the control of Thermo Scientific BindIt software, version 4.0(see plate design below).

King Fisher Plate Design

**Row / Column**	**1**	**2**	**3**	**4**	**5**	**6**	**7**	**8**	**9**	**10**	**11**	**12**
**A:** AAV Sample w/ TCEP (µL)	200	200	200									
**B:** 12-tip cone (Tips)	Tips	Tips	Tips	Tips	Tips	Tips	Tips	Tips	Tips	Tips	Tips	Tips
**C:** (Empty)												
**D:** Pepsin bead mixture (µL)	100	100	100									
**E:** Pepsin bead wash (µL)	200	200	200									
**F**												
**G**												
**H**												

Table S1: BPF 4.1 software parameter settings for peptide mapping analysis

Component Detection Parameters	
Absolute MS Signal Threshold (MS Noise Level * S/N Threshold)	250000
MS Noise Level	1000
S/N Threshold	250
Beginning Peak Width (min)	0.1
Typical Chromatographic Peak Width (min)	0.3
Ending Peak Width (min)	0.75
Maximum Chromatographic Peak Width (min)	0.5
Use Restricted Time	Yes
Time Limits	5 to 115
Relative MS Signal Threshold (% of base peak)	1
Relative Analog Threshold (% of highest peak)	1
Width of Gaussian Filter (represented as 1/n of chromatographic peak width)	3
Minimum Valley to be Considered as Two Chromatographic Peaks (%)	80.00%
Minimum MS Peak Width (Da)	1.2
Maximum MS Peak Width (Da)	4.2
Mass Tolerance (ppm for high-res or Da for low-res)	4
Maximum Retention Time Shift (min)	1
Maximum Mass (Da)	30000
Mass Centroiding Cutoff (% from base)	15
**Identification Parameters**	
Search by Full MS Only	No
Use MS/MS	Use All MS/MS
Maximum Peptide Mass	7000
Mass Accuracy (ppm)	5
Minimum Confidence	0.8
Maximum Number of Modifications for a Peptide	1
Mass Changes for Unspecified Modifications	-129 to 169
Glycosylation	Human
Search for Amino Acid Substitutions	None
Enable Residue Deletion	False
Perform Disulfide Bond Search	No
Protease	Pepsin
N-Term	FIMYWVL
C-Term	CDEFLMTWY
Specificity	High
Static Modifications	None
Variable Modifications	Acetylation (N-term), Acetylation (SideChain), Deamidation (N), Deamidation (Q), Dimethylation (K), Glycation (K), Oxidation (C), Oxidation (M,W), Phosphorylation (S,T,Y), Phopho S (S,T,Y), SUMOylation by SUMO1 (K), SUMOylation by SUMO2 (K), Ubiquitination (K), Amidation (D,E), Methylation (K,R), Formylation (S,T), Double Oxidation (M,W,C), Oxidation to hydroxy-kynurenine (W), Oxidation to kynurenine (W), Double Phosphorylation (S,T,Y), Triple Phosphorylation (S,T,Y), Phosphorylation1x_Oxidation1x (S,T,Y,M,W,C), Cysteaminylation (C), Double Deamidation (N.Q, Succinimide (N), Succinimide (D), NH3 loss_Glutarimide (Q)

Table S2: BPF 4.1 software parameter settings for intact mass analysis

**Component Detection Parameters**	
Chromatogram Parameters	
Use Restricted Time	Enabled
Time Limits	20.4 to 31
m/z Range	1100 to 2100 (15,000 Res)*, 867 to 2100 (45,000 Res)
Chromatogram Trace Type	TIC
Sensitivity	High
Rel. Intensity Threshold (%)	1
Source Spectra Method	Sliding Windows
Sliding Windows Definition	
RT Range	20.4 to 31
Target Avg Spectrum Width	0.6
Target Avg Spectrum Offset Scan	1
Sliding Windows Merging Parameters	
Merge Tolerance	5 ppm
Merge Scheme	Improved Merge Scheme
Max RT Gap	0.1 minutes
Min. Number of Detected Intervals	6
Deconvolution Algorithm	ReSpect™ (Isotopically Unresolved)
ReSpect Main Parameters	
Deconvolution Results Filter	
Output Mass Range	Min 50000 , Max 85000
Deconvoluted Spectra Display Mode	Isotopic Profile (new)
Charge State Distribution	
Deconvolution Mass Tolerance	10 ppm
Choice of Peak Model	
Choice of Peak Model	Intact Protein
Resolution at 400 m/z	
Raw File Specific	
ReSpect Advanced Parameters	
Charge State Distribution	
Model Mass Range	Min 10000 Max 1000000
Charge State Range	5 to 100
Minimum Adjacent Charges (low & high model mass)	4 to 6
Noise Parameters	
Rel. Abundance Threshold (%)	0
Deconvolution Quality	
Quality Score Threshold	0
Choice of Peak Model	
Target Mass	71000 Da
Peak Model Parameters	
Number of Peak Models	1
Left/Right Peak Shape	Left 2 Right 2
Peak Filter Parameters	
Peak Detection Minimum Significance Measure	1
Peak Detection Quality Measure	95%
Specialized Parameters	
Peak Model Width Factor	1
Intensity Threshold Scale	0.01
Deconvolution Parameters	
Noise Compensation	Enabled
Charge Carrier	H+ (1.00727663)
Negative Charge	Disabled
**Identification Parameters**	
Sequence Matching Mass Tolerance	100 ppm (15,000 Res**), 50 ppm (45,000 Res)
Enable Drug-to-Antibody Ratio (Protein Only)	Disabled
Variable Modification Selected	
Multiconsensus Component Merge	
Mass Tolerance	10 ppm
RT Tolerance	0.1 minutes
Minimum Number of Required Occurrences	1

Table S2 Continued:

Sequences, Modifications	
Sequence Name	AAV2_VP1
Static Modifications	Acetylation (N-term)(NTerm,A,x1)
Sequence Name	AAV2_VP2
Static Modifications	None
Sequence Name	AAV2_VP3
Static Modifications	Acetylation (N-term)(NTerm,A,x1)
Sequence Name	AAV2 VP3 Variant
Static Modifications	Acetylation (N-term)(NTerm,A,x1)
Variable Modifications	Acetylation (SideChain), Deamidation (N), Oxidation (M,W), Phosphorylation (S,T,Y), Double Oxidation (M,W,C), Triple Oxidation (M,W,C) Oxidation to kynurenine (W), Double Phosphorylation (S,T,Y), Triple Phosphorylation (S,T,Y), Phosphorylation1x_Oxidation1x (S,T,Y,M,W,C), Double Deamidation (N.Q), Succinimide (D), NH3 loss_Glutarimide (Q)
Max # Modifications	3
Glycosylation	None

* m/z range needed to minimize impact of high background ions in mass spectra from mobile phase

** Needed to expand tolerance from 50 ppm to 100 ppm to detect oxidation on VP2 and VP1

Table S3: BPF 4.1 software parameters for detection of components under VP3 Prime peak*

Component Detection Parameters	
Chromatogram Parameters	
Time Limits	22.5 to 24.0 (45°C, 15,000 Res), 25.2 to 26.7 (25°C, 45,000 Res)
m/z Range	867 to 2100
Sliding Windows Definition	
RT Range	22.5 to 24.0 (45°C, 15,000 Res), 25.2 to 26.7 (25°C, 45,000 Res)
Target Avg Spectrum Width	0.3
Sliding Windows Merging Parameters	
Merge Tolerance	20 ppm
Min. Number of Detected Intervals	3
ReSpect Advanced Parameters	
Specialized Parameters	
Intensity Threshold Scale	0.005

*The BPF 4.1 software parameters used for the determination of components within the VP3 Prime peak are the same as those described in Table S2 for intact mass analysis except for the parameters outlined in the table above.

Table S4: Rapid AAV Identity Testing Method Data Processing Parameters

**MS Detection (Tab)**	
**Extracted Ion Chromatogram**	MS Default Detection Settings
**Detection Algorithm**	Cobra
Parameter: Consider Void Peak	
Retention Time	Initial
Parameter Value	Off
Injection Type	Any
Channel	All Channels
Parameter: Baseline Noise Range	
Retention Time	Initial
Parameter Value	Auto
Injection Type	Any
Channel	All Channels
Parameter: Smoothing Width	
Retention Time	Initial
Parameter Value	0.500 minutes
Injection Type	Any
Channel	All Channels
Parameter: Minimum Area	
Retention Time	0.000 min
Parameter Value	7000 Signal*min
Injection Type	Any
Channel	All Channels
**MS Component Table (Tab)**	
Component: AAV2_VP1	
Retention Time	25.180
Window	0.600 AG
Channel	All Channels
Evaluation Type	Area
Peak Type	Autodetect
MS Quantitation Peaks	1388.43359, 1545.44517, 1489.33590, 1342.91721, 1365.28302, 1412.36224
MS Confirming Peak 1	1388.43359
MS Confirming Peak 2	1545.44517
MS Confirming Peak 3	1489.33590
MS Confirming Peak 4	1342.91721
MS Confirming Peak 5	1365.28302
MS Confirming Peak 6	1412.36224
Display Window	RW
Display Time	RT
Molecular mass	81854.900000
Component: AAV2_VP2	
Retention Time	26.200
Window	0.600 AG
Channel	All Channels
Evaluation Type	Area
Peak Type	Autodetect
MS Quantitation Peaks	1415.63640, 1446.41490, 1255.53048, 1478.50577, 1279.60936, 1304.68622
MS Confirming Peak 1	1415.63640
MS Confirming Peak 2	1446.41490
MS Confirming Peak 3	1255.53048
MS Confirming Peak 4	1478.50577
MS Confirming Peak 5	1279.60936
MS Confirming Peak 6	1304.68622
Display Window	RW
Display Time	RT
Molecular mass	66487.900000
Component: AAV2_VP3	
Retention Time	20.380
Window	1.000 AG
Channel	All Channels
Evaluation Type	Area
Peak Type	Autodetect
MS Quantitation Peaks	1428.95544, 1500.35458, 1463.78919, 1395.74845, 1364.05468, 1538.79198
MS Confirming Peak 1	1428.95544
MS Confirming Peak 2	1500.35458
MS Confirming Peak 3	1463.78919

Table S4 Continued:

MS Confirming Peak 4	**1395.74845**
MS Confirming Peak 5	1364.05468
MS Confirming Peak 6	1538.79198
Display Window	RW
Display Time	RT
Molecular mass	59973.780000
Component: AAV5_VP1	
Retention Time	24.090
Window	0.600 AG
Channel	All Channels
Evaluation Type	Area
Peak Type	Autodetect
MS Quantitation Peaks	1362.62784, 1410.41467, 1435.57674, 1317.99600, 1339.94862, 1488.69741
MS Confirming Peak 1	1362.62784
MS Confirming Peak 2	1410.41467
MS Confirming Peak 3	1435.57674
MS Confirming Peak 4	1317.99600
MS Confirming Peak 5	1339.94862
MS Confirming Peak 6	1488.69741
Display Window	RW
Display Time	RT
Molecular mass	80335.080000
Component: AAV5_VP2	
Retention Time	25.160
Window	0.600 AG
Channel	All Channels
Evaluation Type	Area
Peak Type	Autodetect
MS Quantitation Peaks	1389.99731, 1519.19696, 1484.69186, 1420.19615, 1555.34834, 1451.72711
MS Confirming Peak 1	1389.99731
MS Confirming Peak 2	1519.19696
MS Confirming Peak 3	1484.69186
MS Confirming Peak 4	1420.19615
MS Confirming Peak 5	1555.34834
MS Confirming Peak 6	1451.72711
Display Window	RW
Display Time	RT
Molecular mass	65282.690000
Component: AAV5_VP3	
Retention Time	14.530
Window	1.000 AG
Channel	All Channels
Evaluation Type	Area
Peak Type	Autodetect
MS Quantitation Peaks	1525.66894, 1565.78381, 1608.07850, 1451.30371, 1487.55296, 1416.76686
MS Confirming Peak 1	1525.66894
MS Confirming Peak 2	1565.78381
MS Confirming Peak 3	1608.07850
MS Confirming Peak 4	1451.30371
MS Confirming Peak 5	1487.55296
MS Confirming Peak 6	1416.76686
Display Window	RW
Display Time	RT
Molecular mass	59462.370000
Component: AAV8_VP1	
Retention Time	23.330
Window	0.600 AG
Channel	All Channels
Evaluation Type	Area
Peak Type	Autodetect
MS Quantitation Peaks	1409.08000, 1459.38216, 1385.20869, 1513.38797, 1184.60525, 1339.82767
MS Confirming Peak 1	1409.08000
MS Confirming Peak 2	1459.38216
MS Confirming Peak 3	1385.20869
MS Confirming Peak 4	1513.38797
MS Confirming Peak 5	1184.60525

Table S4 Continued:

MS Confirming Peak 6	**1339.82767**
Display Window	RW
Display Time	RT
Molecular mass	81666.710000
Component: AAV8_VP2	
Retention Time	24.500
Window	0.600 AG
Channel	All Channels
Evaluation Type	Area
Peak Type	Autodetect
MS Quantitation Peaks	1386.80868, 1358.52407, 1331.39296, 1305.30768, 1416.29428, 1623.39744
MS Confirming Peak 1	1386.80868
MS Confirming Peak 2	1358.52407
MS Confirming Peak 3	1331.39296
MS Confirming Peak 4	1305.30768
MS Confirming Peak 5	1416.29428
MS Confirming Peak 6	1623.39744
Display Window	RW
Display Time	RT
Molecular mass	66518.100000
Component: AAV8_VP3	
Retention Time	15.730
Window	1.000 AG
Channel	All Channels
Evaluation Type	Area
Peak Type	Autodetect
MS Quantitation Peaks	1574.80007, 1496.10855, 1534.44183, 1459.64505, 1273.44472, 1391.80234
MS Confirming Peak 1	1574.80007
MS Confirming Peak 2	1496.10855
MS Confirming Peak 3	1534.44183
MS Confirming Peak 4	1459.64505
MS Confirming Peak 5	1273.44472
MS Confirming Peak 6	1391.80234
Display Window	RW
Display Time	RT
Molecular mass	59804.680000
Component: AAV9_VP1	
Retention Time	23.160
Window	0.600 AG
Channel	All Channels
Factor	1.000000
Peak Type	Autodetect
MS Quantitation Peaks	1378.81961, 1333.65071, 1355.86805, 1402.58411, 1534.80787, 1452.63616
MS Confirming Peak 1	1378.81961
MS Confirming Peak 2	1333.65071
MS Confirming Peak 3	1355.86805
MS Confirming Peak 4	1402.58411
MS Confirming Peak 5	1534.80787
MS Confirming Peak 6	1452.63616
Display Window	RW
Display Time	RT
Molecular mass	81290.340000
Component: AAV9_VP2	
Retention Time	24.100
Window	0.600 AG
Channel	All Channels
Evaluation Type	Area
Peak Type	Autodetect
MS Quantitation Peaks	1409.72987, 1162.58873, 1472.33406, 1274.28659, 1380.37065, 1352.23093
MS Confirming Peak 1	1409.72987
MS Confirming Peak 2	1162.58873
MS Confirming Peak 3	1472.33406
MS Confirming Peak 4	1274.28659
MS Confirming Peak 5	1380.37065
MS Confirming Peak 6	1352.23093
Display Window	RW

Table S4 Continued:

Display Time	**RT**
Molecular mass	66209.760000
Component: AAV9_VP3	
Retention Time	18.150
Window	1.000 AG
Channel	All Channels
Evaluation Type	Area
Peak Type	Autodetect
MS Quantitation Peaks	1358.58061, 1390.14960, 1299.55588, 1328.41535, 1271.92993, 1457.90702
MS Confirming Peak 1	1358.58061
MS Confirming Peak 2	1390.14960
MS Confirming Peak 3	1299.55588
MS Confirming Peak 4	1328.41535
MS Confirming Peak 5	1271.92993
MS Confirming Peak 6	1457.90702
Display Window	RW
Display Time	RT
Molecular mass	59732.620000
**MS Settings (Tab)**	
MS Spectra Bunching	
Peak Spectrum Bunch	
Width	1 spectra
Baseline Correction	
No Baseline Correction	Selected
MS Noise Reduction	
Do not reduce noise	Selected
MS Chromatogram Settings	
Mass Precision	
Decimal Places	5
Mass Tolerance	
Mass Tolerance	20 ppm
Integration	
Inhibit integration for TIC channel	Unselected
Smoothing	
Type	Moving Average
Points	21
**Composite Scoring (Tab)**	
Composite Scoring	
Scoring result on checked criteria	
Pass score if at least	1 criteria is passed
Fail score if less than	1 criteria is passed
Indeterminate score if neither passed nor failed	
MS Criteria	
Intact Protein Deconvolution	
Match the following rules	All
(Component.massToChargeRatio-ipdComponent.mass)/component.massToChargeRatio *1000000	Is between -25 and 25

Table S5: AAV PTM Monitoring Method Data Processing Parameters

MS Detection (Tab)	
Parameter: Consider Void Peak	
Retention Time	Initial
Parameter Value	Off
Injection Type	Any
Channel	All Channels
Parameter: Baseline Noise Range	
Retention Time	Initial
Parameter Value	Auto
Injection Type	Any
Channel	All Channels
Parameter: Smoothing Width	
Retention Time	Initial
Parameter Value	0.500 minutes
Injection Type	Any
Channel	All Channels
**MS Component Table (Tab)**	
Component: AAV2_VP1	
Retention Time	28.030
Window	1.000 AG
Channel	All Channels
Evaluation Type	Area
Peak Type	Autodetect
MS Quantitation Peaks	1342.89589, 1388.38818, 1365.27091, 1300.30104, 1187.32055, 1279.99847
MS Confirming Peak 1	1342.89589
MS Confirming Peak 2	1388.38818
MS Confirming Peak 3	1365.27091
MS Confirming Peak 4	1300.30104
MS Confirming Peak 5	1187.32055
MS Confirming Peak 6	1279.99847
Display Window	RW
Display Time	RT
Molecular mass	81854.900000
Component: AAV2_VP1_1xOxidation	
Retention Time	28.030
Window	1.000 AG
Channel	All Channels
Evaluation Type	Area
Peak Type	Autodetect
MS Quantitation Peaks	1343.19118, 1154.15007, 1388.69979, 1187.57166, 1412.61042, 1170.61591
MS Confirming Peak 1	1343.19118
MS Confirming Peak 2	1154.15007
MS Confirming Peak 3	1388.69979
MS Confirming Peak 4	1187.57166
MS Confirming Peak 5	1412.61042
MS Confirming Peak 6	1170.61591
Display Window	RW
Display Time	RT
Molecular mass	81870.890000
Component: AAV2_VP2	
Retention Time	29.340
Window	1.000 AG
Channel	All Channels
Evaluation Type	Area
Peak Type	Autodetect
MS Quantitation Peaks	1446.39156, 1415.62751, 1304.68346, 1478.50307, 1209.87072, 1386.16667
MS Confirming Peak 1	1446.39156
MS Confirming Peak 2	1415.62751
MS Confirming Peak 3	1304.68346
MS Confirming Peak 4	1478.50307
MS Confirming Peak 5	1209.87072
MS Confirming Peak 6	1386.16667
Display Window	RW
Display Time	RT
Molecular mass	66487.900000

Table S5 Continued:

Component: AAV2_VP2_1xOxidation	
Retention Time	29.380
Window	1.000 AG
Channel	All Channels
Evaluation Type	Area
Peak Type	Autodetect
MS Quantitation Peaks	1446.76009, 1305.02224, 1167.73752, 1358.26268, 1147.62767, 1331.10684
MS Confirming Peak 1	1446.76009
MS Confirming Peak 2	1305.02224
MS Confirming Peak 3	1167.73752
MS Confirming Peak 4	1358.26268
MS Confirming Peak 5	1147.62767
MS Confirming Peak 6	1331.10684
Display Window	RW
Display Time	RT
Molecular mass	66503.900000
Component: AAV2_VP3	
Retention Time	23.100
Window	1.000 AG
Channel	All Channels
Evaluation Type	Area
Peak Type	Autodetect
MS Quantitation Peaks	1364.31094, 1500.57013, 1333.96633, 1395.92511, 1463.89207, 1225.37382
MS Confirming Peak 1	1364.31094
MS Confirming Peak 2	1500.57013
MS Confirming Peak 3	1333.96633
MS Confirming Peak 4	1395.92511
MS Confirming Peak 5	1463.89207
MS Confirming Peak 6	1225.37382
Display Window	RW
Display Time	RT
Molecular mass	59973.780000
Component: AAV2_VP3_Variant	
Retention Time	22.890
Window	0.600 AG
Channel	All Channels
Evaluation Type	Area
Peak Type	Autodetect
MS Quantitation Peaks	1447.37441, 957.48841, 1380.09043, 1163.73649, 1099.11976, 973.14452
MS Confirming Peak 1	1447.37441
MS Confirming Peak 2	957.48841
MS Confirming Peak 3	1380.09043
MS Confirming Peak 4	1163.73649
MS Confirming Peak 5	1099.11976
MS Confirming Peak 6	973.14452
Display Window	RW
Display Time	RT
Molecular mass	59301.030000
Component: AAV2_VP3_1xOxidation	
Retention Time	23.370
Window	1.000 AG
Channel	All Channels
Evaluation Type	Area
Peak Type	Autodetect
MS Quantitation Peaks	1277.42805, 1396.17933, 1464.22888, 1250.82261, 1305.18328, 1225.32372
MS Confirming Peak 1	1277.42805
MS Confirming Peak 2	1396.17933
MS Confirming Peak 3	1464.22888
MS Confirming Peak 4	1250.82261
MS Confirming Peak 5	1305.18328
MS Confirming Peak 6	1225.32372
Display Window	RW
Display Time	RT
Molecular mass	59989.780000

Table S5 Continued:

Component: AAV2_VP3_1xSuccinimide2	
Retention Time	23.090
Window	1.000 AG
Channel	All Channels
Evaluation Type	Area
Peak Type	Autodetect
MS Quantitation Peaks	952.67401, 923.37411, 968.01959, 937.78948, 1017.18192, 1000.24990
MS Confirming Peak 1	952.67401
MS Confirming Peak 2	923.37411
MS Confirming Peak 3	968.01959
MS Confirming Peak 4	937.78948
MS Confirming Peak 5	1017.18192
MS Confirming Peak 6	1000.24990
Display Window	RW
Display Time	RT
Molecular mass	59955.770000
Component: AAV2_VP3_2xOxidation	
Retention Time	23.330
Window	1.700 AG
Channel	All Channels
Evaluation Type	Area
Peak Type	Autodetect
MS Quantitation Peaks	1133.19273, 1092.02719, 1112.21724, 1072.51992, 1539.66503, 1464.60605
MS Confirming Peak 1	1133.19273
MS Confirming Peak 2	1092.02719
MS Confirming Peak 3	1112.21724
MS Confirming Peak 4	1072.51992
MS Confirming Peak 5	1539.66503
MS Confirming Peak 6	1464.60605
Display Window	RW
Display Time	RT
Molecular mass	60005.780000
**MS Settings (Tab)**	
MS Spectra Bunching	
Peak Spectrum Bunch	
Width	1 spectra
Baseline Correction	
No Baseline Correction	Selected
MS Noise Reduction	
Do not reduce noise	Selected
MS Chromatogram Settings	
Mass Precision	
Decimal Places	5
Mass Tolerance	
Mass Tolerance	20 ppm
Integration	
Inhibit integration for TIC channel	Unselected
Smoothing	
Type	Moving Average
Points	21
**Composite Scoring (Tab)**	
Composite Scoring	
Scoring result on checked criteria	
Pass score if at least	1 criteria is passed
Fail score if less than	1 criteria is passed
Indeterminate score if neither passed nor failed	
MS Criteria	
Intact Protein Deconvolution	
Match the following rules	All
(Component.massToChargeRatio-ipdComponent.mass)/component.massToChargeRatio *1000000	Is between -35 and 35

Table S6: Acetylated peptides illustrating the presence of the VP3 variant A211(Ac) – L735*

Peptide Sequence	Positions	Modification	Site	Delta (ppm)	Confidence Score	ID Type	RT	M/Z	Charge State	Mono Mass Exp.	Mono Mass Theo.
**ADNNEGADGVGNSSGNWHCDSTWMGDRVIT**	211-240	Acetylation	A211	-0.22	100	MS2	59.42	1070.445	3	3206.3047	3206.3054
**ADNNEGADGVGNSSGNWHCDSTWMGDRVITT**	211-241	Acetylation	A211	-1.2	100	MS2	59.67	1103.792	3	3307.3491	3307.3531
**ADNNEGADGVGNSSGNWHCDSTWMGDRVITTSTRT**	211-245	Acetylation	A211	-0.14	100	MS2	52.64	939.403	4	3752.5811	3752.5816
**ADNNEGADGVGNSSGNWHCDSTWMGDRVITTSTRT**	211-245	Acetylation	A211	-0.33	100	MS2	52.65	1252.535	3	3752.5803	3752.5816
**ADNNEGADGVGNSSGNWHCDSTWMGDRVITTSTRTWALPTYNNHL**	211-255	Acetylation	A211	0.81	100	MS2	62.45	1242.304	4	4962.1787	4962.1747
**ADNNEGADGVGNSSGNWHCDSTWMGDRVITTSTRTWALPTYNNHL**	211-255	Acetylation	A211	0.72	100	MS2	62.45	993.843	5	4962.1782	4962.1747

*Peptides and acetylation PTM were identified using BioPharma Finder

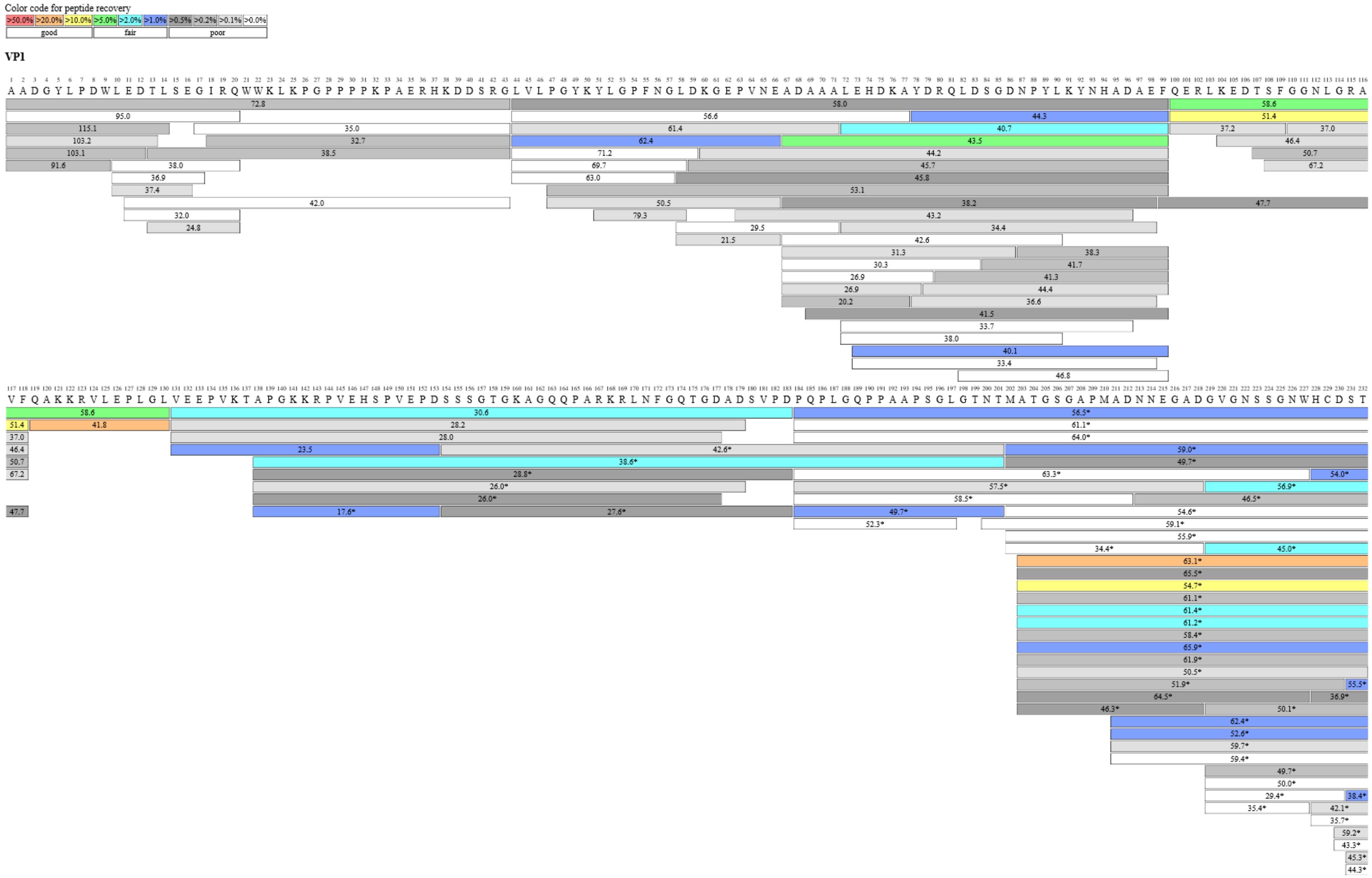

Figure S1: Sequence coverage map for AAV2 capsid protein peptides. Peptides are separated on a PepMap nano RSLC C18 column after pepsin digestion. The coloured bars show the identified peptides, with the numbers in the bars reflecting the retention time. The different colours indicate the peptide recovery in the MS1 scan as illustrated in the "Colour code for peptide recovery" box.

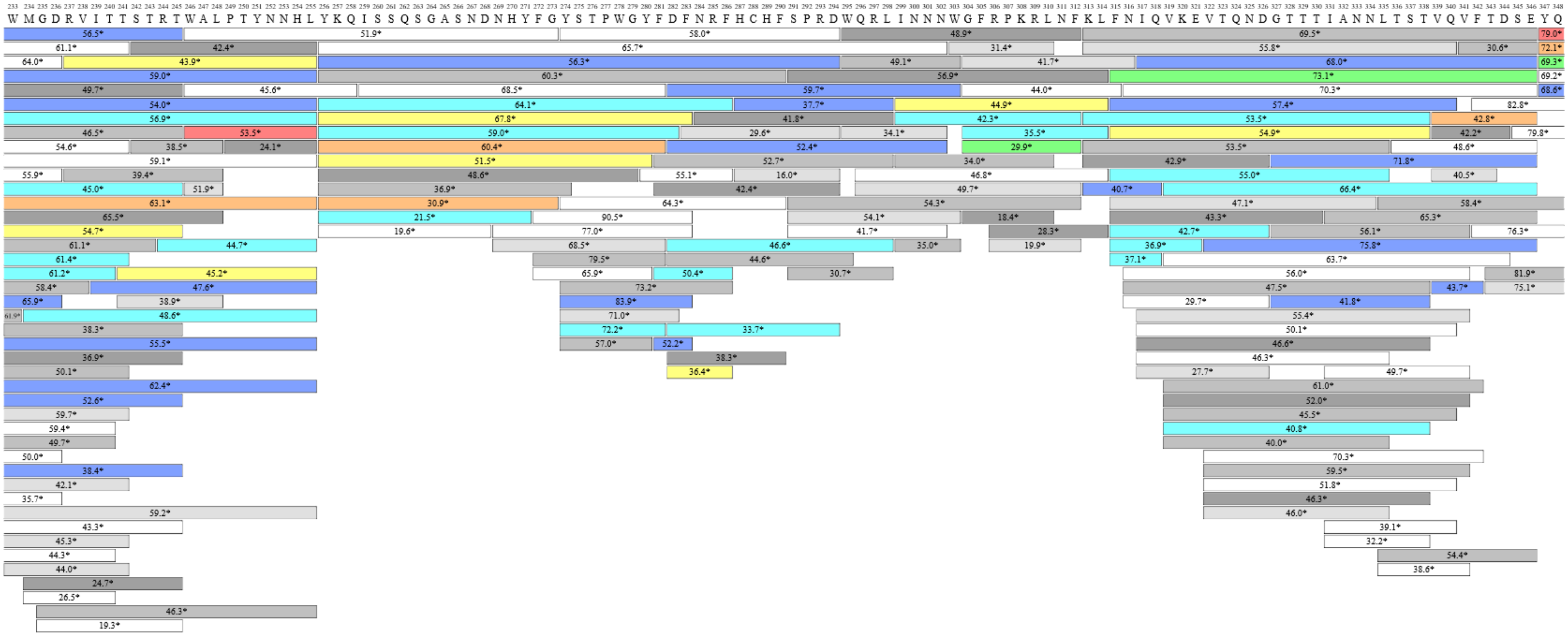

Figure S1 Continued:

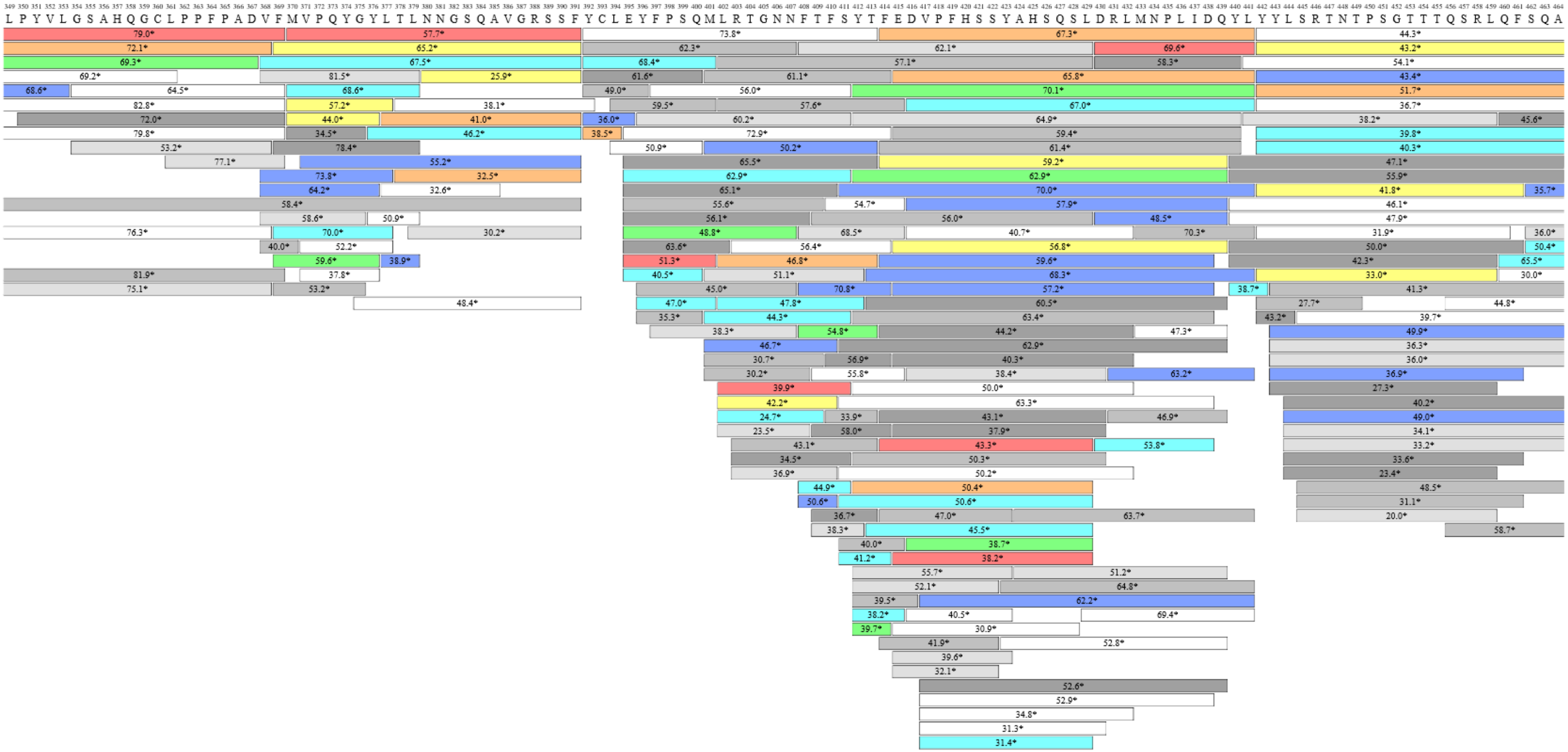

Figure S1 Continued:

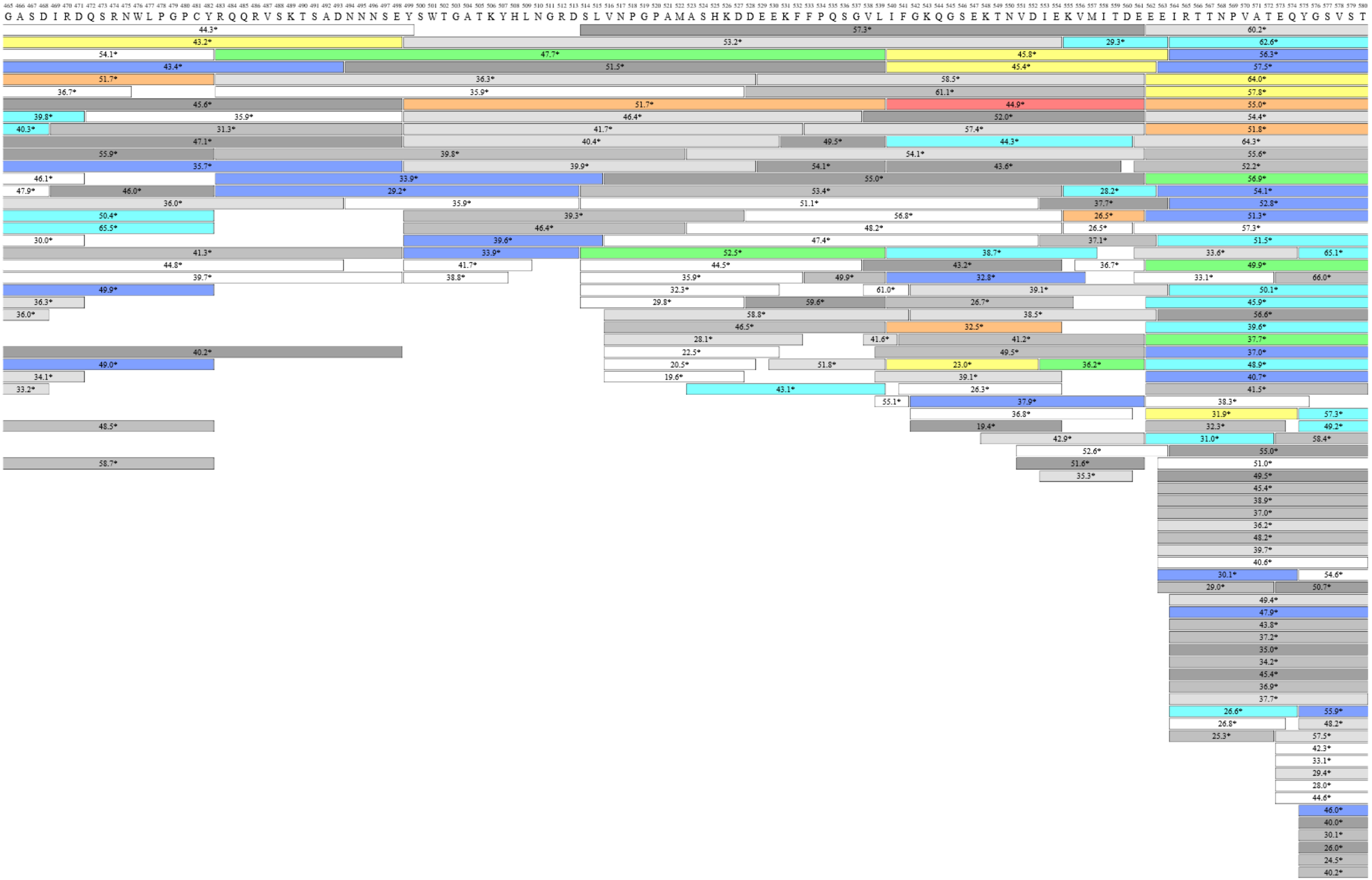

Figure S1 Continued:

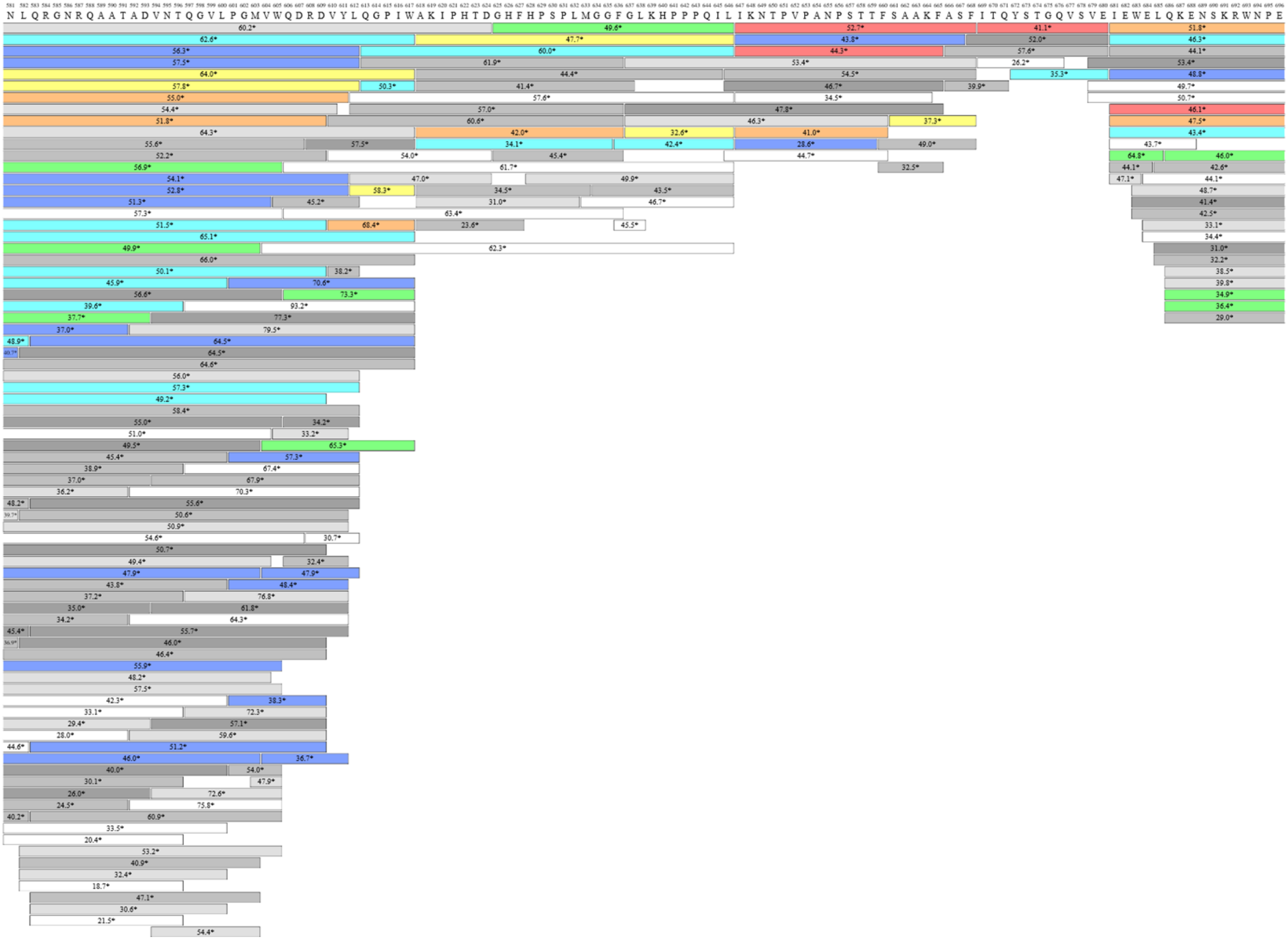

Figure S1 Continued:

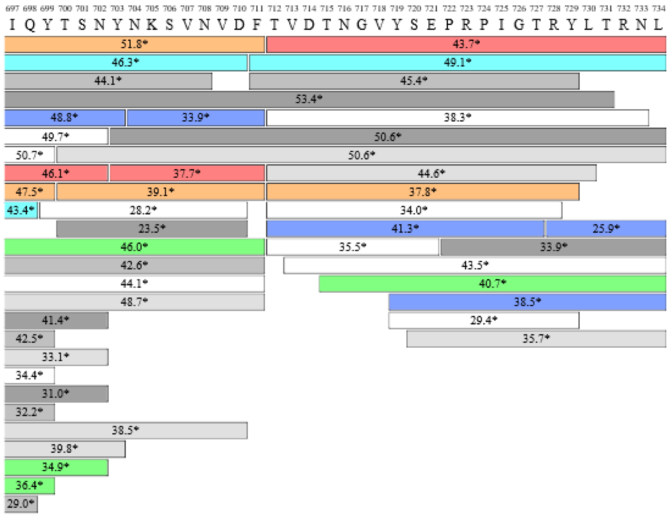

Figure S1 Continued:

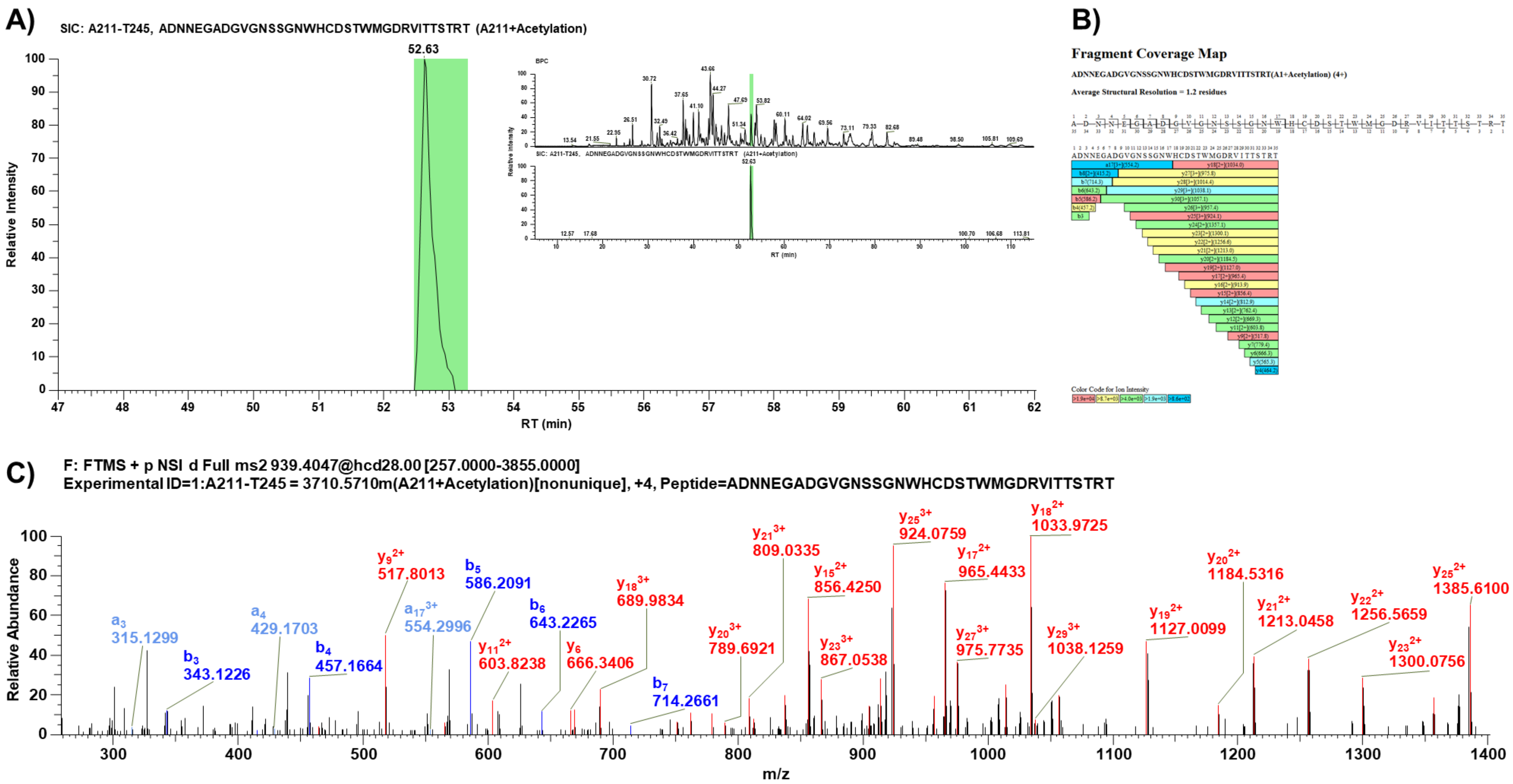

Figure S2: Peptide *ADNNEGADGVGNSSGNWHCDSTWMGDRVITTSTRT* with acetylation at A211. Peptide and PTM are identified using BioPharma Finder (BPF) software. Peptide is representative of the peptides detected in BPF that indicate the presence of the VP3 variant A211(Ac) – L735. A) Selected ion chromatogram (SIC) of acetylated peptide (insert shows SIC in relation to base peak chromatogram (BPC)). B) Peptide fragment coverage map. C) MS/MS spectrum with diagnostic fragment ion assignments.

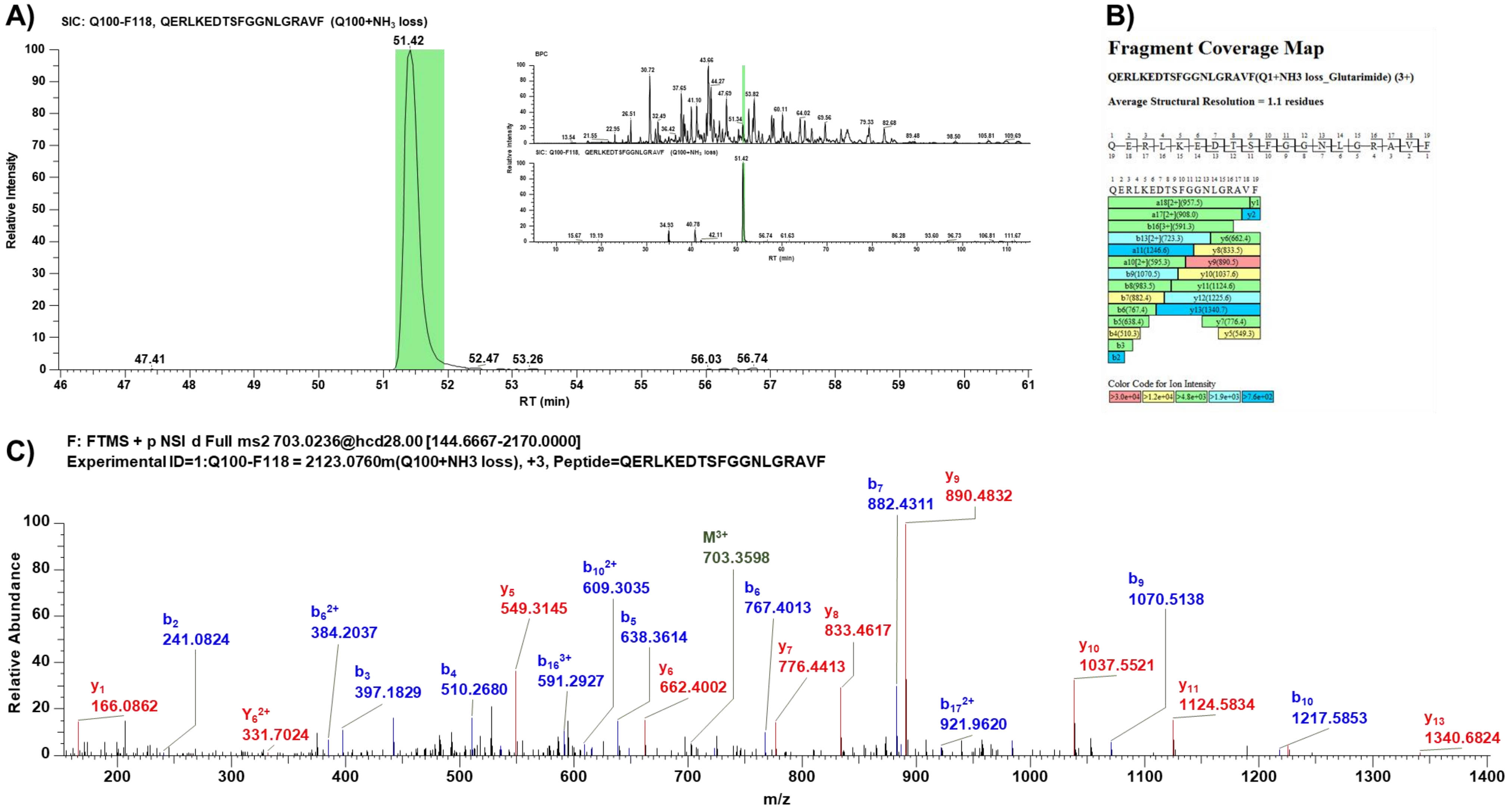

Figure S3: Peptide *QERLKEDTSFGGNLGRAVF* with NH_3 loss at Q100. Peptide and PTM identified using BioPharma Finder software. A) Selected ion chromatogram (SIC) of peptide containing the NH_3 loss PTM (insert shows SIC in relation to base peak chromatogram (BPC)). B) Peptide fragment coverage map. C) MS/MS spectrum with diagnostic fragment ion assignments.

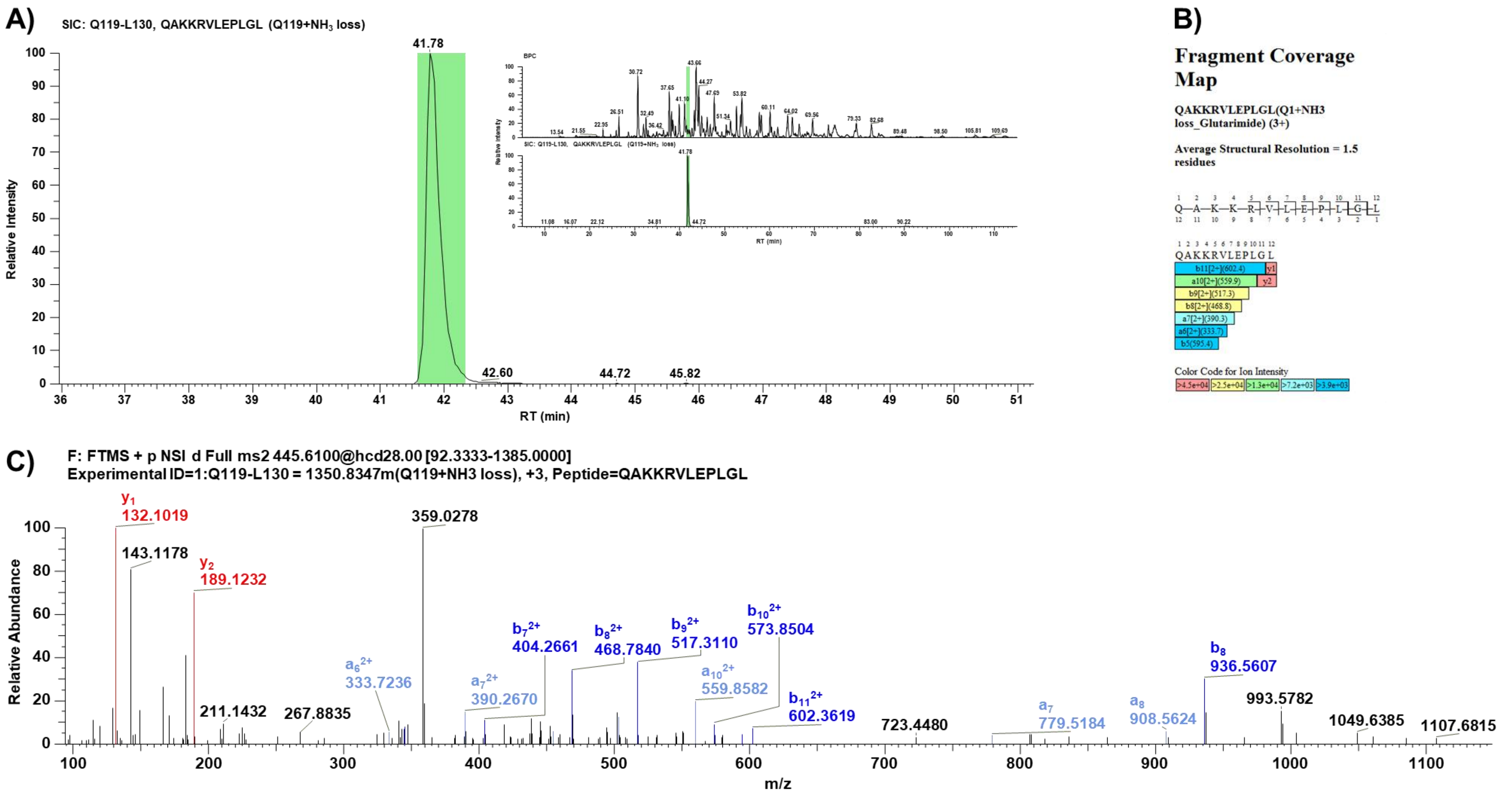

Figure S4: Peptide *QAKKRVLEPLGL* with NH_3 loss at Q119. Peptide and PTM identified using BioPharma Finder software. A) Selected ion chromatogram (SIC) of peptide containing the NH_3 loss PTM (insert shows SIC in relation to base peak chromatogram (BPC)). B) Peptide fragment coverage map. C) MS/MS spectrum with diagnostic fragment ion assignments.

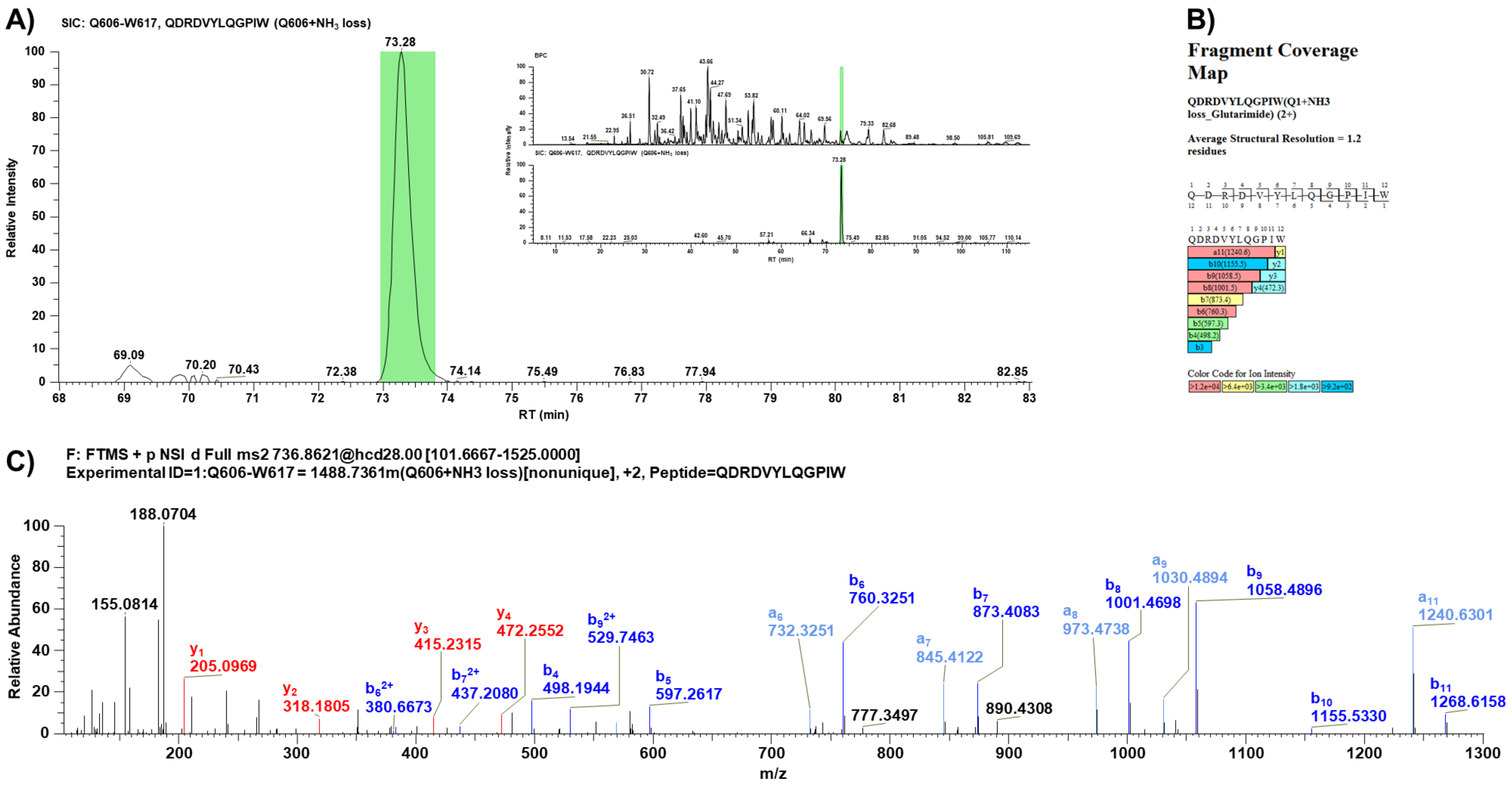

Figure S5: Peptide *QDRDVYLQGPIW* with NH_3 loss at Q606. Peptide and PTM identified using BioPharma Finder software. A) Selected ion chromatogram (SIC) of peptide containing the NH_3 loss PTM (insert shows SIC in relation to base peak chromatogram (BPC)). B) Peptide fragment coverage map. C) MS/MS spectrum with diagnostic fragment ion assignments.

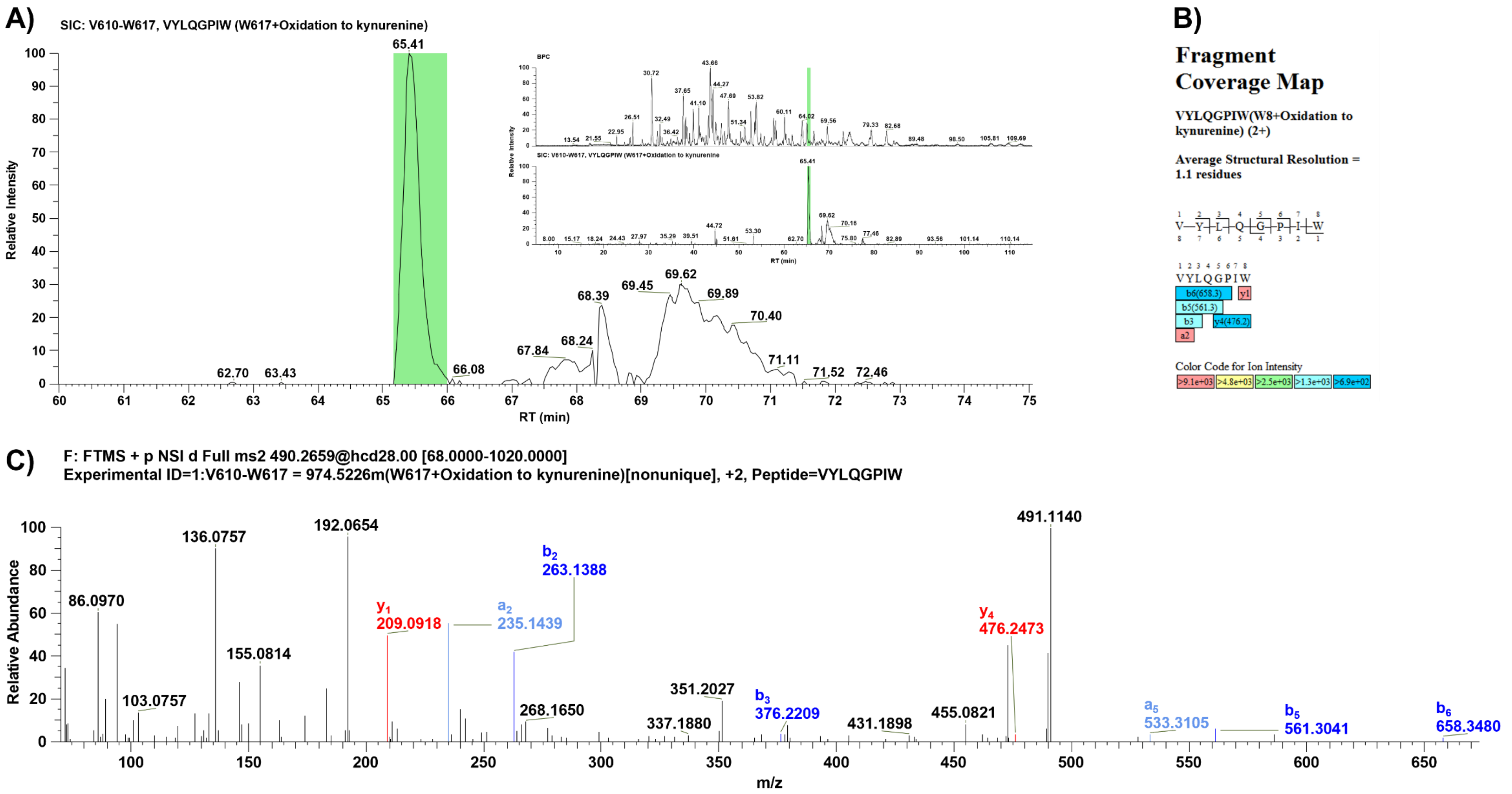

Figure S6: Peptide *VYLQGPIW* with oxidation to kynurenine at W617. Peptide and PTM identified using BioPharma Finder Software. A) Selected ion chromatogram (SIC) of peptide containing the oxidation to kynurenine PTM (insert shows SIC in relation to base peak chromatogram (BPC)). B) Peptide fragment coverage map. C) MS/MS spectrum with diagnostic fragment ion assignments.

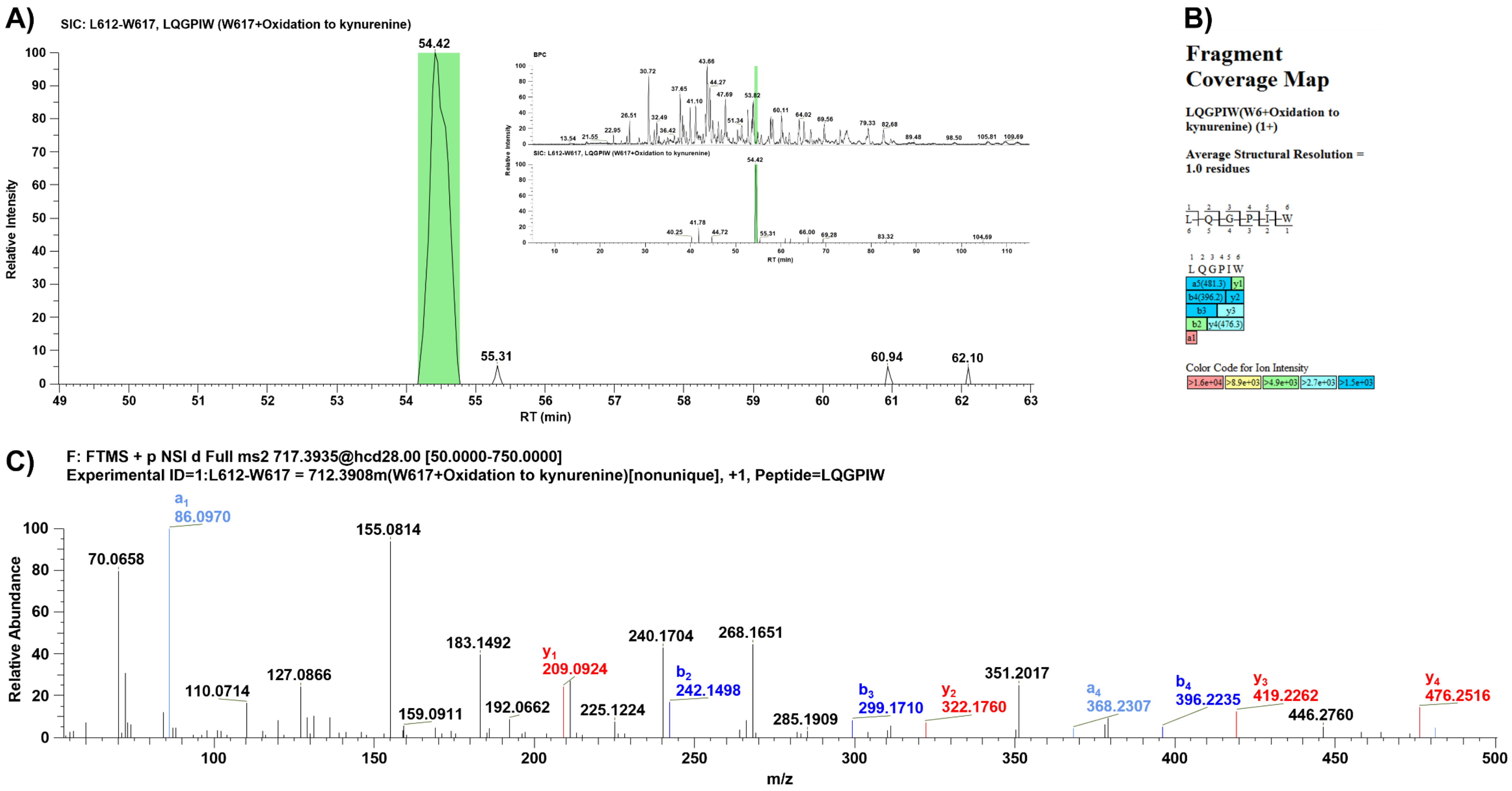

Figure S7: Peptide *LQGPIW* with oxidation to kynurenine at W617. Peptide and PTM identified using BioPharma Finder Software. A) Selected ion chromatogram (SIC) of peptide containing the oxidation to kynurenine PTM (insert shows SIC in relation to base peak chromatogram (BPC)). B) Peptide fragment coverage map. C) MS/MS spectrum with diagnostic fragment ion assignments.

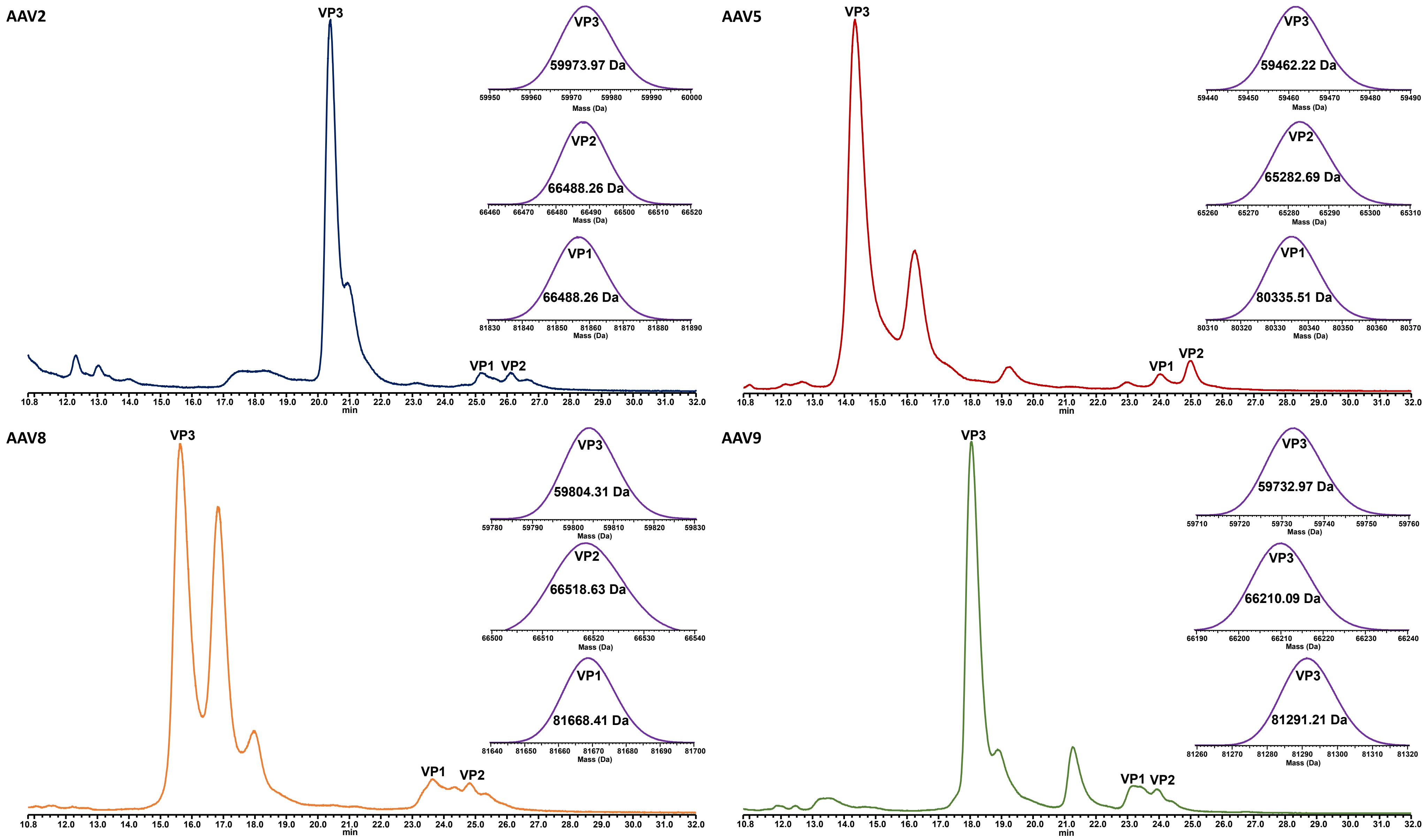

Figure S8: FLR traces of VP separations performed using baculovirus/Sf9 derived AAV2, AAV5, AAV8, and AAV9 serotypes. Serotypes were analysed to help develop a universal processing method for serotype identification. The development of this processing method is a part of the creation of a data processing workflow capable of rapid serotype identification and PTM characterization. The peaks representing the unmodified VP proteoforms are labelled as those masses (along with their respective retention times) were used to differentiate the serotypes. Deconvoluted spectra of each VP for their respective serotypes are shown on the right side of each FLR trace.

Supplementary Report 1:

Report of AAV rapid identity test generated in Chromeleon

Protein Deconvolution Summary

Injection Details

Injection Name:	**20211011_AAV9 E_45C_65-59B_Mod1_1ug_1**	*Injection Volume:*	**2.80**
Vial Number:	**R:A5**	*Instrument Method:*	**AAV Cap_Prot_Sep_BEH Glycoprotein Amide H**
Injection Type:	**Unknown**	*Processing Method:*	**MS Quantitative_45C_65-59B**
Run Time (min):	**45.00**	*Injection Date/Time:*	**12/Oct/21 14:28**

Chromatogram

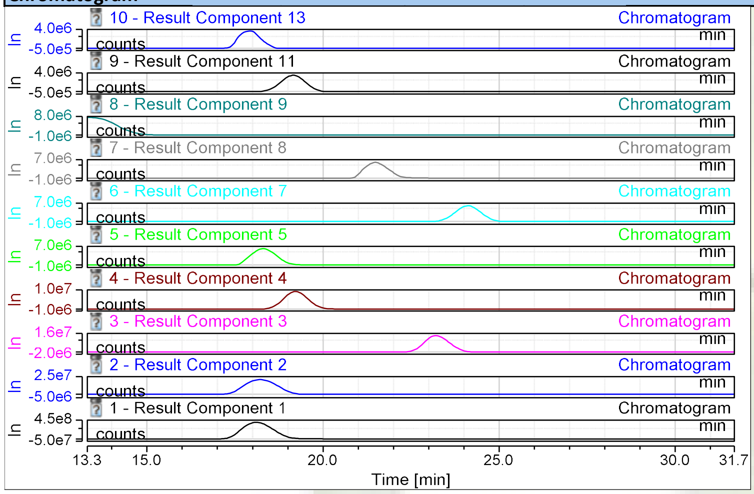

Source Spectrum

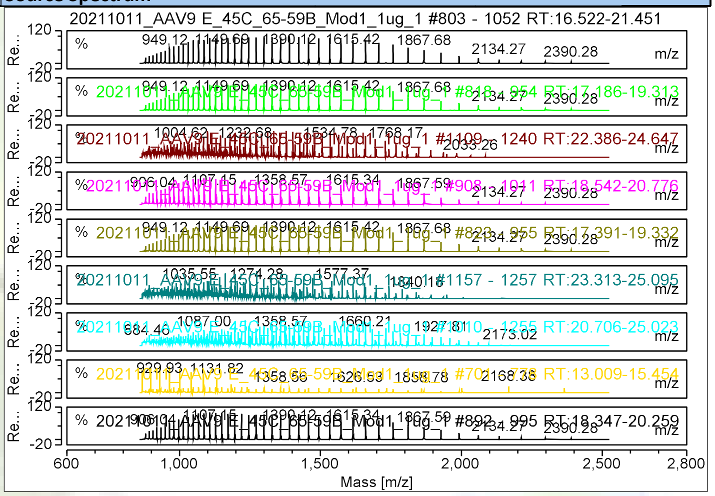

Deconvoluted Spectrum

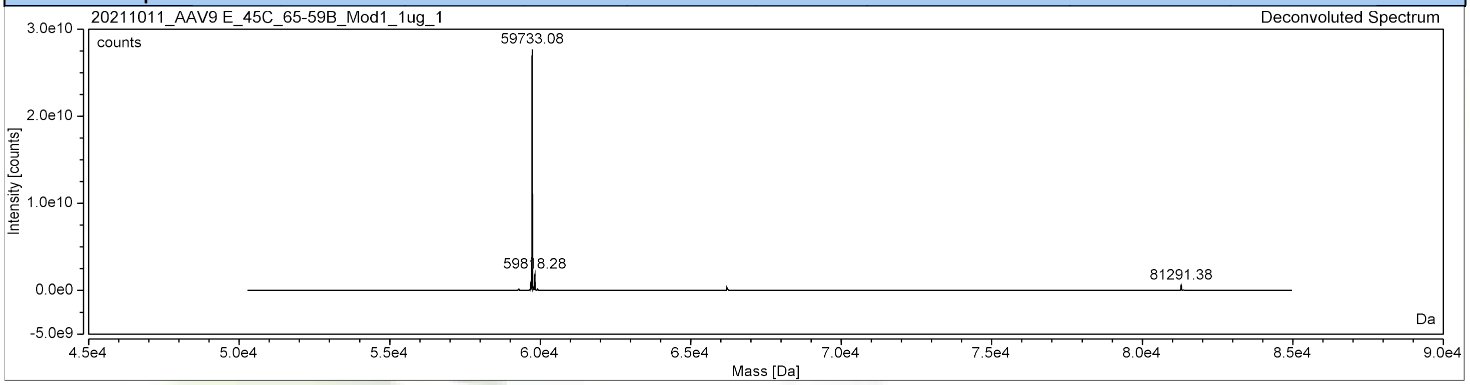

Deconvolution Results

Result Component	RT min	Mass Da	Std. Dev. Da	Std. Dev. ppm	Delta Mass Da	Intensity counts	Fractional Abundance counts	Rel. Abundance %	Number of Charge States	Score
Result Component 1	18.11	59733.033	0.40	6.68	0.000	27034264760	80.1	100.00	45	238.14
Result Component 2	18.22	59820.302	0.95	15.91	87.269	1527586438	4.5	5.65	40	170.79
Result Component 3	23.18	81291.415	0.91	11.13	21558.382	643910272	1.9	2.38	56	291.15
Result Component 4	19.20	59813.944	0.85	14.29	80.911	471832605	1.4	1.75	43	234.57
Result Component 5	18.27	59686.960	1.22	20.37	-46.073	390164875	1.2	1.44	32	176.14
Result Component 7	24.10	66210.268	0.85	12.85	6477.235	340089971	1.0	1.26	44	239.41
Result Component 8	21.47	59731.938	0.90	15.10	-1.095	300593658	0.9	1.11	41	217.18
Result Component 9	13.31	59733.070	0.64	10.75	0.037	298107673	0.9	1.10	42	257.50
Result Component 11	19.18	59933.627	1.10	18.41	200.594	200302659	0.6	0.74	40	199.85
Result Component 13	17.92	59687.388	1.55	25.99	-45.645	162773683	0.5	0.60	31	170.85
Result Component 15	21.21	59731.785	0.70	11.70	-1.249	129722977	0.4	0.48	41	225.58
Result Component 17	19.13	59766.513	1.08	18.11	33.479	91587642	0.3	0.34	38	197.64
Result Component 18	23.54	81372.238	1.92	23.54	21639.205	84227912	0.2	0.31	50	158.32
Result Component 19	18.85	59816.211	1.65	27.63	83.178	73795145	0.2	0.27	39	170.82
Result Component 20	19.45	59690.811	1.05	17.65	-42.222	72092544	0.2	0.27	37	195.30
Result Component 21	24.66	66290.558	1.28	19.26	6557.525	43412692	0.1	0.16	41	172.17

© 2009-2020 Thermo Fisher Scientific Inc. All rights reserved.
Chromeleon 7.3.0.60919

Component Summary

Injection Details

Injection Name:	**20211011_AAV9 E_45C_65-59B_Mod1_1ug**	*Injection Volume:*	**2.80**
Vial Number:	**R:A5**	*Instrument Method:*	**AAV Cap_Prot_Sep_BEH Glycoprotein Amide HILIC_1x150mm**
Injection Type:	**Unknown**	*Processing Method:*	**MS Quantitative_45C_65-59B**
Run Time (min):	**45.00**	*Injection Date/Time:*	**12/Oct/21 14:28**

Peak Name	Retention Time min	Area counts*min	Relative Area %	Quantitation Ions m/z	Mol.Mass	Mass Confirmation Pass/Fail
AAV9_VP3	18.129	5305032.777	88.65	1358.581,1390.150,1299.556,1328.415,1271.930,1457.907	59732.620	PASS
AAV9_VP1	23.207	106915.843	1.79	1378.820,1333.651,1355.868,1402.584,1534.808,1452.636	81290.340	PASS
AAV9_VP2	24.049	57302.637	0.96	1409.730,1162.589,1472.334,1274.287,1380.371,1352.231	66209.760	PASS
			**91.39**			

Component Name: AAV5_VP3

Quantitation ions:	1525.669,1565.784,1608.079,1451.304,1487.553,1416.767
Retention time (min):	14.53
Expected Mass:	59462.370
Area (count*min):	-
Intensity (counts):	-
Amount:	-

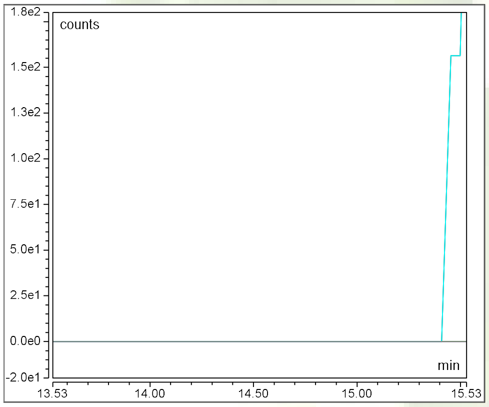

© 2009-2020 Thermo Fisher Scientific Inc. All rights reserved.
Chromeleon 7.3.0.60919

Component Name:	AAV8_VP3
Quantitation ions:	1574.800,1496.109,1534.442,1459.645,1273.445,1391.802
Retention time (min):	15.73
Expected Mass:	59804.680
Area (count*min):	-
Intensity (counts):	-
Amount:	-

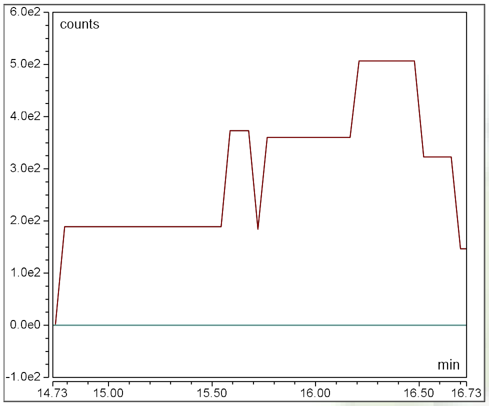

© 2009-2020 Thermo Fisher Scientific Inc. All rights reserved.
Chromeleon 7.3.0.60919

Component Name:	AAV9_VP3
Quantitation ions:	1358.581,1390.150,1299.556,1328.415,1271.930,1457.907
Retention time (min):	18.15
Expected Mass:	59732.620
Area (count*min):	5305033
Intensity (counts):	11472987
Amount:	-

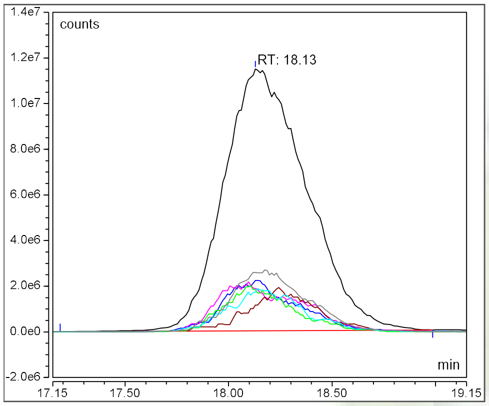

© 2009-2020 Thermo Fisher Scientific Inc. All rights reserved.
Chromeleon 7.3.0.60919

Component Name:	AAV2_VP3
Quantitation ions:	1428.955,1500.355,1463.789,1395.748,1364.055,1538.792
Retention time (min):	20.38
Expected Mass:	59973.780
Area (count*min):	-
Intensity (counts):	-
Amount:	-

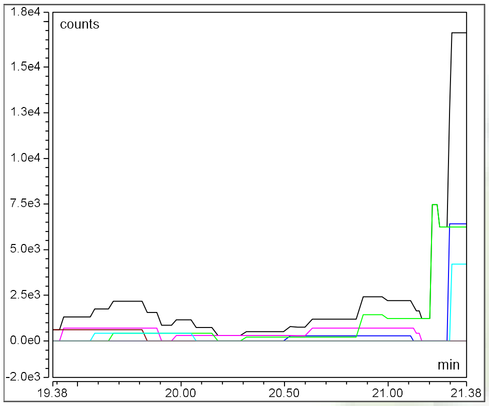

© 2009-2020 Thermo Fisher Scientific Inc. All rights reserved.
Chromeleon 7.3.0.60919

Component Name:	AAV9_VP1
Quantitation ions:	1378.820,1333.651,1355.868,1402.584,1534.808,1452.636
Retention time (min):	23.16
Expected Mass:	81290.340
Area (count*min):	106916
Intensity (counts):	253171
Amount:	-

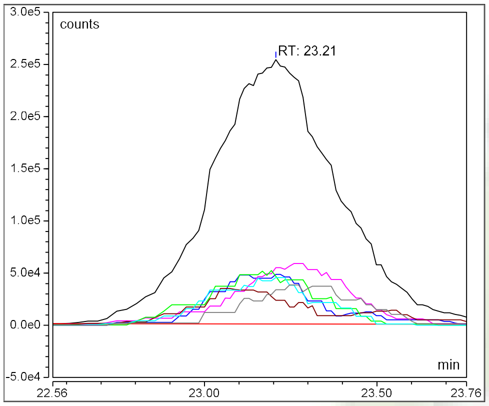

© 2009-2020 Thermo Fisher Scientific Inc. All rights reserved.
Chromeleon 7.3.0.60919

Component Name:	AAV8_VP1
Quantitation ions:	1409.080,1459.382,1385.209,1513.388,1184.605,1339.828
Retention time (min):	23.33
Expected Mass:	81666.710
Area (count*min):	-
Intensity (counts):	-
Amount:	-

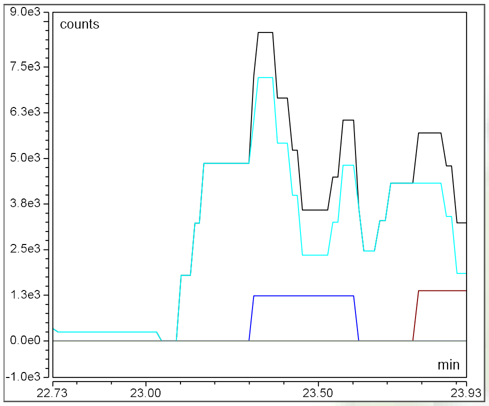

© 2009-2020 Thermo Fisher Scientific Inc. All rights reserved.
Chromeleon 7.3.0.60919

Component Name:	AAV5_VP1
Quantitation ions:	1362.628,1410.415,1435.577,1317.996,1339.949,1488.697
Retention time (min):	24.09
Expected Mass:	80335.080
Area (count*min):	-
Intensity (counts):	-
Amount:	-

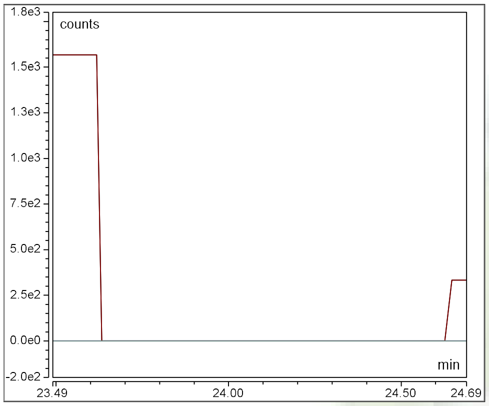

© 2009-2020 Thermo Fisher Scientific Inc. All rights reserved.
Chromeleon 7.3.0.60919

Component Name:	AAV9_VP2
Quantitation ions:	1409.730,1162.589,1472.334,1274.287,1380.371,1352.231
Retention time (min):	24.10
Expected Mass:	66209.760
Area (count*min):	57303
Intensity (counts):	153294
Amount:	-

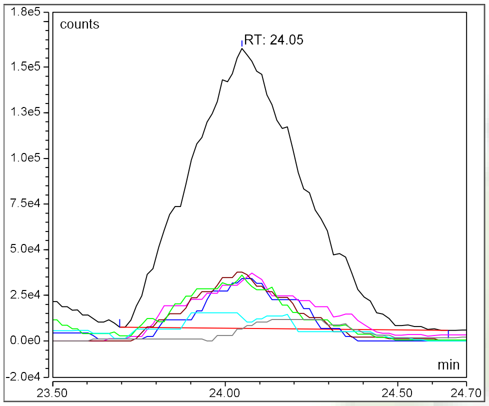

© 2009-2020 Thermo Fisher Scientific Inc. All rights reserved.
Chromeleon 7.3.0.60919

Component Name: AAV8_VP2	
Quantitation ions:	1386.809,1358.524,1331.393,1305.308,1416.294,1623.397
Retention time (min):	24.50
Expected Mass:	66518.100
Area (count*min):	-
Intensity (counts):	-
Amount:	-

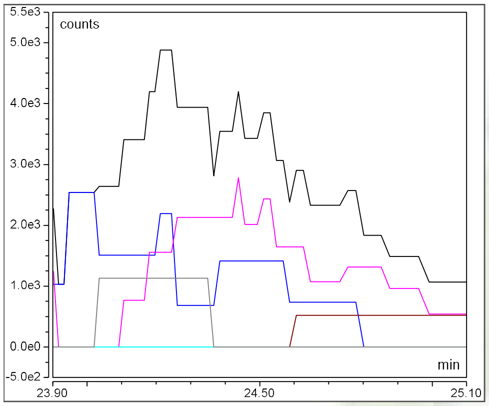

© 2009-2020 Thermo Fisher Scientific Inc. All rights reserved.
Chromeleon 7.3.0.60919

Component Name: AAV5_VP2	
Quantitation ions:	1389.997,1519.197,1484.692,1420.196,1555.348,1451.727
Retention time (min):	25.16
Expected Mass:	65282.690
Area (count*min):	-
Intensity (counts):	-
Amount:	-

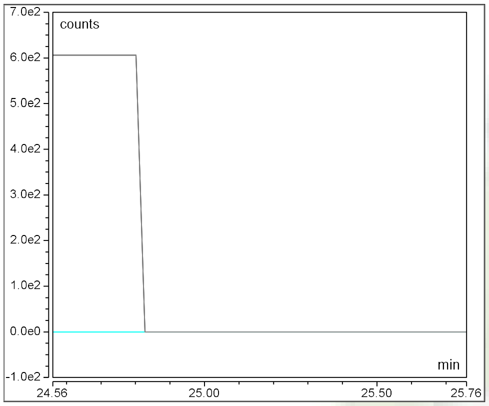

© 2009-2020 Thermo Fisher Scientific Inc. All rights reserved.
Chromeleon 7.3.0.60919

Component Name: AAV2_VP1

Quantitation ions:	1388.434,1545.445,1489.336,1342.917,1365.283,1412.362
Retention time (min):	25.18
Expected Mass:	81854.900
Area (count*min):	-
Intensity (counts):	-
Amount:	-

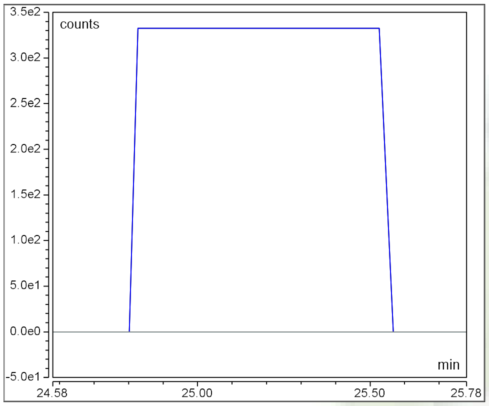

© 2009-2020 Thermo Fisher Scientific Inc. All rights reserved.
Chromeleon 7.3.0.60919

Component Name:	AAV2_VP2
Quantitation ions:	1415.636,1446.415,1255.530,1478.506,1279.609,1304.686
Retention time (min):	26.20
Expected Mass:	66487.900
Area (count*min):	-
Intensity (counts):	-
Amount:	-

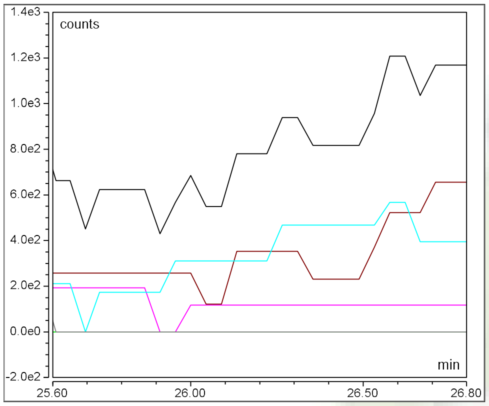

© 2009-2020 Thermo Fisher Scientific Inc. All rights reserved.
Chromeleon 7.3.0.60919

Chromatogram and SST Results

Injection Details

Injection Name:	**20211011_AAV9 E_45C_65-59B_Mod1_1ug_1**	*Run Time (min):*	**45.00**
Vial Number:	**R:A5**	*Injection Volume:*	**2.80**
Injection Type:	**Unknown**		
Calibration Level:			
Instrument Method:	**AAV Cap_Prot_Sep_BEH Glycoprotein Amide HILIC_1x150mm_FLR + MS_CT45C_65-59_S**		
Processing Method:	**MS Quantitative_45C_65-59B**	*Dilution Factor:*	**1.0000**
Injection Date/Time:	**12/Oct/21 14:28**	*Sample Weight:*	**1.0000**

Chromatogram

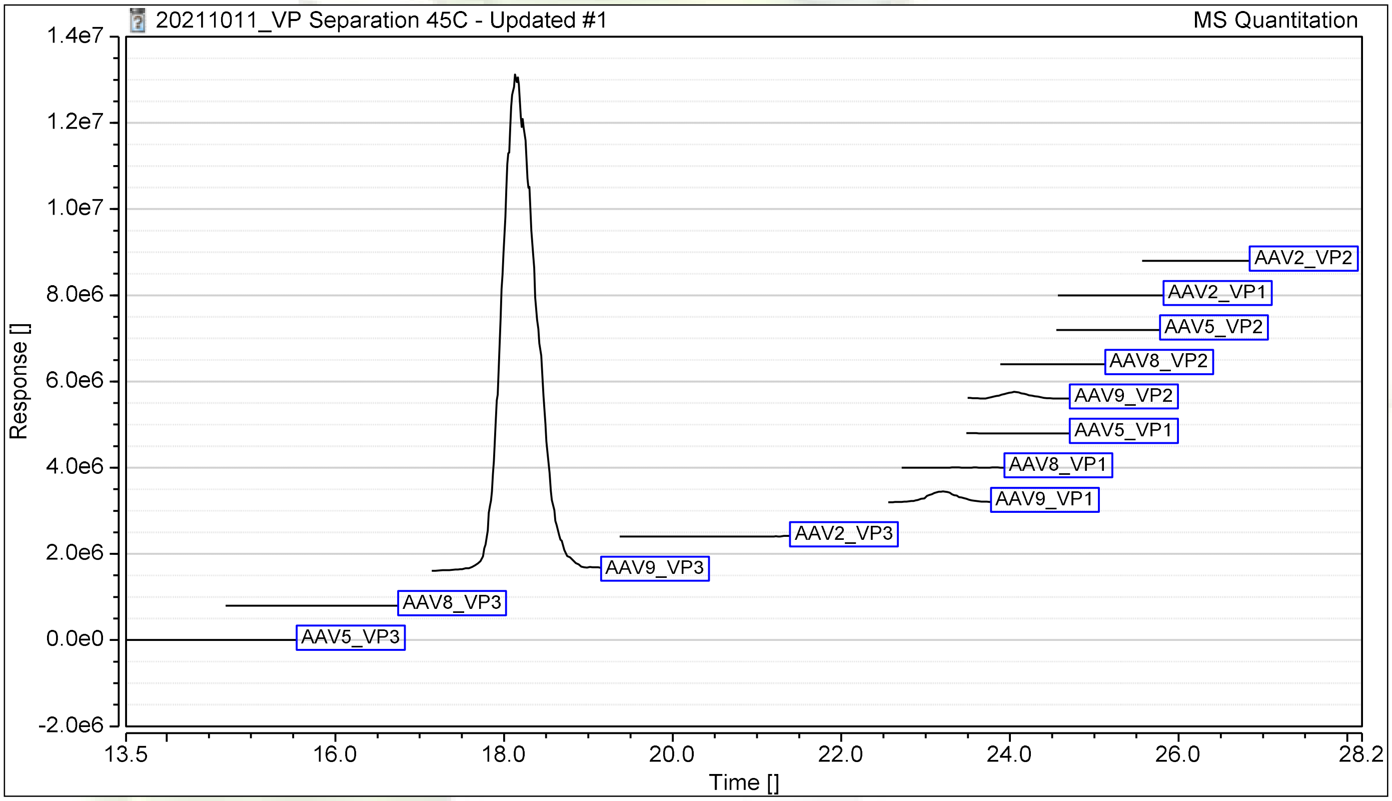

SST Results

No.	Name	Inj.Condition	Peak	Test Result	Injection
**Number of executed test cases:**	**n.a.**		**Total Result:**	**Passed**	

© 2009-2020 Thermo Fisher Scientific Inc. All rights reserved.

Protein Deconvolution Summary

Injection Details

Injection Name:	**20211011_AAV9 E_45C_65-59B_Mod1_1ug_2**	*Injection Volume:*	**2.80**
Vial Number:	**R:A5**	*Instrument Method:*	**AAV Cap_Prot_Sep_BEH Glycoprotein Amide H**
Injection Type:	**Unknown**	*Processing Method:*	**MS Quantitative_45C_65-59B**
Run Time (min):	**44.98**	*Injection Date/Time:*	**12/Oct/21 15:14**

Chromatogram

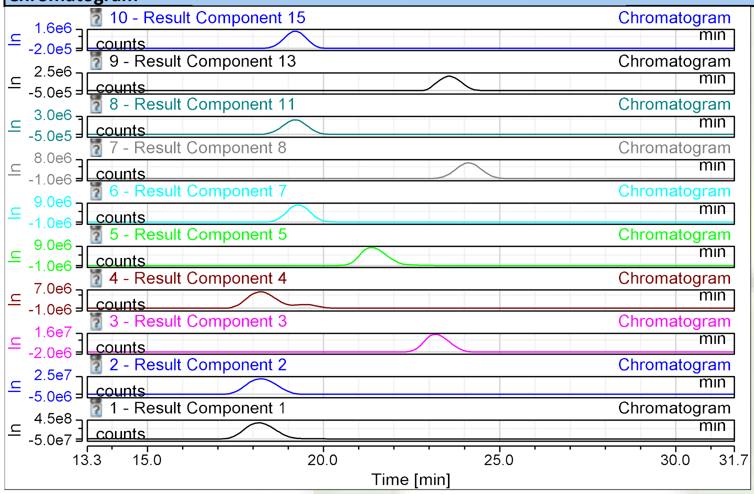

Source Spectrum

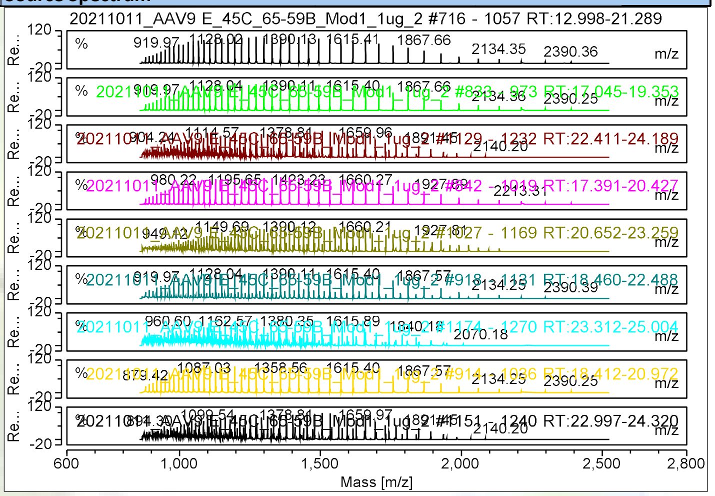

Deconvoluted Spectrum

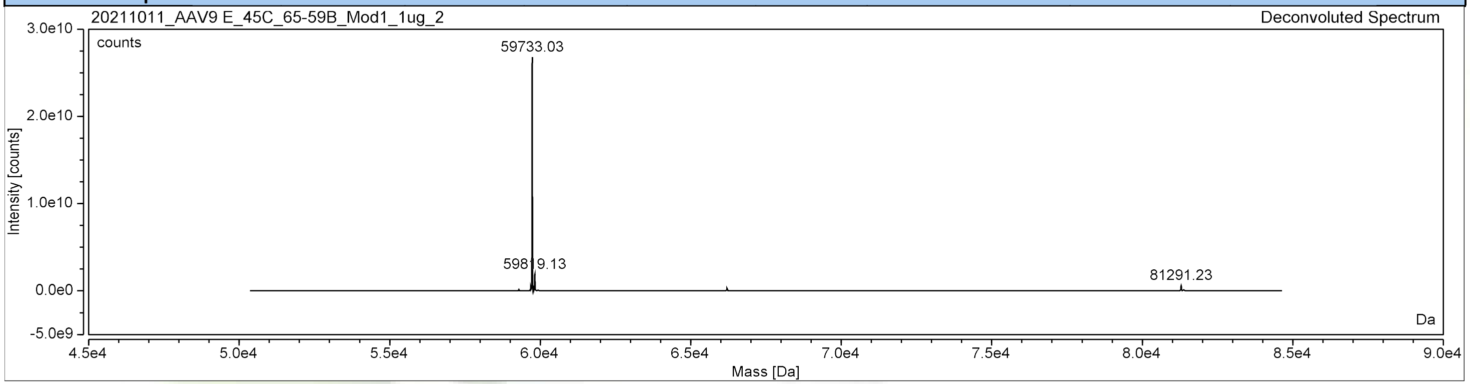

Deconvolution Results

Result Component	RT min	Mass Da	Std. Dev. Da	Std. Dev. ppm	Delta Mass Da	Intensity counts	Fractional Abundance counts	Rel. Abundance %	Number of Charge States	Score
Result Component 1	18.11	59733.033	0.40	6.68	0.000	27034264760	80.1	100.00	45	238.14
Result Component 2	18.22	59820.302	0.95	15.91	87.269	1527586438	4.5	5.65	40	170.79
Result Component 3	23.18	81291.415	0.91	11.13	21558.382	643910272	1.9	2.38	56	291.15
Result Component 4	19.20	59813.944	0.85	14.29	80.911	471832605	1.4	1.75	43	234.57
Result Component 5	18.27	59686.960	1.22	20.37	-46.073	390164875	1.2	1.44	32	176.14
Result Component 7	24.10	66210.268	0.85	12.85	6477.235	340089971	1.0	1.26	44	239.41
Result Component 8	21.47	59731.938	0.90	15.10	-1.095	300593658	0.9	1.11	41	217.18
Result Component 9	13.31	59733.070	0.64	10.75	0.037	298107673	0.9	1.10	42	257.50
Result Component 11	19.18	59933.627	1.10	18.41	200.594	200302659	0.6	0.74	40	199.85
Result Component 13	17.92	59687.388	1.55	25.99	-45.645	162773683	0.5	0.60	31	170.85
Result Component 15	21.21	59731.785	0.70	11.70	-1.249	129722977	0.4	0.48	41	225.58
Result Component 17	19.13	59766.513	1.08	18.11	33.479	91587642	0.3	0.34	38	197.64
Result Component 18	23.54	81372.238	1.92	23.54	21639.205	84227912	0.2	0.31	50	158.32
Result Component 19	18.85	59816.211	1.65	27.63	83.178	73795145	0.2	0.27	39	170.82
Result Component 20	19.45	59690.811	1.05	17.65	-42.222	72092544	0.2	0.27	37	195.30
Result Component 21	24.66	66290.558	1.28	19.26	6557.525	43412692	0.1	0.16	41	172.17

© 2009-2020 Thermo Fisher Scientific Inc. All rights reserved.
Chromeleon 7.3.0.60919

Component Summary

Injection Details

Injection Name:	**20211011_AAV9 E_45C_65-59B_Mod1_1ug**	*Injection Volume:*	**2.80**
Vial Number:	**R:A5**	*Instrument Method:*	**AAV Cap_Prot_Sep_BEH Glycoprotein Amide HILIC_1x150mm**
Injection Type:	**Unknown**	*Processing Method:*	**MS Quantitative_45C_65-59B**
Run Time (min):	**44.98**	*Injection Date/Time:*	**12/Oct/21 15:14**

Peak Name	Retention Time min	Area counts*min	Relative Area %	Quantitation Ions m/z	Mol.Mass	Mass Confirmation Pass/Fail
AAV9_VP3	18.181	4513899.308	88.57	1358.581,1390.150,1299.556,1328.415,1271.930,1457.907	59732.620	**PASS**
AAV9_VP1	23.221	112639.998	2.21	1378.820,1333.651,1355.868,1402.584,1534.808,1452.636	81290.340	**PASS**
AAV9_VP2	24.159	52114.190	1.02	1409.730,1162.589,1472.334,1274.287,1380.371,1352.231	66209.760	**PASS**
			**91.80**			

Component Name: AAV5_VP3

Quantitation ions:	1525.669,1565.784,1608.079,1451.304,1487.553,1416.767
Retention time (min):	14.53
Expected Mass:	59462.370
Area (count*min):	-
Intensity (counts):	-
Amount:	-

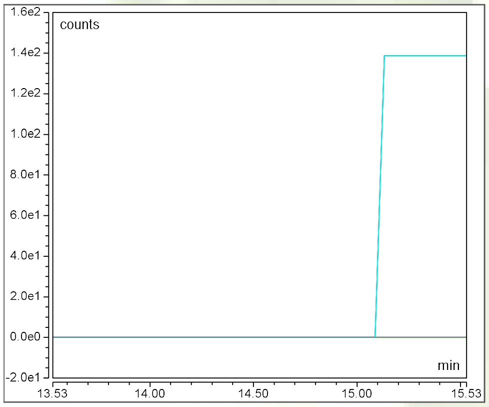

© 2009-2020 Thermo Fisher Scientific Inc. All rights reserved.
Chromeleon 7.3.0.60919

Component Name:	AAV8_VP3
Quantitation ions:	1574.800,1496.109,1534.442,1459.645,1273.445,1391.802
Retention time (min):	15.73
Expected Mass:	59804.680
Area (count*min):	-
Intensity (counts):	-
Amount:	-

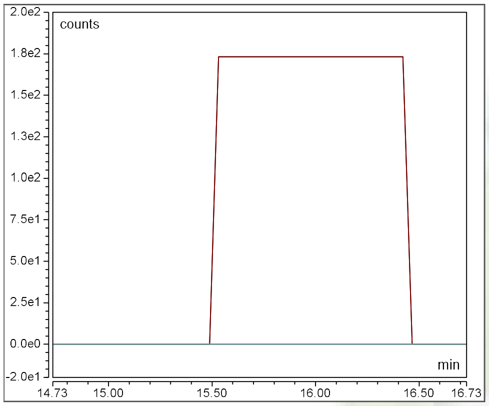

© 2009-2020 Thermo Fisher Scientific Inc. All rights reserved.
Chromeleon 7.3.0.60919

Component Name:	AAV9_VP3
Quantitation ions:	1358.581,1390.150,1299.556,1328.415,1271.930,1457.907
Retention time (min):	18.15
Expected Mass:	59732.620
Area (count*min):	4513899
Intensity (counts):	9305524
Amount:	-

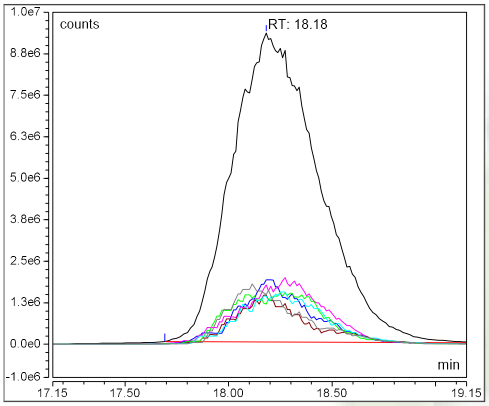

© 2009-2020 Thermo Fisher Scientific Inc. All rights reserved.
Chromeleon 7.3.0.60919

Component Name:	AAV2_VP3
Quantitation ions:	1428.955,1500.355,1463.789,1395.748,1364.055,1538.792
Retention time (min):	20.38
Expected Mass:	59973.780
Area (count*min):	-
Intensity (counts):	-
Amount:	-

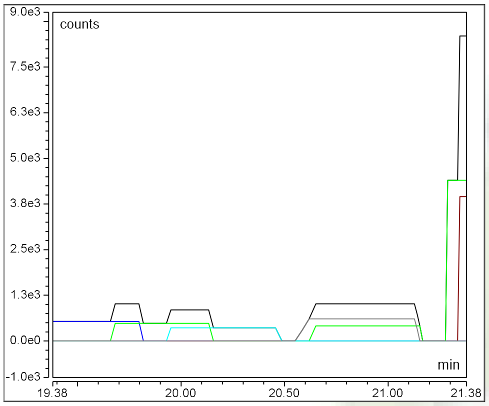

© 2009-2020 Thermo Fisher Scientific Inc. All rights reserved.
Chromeleon 7.3.0.60919

Component Name: AAV9_VP1	
Quantitation ions:	1378.820,1333.651,1355.868,1402.584,1534.808,1452.636
Retention time (min):	23.16
Expected Mass:	81290.340
Area (count*min):	112640
Intensity (counts):	308856
Amount:	-

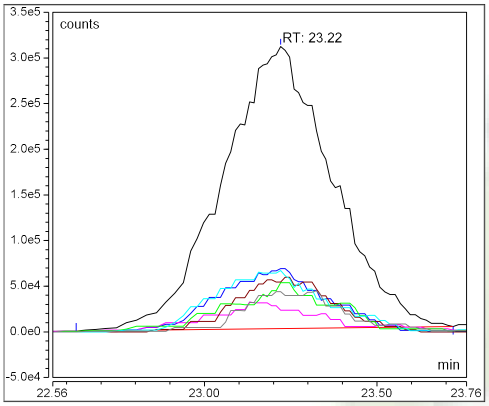

© 2009-2020 Thermo Fisher Scientific Inc. All rights reserved.
Chromeleon 7.3.0.60919

Component Name:	AAV8_VP1
Quantitation ions:	1409.080,1459.382,1385.209,1513.388,1184.605,1339.828
Retention time (min):	23.33
Expected Mass:	81666.710
Area (count*min):	-
Intensity (counts):	-
Amount:	-

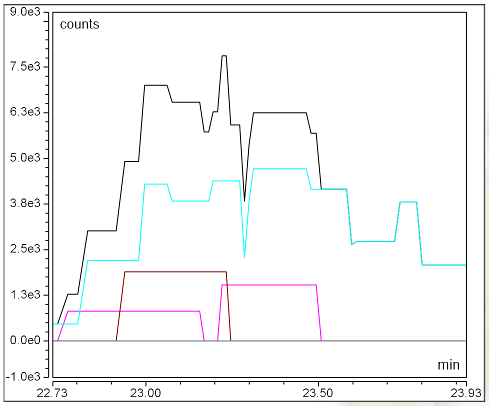

© 2009-2020 Thermo Fisher Scientific Inc. All rights reserved.
Chromeleon 7.3.0.60919

Component Name:	AAV5_VP1
Quantitation ions:	1362.628,1410.415,1435.577,1317.996,1339.949,1488.697
Retention time (min):	24.09
Expected Mass:	80335.080
Area (count*min):	-
Intensity (counts):	-
Amount:	-

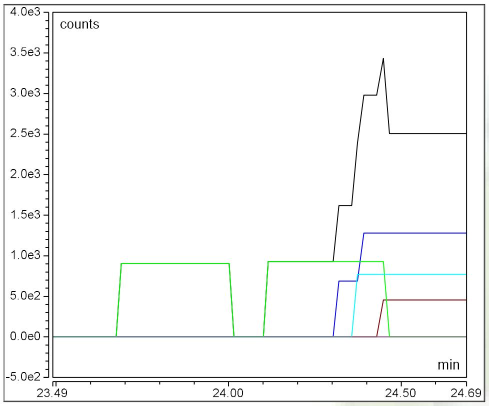

© 2009-2020 Thermo Fisher Scientific Inc. All rights reserved.
Chromeleon 7.3.0.60919

Component Name:	AAV9_VP2
Quantitation ions:	1409.730,1162.589,1472.334,1274.287,1380.371,1352.231
Retention time (min):	24.10
Expected Mass:	66209.760
Area (count*min):	52114
Intensity (counts):	131634
Amount:	-

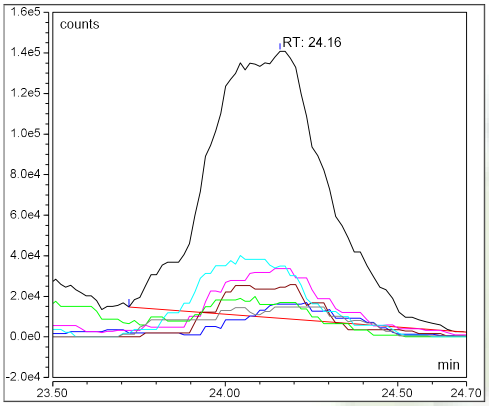

© 2009-2020 Thermo Fisher Scientific Inc. All rights reserved.
Chromeleon 7.3.0.60919

Component Name:	AAV8_VP2
Quantitation ions:	1386.809,1358.524,1331.393,1305.308,1416.294,1623.397
Retention time (min):	24.50
Expected Mass:	66518.100
Area (count*min):	-
Intensity (counts):	-
Amount:	-

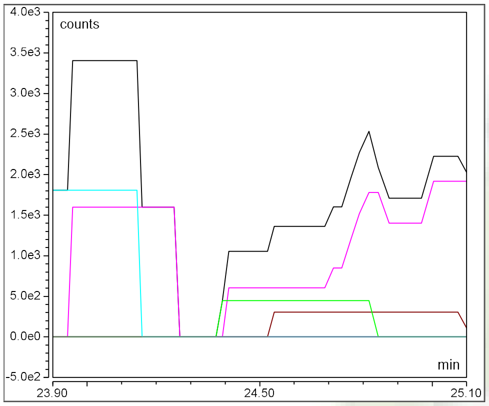

© 2009-2020 Thermo Fisher Scientific Inc. All rights reserved.
Chromeleon 7.3.0.60919

Component Name: AAV5_VP2

Quantitation ions:	1389.997,1519.197,1484.692,1420.196,1555.348,1451.727
Retention time (min):	25.16
Expected Mass:	65282.690
Area (count*min):	-
Intensity (counts):	-
Amount:	-

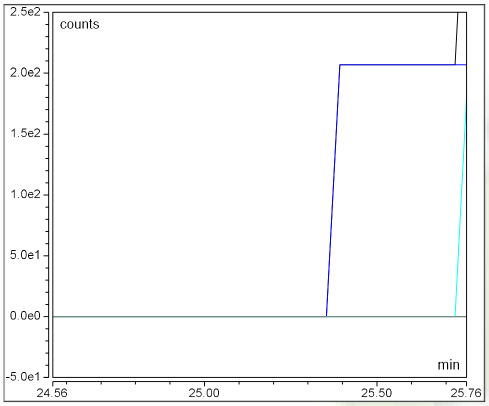

© 2009-2020 Thermo Fisher Scientific Inc. All rights reserved.
Chromeleon 7.3.0.60919

Component Name:	AAV2_VP1
Quantitation ions:	1388.434,1545.445,1489.336,1342.917,1365.283,1412.362
Retention time (min):	25.18
Expected Mass:	81854.900
Area (count*min):	-
Intensity (counts):	-
Amount:	-

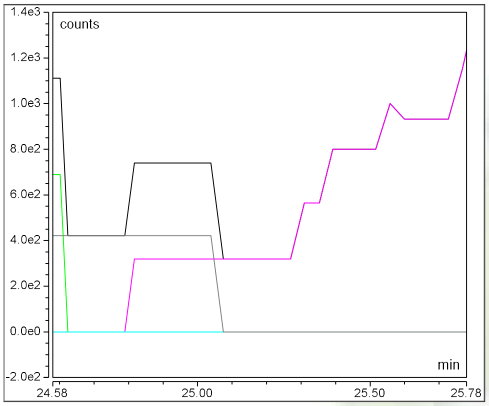

© 2009-2020 Thermo Fisher Scientific Inc. All rights reserved.
Chromeleon 7.3.0.60919

Component Name:	AAV2_VP2
Quantitation ions:	1415.636,1446.415,1255.530,1478.506,1279.609,1304.686
Retention time (min):	26.20
Expected Mass:	66487.900
Area (count*min):	-
Intensity (counts):	-
Amount:	-

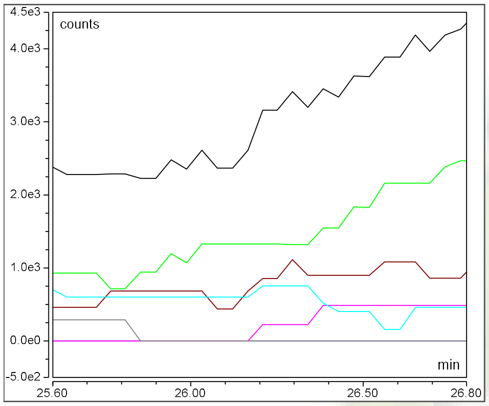

© 2009-2020 Thermo Fisher Scientific Inc. All rights reserved.
Chromeleon 7.3.0.60919

Chromatogram and SST Results

Injection Details

Injection Name:	**20211011_AAV9 E_45C_65-59B_Mod1_1ug_2**	*Run Time (min):*	**44.98**
Vial Number:	**R:A5**	*Injection Volume:*	**2.80**
Injection Type:	**Unknown**		
Calibration Level:			
Instrument Method:	**AAV Cap_Prot_Sep_BEH Glycoprotein Amide HILIC_1x150mm_FLR + MS_CT45C_65-59_S**		
Processing Method:	**MS Quantitative_45C_65-59B**	*Dilution Factor:*	**1.0000**
Injection Date/Time:	**12/Oct/21 15:14**	*Sample Weight:*	**1.0000**

Chromatogram

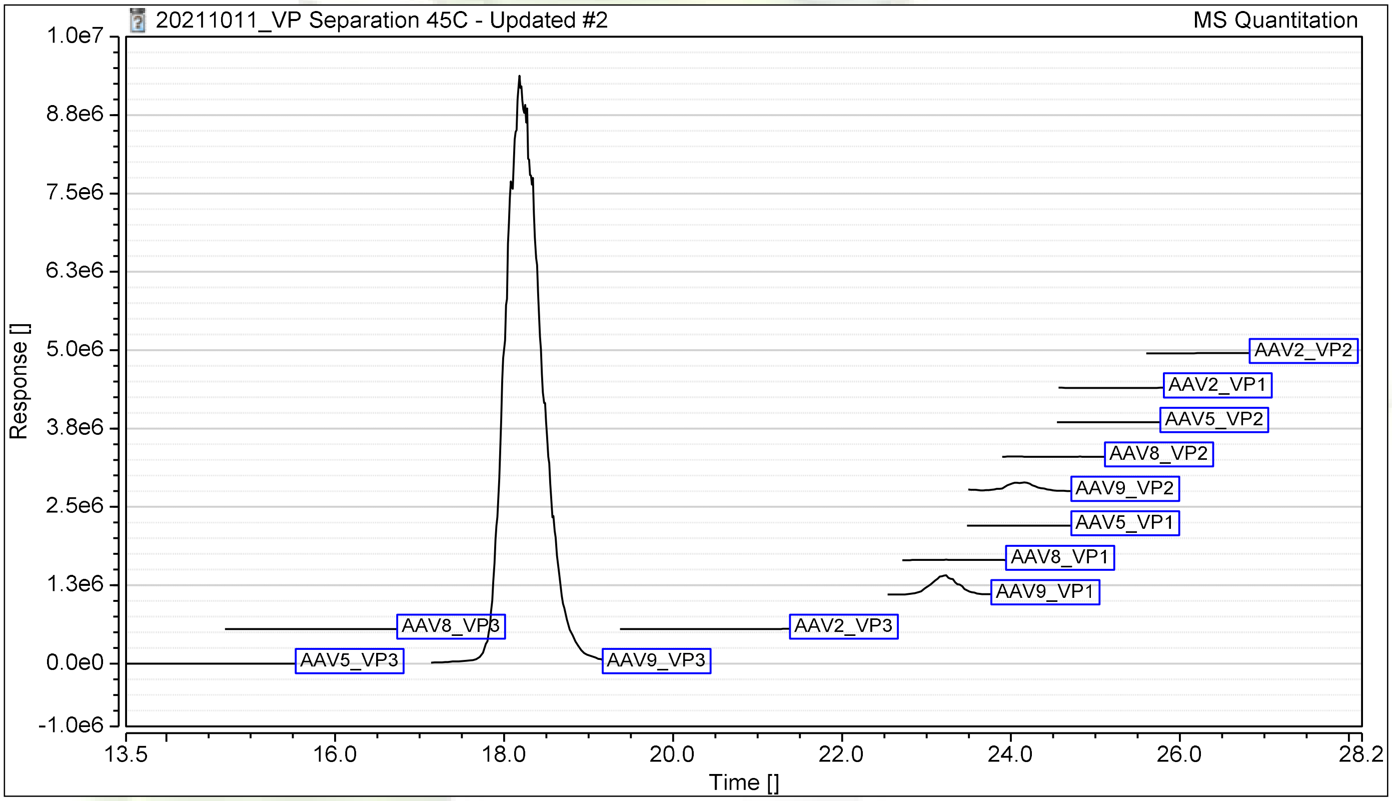

SST Results

No.	Name	Inj.Condition	Peak	Test Result	Injection
**Number of executed test cases:**	**n.a.**		**Total Result:**	**Passed**	

© 2009-2020 Thermo Fisher Scientific Inc. All rights reserved.

Protein Deconvolution Summary

Injection Details

Injection Name:	**20211011_AAV9 E_45C_65-59B_Mod1_1ug_3**	*Injection Volume:*	**2.80**
Vial Number:	**R:A5**	*Instrument Method:*	**AAV Cap_Prot_Sep_BEH Glycoprotein Amide H**
Injection Type:	**Unknown**	*Processing Method:*	**MS Quantitative_45C_65-59B**
Run Time (min):	**45.00**	*Injection Date/Time:*	**12/Oct/21 16:00**

Chromatogram

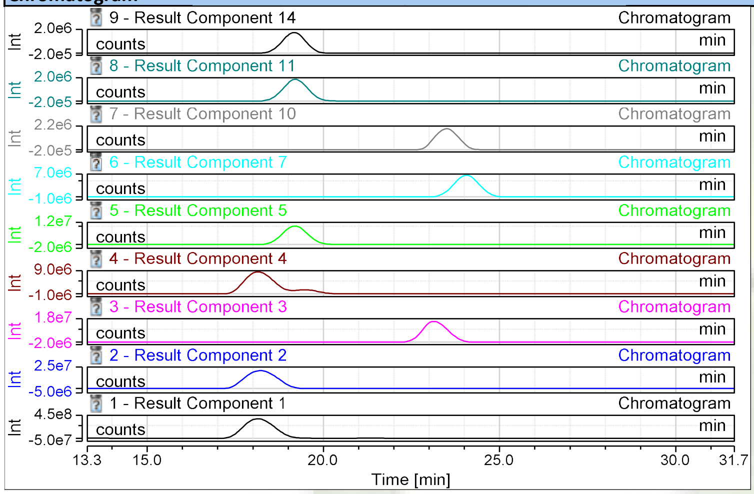

Source Spectrum

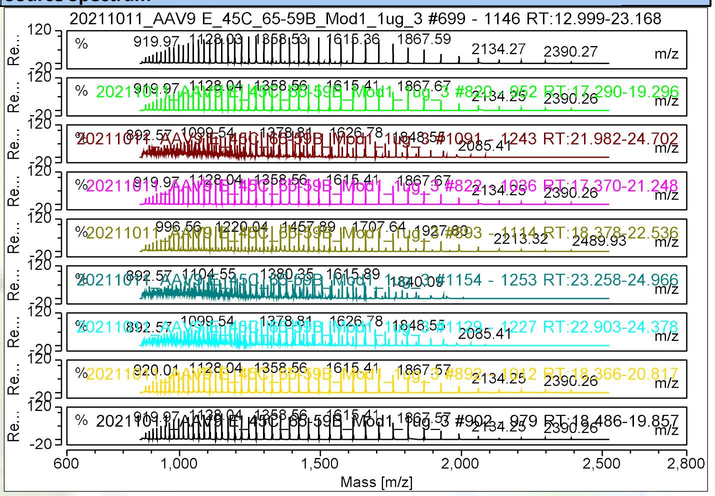

Deconvoluted Spectrum

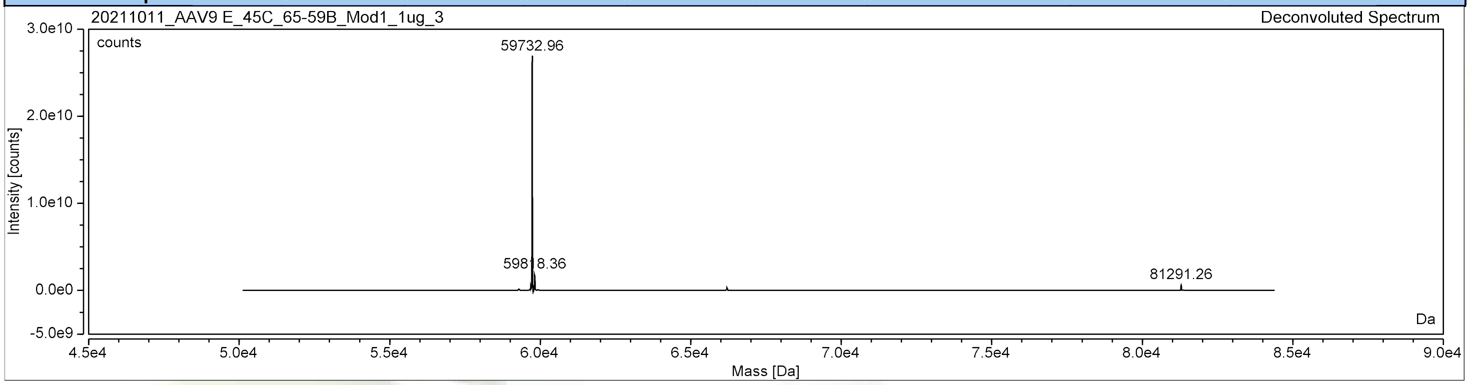

Deconvolution Results

Result Component	RT min	Mass Da	Std. Dev. Da	Std. Dev. ppm	Delta Mass Da	Intensity counts	Fractional Abundance counts	Rel. Abundance %	Number of Charge States	Score
Result Component 1	18.11	59733.033	0.40	6.68	0.000	27034264760	80.1	100.00	45	238.14
Result Component 2	18.22	59820.302	0.95	15.91	87.269	1527586438	4.5	5.65	40	170.79
Result Component 3	23.18	81291.415	0.91	11.13	21558.382	643910272	1.9	2.38	56	291.15
Result Component 4	19.20	59813.944	0.85	14.29	80.911	471832605	1.4	1.75	43	234.57
Result Component 5	18.27	59686.960	1.22	20.37	-46.073	390164875	1.2	1.44	32	176.14
Result Component 7	24.10	66210.268	0.85	12.85	6477.235	340089971	1.0	1.26	44	239.41
Result Component 8	21.47	59731.938	0.90	15.10	-1.095	300593658	0.9	1.11	41	217.18
Result Component 9	13.31	59733.070	0.64	10.75	0.037	298107673	0.9	1.10	42	257.50
Result Component 11	19.18	59933.627	1.10	18.41	200.594	200302659	0.6	0.74	40	199.85
Result Component 13	17.92	59687.388	1.55	25.99	-45.645	162773683	0.5	0.60	31	170.85
Result Component 15	21.21	59731.785	0.70	11.70	-1.249	129722977	0.4	0.48	41	225.58
Result Component 17	19.13	59766.513	1.08	18.11	33.479	91587642	0.3	0.34	38	197.64
Result Component 18	23.54	81372.238	1.92	23.54	21639.205	84227912	0.2	0.31	50	158.32
Result Component 19	18.85	59816.211	1.65	27.63	83.178	73795145	0.2	0.27	39	170.82
Result Component 20	19.45	59690.811	1.05	17.65	-42.222	72092544	0.2	0.27	37	195.30
Result Component 21	24.66	66290.558	1.28	19.26	6557.525	43412692	0.1	0.16	41	172.17

© 2009-2020 Thermo Fisher Scientific Inc. All rights reserved.
Chromeleon 7.3.0.60919

Component Summary

Injection Details

Injection Name:	**20211011_AAV9 E_45C_65-59B_Mod1_1ug**	*Injection Volume:*	**2.80**
Vial Number:	**R:A5**	*Instrument Method:*	**AAV Cap_Prot_Sep_BEH Glycoprotein Amide HILIC_1x150mm**
Injection Type:	**Unknown**	*Processing Method:*	**MS Quantitative_45C_65-59B**
Run Time (min):	**45.00**	*Injection Date/Time:*	**12/Oct/21 16:00**

Peak Name	Retention Time min	Area counts*min	Relative Area %	Quantitation Ions m/z	Mol.Mass	Mass Confirmation Pass/Fail
AAV9_VP3	18.149	4808333.069	87.11	1358.581,1390.150,1299.556,1328.415,1271.930,1457.907	59732.620	**PASS**
AAV9_VP1	23.155	122883.800	2.23	1378.820,1333.651,1355.868,1402.584,1534.808,1452.636	81290.340	**PASS**
AAV9_VP2	24.010	48701.492	0.88	1409.730,1162.589,1472.334,1274.287,1380.371,1352.231	66209.760	**PASS**
			**90.21**			

Component Name: AAV5_VP3

Quantitation ions:	1525.669,1565.784,1608.079,1451.304,1487.553,1416.767
Retention time (min):	14.53
Expected Mass:	59462.370
Area (count*min):	-
Intensity (counts):	-
Amount:	-

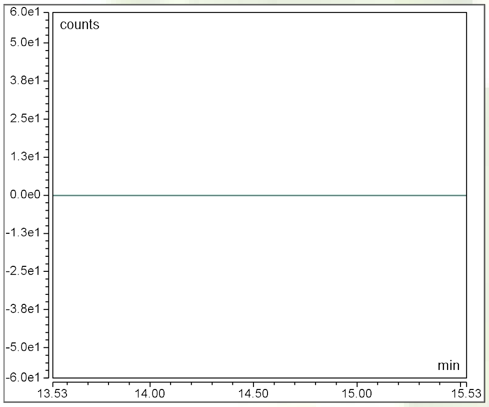

© 2009-2020 Thermo Fisher Scientific Inc. All rights reserved.
Chromeleon 7.3.0.60919

Component Name: AAV8_VP3

Quantitation ions:	1574.800,1496.109,1534.442,1459.645,1273.445,1391.802
Retention time (min):	15.73
Expected Mass:	59804.680
Area (count*min):	-
Intensity (counts):	-
Amount:	-

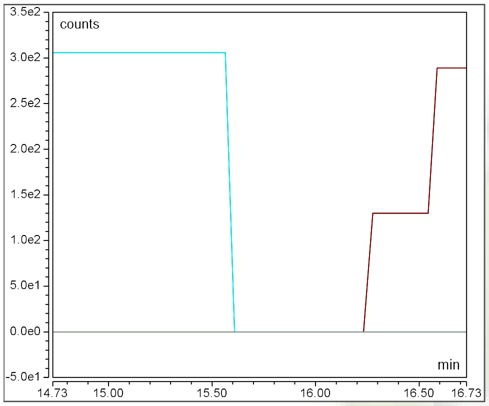

© 2009-2020 Thermo Fisher Scientific Inc. All rights reserved.
Chromeleon 7.3.0.60919

Component Name:	AAV9_VP3
Quantitation ions:	1358.581,1390.150,1299.556,1328.415,1271.930,1457.907
Retention time (min):	18.15
Expected Mass:	59732.620
Area (count*min):	4808333
Intensity (counts):	9879358
Amount:	-

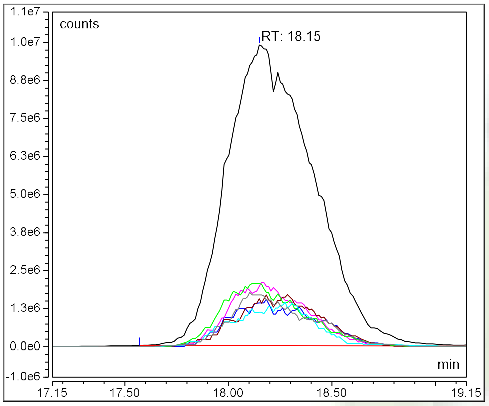

© 2009-2020 Thermo Fisher Scientific Inc. All rights reserved.
Chromeleon 7.3.0.60919

Component Name:	AAV2_VP3
Quantitation ions:	1428.955,1500.355,1463.789,1395.748,1364.055,1538.792
Retention time (min):	20.38
Expected Mass:	59973.780
Area (count*min):	-
Intensity (counts):	-
Amount:	-

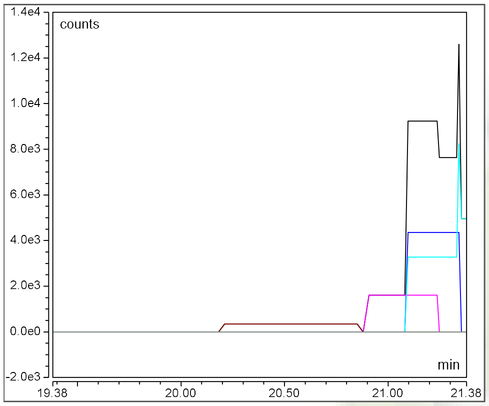

© 2009-2020 Thermo Fisher Scientific Inc. All rights reserved.
Chromeleon 7.3.0.60919

Component Name:	AAV9_VP1
Quantitation ions:	1378.820,1333.651,1355.868,1402.584,1534.808,1452.636
Retention time (min):	23.16
Expected Mass:	81290.340
Area (count*min):	122884
Intensity (counts):	330166
Amount:	-

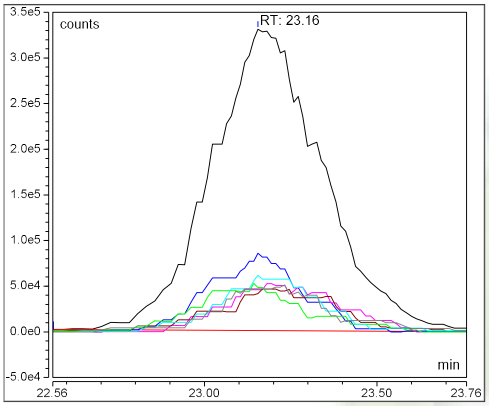

© 2009-2020 Thermo Fisher Scientific Inc. All rights reserved.
Chromeleon 7.3.0.60919

Component Name: AAV8_VP1

Quantitation ions:	1409.080,1459.382,1385.209,1513.388,1184.605,1339.828
Retention time (min):	23.33
Expected Mass:	81666.710
Area (count*min):	-
Intensity (counts):	-
Amount:	-

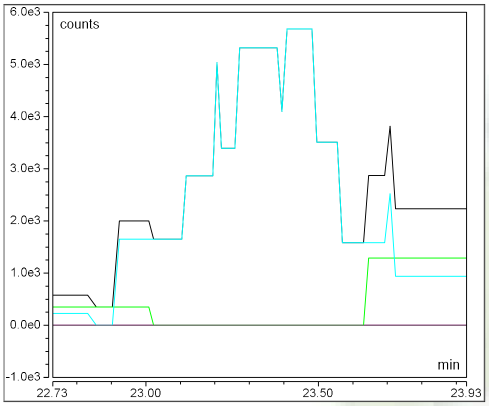

© 2009-2020 Thermo Fisher Scientific Inc. All rights reserved.
Chromeleon 7.3.0.60919

Component Name:	AAV5_VP1
Quantitation ions:	1362.628,1410.415,1435.577,1317.996,1339.949,1488.697
Retention time (min):	24.09
Expected Mass:	80335.080
Area (count*min):	-
Intensity (counts):	-
Amount:	-

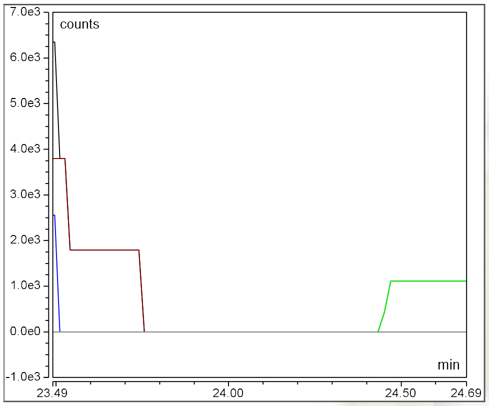

© 2009-2020 Thermo Fisher Scientific Inc. All rights reserved.
Chromeleon 7.3.0.60919

Component Name:	AAV9_VP2
Quantitation ions:	1409.730,1162.589,1472.334,1274.287,1380.371,1352.231
Retention time (min):	24.10
Expected Mass:	66209.760
Area (count*min):	48701
Intensity (counts):	118626
Amount:	-

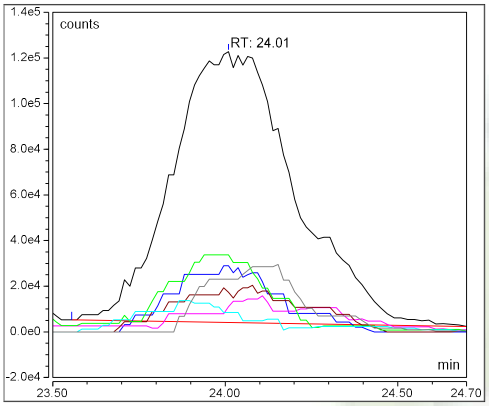

© 2009-2020 Thermo Fisher Scientific Inc. All rights reserved.
Chromeleon 7.3.0.60919

Component Name: AAV8_VP2

Quantitation ions:	1386.809,1358.524,1331.393,1305.308,1416.294,1623.397
Retention time (min):	24.50
Expected Mass:	66518.100
Area (count*min):	-
Intensity (counts):	-
Amount:	-

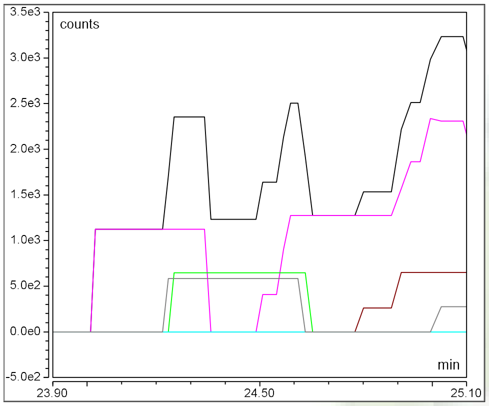

© 2009-2020 Thermo Fisher Scientific Inc. All rights reserved.
Chromeleon 7.3.0.60919

Component Name:	AAV5_VP2
Quantitation ions:	1389.997,1519.197,1484.692,1420.196,1555.348,1451.727
Retention time (min):	25.16
Expected Mass:	65282.690
Area (count*min):	-
Intensity (counts):	-
Amount:	-

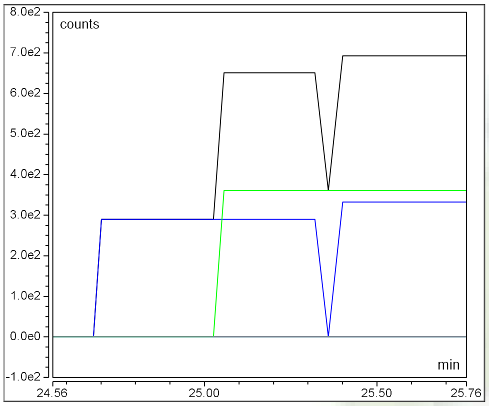

© 2009-2020 Thermo Fisher Scientific Inc. All rights reserved.
Chromeleon 7.3.0.60919

Component Name:	AAV2_VP1
Quantitation ions:	1388.434,1545.445,1489.336,1342.917,1365.283,1412.362
Retention time (min):	25.18
Expected Mass:	81854.900
Area (count*min):	-
Intensity (counts):	-
Amount:	-

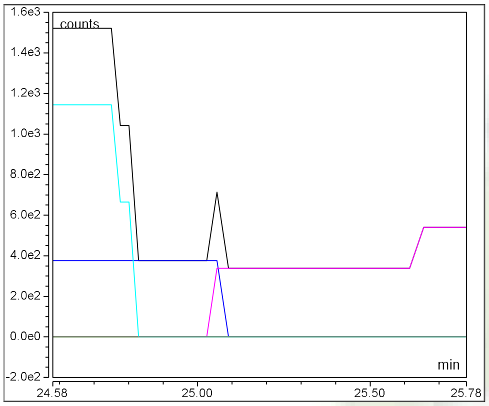

© 2009-2020 Thermo Fisher Scientific Inc. All rights reserved.
Chromeleon 7.3.0.60919

Component Name:	AAV2_VP2
Quantitation ions:	1415.636,1446.415,1255.530,1478.506,1279.609,1304.686
Retention time (min):	26.20
Expected Mass:	66487.900
Area (count*min):	-
Intensity (counts):	-
Amount:	-

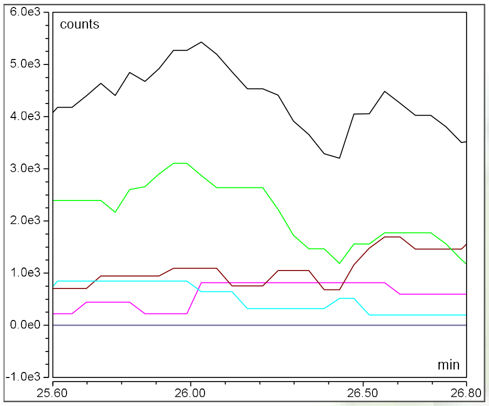

© 2009-2020 Thermo Fisher Scientific Inc. All rights reserved.
Chromeleon 7.3.0.60919

Chromatogram and SST Results

Injection Details

Injection Name:	**20211011_AAV9 E_45C_65-59B_Mod1_1ug_3**	*Run Time (min):*	**45.00**
Vial Number:	**R:A5**	*Injection Volume:*	**2.80**
Injection Type:	**Unknown**		
Calibration Level:			
Instrument Method:	**AAV Cap_Prot_Sep_BEH Glycoprotein Amide HILIC_1x150mm_FLR + MS_CT45C_65-59_S**		
Processing Method:	**MS Quantitative_45C_65-59B**	*Dilution Factor:*	**1.0000**
Injection Date/Time:	**12/Oct/21 16:00**	*Sample Weight:*	**1.0000**

Chromatogram

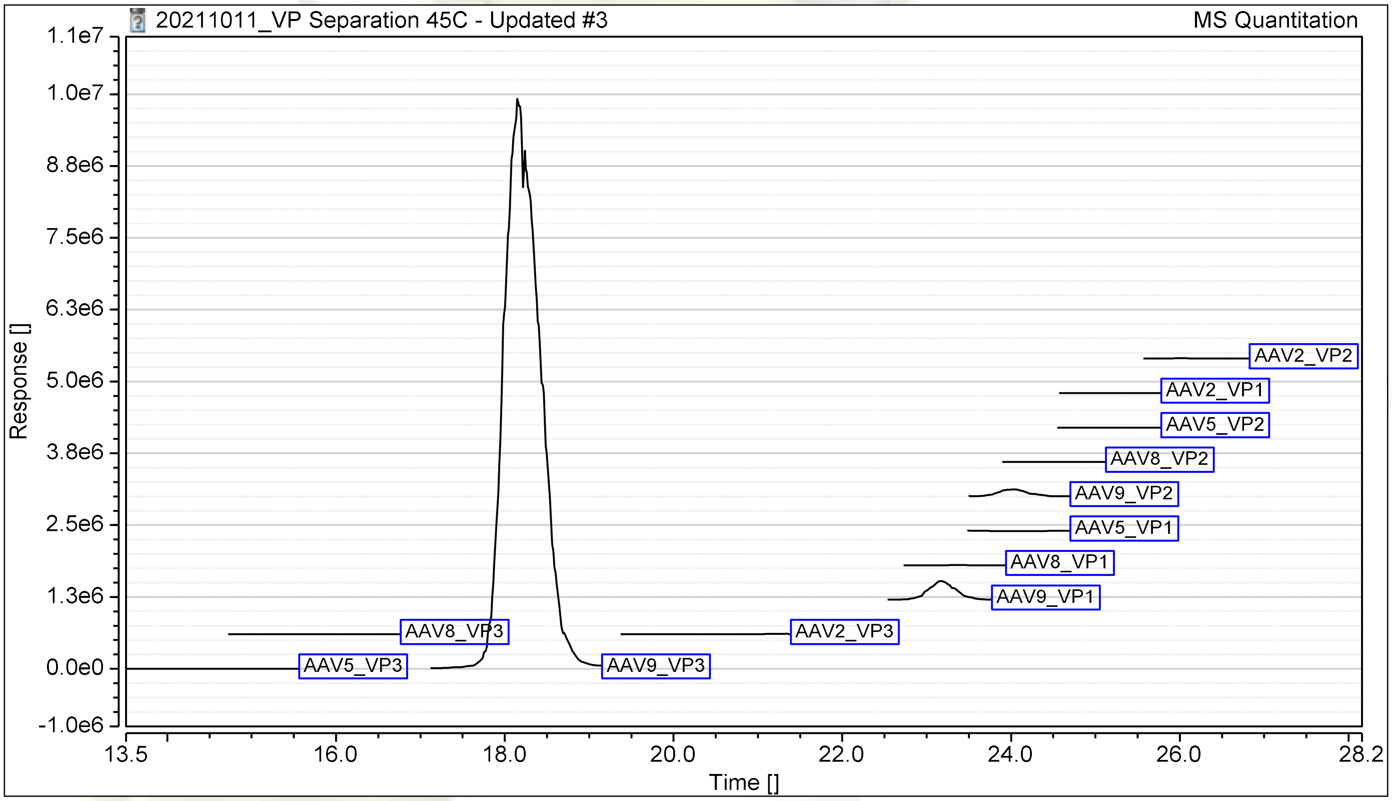

SST Results

No.	Name	Inj.Condition	Peak	Test Result	Injection
**Number of executed test cases:**	**n.a.**		**Total Result:**	**Passed**	

© 2009-2020 Thermo Fisher Scientific Inc. All rights reserved.
Chromeleon 7.3.0.60919

Protein Deconvolution Summary

Injection Details

Injection Name:	**20211011_AAV2 E_45C_65-59B_Mod1_1ug_1**	*Injection Volume:*	**2.86**
Vial Number:	**R:A1**	*Instrument Method:*	**AAV Cap_Prot_Sep_BEH Glycoprotein Amide H**
Injection Type:	**Unknown**	*Processing Method:*	**MS Quantitative_45C_65-59B**
Run Time (min):	**45.00**	*Injection Date/Time:*	**12/Oct/21 21:57**

Chromatogram

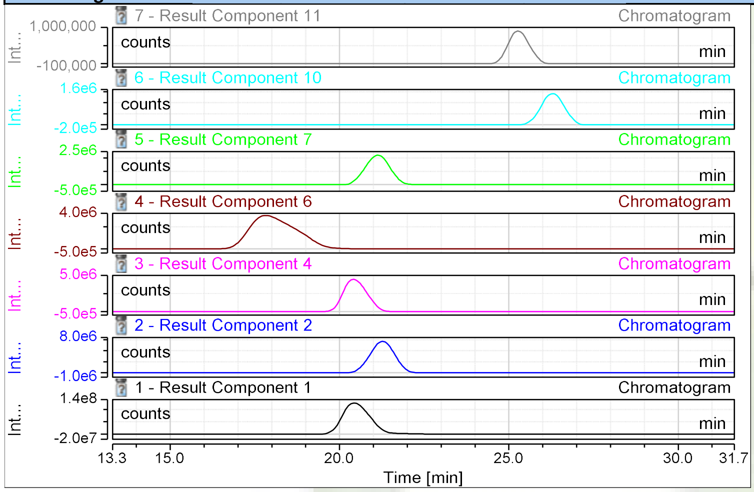

Source Spectrum

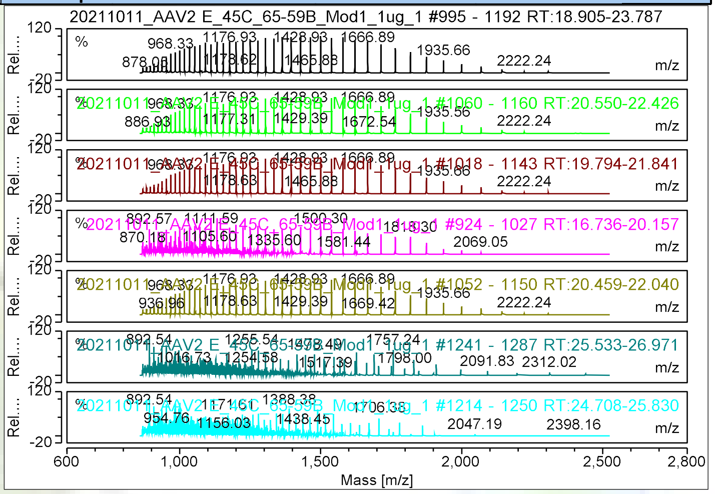

Deconvoluted Spectrum

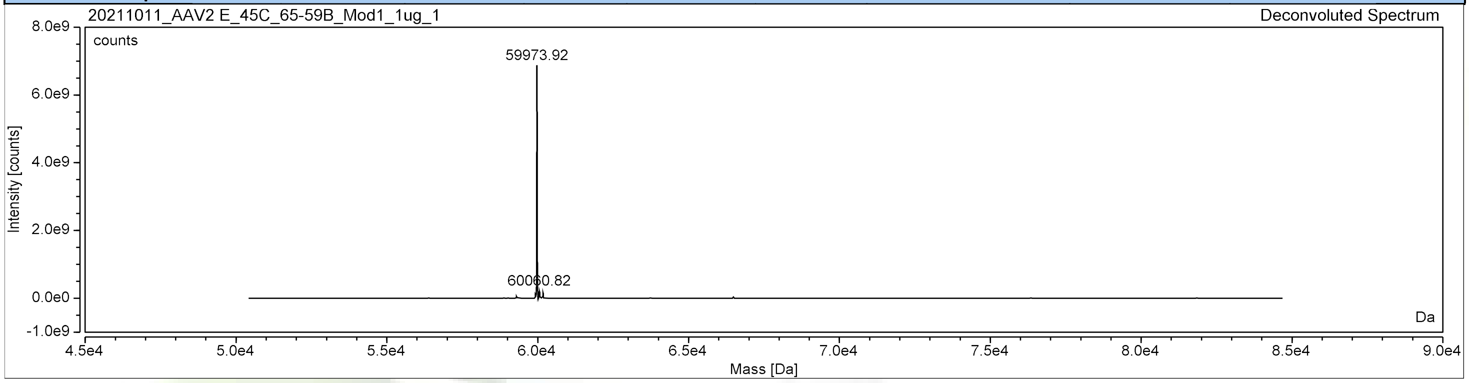

Deconvolution Results

Result Component	RT min	Mass Da	Std. Dev. Da	Std. Dev. ppm	Delta Mass Da	Intensity counts	Fractional Abundance counts	Rel. Abundance %	Number of Charge States	Score
Result Component 1	18.11	59733.033	0.40	6.68	0.000	27034264760	80.1	100.00	45	238.14
Result Component 2	18.22	59820.302	0.95	15.91	87.269	1527586438	4.5	5.65	40	170.79
Result Component 3	23.18	81291.415	0.91	11.13	21558.382	643910272	1.9	2.38	56	291.15
Result Component 4	19.20	59813.944	0.85	14.29	80.911	471832605	1.4	1.75	43	234.57
Result Component 5	18.27	59686.960	1.22	20.37	-46.073	390164875	1.2	1.44	32	176.14
Result Component 7	24.10	66210.268	0.85	12.85	6477.235	340089971	1.0	1.26	44	239.41
Result Component 8	21.47	59731.938	0.90	15.10	-1.095	300593658	0.9	1.11	41	217.18
Result Component 9	13.31	59733.070	0.64	10.75	0.037	298107673	0.9	1.10	42	257.50
Result Component 11	19.18	59933.627	1.10	18.41	200.594	200302659	0.6	0.74	40	199.85
Result Component 13	17.92	59687.388	1.55	25.99	-45.645	162773683	0.5	0.60	31	170.85
Result Component 15	21.21	59731.785	0.70	11.70	-1.249	129722977	0.4	0.48	41	225.58
Result Component 17	19.13	59766.513	1.08	18.11	33.479	91587642	0.3	0.34	38	197.64
Result Component 18	23.54	81372.238	1.92	23.54	21639.205	84227912	0.2	0.31	50	158.32
Result Component 19	18.85	59816.211	1.65	27.63	83.178	73795145	0.2	0.27	39	170.82
Result Component 20	19.45	59690.811	1.05	17.65	-42.222	72092544	0.2	0.27	37	195.30
Result Component 21	24.66	66290.558	1.28	19.26	6557.525	43412692	0.1	0.16	41	172.17

© 2009-2020 Thermo Fisher Scientific Inc. All rights reserved.
Chromeleon 7.3.0.60919

Component Summary

Injection Details

Injection Name:	**20211011_AAV2 E_45C_65-59B_Mod1_1ug**	*Injection Volume:*	**2.86**
Vial Number:	**R:A1**	*Instrument Method:*	**AAV Cap_Prot_Sep_BEH Glycoprotein Amide HILIC_1x150mm**
Injection Type:	**Unknown**	*Processing Method:*	**MS Quantitative_45C_65-59B**
Run Time (min):	**45.00**	*Injection Date/Time:*	**12/Oct/21 21:57**

Peak Name	Retention Time min	Area counts*min	Relative Area %	Quantitation Ions m/z	Mol.Mass	Mass Confirmation Pass/Fail
AAV2_VP3	20.493	1423308.517	86.33	1428.955,1500.355,1463.789,1395.748,1364.055,1538.792	59973.780	**PASS**
AAV2_VP1	25.313	13464.491	0.82	1388.434,1545.445,1489.336,1342.917,1365.283,1412.362	81854.900	**PASS**
AAV2_VP2	26.236	15926.651	0.97	1415.636,1446.415,1255.530,1478.506,1279.609,1304.686	66487.900	**PASS**
			**88.11**			

Component Name: AAV5_VP3

Quantitation ions:	1525.669,1565.784,1608.079,1451.304,1487.553,1416.767
Retention time (min):	14.53
Expected Mass:	59462.370
Area (count*min):	-
Intensity (counts):	-
Amount:	-

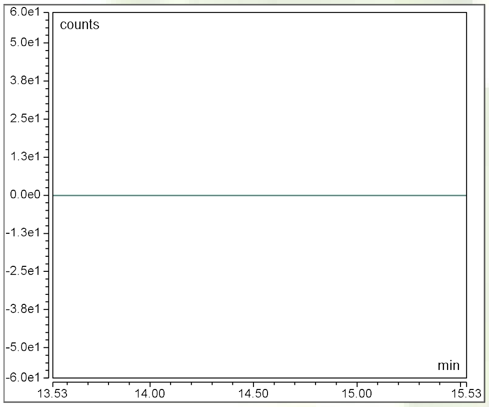

© 2009-2020 Thermo Fisher Scientific Inc. All rights reserved.
Chromeleon 7.3.0.60919

Component Name:	AAV8_VP3
Quantitation ions:	1574.800,1496.109,1534.442,1459.645,1273.445,1391.802
Retention time (min):	15.73
Expected Mass:	59804.680
Area (count*min):	-
Intensity (counts):	-
Amount:	-

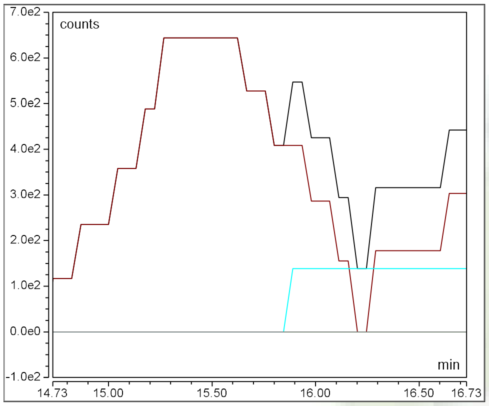

© 2009-2020 Thermo Fisher Scientific Inc. All rights reserved.
Chromeleon 7.3.0.60919

Component Name:	AAV9_VP3
Quantitation ions:	1358.581,1390.150,1299.556,1328.415,1271.930,1457.907
Retention time (min):	18.15
Expected Mass:	59732.620
Area (count*min):	-
Intensity (counts):	-
Amount:	-

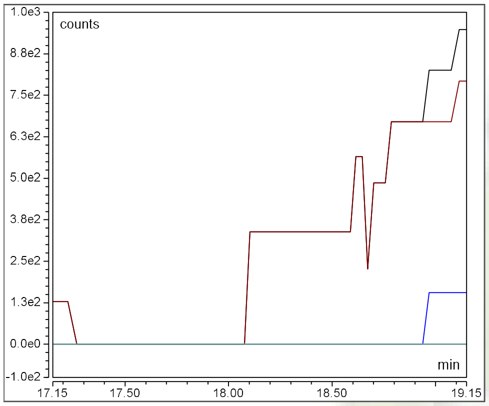

© 2009-2020 Thermo Fisher Scientific Inc. All rights reserved.
Chromeleon 7.3.0.60919

Component Name:	AAV2_VP3
Quantitation ions:	1428.955,1500.355,1463.789,1395.748,1364.055,1538.792
Retention time (min):	20.38
Expected Mass:	59973.780
Area (count*min):	1423309
Intensity (counts):	3398310
Amount:	-

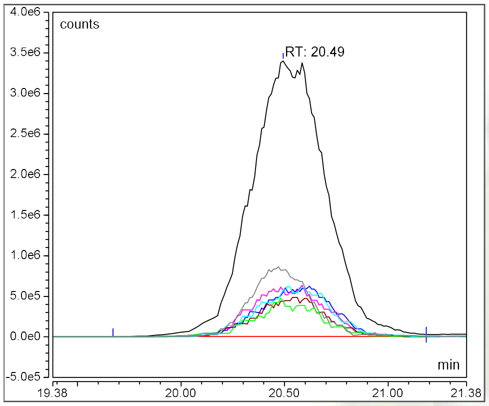

© 2009-2020 Thermo Fisher Scientific Inc. All rights reserved.
Chromeleon 7.3.0.60919

Component Name:	AAV9_VP1
Quantitation ions:	1378.820,1333.651,1355.868,1402.584,1534.808,1452.636
Retention time (min):	23.16
Expected Mass:	81290.340
Area (count*min):	-
Intensity (counts):	-
Amount:	-

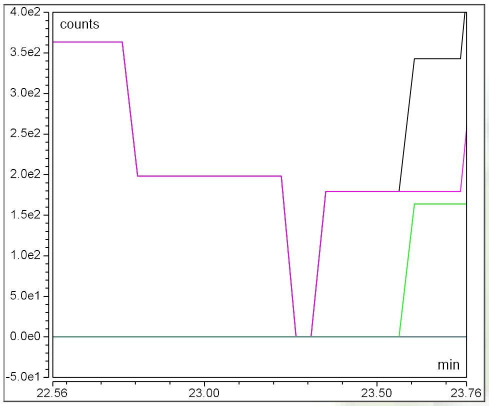

© 2009-2020 Thermo Fisher Scientific Inc. All rights reserved.
Chromeleon 7.3.0.60919

Component Name:	AAV8_VP1
Quantitation ions:	1409.080,1459.382,1385.209,1513.388,1184.605,1339.828
Retention time (min):	23.33
Expected Mass:	81666.710
Area (count*min):	-
Intensity (counts):	-
Amount:	-

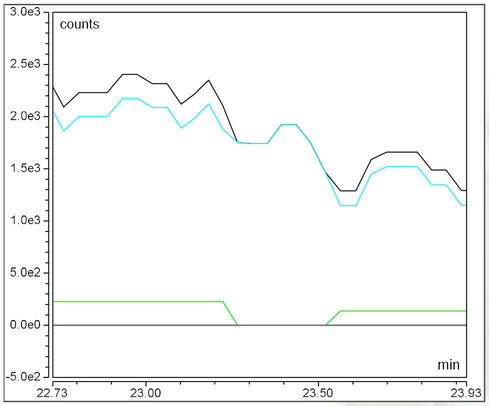

© 2009-2020 Thermo Fisher Scientific Inc. All rights reserved.
Chromeleon 7.3.0.60919

Component Name: AAV5_VP1

Quantitation ions:	1362.628,1410.415,1435.577,1317.996,1339.949,1488.697
Retention time (min):	24.09
Expected Mass:	80335.080
Area (count*min):	-
Intensity (counts):	-
Amount:	-

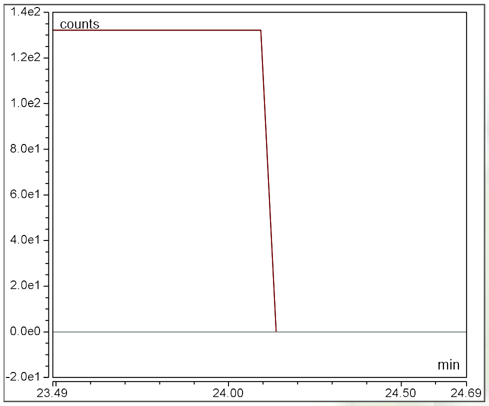

© 2009-2020 Thermo Fisher Scientific Inc. All rights reserved.
Chromeleon 7.3.0.60919

Component Name:	AAV9_VP2
Quantitation ions:	1409.730,1162.589,1472.334,1274.287,1380.371,1352.231
Retention time (min):	24.10
Expected Mass:	66209.760
Area (count*min):	-
Intensity (counts):	-
Amount:	-

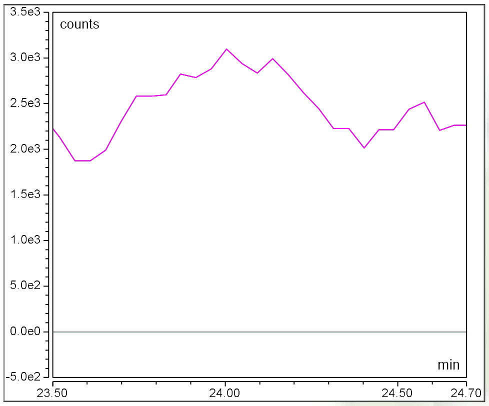

© 2009-2020 Thermo Fisher Scientific Inc. All rights reserved.
Chromeleon 7.3.0.60919

Component Name:	AAV8_VP2
Quantitation ions:	1386.809,1358.524,1331.393,1305.308,1416.294,1623.397
Retention time (min):	24.50
Expected Mass:	66518.100
Area (count*min):	-
Intensity (counts):	-
Amount:	-

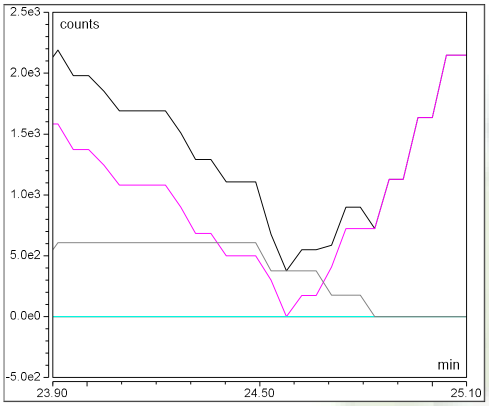

© 2009-2020 Thermo Fisher Scientific Inc. All rights reserved.
Chromeleon 7.3.0.60919

Component Name:	AAV5_VP2
Quantitation ions:	1389.997,1519.197,1484.692,1420.196,1555.348,1451.727
Retention time (min):	25.16
Expected Mass:	65282.690
Area (count*min):	-
Intensity (counts):	-
Amount:	-

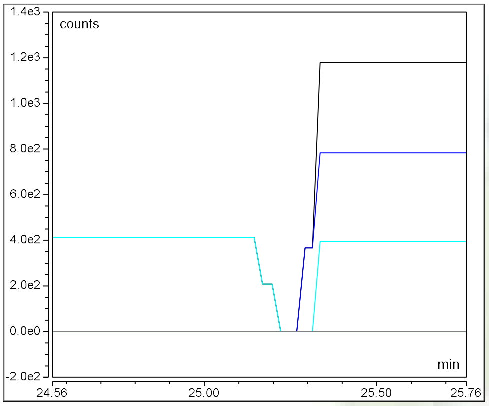

© 2009-2020 Thermo Fisher Scientific Inc. All rights reserved.
Chromeleon 7.3.0.60919

Component Name: AAV2_VP1	
Quantitation ions:	1388.434,1545.445,1489.336,1342.917,1365.283,1412.362
Retention time (min):	25.18
Expected Mass:	81854.900
Area (count*min):	13464
Intensity (counts):	22733
Amount:	-

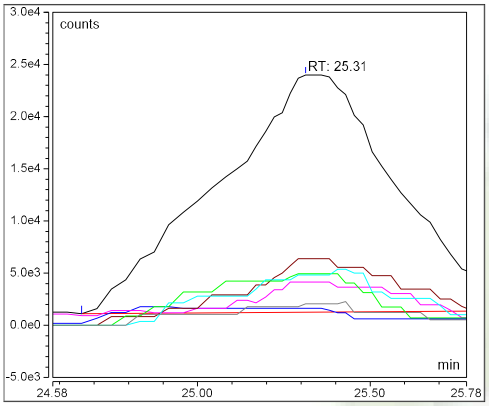

© 2009-2020 Thermo Fisher Scientific Inc. All rights reserved.
Chromeleon 7.3.0.60919

Component Name:	AAV2_VP2
Quantitation ions:	1415.636,1446.415,1255.530,1478.506,1279.609,1304.686
Retention time (min):	26.20
Expected Mass:	66487.900
Area (count*min):	15927
Intensity (counts):	26899
Amount:	-

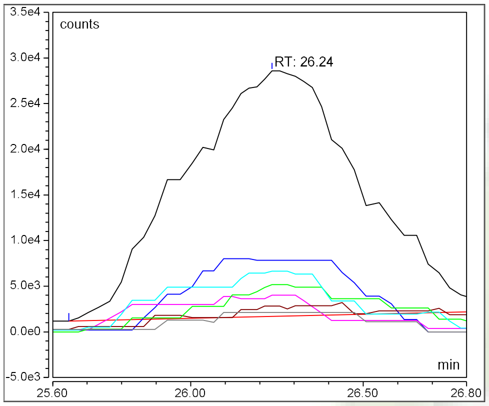

© 2009-2020 Thermo Fisher Scientific Inc. All rights reserved.
Chromeleon 7.3.0.60919

Chromatogram and SST Results

Injection Details

Injection Name:	**20211011_AAV2 E_45C_65-59B_Mod1_1ug_1**	*Run Time (min):*	**45.00**
Vial Number:	**R:A1**	*Injection Volume:*	**2.86**
Injection Type:	**Unknown**		
Calibration Level:			
Instrument Method:	**AAV Cap_Prot_Sep_BEH Glycoprotein Amide HILIC_1x150mm_FLR + MS_CT45C_65-59_S**		
Processing Method:	**MS Quantitative_45C_65-59B**	*Dilution Factor:*	**1.0000**
Injection Date/Time:	**12/Oct/21 21:57**	*Sample Weight:*	**1.0000**

Chromatogram

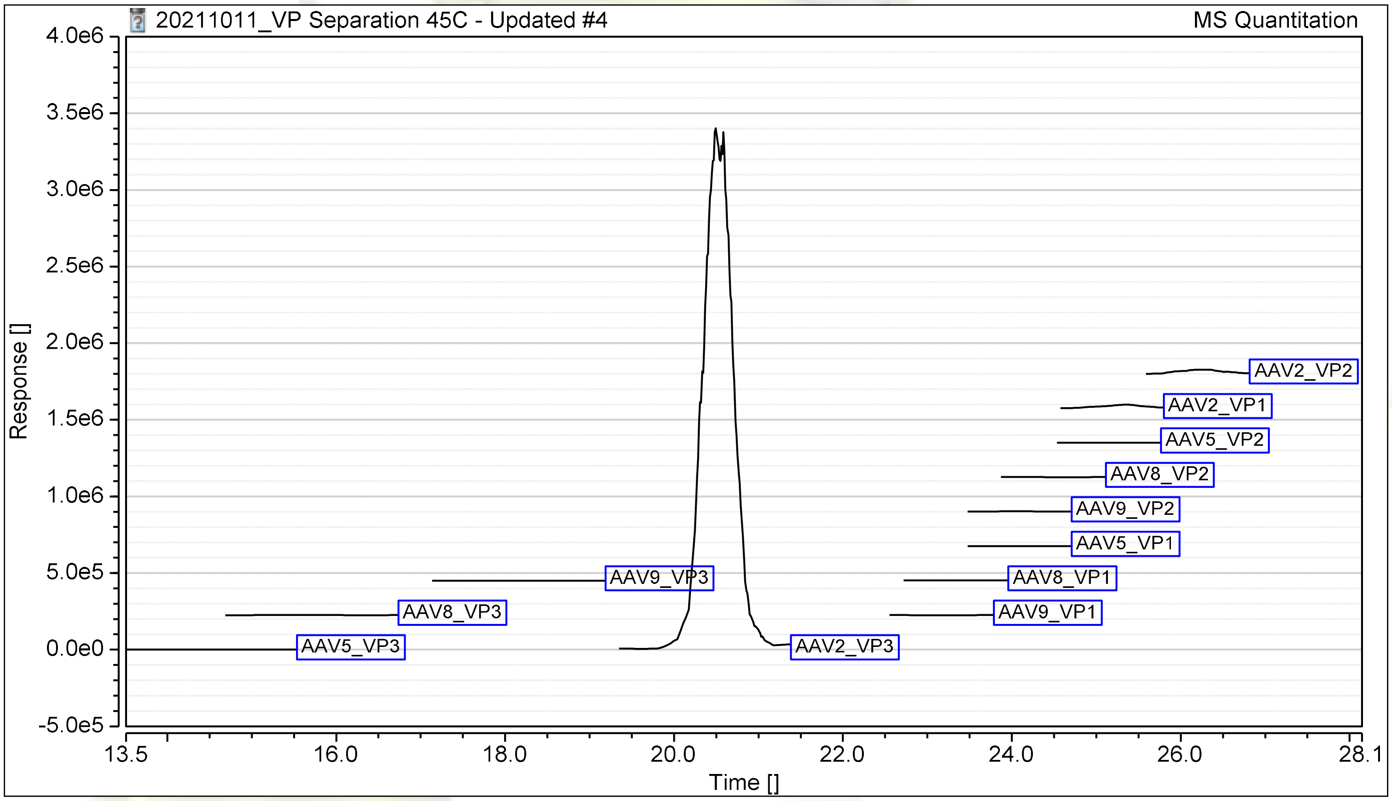

SST Results

No.	Name	Inj.Condition	Peak	Test Result	Injection

Number of executed test cases: **n.a.** **Total Result:** **Passed**

© 2009-2020 Thermo Fisher Scientific Inc. All rights reserved.
Chromeleon 7.3.0.60919

Protein Deconvolution Summary

Injection Details

Injection Name:	**20211011_AAV2 E_45C_65-59B_Mod1_1ug_2**	*Injection Volume:*	**2.86**
Vial Number:	**R:A1**	*Instrument Method:*	**AAV Cap_Prot_Sep_BEH Glycoprotein Amide H**
Injection Type:	**Unknown**	*Processing Method:*	**MS Quantitative_45C_65-59B**
Run Time (min):	**44.99**	*Injection Date/Time:*	**12/Oct/21 22:43**

Chromatogram

Source Spectrum

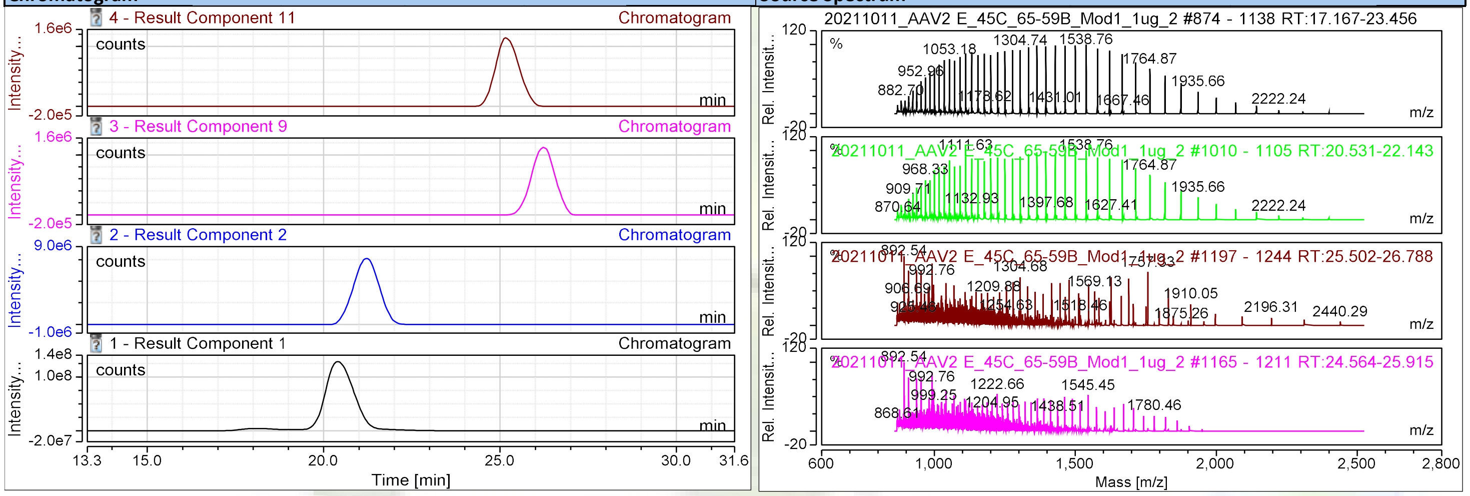

Deconvoluted Spectrum

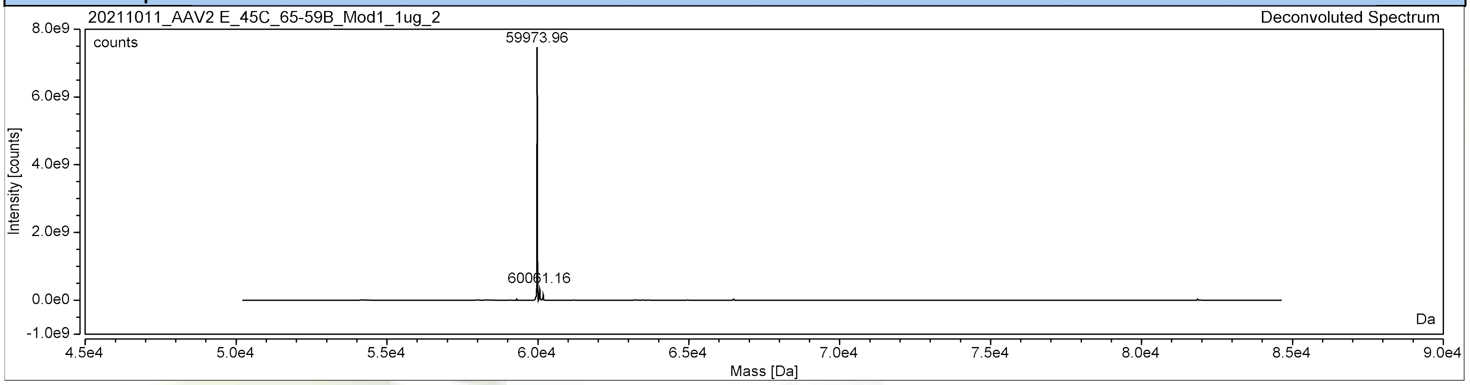

Deconvolution Results

Result Component	RT min	Mass Da	Std. Dev. Da	Std. Dev. ppm	Delta Mass Da	Intensity counts	Fractional Abundance counts	Rel. Abundance %	Number of Charge States	Score
Result Component 1	18.11	59733.033	0.40	6.68	0.000	27034264760	80.1	100.00	45	238.14
Result Component 2	18.22	59820.302	0.95	15.91	87.269	1527586438	4.5	5.65	40	170.79
Result Component 3	23.18	81291.415	0.91	11.13	21558.382	643910272	1.9	2.38	56	291.15
Result Component 4	19.20	59813.944	0.85	14.29	80.911	471832605	1.4	1.75	43	234.57
Result Component 5	18.27	59686.960	1.22	20.37	-46.073	390164875	1.2	1.44	32	176.14
Result Component 7	24.10	66210.268	0.85	12.85	6477.235	340089971	1.0	1.26	44	239.41
Result Component 8	21.47	59731.938	0.90	15.10	-1.095	300593658	0.9	1.11	41	217.18
Result Component 9	13.31	59733.070	0.64	10.75	0.037	298107673	0.9	1.10	42	257.50
Result Component 11	19.18	59933.627	1.10	18.41	200.594	200302659	0.6	0.74	40	199.85
Result Component 13	17.92	59687.388	1.55	25.99	-45.645	162773683	0.5	0.60	31	170.85
Result Component 15	21.21	59731.785	0.70	11.70	-1.249	129722977	0.4	0.48	41	225.58
Result Component 17	19.13	59766.513	1.08	18.11	33.479	91587642	0.3	0.34	38	197.64
Result Component 18	23.54	81372.238	1.92	23.54	21639.205	84227912	0.2	0.31	50	158.32
Result Component 19	18.85	59816.211	1.65	27.63	83.178	73795145	0.2	0.27	39	170.82
Result Component 20	19.45	59690.811	1.05	17.65	-42.222	72092544	0.2	0.27	37	195.30
Result Component 21	24.66	66290.558	1.28	19.26	6557.525	43412692	0.1	0.16	41	172.17

© 2009-2020 Thermo Fisher Scientific Inc. All rights reserved.
Chromeleon 7.3.0.60919

Component Summary

Injection Details

Injection Name:	**20211011_AAV2 E_45C_65-59B_Mod1_1ug**	*Injection Volume:*	**2.86**
Vial Number:	**R:A1**	*Instrument Method:*	**AAV Cap_Prot_Sep_BEH Glycoprotein Amide HILIC_1x150mm**
Injection Type:	**Unknown**	*Processing Method:*	**MS Quantitative_45C_65-59B**
Run Time (min):	**44.99**	*Injection Date/Time:*	**12/Oct/21 22:43**

Peak Name	Retention Time min	Area counts*min	Relative Area %	Quantitation Ions m/z	Mol.Mass	Mass Confirmation Pass/Fail
AAV2_VP3	20.463	1500924.040	74.26	1428.955,1500.355,1463.789,1395.748,1364.055,1538.792	59973.780	**PASS**
AAV2_VP1	25.294	10480.860	0.52	1388.434,1545.445,1489.336,1342.917,1365.283,1412.362	81854.900	**PASS**
AAV2_VP2	26.228	20760.822	1.03	1415.636,1446.415,1255.530,1478.506,1279.609,1304.686	66487.900	**PASS**
			**75.80**			

Component Name: AAV5_VP3

Quantitation ions:	1525.669,1565.784,1608.079,1451.304,1487.553,1416.767
Retention time (min):	14.53
Expected Mass:	59462.370
Area (count*min):	-
Intensity (counts):	-
Amount:	-

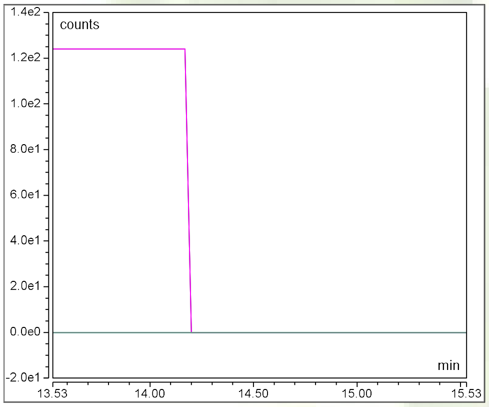

© 2009-2020 Thermo Fisher Scientific Inc. All rights reserved.
Chromeleon 7.3.0.60919

Component Name:	AAV8_VP3
Quantitation ions:	1574.800,1496.109,1534.442,1459.645,1273.445,1391.802
Retention time (min):	15.73
Expected Mass:	59804.680
Area (count*min):	-
Intensity (counts):	-
Amount:	-

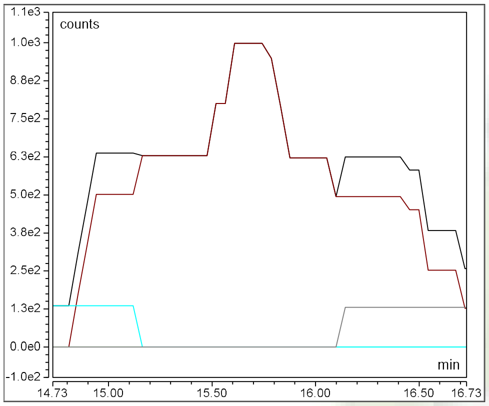

© 2009-2020 Thermo Fisher Scientific Inc. All rights reserved.
Chromeleon 7.3.0.60919

Component Name:	AAV9_VP3
Quantitation ions:	1358.581,1390.150,1299.556,1328.415,1271.930,1457.907
Retention time (min):	18.15
Expected Mass:	59732.620
Area (count*min):	-
Intensity (counts):	-
Amount:	-

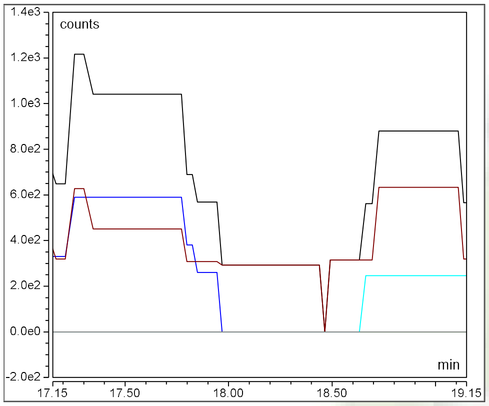

© 2009-2020 Thermo Fisher Scientific Inc. All rights reserved.
Chromeleon 7.3.0.60919

Component Name: AAV2_VP3	
Quantitation ions:	1428.955,1500.355,1463.789,1395.748,1364.055,1538.792
Retention time (min):	20.38
Expected Mass:	59973.780
Area (count*min):	1500924
Intensity (counts):	4051828
Amount:	-

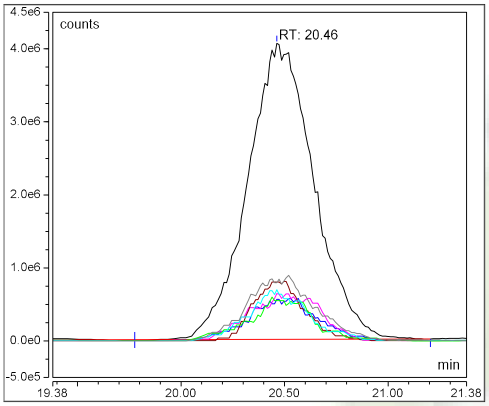

© 2009-2020 Thermo Fisher Scientific Inc. All rights reserved.
Chromeleon 7.3.0.60919

Component Name: AAV9_VP1

Quantitation ions:	1378.820,1333.651,1355.868,1402.584,1534.808,1452.636
Retention time (min):	23.16
Expected Mass:	81290.340
Area (count*min):	-
Intensity (counts):	-
Amount:	-

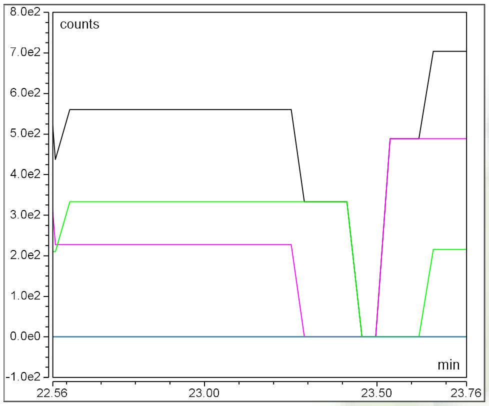

© 2009-2020 Thermo Fisher Scientific Inc. All rights reserved.
Chromeleon 7.3.0.60919

Component Name:	AAV8_VP1
Quantitation ions:	1409.080,1459.382,1385.209,1513.388,1184.605,1339.828
Retention time (min):	23.33
Expected Mass:	81666.710
Area (count*min):	-
Intensity (counts):	-
Amount:	-

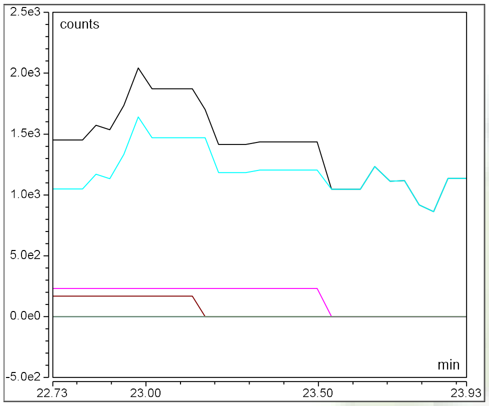

© 2009-2020 Thermo Fisher Scientific Inc. All rights reserved.
Chromeleon 7.3.0.60919

Component Name:	AAV5_VP1
Quantitation ions:	1362.628,1410.415,1435.577,1317.996,1339.949,1488.697
Retention time (min):	24.09
Expected Mass:	80335.080
Area (count*min):	-
Intensity (counts):	-
Amount:	-

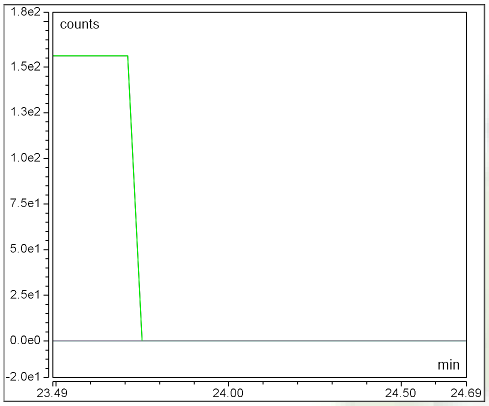

© 2009-2020 Thermo Fisher Scientific Inc. All rights reserved.
Chromeleon 7.3.0.60919

Component Name:	AAV9_VP2
Quantitation ions:	1409.730,1162.589,1472.334,1274.287,1380.371,1352.231
Retention time (min):	24.10
Expected Mass:	66209.760
Area (count*min):	-
Intensity (counts):	-
Amount:	-

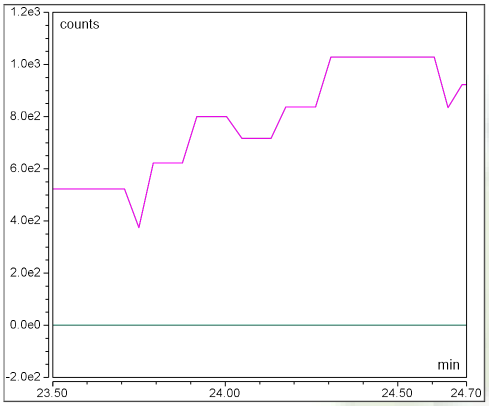

© 2009-2020 Thermo Fisher Scientific Inc. All rights reserved.
Chromeleon 7.3.0.60919

Component Name:	AAV8_VP2
Quantitation ions:	1386.809,1358.524,1331.393,1305.308,1416.294,1623.397
Retention time (min):	24.50
Expected Mass:	66518.100
Area (count*min):	-
Intensity (counts):	-
Amount:	-

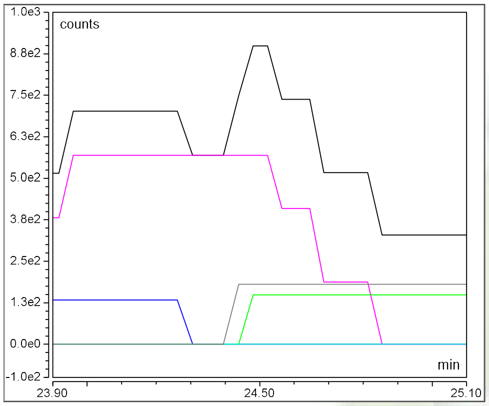

© 2009-2020 Thermo Fisher Scientific Inc. All rights reserved.
Chromeleon 7.3.0.60919

Component Name: AAV5_VP2

Quantitation ions:	1389.997,1519.197,1484.692,1420.196,1555.348,1451.727
Retention time (min):	25.16
Expected Mass:	65282.690
Area (count*min):	-
Intensity (counts):	-
Amount:	-

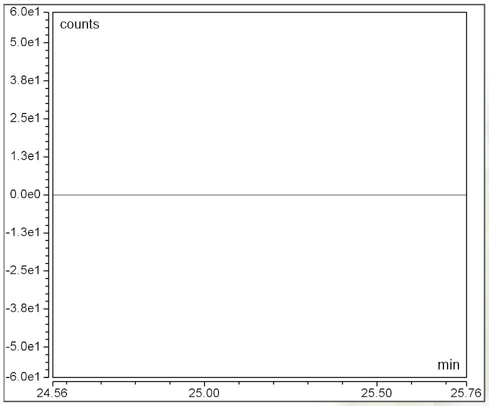

© 2009-2020 Thermo Fisher Scientific Inc. All rights reserved.
Chromeleon 7.3.0.60919

Component Name:	AAV2_VP1
Quantitation ions:	1388.434,1545.445,1489.336,1342.917,1365.283,1412.362
Retention time (min):	25.18
Expected Mass:	81854.900
Area (count*min):	10481
Intensity (counts):	18929
Amount:	-

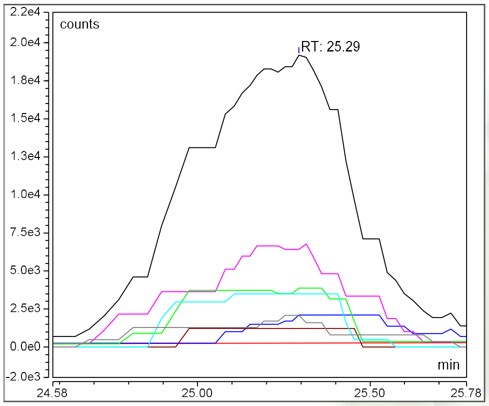

© 2009-2020 Thermo Fisher Scientific Inc. All rights reserved.
Chromeleon 7.3.0.60919

Component Name:	AAV2_VP2
Quantitation ions:	1415.636,1446.415,1255.530,1478.506,1279.609,1304.686
Retention time (min):	26.20
Expected Mass:	66487.900
Area (count*min):	20761
Intensity (counts):	38577
Amount:	-

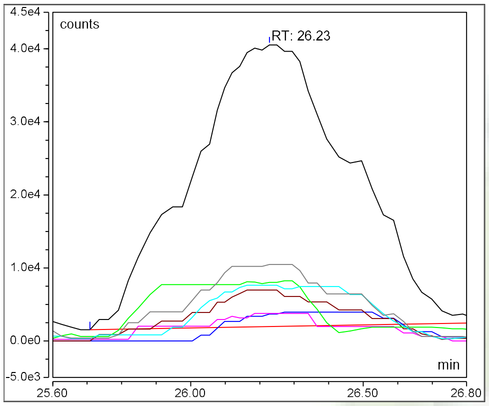

© 2009-2020 Thermo Fisher Scientific Inc. All rights reserved.
Chromeleon 7.3.0.60919

Chromatogram and SST Results

Injection Details

Injection Name:	**20211011_AAV2 E_45C_65-59B_Mod1_1ug_2**	*Run Time (min):*	**44.99**
Vial Number:	**R:A1**	*Injection Volume:*	**2.86**
Injection Type:	**Unknown**		
Calibration Level:			
Instrument Method:	**AAV Cap_Prot_Sep_BEH Glycoprotein Amide HILIC_1x150mm_FLR + MS_CT45C_65-59_S**		
Processing Method:	**MS Quantitative_45C_65-59B**	*Dilution Factor:*	**1.0000**
Injection Date/Time:	**12/Oct/21 22:43**	*Sample Weight:*	**1.0000**

Chromatogram

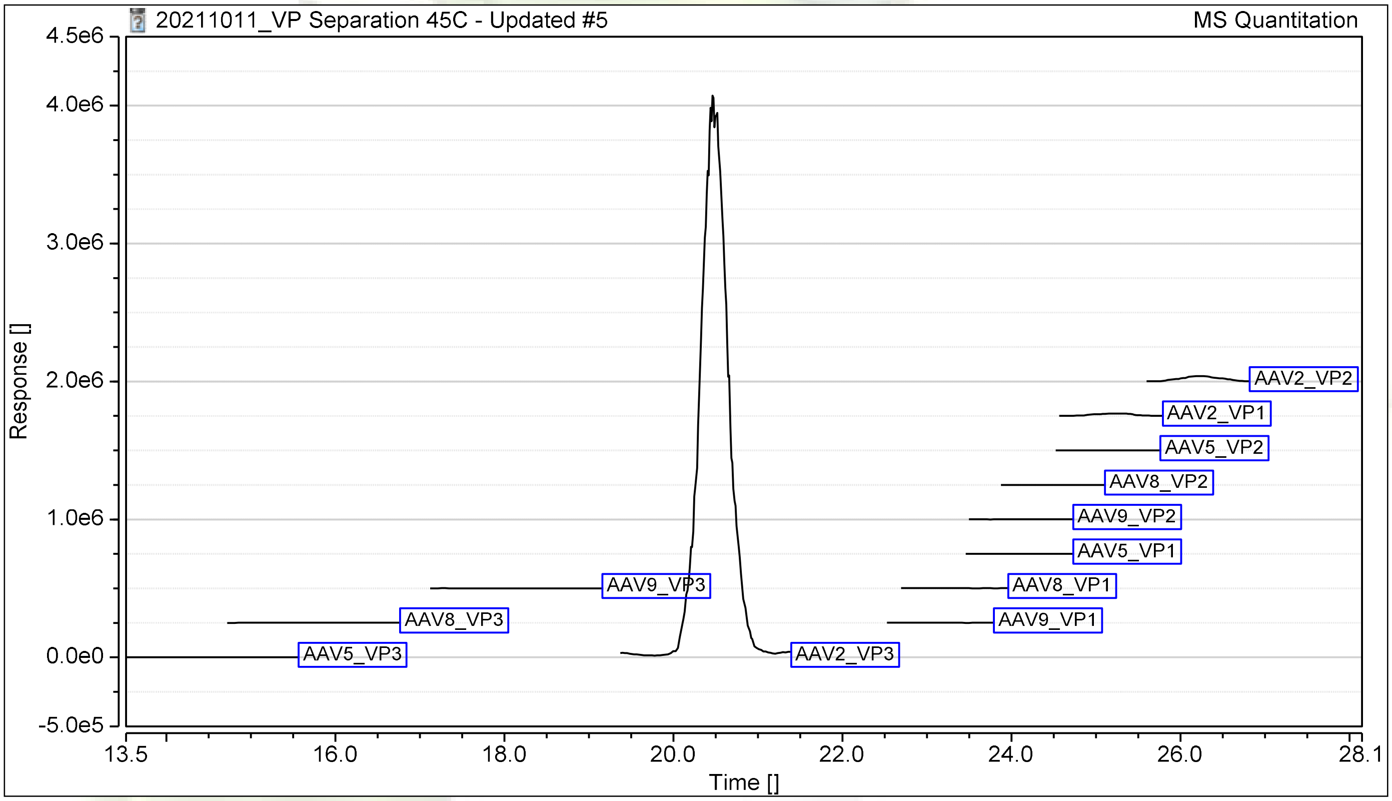

SST Results

No.	Name	Inj.Condition	Peak	Test Result	Injection

Number of executed test cases: **n.a.** **Total Result:** **Passed**

© 2009-2020 Thermo Fisher Scientific Inc. All rights reserved.
Chromeleon 7.3.0.60919

Protein Deconvolution Summary

Injection Details

Injection Name:	**20211011_AAV2 E_45C_65-59B_Mod1_1ug_3**	*Injection Volume:*	**2.86**
Vial Number:	**R:A1**	*Instrument Method:*	**AAV Cap_Prot_Sep_BEH Glycoprotein Amide H**
Injection Type:	**Unknown**	*Processing Method:*	**MS Quantitative_45C_65-59B**
Run Time (min):	**45.00**	*Injection Date/Time:*	**12/Oct/21 23:29**

Chromatogram

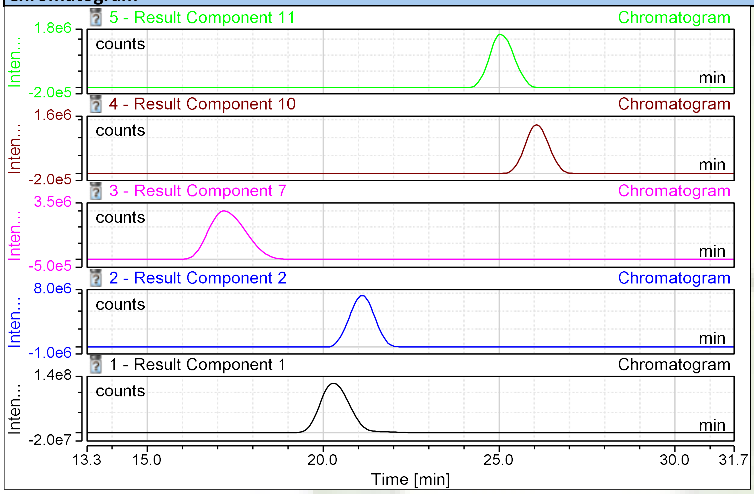

Source Spectrum

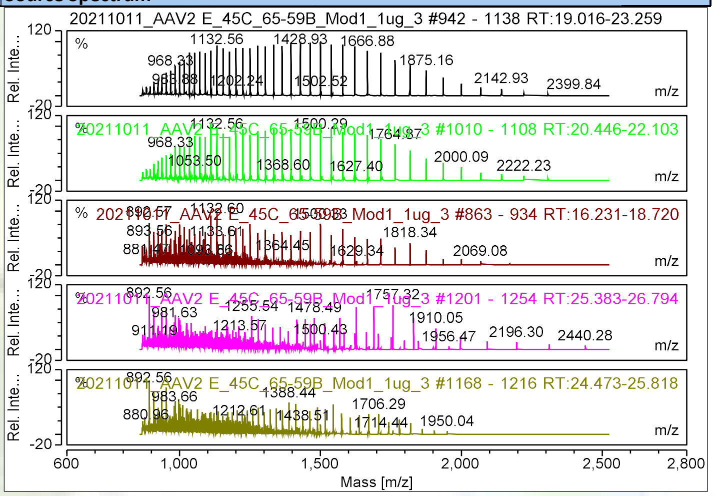

Deconvoluted Spectrum

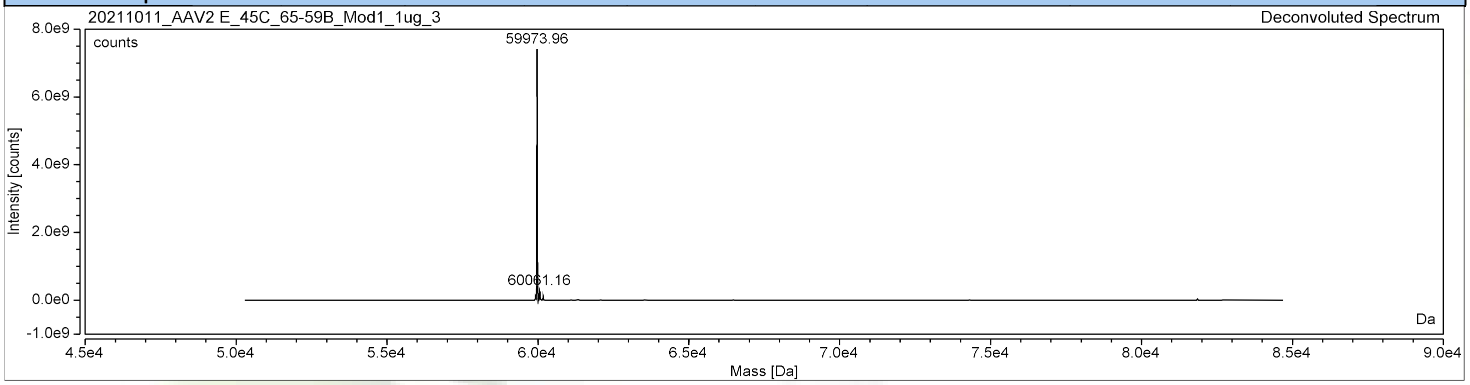

Deconvolution Results

Result Component	RT min	Mass Da	Std. Dev. Da	Std. Dev. ppm	Delta Mass Da	Intensity counts	Fractional Abundance counts	Rel. Abundance %	Number of Charge States	Score
Result Component 1	18.11	59733.033	0.40	6.68	0.000	27034264760	80.1	100.00	45	238.14
Result Component 2	18.22	59820.302	0.95	15.91	87.269	1527586438	4.5	5.65	40	170.79
Result Component 3	23.18	81291.415	0.91	11.13	21558.382	643910272	1.9	2.38	56	291.15
Result Component 4	19.20	59813.944	0.85	14.29	80.911	471832605	1.4	1.75	43	234.57
Result Component 5	18.27	59686.960	1.22	20.37	-46.073	390164875	1.2	1.44	32	176.14
Result Component 7	24.10	66210.268	0.85	12.85	6477.235	340089971	1.0	1.26	44	239.41
Result Component 8	21.47	59731.938	0.90	15.10	-1.095	300593658	0.9	1.11	41	217.18
Result Component 9	13.31	59733.070	0.64	10.75	0.037	298107673	0.9	1.10	42	257.50
Result Component 11	19.18	59933.627	1.10	18.41	200.594	200302659	0.6	0.74	40	199.85
Result Component 13	17.92	59687.388	1.55	25.99	-45.645	162773683	0.5	0.60	31	170.85
Result Component 15	21.21	59731.785	0.70	11.70	-1.249	129722977	0.4	0.48	41	225.58
Result Component 17	19.13	59766.513	1.08	18.11	33.479	91587642	0.3	0.34	38	197.64
Result Component 18	23.54	81372.238	1.92	23.54	21639.205	84227912	0.2	0.31	50	158.32
Result Component 19	18.85	59816.211	1.65	27.63	83.178	73795145	0.2	0.27	39	170.82
Result Component 20	19.45	59690.811	1.05	17.65	-42.222	72092544	0.2	0.27	37	195.30
Result Component 21	24.66	66290.558	1.28	19.26	6557.525	43412692	0.1	0.16	41	172.17

© 2009-2020 Thermo Fisher Scientific Inc. All rights reserved.
Chromeleon 7.3.0.60919

Component Summary

Injection Details

Injection Name:	**20211011_AAV2 E_45C_65-59B_Mod1_1ug**	*Injection Volume:*	**2.86**
Vial Number:	**R:A1**	*Instrument Method:*	**AAV Cap_Prot_Sep_BEH Glycoprotein Amide HILIC_1x150mm**
Injection Type:	**Unknown**	*Processing Method:*	**MS Quantitative_45C_65-59B**
Run Time (min):	**45.00**	*Injection Date/Time:*	**12/Oct/21 23:29**

Peak Name	Retention Time min	Area counts*min	Relative Area %	Quantitation Ions m/z	Mol.Mass	Mass Confirmation Pass/Fail
AAV2_VP3	20.331	1247828.061	87.99	1428.955,1500.355,1463.789,1395.748,1364.055,1538.792	59973.780	**PASS**
AAV2_VP1	25.120	11832.608	0.83	1388.434,1545.445,1489.336,1342.917,1365.283,1412.362	81854.900	**PASS**
AAV2_VP2	26.051	16781.587	1.18	1415.636,1446.415,1255.530,1478.506,1279.609,1304.686	66487.900	**PASS**
			**90.01**			

Component Name: AAV5_VP3

Quantitation ions:	1525.669,1565.784,1608.079,1451.304,1487.553,1416.767
Retention time (min):	14.53
Expected Mass:	59462.370
Area (count*min):	-
Intensity (counts):	-
Amount:	-

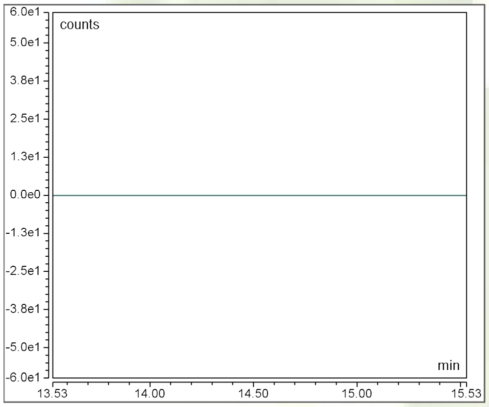

© 2009-2020 Thermo Fisher Scientific Inc. All rights reserved.
Chromeleon 7.3.0.60919

Component Name:	AAV8_VP3
Quantitation ions:	1574.800,1496.109,1534.442,1459.645,1273.445,1391.802
Retention time (min):	15.73
Expected Mass:	59804.680
Area (count*min):	-
Intensity (counts):	-
Amount:	-

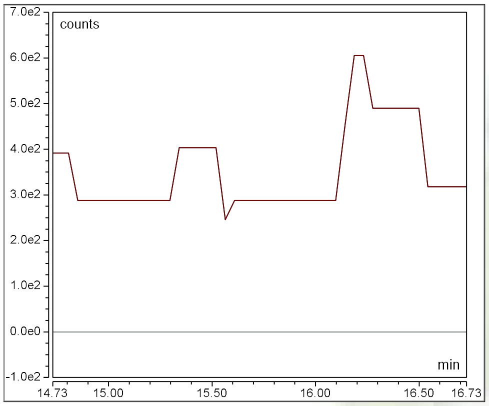

© 2009-2020 Thermo Fisher Scientific Inc. All rights reserved.
Chromeleon 7.3.0.60919

Component Name:	AAV9_VP3
Quantitation ions:	1358.581,1390.150,1299.556,1328.415,1271.930,1457.907
Retention time (min):	18.15
Expected Mass:	59732.620
Area (count*min):	-
Intensity (counts):	-
Amount:	-

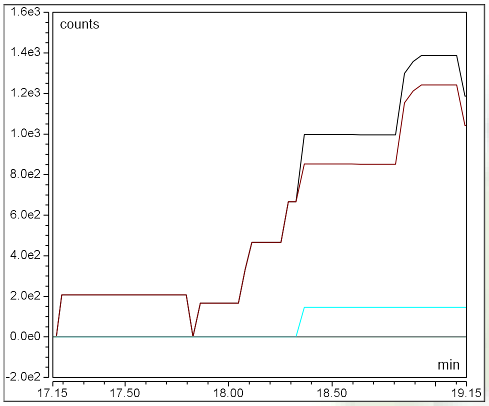

© 2009-2020 Thermo Fisher Scientific Inc. All rights reserved.
Chromeleon 7.3.0.60919

Component Name:	AAV2_VP3
Quantitation ions:	1428.955,1500.355,1463.789,1395.748,1364.055,1538.792
Retention time (min):	20.38
Expected Mass:	59973.780
Area (count*min):	1247828
Intensity (counts):	3256317
Amount:	-

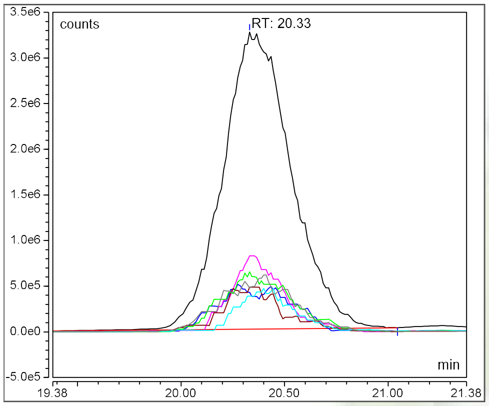

© 2009-2020 Thermo Fisher Scientific Inc. All rights reserved.
Chromeleon 7.3.0.60919

Component Name: AAV9_VP1

Quantitation ions:	1378.820,1333.651,1355.868,1402.584,1534.808,1452.636
Retention time (min):	23.16
Expected Mass:	81290.340
Area (count*min):	-
Intensity (counts):	-
Amount:	-

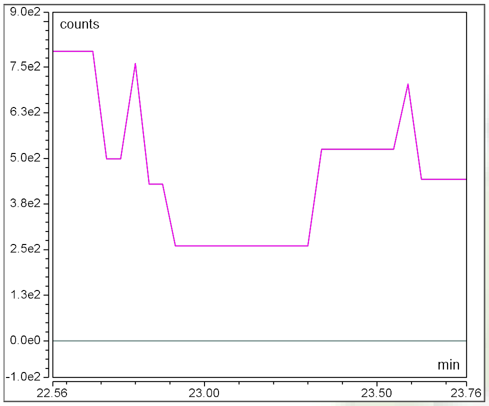

© 2009-2020 Thermo Fisher Scientific Inc. All rights reserved.
Chromeleon 7.3.0.60919

Component Name:	AAV8_VP1
Quantitation ions:	1409.080,1459.382,1385.209,1513.388,1184.605,1339.828
Retention time (min):	23.33
Expected Mass:	81666.710
Area (count*min):	-
Intensity (counts):	-
Amount:	-

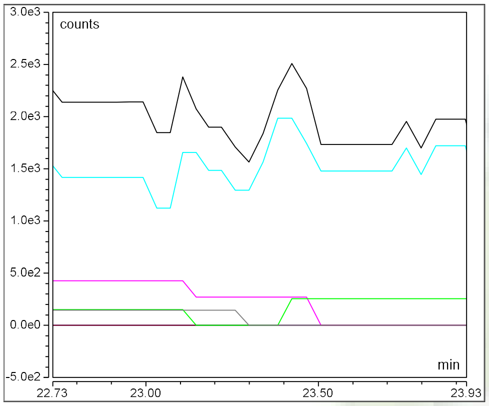

© 2009-2020 Thermo Fisher Scientific Inc. All rights reserved.
Chromeleon 7.3.0.60919

Component Name:	AAV5_VP1
Quantitation ions:	1362.628,1410.415,1435.577,1317.996,1339.949,1488.697
Retention time (min):	24.09
Expected Mass:	80335.080
Area (count*min):	-
Intensity (counts):	-
Amount:	-

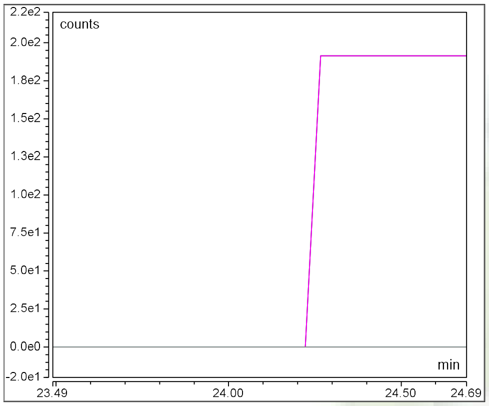

© 2009-2020 Thermo Fisher Scientific Inc. All rights reserved.
Chromeleon 7.3.0.60919

Component Name: AAV9_VP2

Quantitation ions:	1409.730,1162.589,1472.334,1274.287,1380.371,1352.231
Retention time (min):	24.10
Expected Mass:	66209.760
Area (count*min):	-
Intensity (counts):	-
Amount:	-

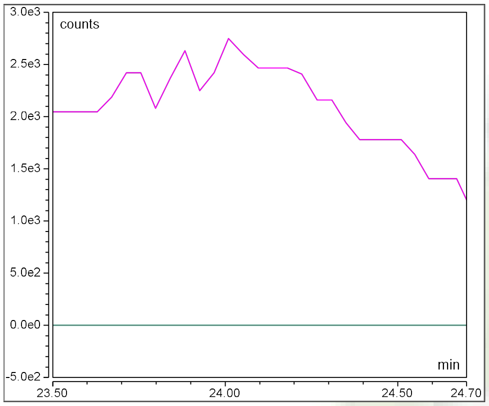

© 2009-2020 Thermo Fisher Scientific Inc. All rights reserved.
Chromeleon 7.3.0.60919

Component Name:	AAV8_VP2
Quantitation ions:	1386.809,1358.524,1331.393,1305.308,1416.294,1623.397
Retention time (min):	24.50
Expected Mass:	66518.100
Area (count*min):	-
Intensity (counts):	-
Amount:	-

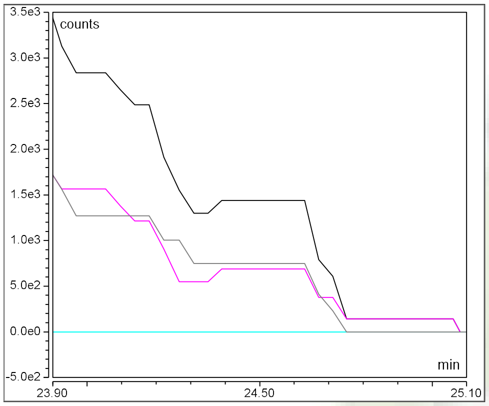

© 2009-2020 Thermo Fisher Scientific Inc. All rights reserved.
Chromeleon 7.3.0.60919

Component Name: AAV5_VP2

Quantitation ions:	1389.997,1519.197,1484.692,1420.196,1555.348,1451.727
Retention time (min):	25.16
Expected Mass:	65282.690
Area (count*min):	-
Intensity (counts):	-
Amount:	-

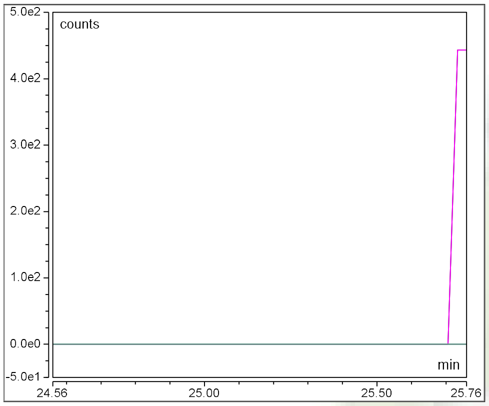

© 2009-2020 Thermo Fisher Scientific Inc. All rights reserved.
Chromeleon 7.3.0.60919

Component Name:	AAV2_VP1
Quantitation ions:	1388.434,1545.445,1489.336,1342.917,1365.283,1412.362
Retention time (min):	25.18
Expected Mass:	81854.900
Area (count*min):	11833
Intensity (counts):	20477
Amount:	-

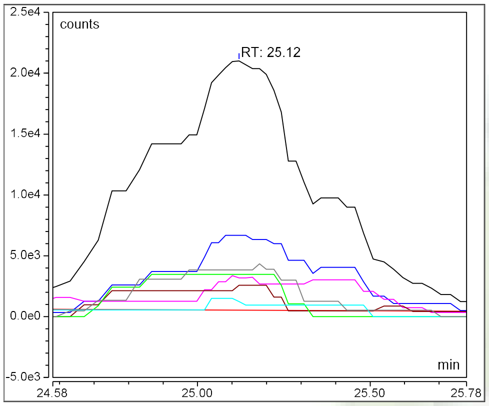

© 2009-2020 Thermo Fisher Scientific Inc. All rights reserved.
Chromeleon 7.3.0.60919

Component Name:	AAV2_VP2
Quantitation ions:	1415.636,1446.415,1255.530,1478.506,1279.609,1304.686
Retention time (min):	26.20
Expected Mass:	66487.900
Area (count*min):	16782
Intensity (counts):	32732
Amount:	-

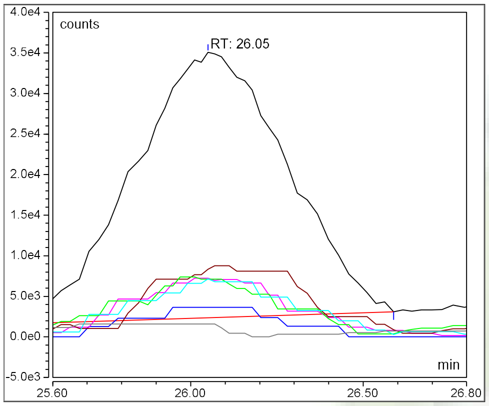

© 2009-2020 Thermo Fisher Scientific Inc. All rights reserved.
Chromeleon 7.3.0.60919

Chromatogram and SST Results

Injection Details

Injection Name:	**20211011_AAV2 E_45C_65-59B_Mod1_1ug_3**	*Run Time (min):*	**45.00**
Vial Number:	**R:A1**	*Injection Volume:*	**2.86**
Injection Type:	**Unknown**		
Calibration Level:			
Instrument Method:	**AAV Cap_Prot_Sep_BEH Glycoprotein Amide HILIC_1x150mm_FLR + MS_CT45C_65-59_S**		
Processing Method:	**MS Quantitative_45C_65-59B**	*Dilution Factor:*	**1.0000**
Injection Date/Time:	**12/Oct/21 23:29**	*Sample Weight:*	**1.0000**

Chromatogram

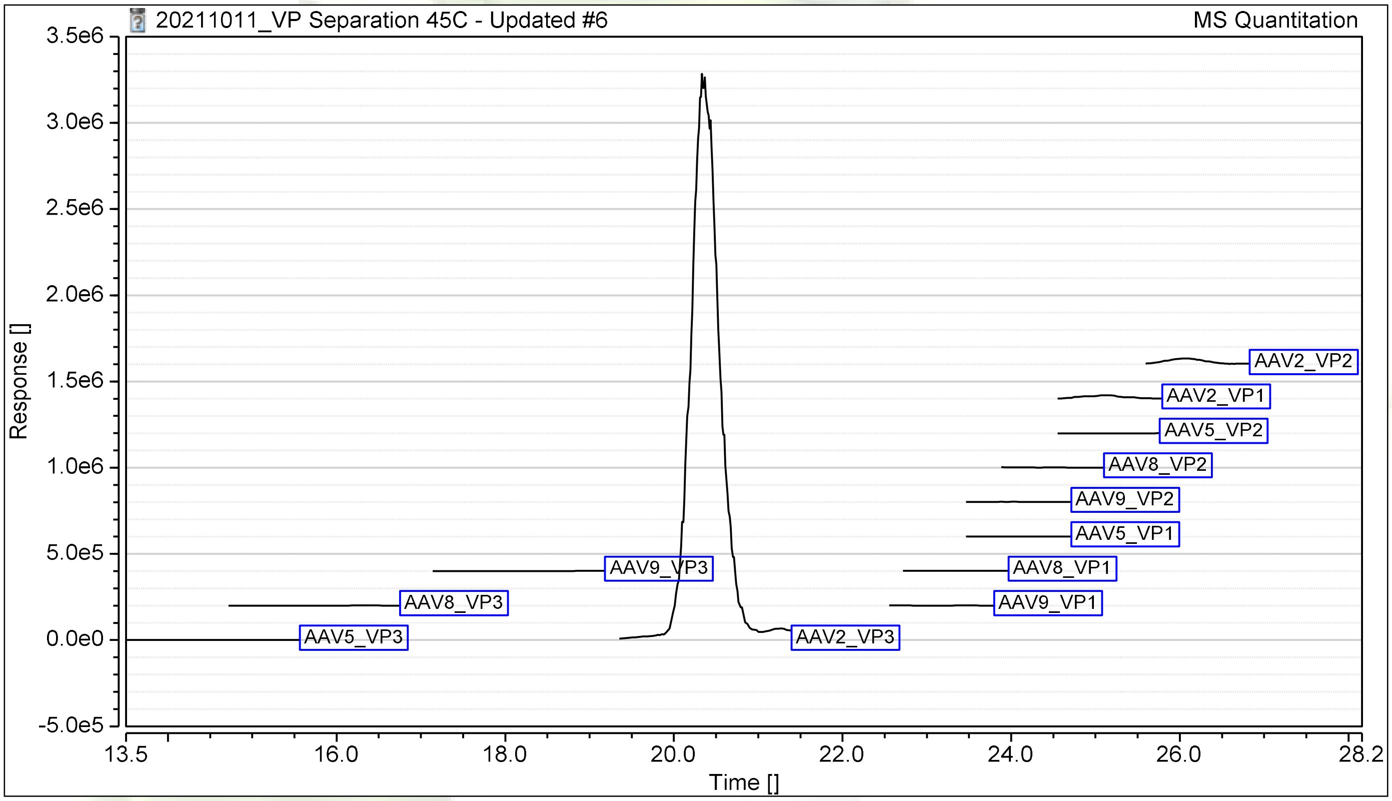

SST Results

No.	Name	Inj.Condition	Peak	Test Result	Injection

Number of executed test cases: **n.a.** **Total Result:** **Passed**

© 2009-2020 Thermo Fisher Scientific Inc. All rights reserved.
Chromeleon 7.3.0.60919

Protein Deconvolution Summary

Injection Details

Injection Name:	**20211011_AAV8 E_45C_65-59B_Mod1_1ug_1**	*Injection Volume:*	**6.25**
Vial Number:	**R:A3**	*Instrument Method:*	**AAV Cap_Prot_Sep_BEH Glycoprotein Amide H**
Injection Type:	**Unknown**	*Processing Method:*	**MS Quantitative_45C_65-59B**
Run Time (min):	**45.00**	*Injection Date/Time:*	**13/Oct/21 01:42**

Chromatogram

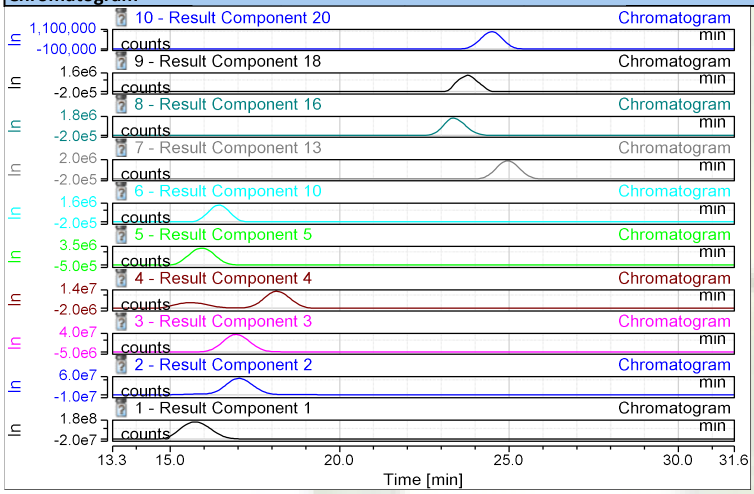

Source Spectrum

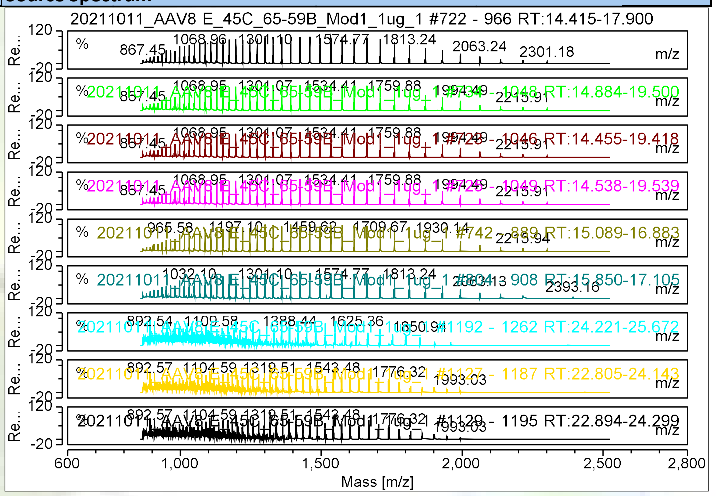

Deconvoluted Spectrum

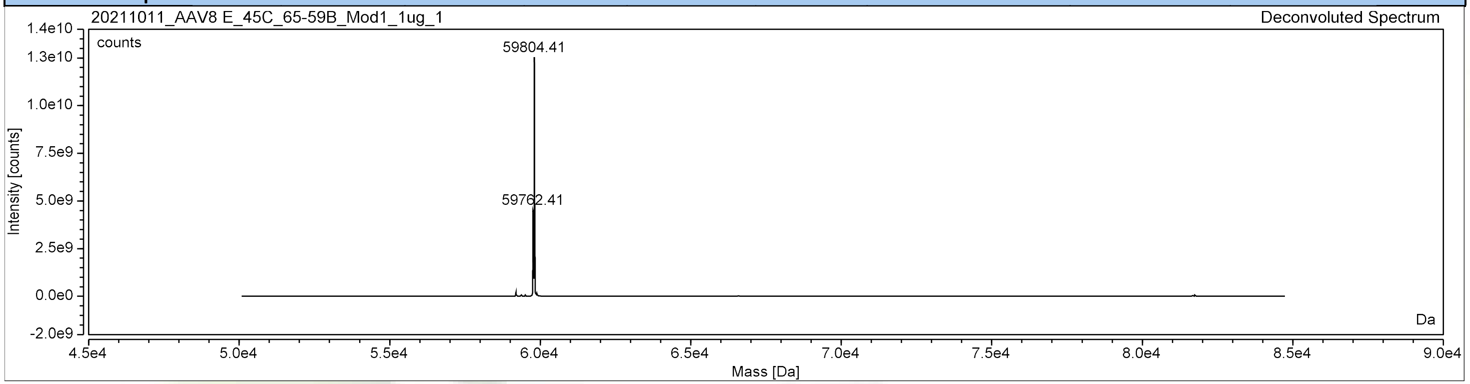

Deconvolution Results

Result Component	RT min	Mass Da	Std. Dev. Da	Std. Dev. ppm	Delta Mass Da	Intensity counts	Fractional Abundance counts	Rel. Abundance %	Number of Charge States	Score
Result Component 1	18.11	59733.033	0.40	6.68	0.000	27034264760	80.1	100.00	45	238.14
Result Component 2	18.22	59820.302	0.95	15.91	87.269	1527586438	4.5	5.65	40	170.79
Result Component 3	23.18	81291.415	0.91	11.13	21558.382	643910272	1.9	2.38	56	291.15
Result Component 4	19.20	59813.944	0.85	14.29	80.911	471832605	1.4	1.75	43	234.57
Result Component 5	18.27	59686.960	1.22	20.37	-46.073	390164875	1.2	1.44	32	176.14
Result Component 7	24.10	66210.268	0.85	12.85	6477.235	340089971	1.0	1.26	44	239.41
Result Component 8	21.47	59731.938	0.90	15.10	-1.095	300593658	0.9	1.11	41	217.18
Result Component 9	13.31	59733.070	0.64	10.75	0.037	298107673	0.9	1.10	42	257.50
Result Component 11	19.18	59933.627	1.10	18.41	200.594	200302659	0.6	0.74	40	199.85
Result Component 13	17.92	59687.388	1.55	25.99	-45.645	162773683	0.5	0.60	31	170.85
Result Component 15	21.21	59731.785	0.70	11.70	-1.249	129722977	0.4	0.48	41	225.58
Result Component 17	19.13	59766.513	1.08	18.11	33.479	91587642	0.3	0.34	38	197.64
Result Component 18	23.54	81372.238	1.92	23.54	21639.205	84227912	0.2	0.31	50	158.32
Result Component 19	18.85	59816.211	1.65	27.63	83.178	73795145	0.2	0.27	39	170.82
Result Component 20	19.45	59690.811	1.05	17.65	-42.222	72092544	0.2	0.27	37	195.30
Result Component 21	24.66	66290.558	1.28	19.26	6557.525	43412692	0.1	0.16	41	172.17

© 2009-2020 Thermo Fisher Scientific Inc. All rights reserved.
Chromeleon 7.3.0.60919

Component Summary

Injection Details

Injection Name:	**20211011_AAV8 E_45C_65-59B_Mod1_1ug**	*Injection Volume:*	**6.25**
Vial Number:	**R:A3**	*Instrument Method:*	**AAV Cap_Prot_Sep_BEH Glycoprotein Amide HILIC_1x150mm**
Injection Type:	**Unknown**	*Processing Method:*	**MS Quantitative_45C_65-59B**
Run Time (min):	**45.00**	*Injection Date/Time:*	**13/Oct/21 01:42**

Peak Name	Retention Time min	Area counts*min	Relative Area %	Quantitation Ions m/z	Mol.Mass	Mass Confirmation Pass/Fail
AAV8_VP3	15.727	1734029.695	78.06	1574.800,1496.109,1534.442,1459.645,1273.445,1391.802	59804.680	**PASS**
AAV8_VP1	23.494	21159.537	0.95	1409.080,1459.382,1385.209,1513.388,1184.605,1339.828	81666.710	**PASS**
AAV8_VP2	24.467	14492.280	0.65	1386.809,1358.524,1331.393,1305.308,1416.294,1623.397	66518.100	**PASS**
			**79.66**			

Component Name: AAV5_VP3

Quantitation ions:	1525.669,1565.784,1608.079,1451.304,1487.553,1416.767
Retention time (min):	14.53
Expected Mass:	59462.370
Area (count*min):	-
Intensity (counts):	-
Amount:	-

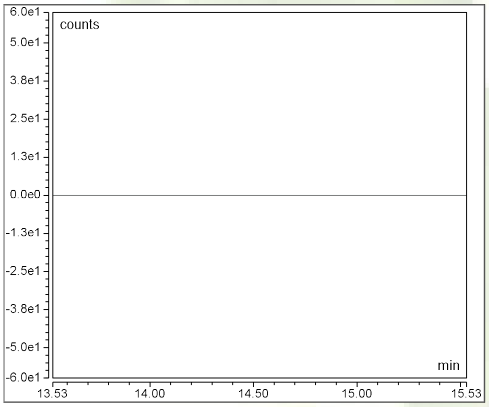

© 2009-2020 Thermo Fisher Scientific Inc. All rights reserved.
Chromeleon 7.3.0.60919

Component Name:	AAV8_VP3
Quantitation ions:	1574.800,1496.109,1534.442,1459.645,1273.445,1391.802
Retention time (min):	15.73
Expected Mass:	59804.680
Area (count*min):	1734030
Intensity (counts):	3342550
Amount:	-

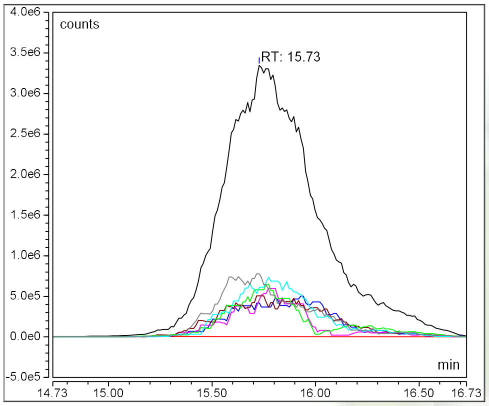

© 2009-2020 Thermo Fisher Scientific Inc. All rights reserved.
Chromeleon 7.3.0.60919

Component Name:	AAV9_VP3
Quantitation ions:	1358.581,1390.150,1299.556,1328.415,1271.930,1457.907
Retention time (min):	18.15
Expected Mass:	59732.620
Area (count*min):	-
Intensity (counts):	-
Amount:	-

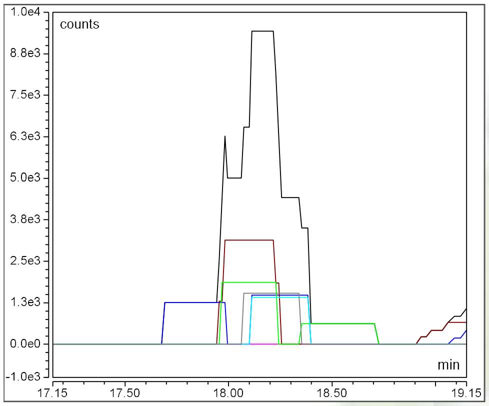

© 2009-2020 Thermo Fisher Scientific Inc. All rights reserved.
Chromeleon 7.3.0.60919

Component Name:	AAV2_VP3
Quantitation ions:	1428.955,1500.355,1463.789,1395.748,1364.055,1538.792
Retention time (min):	20.38
Expected Mass:	59973.780
Area (count*min):	-
Intensity (counts):	-
Amount:	-

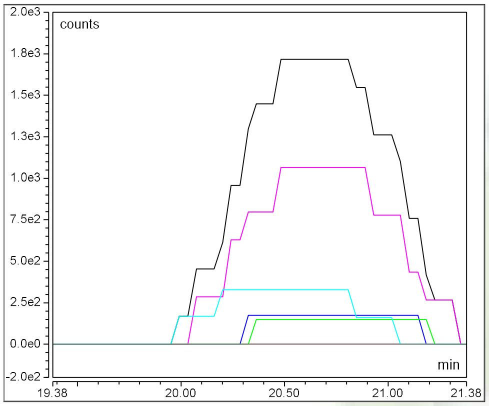

© 2009-2020 Thermo Fisher Scientific Inc. All rights reserved.
Chromeleon 7.3.0.60919

Component Name:	AAV9_VP1
Quantitation ions:	1378.820,1333.651,1355.868,1402.584,1534.808,1452.636
Retention time (min):	23.16
Expected Mass:	81290.340
Area (count*min):	-
Intensity (counts):	-
Amount:	-

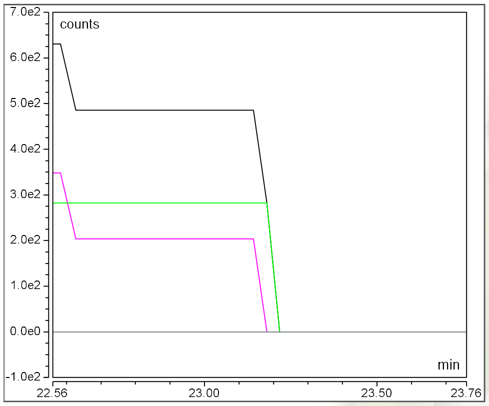

© 2009-2020 Thermo Fisher Scientific Inc. All rights reserved.
Chromeleon 7.3.0.60919

Component Name:	AAV8_VP1
Quantitation ions:	1409.080,1459.382,1385.209,1513.388,1184.605,1339.828
Retention time (min):	23.33
Expected Mass:	81666.710
Area (count*min):	21160
Intensity (counts):	47056
Amount:	-

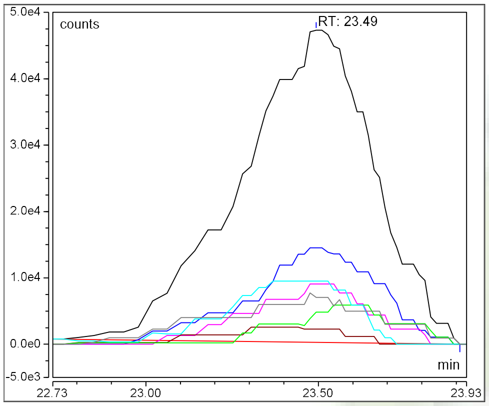

© 2009-2020 Thermo Fisher Scientific Inc. All rights reserved.
Chromeleon 7.3.0.60919

Component Name:	AAV5_VP1
Quantitation ions:	1362.628,1410.415,1435.577,1317.996,1339.949,1488.697
Retention time (min):	24.09
Expected Mass:	80335.080
Area (count*min):	-
Intensity (counts):	-
Amount:	-

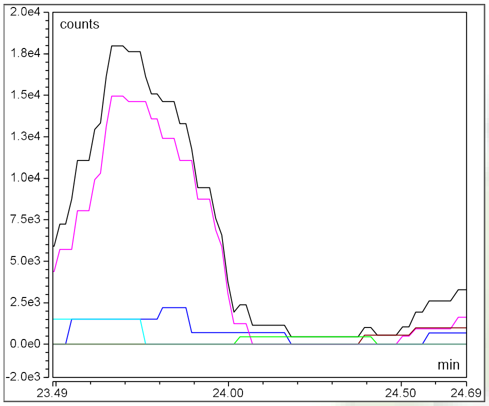

© 2009-2020 Thermo Fisher Scientific Inc. All rights reserved.
Chromeleon 7.3.0.60919

Component Name:	AAV9_VP2
Quantitation ions:	1409.730,1162.589,1472.334,1274.287,1380.371,1352.231
Retention time (min):	24.10
Expected Mass:	66209.760
Area (count*min):	-
Intensity (counts):	-
Amount:	-

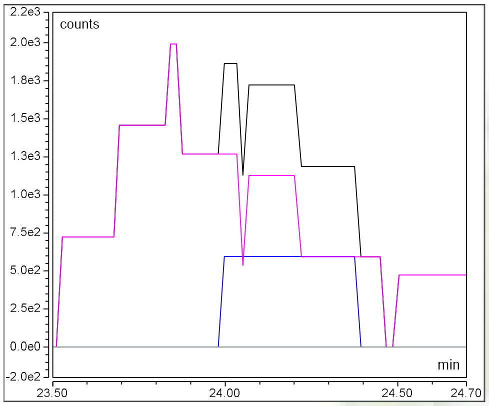

© 2009-2020 Thermo Fisher Scientific Inc. All rights reserved.
Chromeleon 7.3.0.60919

Component Name:	AAV8_VP2
Quantitation ions:	1386.809,1358.524,1331.393,1305.308,1416.294,1623.397
Retention time (min):	24.50
Expected Mass:	66518.100
Area (count*min):	14492
Intensity (counts):	28825
Amount:	-

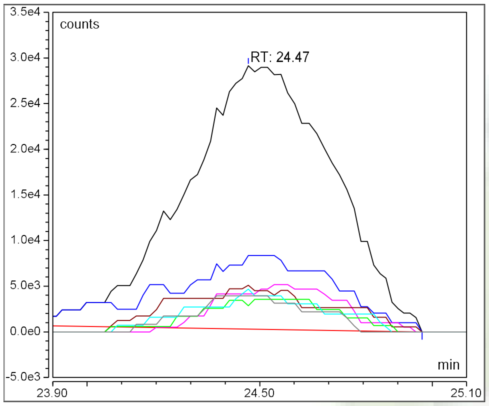

© 2009-2020 Thermo Fisher Scientific Inc. All rights reserved.
Chromeleon 7.3.0.60919

Component Name:	AAV5_VP2
Quantitation ions:	1389.997,1519.197,1484.692,1420.196,1555.348,1451.727
Retention time (min):	25.16
Expected Mass:	65282.690
Area (count*min):	-
Intensity (counts):	-
Amount:	-

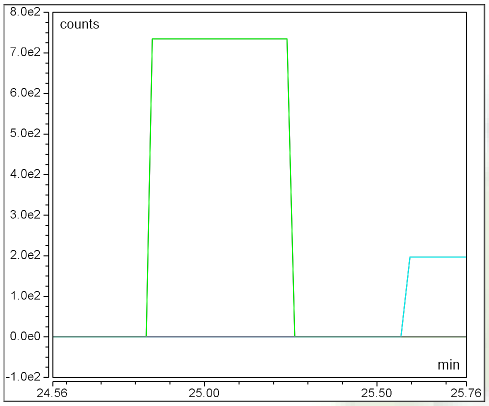

© 2009-2020 Thermo Fisher Scientific Inc. All rights reserved.
Chromeleon 7.3.0.60919

Component Name:	AAV2_VP1
Quantitation ions:	1388.434,1545.445,1489.336,1342.917,1365.283,1412.362
Retention time (min):	25.18
Expected Mass:	81854.900
Area (count*min):	-
Intensity (counts):	-
Amount:	-

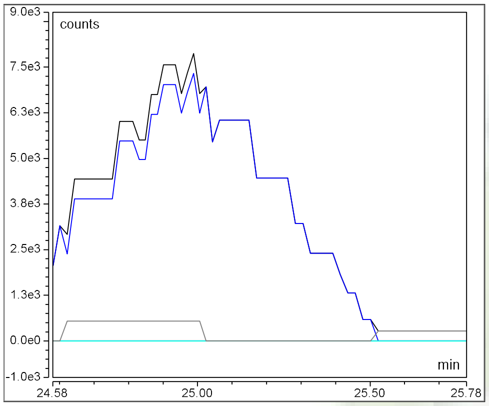

© 2009-2020 Thermo Fisher Scientific Inc. All rights reserved.
Chromeleon 7.3.0.60919

Component Name:	AAV2_VP2
Quantitation ions:	1415.636,1446.415,1255.530,1478.506,1279.609,1304.686
Retention time (min):	26.20
Expected Mass:	66487.900
Area (count*min):	-
Intensity (counts):	-
Amount:	-

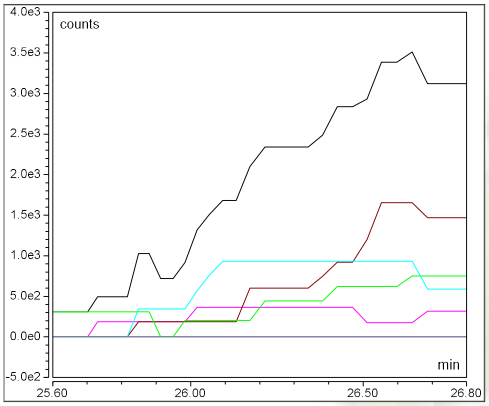

© 2009-2020 Thermo Fisher Scientific Inc. All rights reserved.
Chromeleon 7.3.0.60919

Chromatogram and SST Results

Injection Details

Injection Name:	**20211011_AAV8 E_45C_65-59B_Mod1_1ug_1**	*Run Time (min):*	**45.00**
Vial Number:	**R:A3**	*Injection Volume:*	**6.25**
Injection Type:	**Unknown**		
Calibration Level:			
Instrument Method:	**AAV Cap_Prot_Sep_BEH Glycoprotein Amide HILIC_1x150mm_FLR + MS_CT45C_65-59_S**		
Processing Method:	**MS Quantitative_45C_65-59B**	*Dilution Factor:*	**1.0000**
Injection Date/Time:	**13/Oct/21 01:42**	*Sample Weight:*	**1.0000**

Chromatogram

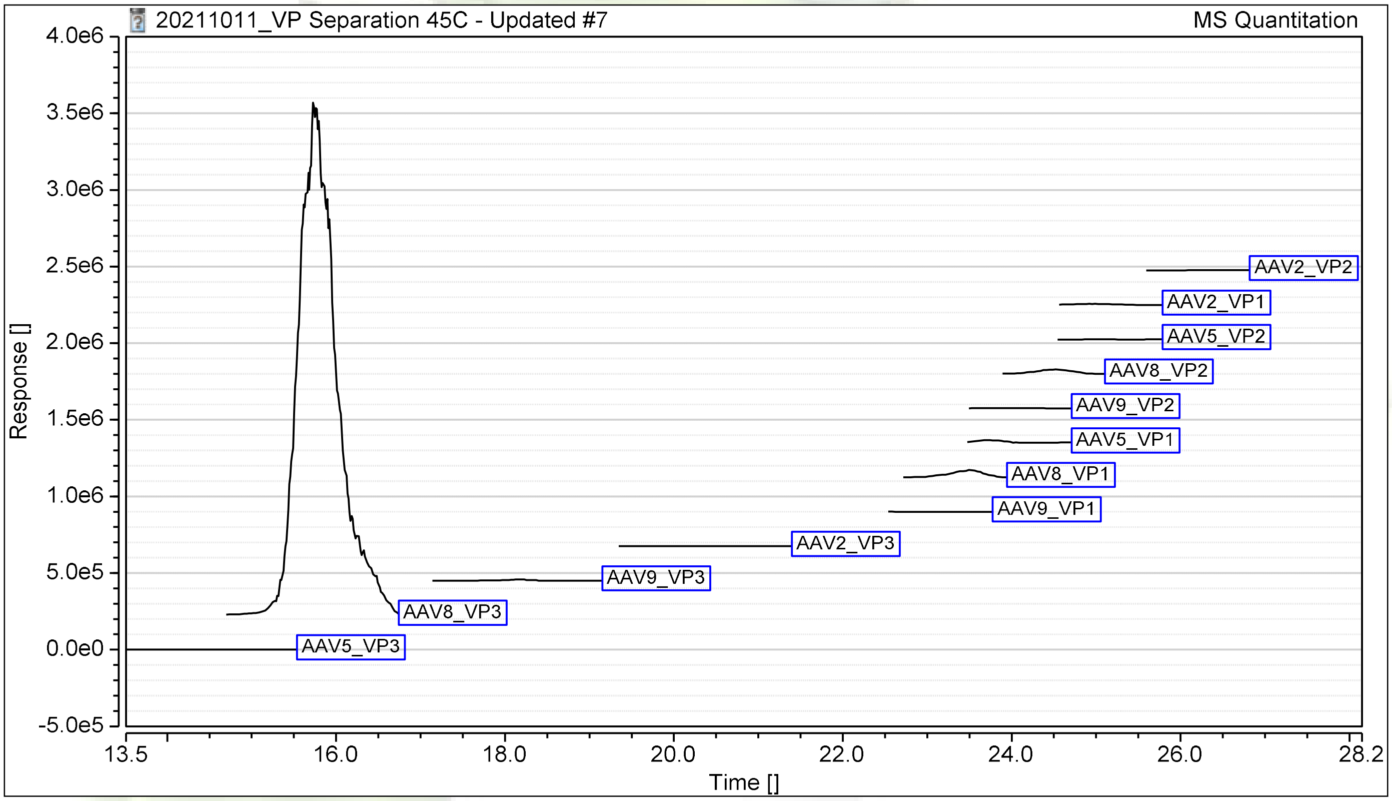

SST Results

No.	Name	Inj.Condition	Peak	Test Result	Injection
**Number of executed test cases:**	**n.a.**		**Total Result:**	**Passed**	

© 2009-2020 Thermo Fisher Scientific Inc. All rights reserved.

Protein Deconvolution Summary

Injection Details

Injection Name:	**20211011_AAV8 E_45C_65-59B_Mod1_1ug_2**	*Injection Volume:*	**6.25**
Vial Number:	**R:A3**	*Instrument Method:*	**AAV Cap_Prot_Sep_BEH Glycoprotein Amide H**
Injection Type:	**Unknown**	*Processing Method:*	**MS Quantitative_45C_65-59B**
Run Time (min):	**45.00**	*Injection Date/Time:*	**13/Oct/21 02:28**

Chromatogram

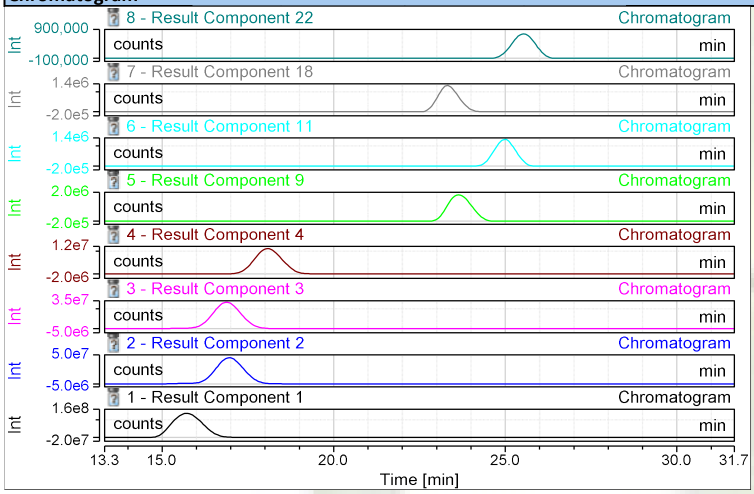

Source Spectrum

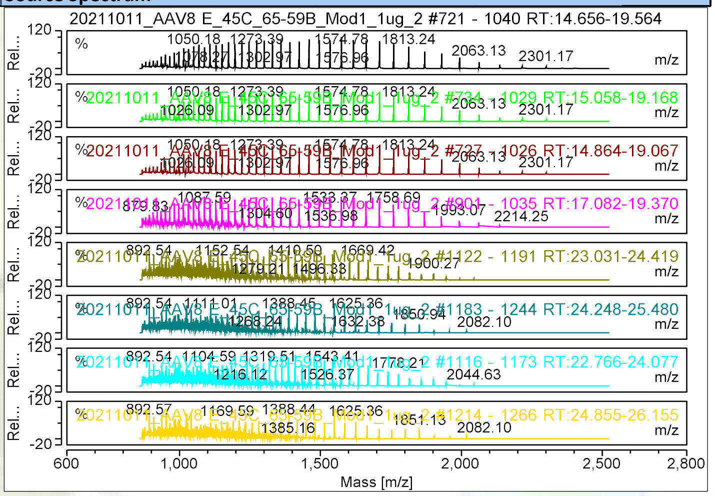

Deconvoluted Spectrum

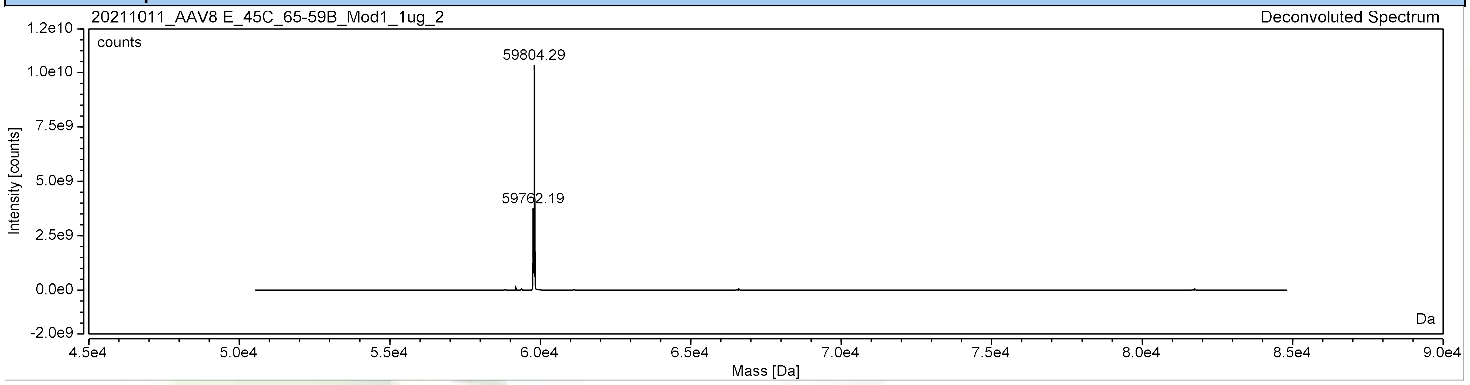

Deconvolution Results

Result Component	RT min	Mass Da	Std. Dev. Da	Std. Dev. ppm	Delta Mass Da	Intensity counts	Fractional Abundance counts	Rel. Abundance %	Number of Charge States	Score
Result Component 1	18.11	59733.033	0.40	6.68	0.000	27034264760	80.1	100.00	45	238.14
Result Component 2	18.22	59820.302	0.95	15.91	87.269	1527586438	4.5	5.65	40	170.79
Result Component 3	23.18	81291.415	0.91	11.13	21558.382	643910272	1.9	2.38	56	291.15
Result Component 4	19.20	59813.944	0.85	14.29	80.911	471832605	1.4	1.75	43	234.57
Result Component 5	18.27	59686.960	1.22	20.37	-46.073	390164875	1.2	1.44	32	176.14
Result Component 7	24.10	66210.268	0.85	12.85	6477.235	340089971	1.0	1.26	44	239.41
Result Component 8	21.47	59731.938	0.90	15.10	-1.095	300593658	0.9	1.11	41	217.18
Result Component 9	13.31	59733.070	0.64	10.75	0.037	298107673	0.9	1.10	42	257.50
Result Component 11	19.18	59933.627	1.10	18.41	200.594	200302659	0.6	0.74	40	199.85
Result Component 13	17.92	59687.388	1.55	25.99	-45.645	162773683	0.5	0.60	31	170.85
Result Component 15	21.21	59731.785	0.70	11.70	-1.249	129722977	0.4	0.48	41	225.58
Result Component 17	19.13	59766.513	1.08	18.11	33.479	91587642	0.3	0.34	38	197.64
Result Component 18	23.54	81372.238	1.92	23.54	21639.205	84227912	0.2	0.31	50	158.32
Result Component 19	18.85	59816.211	1.65	27.63	83.178	73795145	0.2	0.27	39	170.82
Result Component 20	19.45	59690.811	1.05	17.65	-42.222	72092544	0.2	0.27	37	195.30
Result Component 21	24.66	66290.558	1.28	19.26	6557.525	43412692	0.1	0.16	41	172.17

© 2009-2020 Thermo Fisher Scientific Inc. All rights reserved.
Chromeleon 7.3.0.60919

Component Summary

Injection Details

Injection Name:	**20211011_AAV8 E_45C_65-59B_Mod1_1ug**	*Injection Volume:*	**6.25**
Vial Number:	**R:A3**	*Instrument Method:*	**AAV Cap_Prot_Sep_BEH Glycoprotein Amide HILIC_1x150mm**
Injection Type:	**Unknown**	*Processing Method:*	**MS Quantitative_45C_65-59B**
Run Time (min):	**45.00**	*Injection Date/Time:*	**13/Oct/21 02:28**

Peak Name	Retention Time min	Area counts*min	Relative Area %	Quantitation Ions m/z	Mol.Mass	Mass Confirmation Pass/Fail
AAV8_VP3	15.698	1326273.881	71.23	1574.800,1496.109,1534.442,1459.645,1273.445,1391.802	59804.680	**PASS**
AAV8_VP1	23.490	13385.770	0.72	1409.080,1459.382,1385.209,1513.388,1184.605,1339.828	81666.710	**PASS**
AAV8_VP2	24.474	10871.521	0.58	1386.809,1358.524,1331.393,1305.308,1416.294,1623.397	66518.100	**PASS**
			**72.53**			

Component Name: AAV5_VP3

Quantitation ions:	1525.669,1565.784,1608.079,1451.304,1487.553,1416.767
Retention time (min):	14.53
Expected Mass:	59462.370
Area (count*min):	-
Intensity (counts):	-
Amount:	-

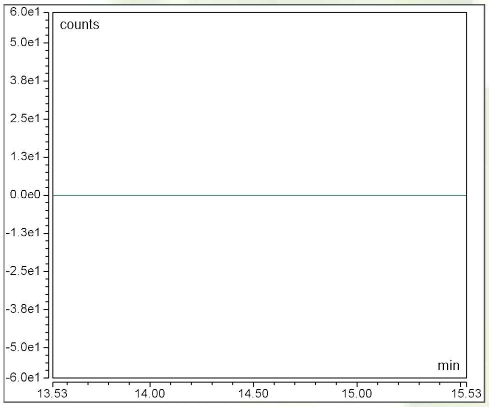

© 2009-2020 Thermo Fisher Scientific Inc. All rights reserved.
Chromeleon 7.3.0.60919

Component Name:	AAV8_VP3
Quantitation ions:	1574.800,1496.109,1534.442,1459.645,1273.445,1391.802
Retention time (min):	15.73
Expected Mass:	59804.680
Area (count*min):	1326274
Intensity (counts):	2388981
Amount:	-

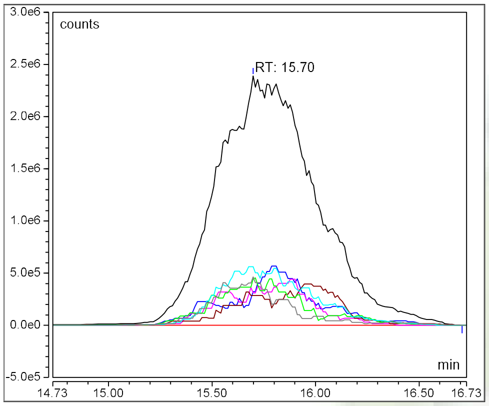

© 2009-2020 Thermo Fisher Scientific Inc. All rights reserved.
Chromeleon 7.3.0.60919

Component Name: AAV9_VP3

Quantitation ions:	1358.581,1390.150,1299.556,1328.415,1271.930,1457.907
Retention time (min):	18.15
Expected Mass:	59732.620
Area (count*min):	-
Intensity (counts):	-
Amount:	-

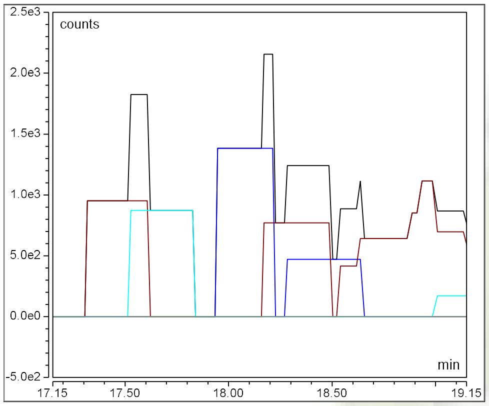

© 2009-2020 Thermo Fisher Scientific Inc. All rights reserved.
Chromeleon 7.3.0.60919

Component Name: AAV2_VP3

Quantitation ions:	1428.955,1500.355,1463.789,1395.748,1364.055,1538.792
Retention time (min):	20.38
Expected Mass:	59973.780
Area (count*min):	-
Intensity (counts):	-
Amount:	-

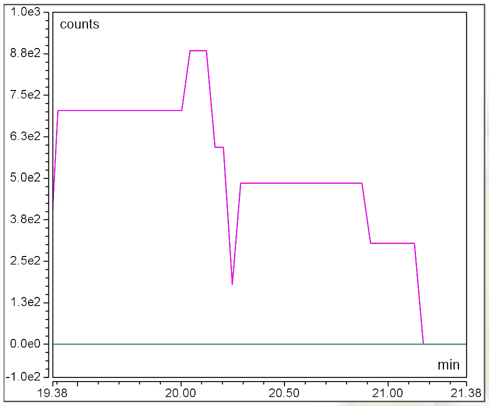

© 2009-2020 Thermo Fisher Scientific Inc. All rights reserved.
Chromeleon 7.3.0.60919

Component Name:	AAV9_VP1
Quantitation ions:	1378.820,1333.651,1355.868,1402.584,1534.808,1452.636
Retention time (min):	23.16
Expected Mass:	81290.340
Area (count*min):	-
Intensity (counts):	-
Amount:	-

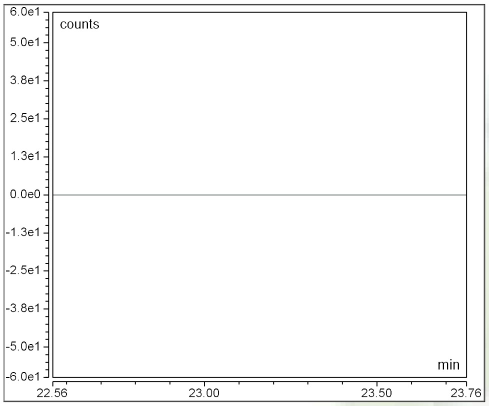

© 2009-2020 Thermo Fisher Scientific Inc. All rights reserved.
Chromeleon 7.3.0.60919

Component Name:	AAV8_VP1
Quantitation ions:	1409.080,1459.382,1385.209,1513.388,1184.605,1339.828
Retention time (min):	23.33
Expected Mass:	81666.710
Area (count*min):	13386
Intensity (counts):	26629
Amount:	-

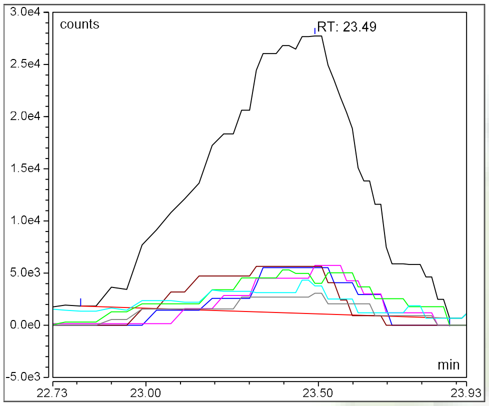

© 2009-2020 Thermo Fisher Scientific Inc. All rights reserved.
Chromeleon 7.3.0.60919

Component Name:	AAV5_VP1
Quantitation ions:	1362.628,1410.415,1435.577,1317.996,1339.949,1488.697
Retention time (min):	24.09
Expected Mass:	80335.080
Area (count*min):	-
Intensity (counts):	-
Amount:	-

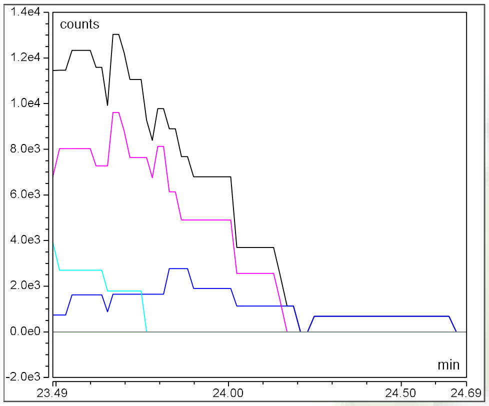

© 2009-2020 Thermo Fisher Scientific Inc. All rights reserved.
Chromeleon 7.3.0.60919

Component Name:	AAV9_VP2
Quantitation ions:	1409.730,1162.589,1472.334,1274.287,1380.371,1352.231
Retention time (min):	24.10
Expected Mass:	66209.760
Area (count*min):	-
Intensity (counts):	-
Amount:	-

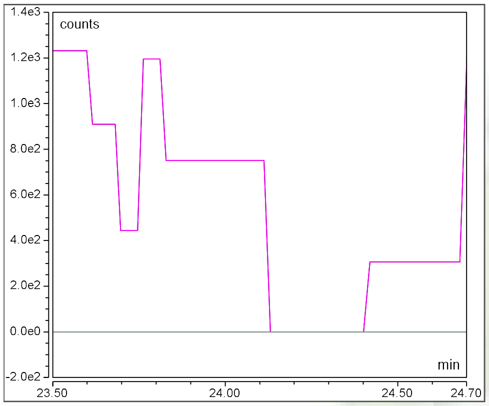

© 2009-2020 Thermo Fisher Scientific Inc. All rights reserved.
Chromeleon 7.3.0.60919

Component Name:	AAV8_VP2
Quantitation ions:	1386.809,1358.524,1331.393,1305.308,1416.294,1623.397
Retention time (min):	24.50
Expected Mass:	66518.100
Area (count*min):	10872
Intensity (counts):	20978
Amount:	-

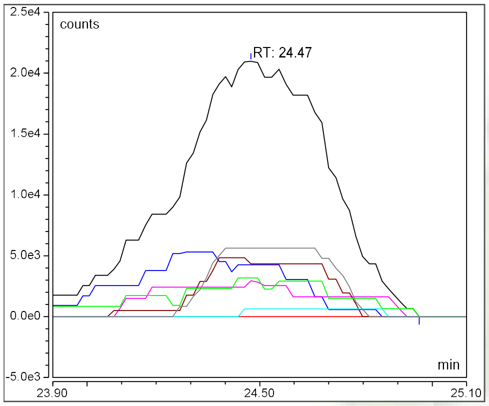

© 2009-2020 Thermo Fisher Scientific Inc. All rights reserved.
Chromeleon 7.3.0.60919

Component Name: AAV5_VP2

Quantitation ions:	1389.997,1519.197,1484.692,1420.196,1555.348,1451.727
Retention time (min):	25.16
Expected Mass:	65282.690
Area (count*min):	-
Intensity (counts):	-
Amount:	-

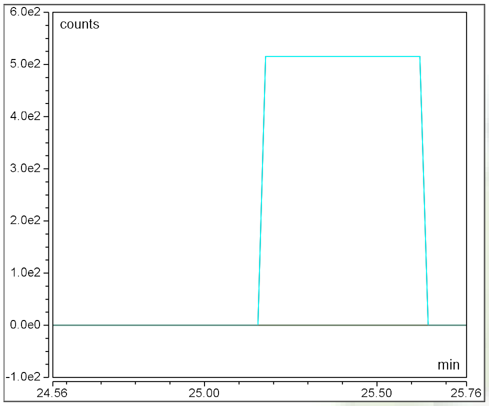

© 2009-2020 Thermo Fisher Scientific Inc. All rights reserved.
Chromeleon 7.3.0.60919

Component Name:	AAV2_VP1
Quantitation ions:	1388.434,1545.445,1489.336,1342.917,1365.283,1412.362
Retention time (min):	25.18
Expected Mass:	81854.900
Area (count*min):	-
Intensity (counts):	-
Amount:	-

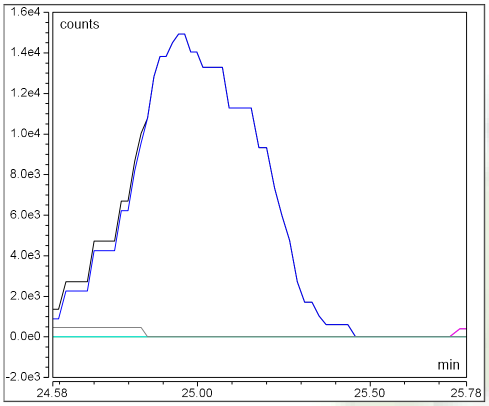

© 2009-2020 Thermo Fisher Scientific Inc. All rights reserved.
Chromeleon 7.3.0.60919

Component Name:	AAV2_VP2
Quantitation ions:	1415.636,1446.415,1255.530,1478.506,1279.609,1304.686
Retention time (min):	26.20
Expected Mass:	66487.900
Area (count*min):	-
Intensity (counts):	-
Amount:	-

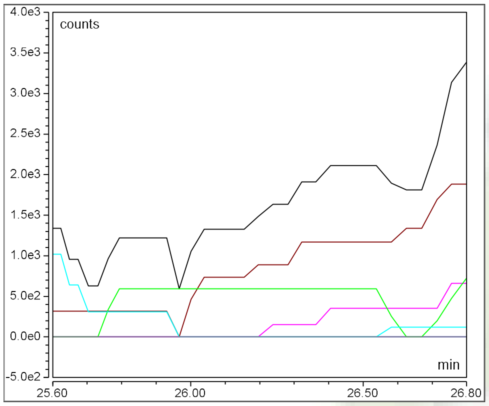

© 2009-2020 Thermo Fisher Scientific Inc. All rights reserved.
Chromeleon 7.3.0.60919

Chromatogram and SST Results

Injection Details

Injection Name:	**20211011_AAV8 E_45C_65-59B_Mod1_1ug_2**	*Run Time (min):*	**45.00**
Vial Number:	**R:A3**	*Injection Volume:*	**6.25**
Injection Type:	**Unknown**		
Calibration Level:			
Instrument Method:	**AAV Cap_Prot_Sep_BEH Glycoprotein Amide HILIC_1x150mm_FLR + MS_CT45C_65-59_S**		
Processing Method:	**MS Quantitative_45C_65-59B**	*Dilution Factor:*	**1.0000**
Injection Date/Time:	**13/Oct/21 02:28**	*Sample Weight:*	**1.0000**

Chromatogram

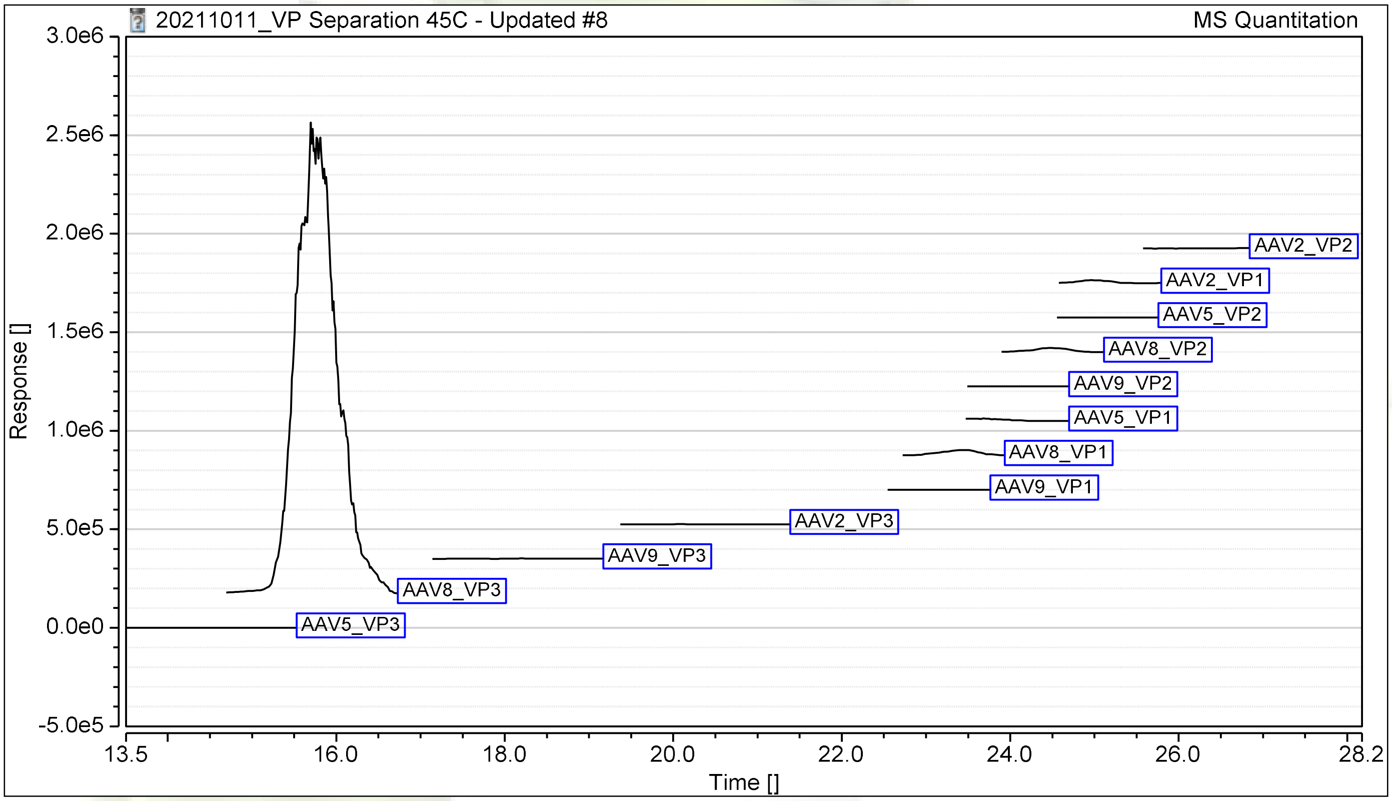

SST Results

No.	Name	Inj.Condition	Peak	Test Result	Injection
**Number of executed test cases:**	**n.a.**		**Total Result:**	**Passed**	

© 2009-2020 Thermo Fisher Scientific Inc. All rights reserved.

Protein Deconvolution Summary

Injection Details

Injection Name:	**20211011_AAV8 E_45C_65-59B_Mod1_1ug_3**	*Injection Volume:*	**6.25**
Vial Number:	**R:A3**	*Instrument Method:*	**AAV Cap_Prot_Sep_BEH Glycoprotein Amide H**
Injection Type:	**Unknown**	*Processing Method:*	**MS Quantitative_45C_65-59B**
Run Time (min):	**44.99**	*Injection Date/Time:*	**13/Oct/21 03:14**

Chromatogram

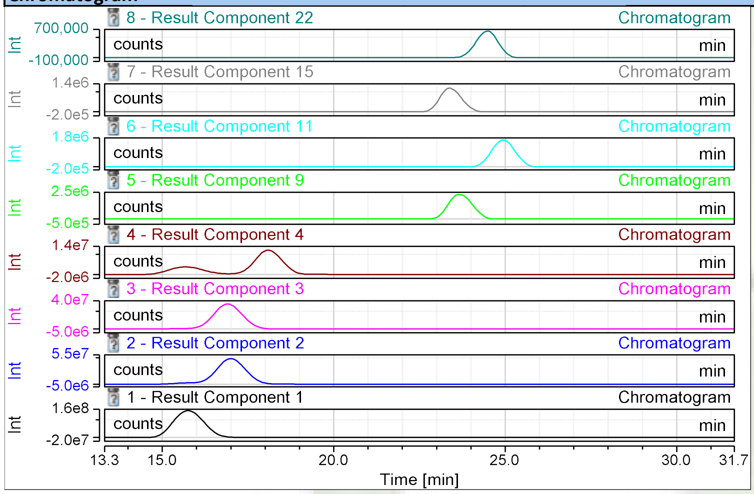

Source Spectrum

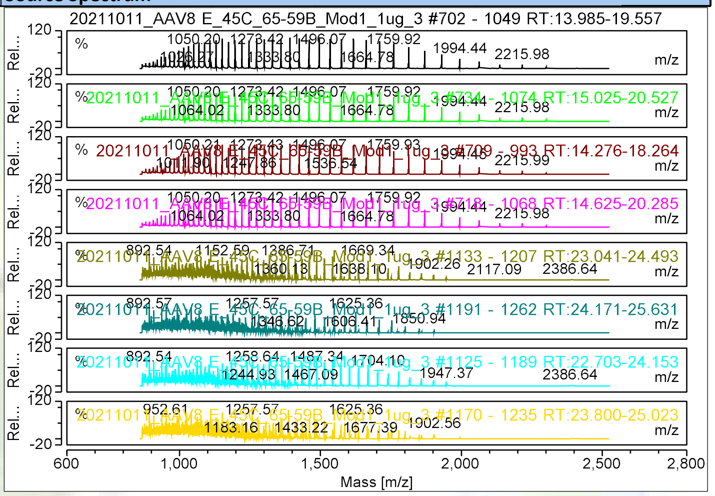

Deconvoluted Spectrum

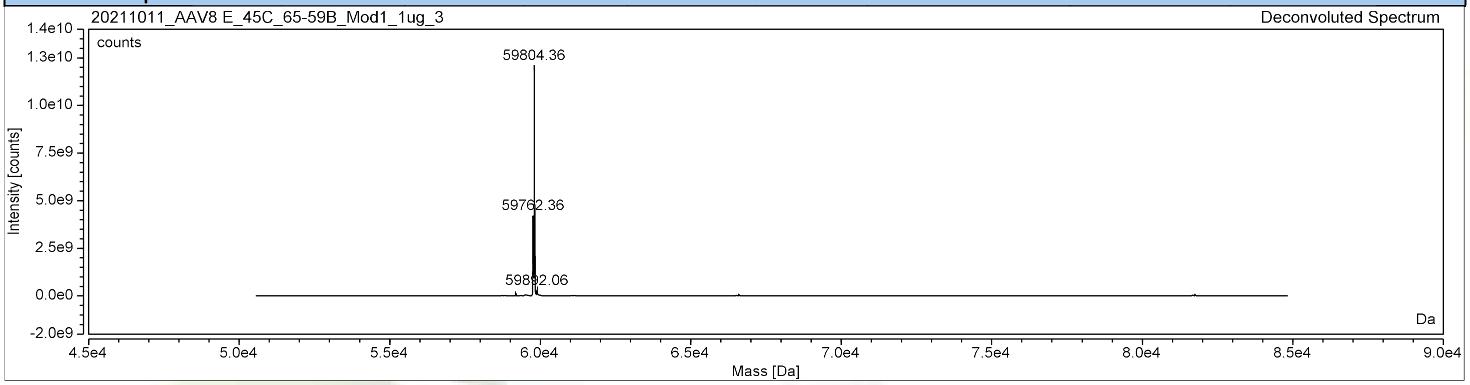

Deconvolution Results

Result Component	RT min	Mass Da	Std. Dev. Da	Std. Dev. ppm	Delta Mass Da	Intensity counts	Fractional Abundance counts	Rel. Abundance %	Number of Charge States	Score
Result Component 1	18.11	59733.033	0.40	6.68	0.000	27034264760	80.1	100.00	45	238.14
Result Component 2	18.22	59820.302	0.95	15.91	87.269	1527586438	4.5	5.65	40	170.79
Result Component 3	23.18	81291.415	0.91	11.13	21558.382	643910272	1.9	2.38	56	291.15
Result Component 4	19.20	59813.944	0.85	14.29	80.911	471832605	1.4	1.75	43	234.57
Result Component 5	18.27	59686.960	1.22	20.37	-46.073	390164875	1.2	1.44	32	176.14
Result Component 7	24.10	66210.268	0.85	12.85	6477.235	340089971	1.0	1.26	44	239.41
Result Component 8	21.47	59731.938	0.90	15.10	-1.095	300593658	0.9	1.11	41	217.18
Result Component 9	13.31	59733.070	0.64	10.75	0.037	298107673	0.9	1.10	42	257.50
Result Component 11	19.18	59933.627	1.10	18.41	200.594	200302659	0.6	0.74	40	199.85
Result Component 13	17.92	59687.388	1.55	25.99	-45.645	162773683	0.5	0.60	31	170.85
Result Component 15	21.21	59731.785	0.70	11.70	-1.249	129722977	0.4	0.48	41	225.58
Result Component 17	19.13	59766.513	1.08	18.11	33.479	91587642	0.3	0.34	38	197.64
Result Component 18	23.54	81372.238	1.92	23.54	21639.205	84227912	0.2	0.31	50	158.32
Result Component 19	18.85	59816.211	1.65	27.63	83.178	73795145	0.2	0.27	39	170.82
Result Component 20	19.45	59690.811	1.05	17.65	-42.222	72092544	0.2	0.27	37	195.30
Result Component 21	24.66	66290.558	1.28	19.26	6557.525	43412692	0.1	0.16	41	172.17

© 2009-2020 Thermo Fisher Scientific Inc. All rights reserved.
Chromeleon 7.3.0.60919

Component Summary

Injection Details			
Injection Name:	**20211011_AAV8 E_45C_65-59B_Mod1_1ug**	*Injection Volume:*	**6.25**
Vial Number:	**R:A3**	*Instrument Method:*	**AAV Cap_Prot_Sep_BEH Glycoprotein Amide HILIC_1x150mm**
Injection Type:	**Unknown**	*Processing Method:*	**MS Quantitative_45C_65-59B**
Run Time (min):	**44.99**	*Injection Date/Time:*	**13/Oct/21 03:14**

Peak Name	Retention Time min	Area counts*min	Relative Area %	Quantitation Ions m/z	Mol.Mass	Mass Confirmation Pass/Fail
AAV8_VP3	15.758	1818378.111	79.07	1574.800,1496.109,1534.442,1459.645,1273.445,1391.802	59804.680	**PASS**
AAV8_VP1	23.482	10174.093	0.44	1409.080,1459.382,1385.209,1513.388,1184.605,1339.828	81666.710	**PASS**
AAV8_VP2	24.344	8116.115	0.35	1386.809,1358.524,1331.393,1305.308,1416.294,1623.397	66518.100	**PASS**
			**79.86**			

Component Name: AAV5_VP3

Quantitation ions:	1525.669,1565.784,1608.079,1451.304,1487.553,1416.767
Retention time (min):	14.53
Expected Mass:	59462.370
Area (count*min):	-
Intensity (counts):	-
Amount:	-

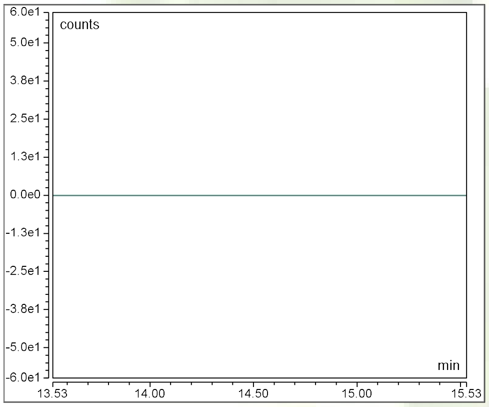

© 2009-2020 Thermo Fisher Scientific Inc. All rights reserved.
Chromeleon 7.3.0.60919

Component Name:	AAV8_VP3
Quantitation ions:	1574.800,1496.109,1534.442,1459.645,1273.445,1391.802
Retention time (min):	15.73
Expected Mass:	59804.680
Area (count*min):	1818378
Intensity (counts):	3555275
Amount:	-

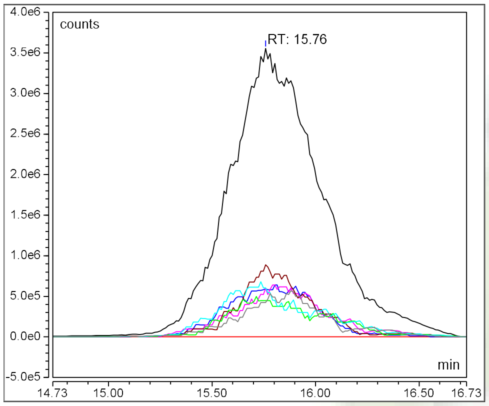

© 2009-2020 Thermo Fisher Scientific Inc. All rights reserved.
Chromeleon 7.3.0.60919

Component Name:	AAV9_VP3
Quantitation ions:	1358.581,1390.150,1299.556,1328.415,1271.930,1457.907
Retention time (min):	18.15
Expected Mass:	59732.620
Area (count*min):	-
Intensity (counts):	-
Amount:	-

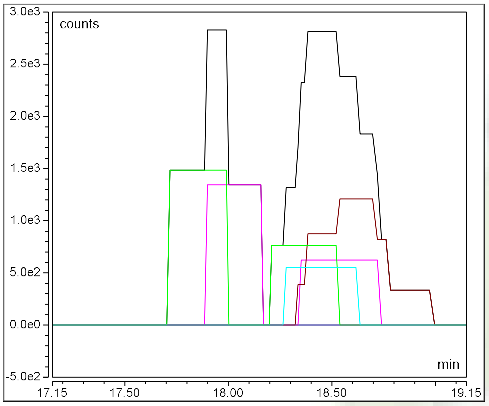

© 2009-2020 Thermo Fisher Scientific Inc. All rights reserved.
Chromeleon 7.3.0.60919

Component Name:	AAV2_VP3
Quantitation ions:	1428.955,1500.355,1463.789,1395.748,1364.055,1538.792
Retention time (min):	20.38
Expected Mass:	59973.780
Area (count*min):	-
Intensity (counts):	-
Amount:	-

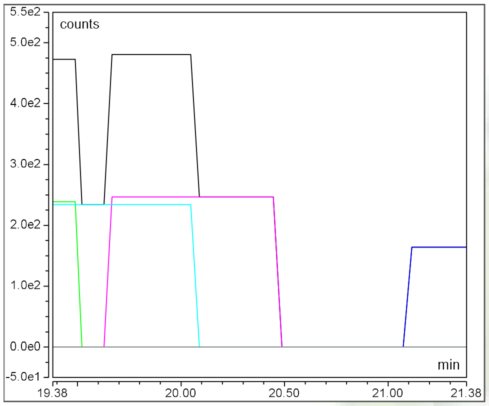

© 2009-2020 Thermo Fisher Scientific Inc. All rights reserved.
Chromeleon 7.3.0.60919

Component Name:	AAV9_VP1
Quantitation ions:	1378.820,1333.651,1355.868,1402.584,1534.808,1452.636
Retention time (min):	23.16
Expected Mass:	81290.340
Area (count*min):	-
Intensity (counts):	-
Amount:	-

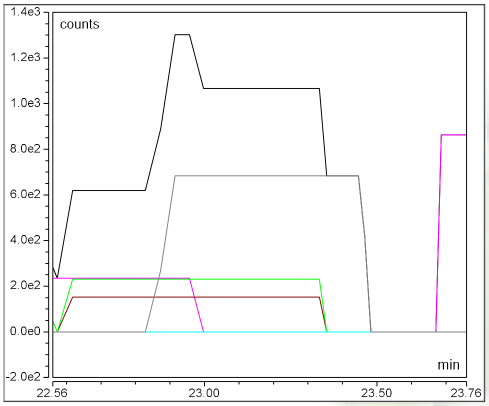

© 2009-2020 Thermo Fisher Scientific Inc. All rights reserved.
Chromeleon 7.3.0.60919

Component Name:	AAV8_VP1
Quantitation ions:	1409.080,1459.382,1385.209,1513.388,1184.605,1339.828
Retention time (min):	23.33
Expected Mass:	81666.710
Area (count*min):	10174
Intensity (counts):	23484
Amount:	-

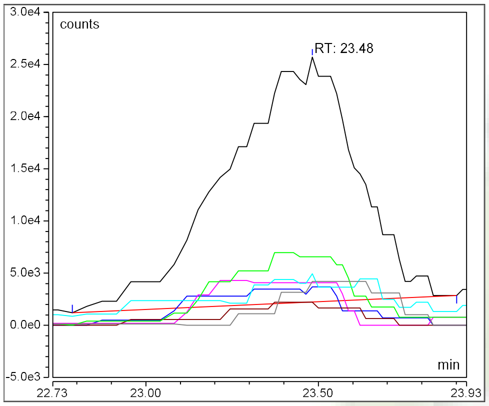

© 2009-2020 Thermo Fisher Scientific Inc. All rights reserved.
Chromeleon 7.3.0.60919

Component Name: AAV5_VP1

Quantitation ions:	1362.628,1410.415,1435.577,1317.996,1339.949,1488.697
Retention time (min):	24.09
Expected Mass:	80335.080
Area (count*min):	-
Intensity (counts):	-
Amount:	-

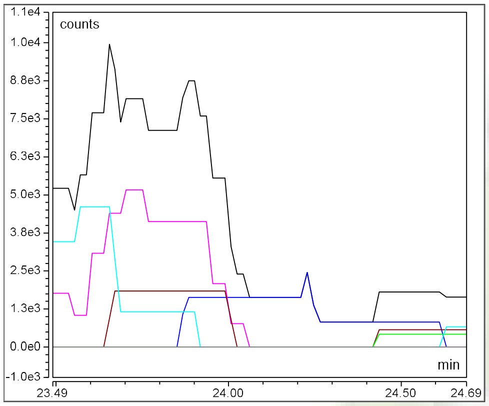

© 2009-2020 Thermo Fisher Scientific Inc. All rights reserved.
Chromeleon 7.3.0.60919

Component Name:	AAV9_VP2
Quantitation ions:	1409.730,1162.589,1472.334,1274.287,1380.371,1352.231
Retention time (min):	24.10
Expected Mass:	66209.760
Area (count*min):	-
Intensity (counts):	-
Amount:	-

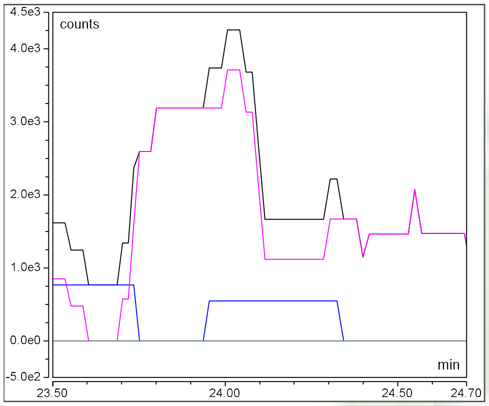

© 2009-2020 Thermo Fisher Scientific Inc. All rights reserved.
Chromeleon 7.3.0.60919

Component Name: AAV8_VP2

Quantitation ions:	1386.809,1358.524,1331.393,1305.308,1416.294,1623.397
Retention time (min):	24.50
Expected Mass:	66518.100
Area (count*min):	8116
Intensity (counts):	14575
Amount:	-

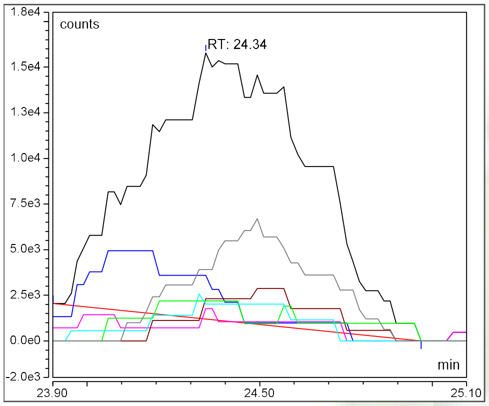

© 2009-2020 Thermo Fisher Scientific Inc. All rights reserved.
Chromeleon 7.3.0.60919

Component Name:	AAV5_VP2
Quantitation ions:	1389.997,1519.197,1484.692,1420.196,1555.348,1451.727
Retention time (min):	25.16
Expected Mass:	65282.690
Area (count*min):	-
Intensity (counts):	-
Amount:	-

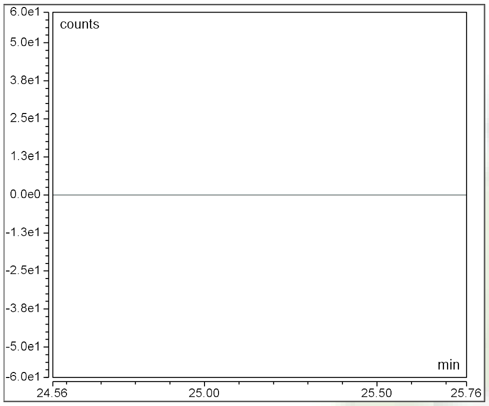

© 2009-2020 Thermo Fisher Scientific Inc. All rights reserved.
Chromeleon 7.3.0.60919

**Component Name:**	**AAV2_VP1**
Quantitation ions:	1388.434,1545.445,1489.336,1342.917,1365.283,1412.362
Retention time (min):	25.18
Expected Mass:	81854.900
Area (count*min):	-
Intensity (counts):	-
Amount:	-

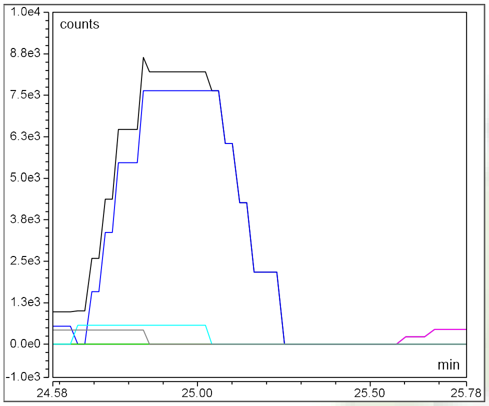

© 2009-2020 Thermo Fisher Scientific Inc. All rights reserved.
Chromeleon 7.3.0.60919

Component Name:	AAV2_VP2
Quantitation ions:	1415.636,1446.415,1255.530,1478.506,1279.609,1304.686
Retention time (min):	26.20
Expected Mass:	66487.900
Area (count*min):	-
Intensity (counts):	-
Amount:	-

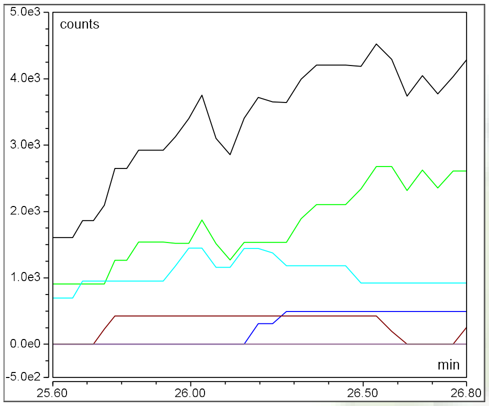

© 2009-2020 Thermo Fisher Scientific Inc. All rights reserved.
Chromeleon 7.3.0.60919

Chromatogram and SST Results

Injection Details

Injection Name:	**20211011_AAV8 E_45C_65-59B_Mod1_1ug_3**	*Run Time (min):*	**44.99**
Vial Number:	**R:A3**	*Injection Volume:*	**6.25**
Injection Type:	**Unknown**		
Calibration Level:			
Instrument Method:	**AAV Cap_Prot_Sep_BEH Glycoprotein Amide HILIC_1x150mm_FLR + MS_CT45C_65-59_S**		
Processing Method:	**MS Quantitative_45C_65-59B**	*Dilution Factor:*	**1.0000**
Injection Date/Time:	**13/Oct/21 03:14**	*Sample Weight:*	**1.0000**

Chromatogram

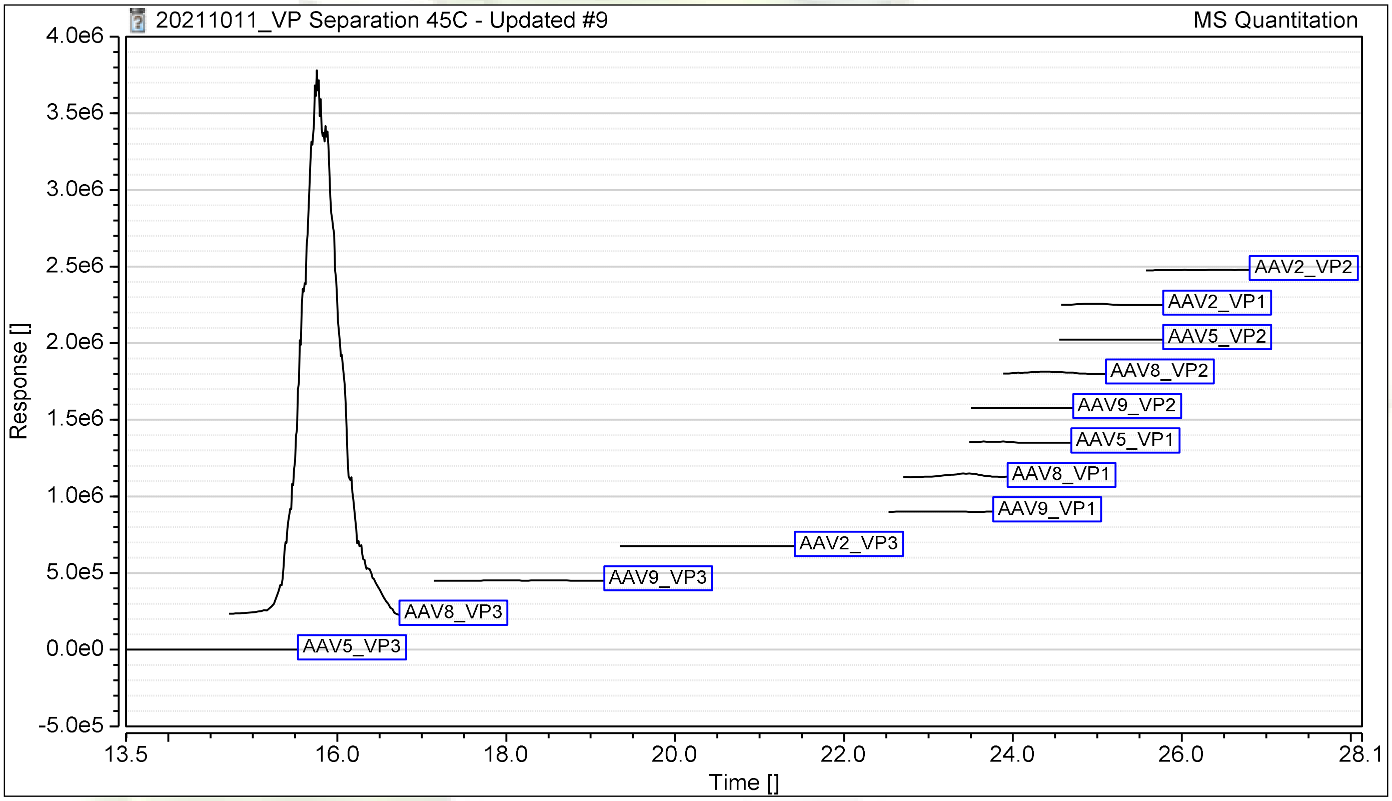

SST Results

No.	Name	Inj.Condition	Peak	Test Result	Injection
**Number of executed test cases:**	**n.a.**		**Total Result:**	**Passed**	

© 2009-2020 Thermo Fisher Scientific Inc. All rights reserved.

Protein Deconvolution Summary

Injection Details

Injection Name:	**20211011_AAV5 E_45C_65-59B_Mod1_1ug_1**	*Injection Volume:*	**3.17**
Vial Number:	**R:A7**	*Instrument Method:*	**AAV Cap_Prot_Sep_BEH Glycoprotein Amide H**
Injection Type:	**Unknown**	*Processing Method:*	**MS Quantitative_45C_65-59B**
Run Time (min):	**44.99**	*Injection Date/Time:*	**13/Oct/21 05:26**

Chromatogram

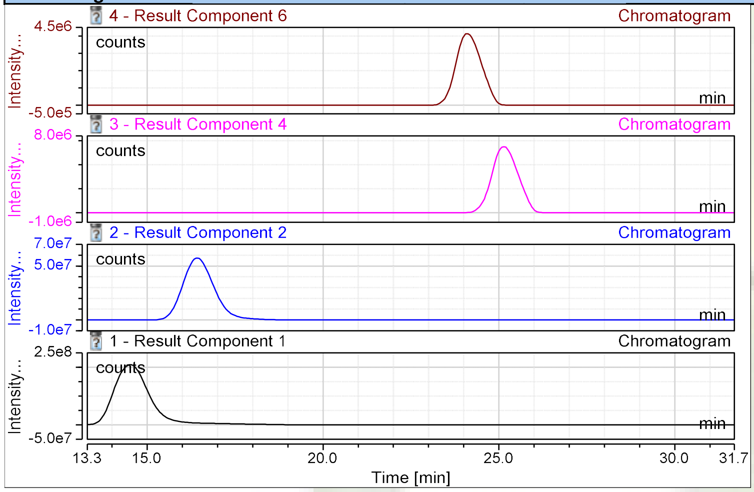

Source Spectrum

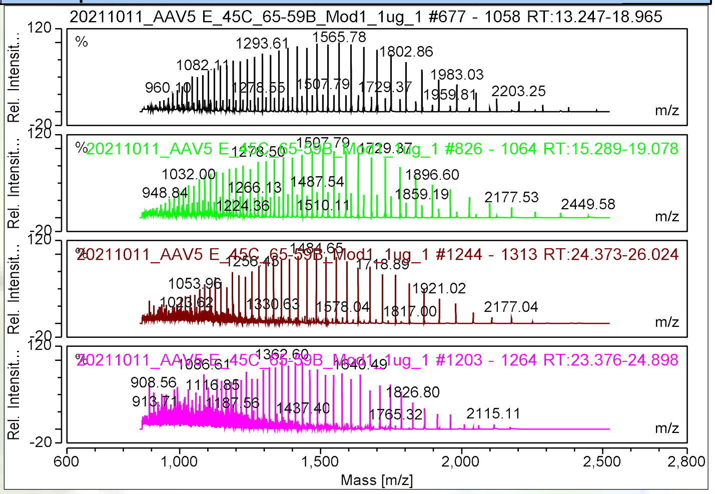

Deconvoluted Spectrum

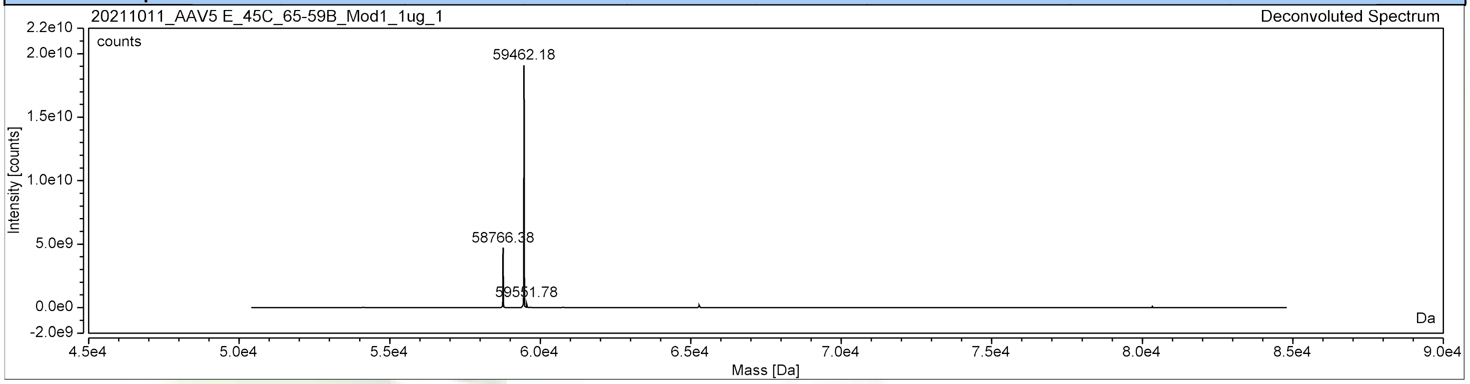

Deconvolution Results

Result Component	RT min	Mass Da	Std. Dev. Da	Std. Dev. ppm	Delta Mass Da	Intensity counts	Fractional Abundance counts	Rel. Abundance %	Number of Charge States	Score
Result Component 1	18.11	59733.033	0.40	6.68	0.000	27034264760	80.1	100.00	45	238.14
Result Component 2	18.22	59820.302	0.95	15.91	87.269	1527586438	4.5	5.65	40	170.79
Result Component 3	23.18	81291.415	0.91	11.13	21558.382	643910272	1.9	2.38	56	291.15
Result Component 4	19.20	59813.944	0.85	14.29	80.911	471832605	1.4	1.75	43	234.57
Result Component 5	18.27	59686.960	1.22	20.37	-46.073	390164875	1.2	1.44	32	176.14
Result Component 7	24.10	66210.268	0.85	12.85	6477.235	340089971	1.0	1.26	44	239.41
Result Component 8	21.47	59731.938	0.90	15.10	-1.095	300593658	0.9	1.11	41	217.18
Result Component 9	13.31	59733.070	0.64	10.75	0.037	298107673	0.9	1.10	42	257.50
Result Component 11	19.18	59933.627	1.10	18.41	200.594	200302659	0.6	0.74	40	199.85
Result Component 13	17.92	59687.388	1.55	25.99	-45.645	162773683	0.5	0.60	31	170.85
Result Component 15	21.21	59731.785	0.70	11.70	-1.249	129722977	0.4	0.48	41	225.58
Result Component 17	19.13	59766.513	1.08	18.11	33.479	91587642	0.3	0.34	38	197.64
Result Component 18	23.54	81372.238	1.92	23.54	21639.205	84227912	0.2	0.31	50	158.32
Result Component 19	18.85	59816.211	1.65	27.63	83.178	73795145	0.2	0.27	39	170.82
Result Component 20	19.45	59690.811	1.05	17.65	-42.222	72092544	0.2	0.27	37	195.30
Result Component 21	24.66	66290.558	1.28	19.26	6557.525	43412692	0.1	0.16	41	172.17

© 2009-2020 Thermo Fisher Scientific Inc. All rights reserved.
Chromeleon 7.3.0.60919

Component Summary

Injection Details

Injection Name:	**20211011_AAV5 E_45C_65-59B_Mod1_1ug**	*Injection Volume:*	**3.17**
Vial Number:	**R:A7**	*Instrument Method:*	**AAV Cap_Prot_Sep_BEH Glycoprotein Amide HILIC_1x150mm**
Injection Type:	**Unknown**	*Processing Method:*	**MS Quantitative_45C_65-59B**
Run Time (min):	**44.99**	*Injection Date/Time:*	**13/Oct/21 05:26**

Peak Name	Retention Time min	Area counts*min	Relative Area %	Quantitation Ions m/z	Mol.Mass	Mass Confirmation Pass/Fail
AAV5_VP3	14.592	2892946.404	89.95	1525.669,1565.784,1608.079,1451.304,1487.553,1416.767	59462.370	**PASS**
AAV5_VP1	24.157	33639.319	1.05	1362.628,1410.415,1435.577,1317.996,1339.949,1488.697	80335.080	**PASS**
AAV5_VP2	25.077	84281.750	2.62	1389.997,1519.197,1484.692,1420.196,1555.348,1451.727	65282.690	**PASS**
			**93.62**			

Component Name: AAV5_VP3

Quantitation ions:	1525.669,1565.784,1608.079,1451.304,1487.553,1416.767
Retention time (min):	14.53
Expected Mass:	59462.370
Area (count*min):	2892946
Intensity (counts):	4794382
Amount:	-

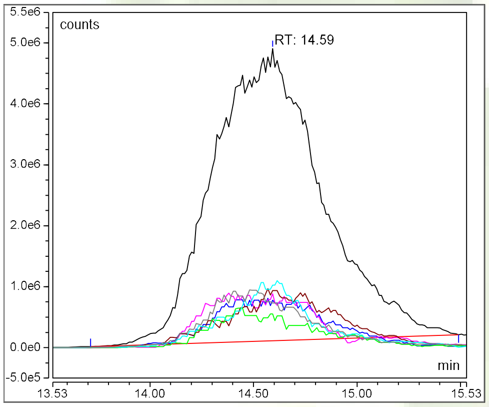

© 2009-2020 Thermo Fisher Scientific Inc. All rights reserved.
Chromeleon 7.3.0.60919

Component Name:	AAV8_VP3
Quantitation ions:	1574.800,1496.109,1534.442,1459.645,1273.445,1391.802
Retention time (min):	15.73
Expected Mass:	59804.680
Area (count*min):	-
Intensity (counts):	-
Amount:	-

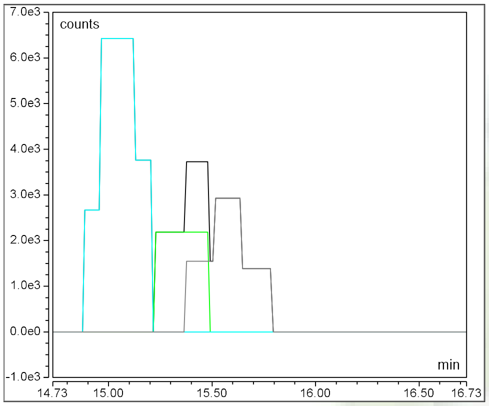

© 2009-2020 Thermo Fisher Scientific Inc. All rights reserved.
Chromeleon 7.3.0.60919

Component Name:	AAV9_VP3
Quantitation ions:	1358.581,1390.150,1299.556,1328.415,1271.930,1457.907
Retention time (min):	18.15
Expected Mass:	59732.620
Area (count*min):	-
Intensity (counts):	-
Amount:	-

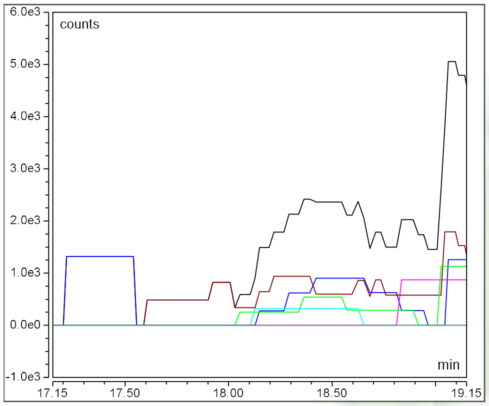

© 2009-2020 Thermo Fisher Scientific Inc. All rights reserved.
Chromeleon 7.3.0.60919

Component Name: AAV2_VP3

Quantitation ions:	1428.955,1500.355,1463.789,1395.748,1364.055,1538.792
Retention time (min):	20.38
Expected Mass:	59973.780
Area (count*min):	-
Intensity (counts):	-
Amount:	-

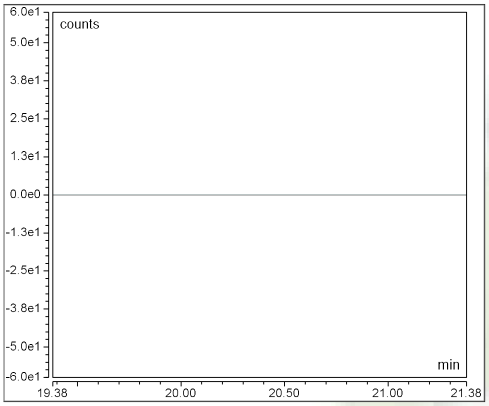

© 2009-2020 Thermo Fisher Scientific Inc. All rights reserved.
Chromeleon 7.3.0.60919

Component Name:	AAV9_VP1
Quantitation ions:	1378.820,1333.651,1355.868,1402.584,1534.808,1452.636
Retention time (min):	23.16
Expected Mass:	81290.340
Area (count*min):	-
Intensity (counts):	-
Amount:	-

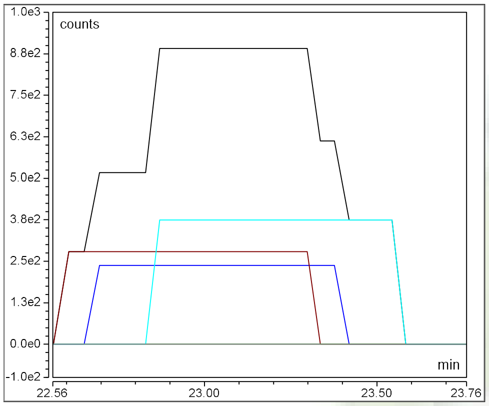

© 2009-2020 Thermo Fisher Scientific Inc. All rights reserved.
Chromeleon 7.3.0.60919

Component Name:	AAV8_VP1
Quantitation ions:	1409.080,1459.382,1385.209,1513.388,1184.605,1339.828
Retention time (min):	23.33
Expected Mass:	81666.710
Area (count*min):	-
Intensity (counts):	-
Amount:	-

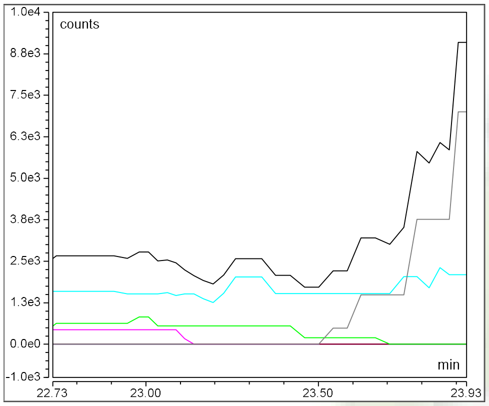

© 2009-2020 Thermo Fisher Scientific Inc. All rights reserved.
Chromeleon 7.3.0.60919

Component Name:	AAV5_VP1
Quantitation ions:	1362.628,1410.415,1435.577,1317.996,1339.949,1488.697
Retention time (min):	24.09
Expected Mass:	80335.080
Area (count*min):	33639
Intensity (counts):	71494
Amount:	-

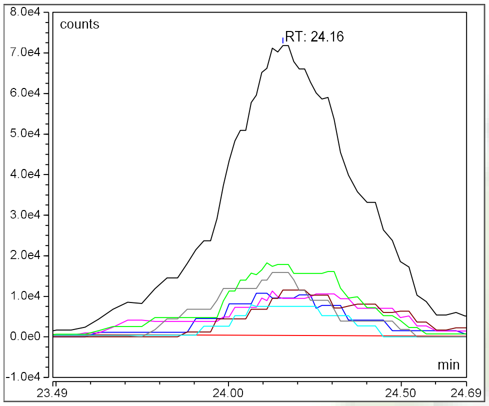

© 2009-2020 Thermo Fisher Scientific Inc. All rights reserved.
Chromeleon 7.3.0.60919

Component Name:	AAV9_VP2
Quantitation ions:	1409.730,1162.589,1472.334,1274.287,1380.371,1352.231
Retention time (min):	24.10
Expected Mass:	66209.760
Area (count*min):	-
Intensity (counts):	-
Amount:	-

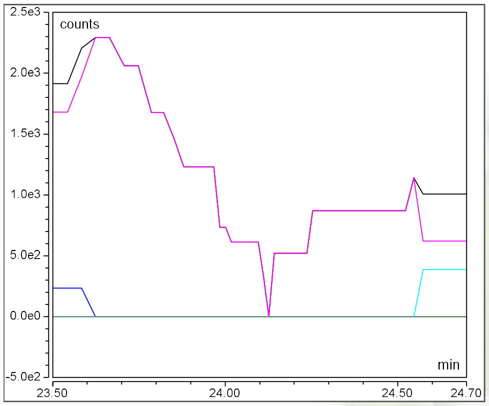

© 2009-2020 Thermo Fisher Scientific Inc. All rights reserved.
Chromeleon 7.3.0.60919

Component Name:	AAV8_VP2
Quantitation ions:	1386.809,1358.524,1331.393,1305.308,1416.294,1623.397
Retention time (min):	24.50
Expected Mass:	66518.100
Area (count*min):	-
Intensity (counts):	-
Amount:	-

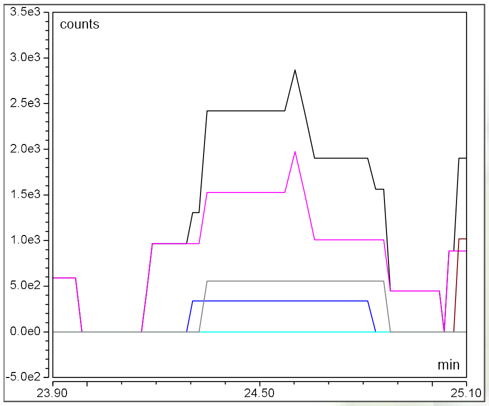

© 2009-2020 Thermo Fisher Scientific Inc. All rights reserved.
Chromeleon 7.3.0.60919

Component Name: AAV5_VP2

Quantitation ions:	1389.997,1519.197,1484.692,1420.196,1555.348,1451.727
Retention time (min):	25.16
Expected Mass:	65282.690
Area (count*min):	84282
Intensity (counts):	189087
Amount:	-

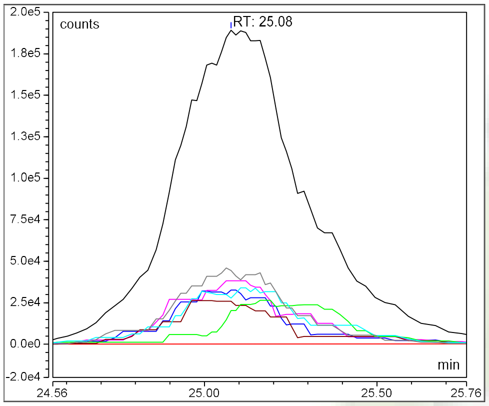

© 2009-2020 Thermo Fisher Scientific Inc. All rights reserved.
Chromeleon 7.3.0.60919

Component Name:	AAV2_VP1
Quantitation ions:	1388.434,1545.445,1489.336,1342.917,1365.283,1412.362
Retention time (min):	25.18
Expected Mass:	81854.900
Area (count*min):	-
Intensity (counts):	-
Amount:	-

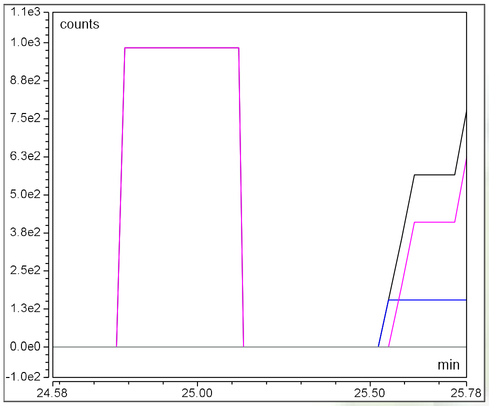

© 2009-2020 Thermo Fisher Scientific Inc. All rights reserved.
Chromeleon 7.3.0.60919

Component Name: AAV2_VP2	
Quantitation ions:	1415.636,1446.415,1255.530,1478.506,1279.609,1304.686
Retention time (min):	26.20
Expected Mass:	66487.900
Area (count*min):	-
Intensity (counts):	-
Amount:	-

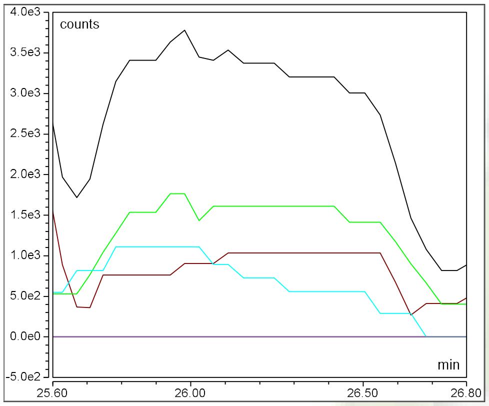

© 2009-2020 Thermo Fisher Scientific Inc. All rights reserved.
Chromeleon 7.3.0.60919

Chromatogram and SST Results

Injection Details

Injection Name:	**20211011_AAV5 E_45C_65-59B_Mod1_1ug_1**	*Run Time (min):*	**44.99**
Vial Number:	**R:A7**	*Injection Volume:*	**3.17**
Injection Type:	**Unknown**		
Calibration Level:			
Instrument Method:	**AAV Cap_Prot_Sep_BEH Glycoprotein Amide HILIC_1x150mm_FLR + MS_CT45C_65-59_S**		
Processing Method:	**MS Quantitative_45C_65-59B**	*Dilution Factor:*	**1.0000**
Injection Date/Time:	**13/Oct/21 05:26**	*Sample Weight:*	**1.0000**

Chromatogram

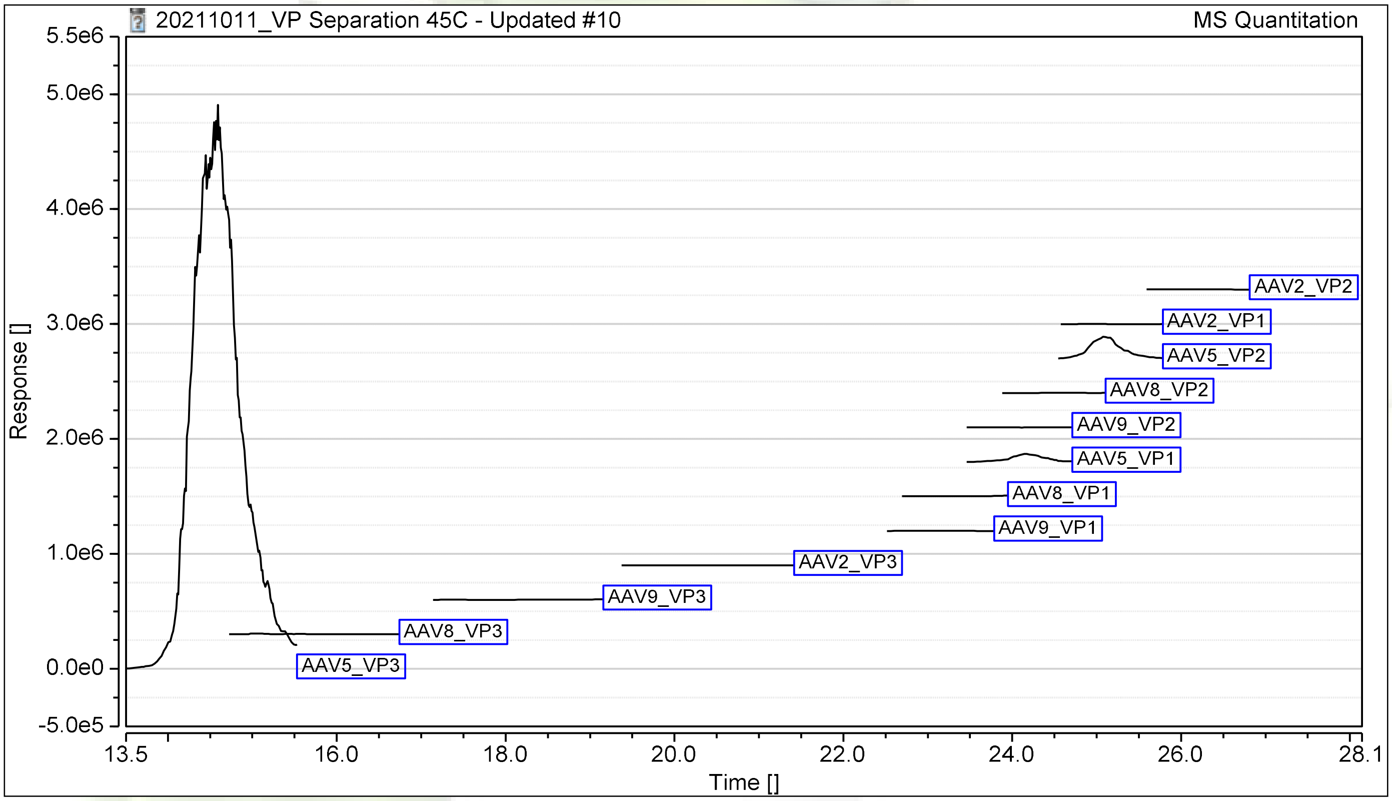

SST Results

No.	Name	Inj.Condition	Peak	Test Result	Injection

Number of executed test cases: **n.a.** **Total Result:** **Passed**

© 2009-2020 Thermo Fisher Scientific Inc. All rights reserved.

Protein Deconvolution Summary

Injection Details

Injection Name:	**20211011_AAV5 E_45C_65-59B_Mod1_1ug_2**	*Injection Volume:*	**3.17**
Vial Number:	**R:A7**	*Instrument Method:*	**AAV Cap_Prot_Sep_BEH Glycoprotein Amide H**
Injection Type:	**Unknown**	*Processing Method:*	**MS Quantitative_45C_65-59B**
Run Time (min):	**44.98**	*Injection Date/Time:*	**13/Oct/21 06:12**

Chromatogram

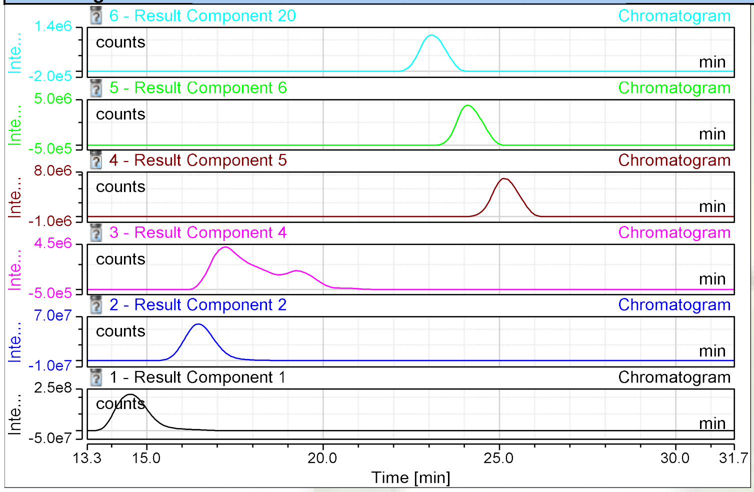

Source Spectrum

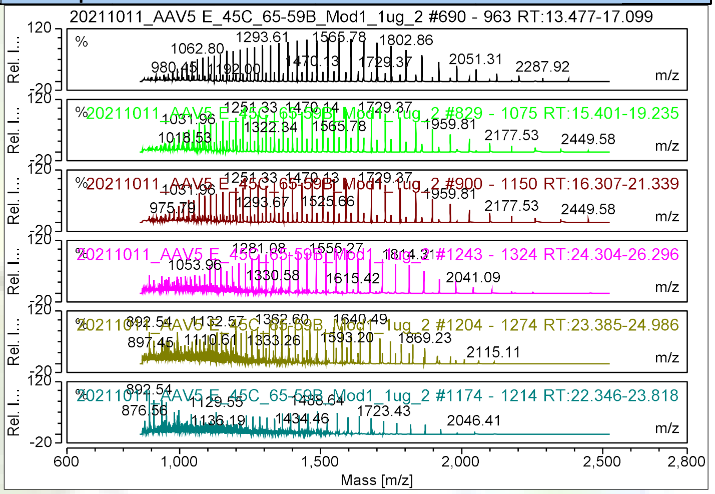

Deconvoluted Spectrum

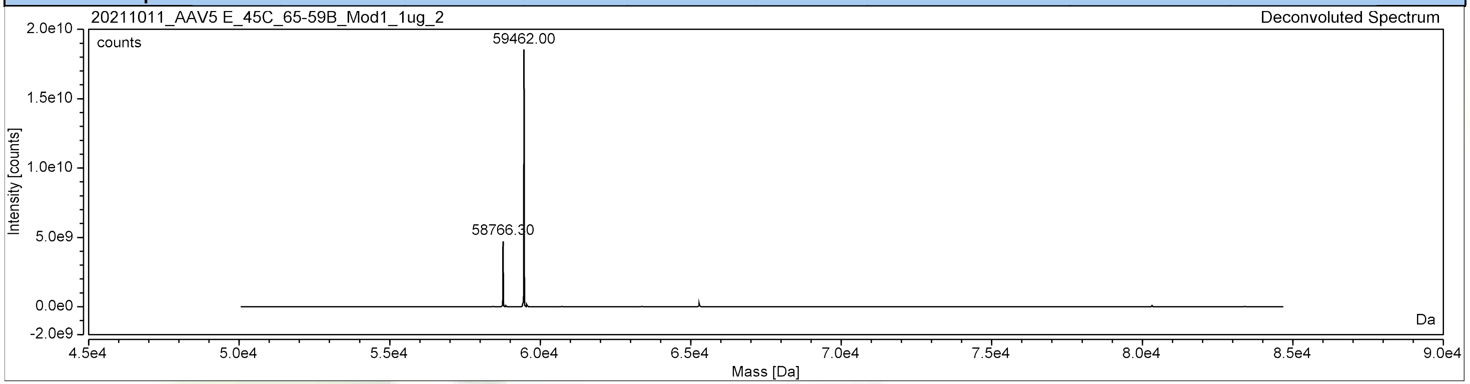

Deconvolution Results

Result Component	RT min	Mass Da	Std. Dev. Da	Std. Dev. ppm	Delta Mass Da	Intensity counts	Fractional Abundance counts	Rel. Abundance %	Number of Charge States	Score
Result Component 1	18.11	59733.033	0.40	6.68	0.000	27034264760	80.1	100.00	45	238.14
Result Component 2	18.22	59820.302	0.95	15.91	87.269	1527586438	4.5	5.65	40	170.79
Result Component 3	23.18	81291.415	0.91	11.13	21558.382	643910272	1.9	2.38	56	291.15
Result Component 4	19.20	59813.944	0.85	14.29	80.911	471832605	1.4	1.75	43	234.57
Result Component 5	18.27	59686.960	1.22	20.37	-46.073	390164875	1.2	1.44	32	176.14
Result Component 7	24.10	66210.268	0.85	12.85	6477.235	340089971	1.0	1.26	44	239.41
Result Component 8	21.47	59731.938	0.90	15.10	-1.095	300593658	0.9	1.11	41	217.18
Result Component 9	13.31	59733.070	0.64	10.75	0.037	298107673	0.9	1.10	42	257.50
Result Component 11	19.18	59933.627	1.10	18.41	200.594	200302659	0.6	0.74	40	199.85
Result Component 13	17.92	59687.388	1.55	25.99	-45.645	162773683	0.5	0.60	31	170.85
Result Component 15	21.21	59731.785	0.70	11.70	-1.249	129722977	0.4	0.48	41	225.58
Result Component 17	19.13	59766.513	1.08	18.11	33.479	91587642	0.3	0.34	38	197.64
Result Component 18	23.54	81372.238	1.92	23.54	21639.205	84227912	0.2	0.31	50	158.32
Result Component 19	18.85	59816.211	1.65	27.63	83.178	73795145	0.2	0.27	39	170.82
Result Component 20	19.45	59690.811	1.05	17.65	-42.222	72092544	0.2	0.27	37	195.30
Result Component 21	24.66	66290.558	1.28	19.26	6557.525	43412692	0.1	0.16	41	172.17

© 2009-2020 Thermo Fisher Scientific Inc. All rights reserved.
Chromeleon 7.3.0.60919

Component Summary

Injection Details

Injection Name:	**20211011_AAV5 E_45C_65-59B_Mod1_1ug**	*Injection Volume:*	**3.17**
Vial Number:	**R:A7**	*Instrument Method:*	**AAV Cap_Prot_Sep_BEH Glycoprotein Amide HILIC_1x150mm**
Injection Type:	**Unknown**	*Processing Method:*	**MS Quantitative_45C_65-59B**
Run Time (min):	**44.98**	*Injection Date/Time:*	**13/Oct/21 06:12**

Peak Name	Retention Time min	Area counts*min	Relative Area %	Quantitation Ions m/z	Mol.Mass	Mass Confirmation Pass/Fail
AAV5_VP3	14.579	2813589.775	87.79	1525.669,1565.784,1608.079,1451.304,1487.553,1416.767	59462.370	**PASS**
AAV5_VP1	24.095	40080.572	1.25	1362.628,1410.415,1435.577,1317.996,1339.949,1488.697	80335.080	**PASS**
AAV5_VP2	25.069	86266.766	2.69	1389.997,1519.197,1484.692,1420.196,1555.348,1451.727	65282.690	**PASS**
			**91.73**			

Component Name: AAV5_VP3

Quantitation ions:	1525.669,1565.784,1608.079,1451.304,1487.553,1416.767
Retention time (min):	14.53
Expected Mass:	59462.370
Area (count*min):	2813590
Intensity (counts):	5343269
Amount:	-

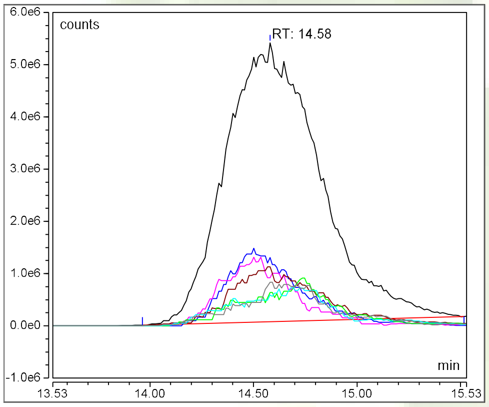

© 2009-2020 Thermo Fisher Scientific Inc. All rights reserved.
Chromeleon 7.3.0.60919

Component Name:	AAV8_VP3
Quantitation ions:	1574.800,1496.109,1534.442,1459.645,1273.445,1391.802
Retention time (min):	15.73
Expected Mass:	59804.680
Area (count*min):	-
Intensity (counts):	-
Amount:	-

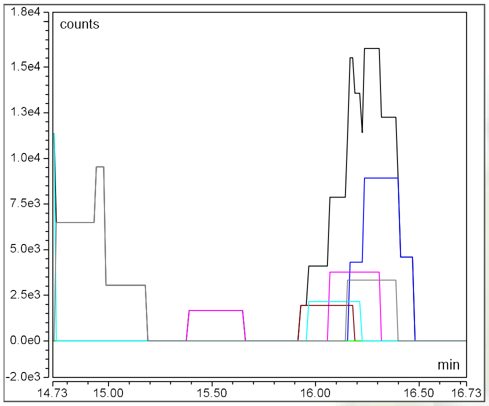

© 2009-2020 Thermo Fisher Scientific Inc. All rights reserved.
Chromeleon 7.3.0.60919

Component Name:	AAV9_VP3
Quantitation ions:	1358.581,1390.150,1299.556,1328.415,1271.930,1457.907
Retention time (min):	18.15
Expected Mass:	59732.620
Area (count*min):	-
Intensity (counts):	-
Amount:	-

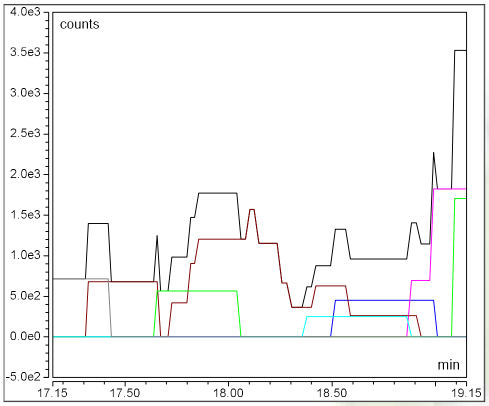

© 2009-2020 Thermo Fisher Scientific Inc. All rights reserved.
Chromeleon 7.3.0.60919

Component Name:	AAV2_VP3
Quantitation ions:	1428.955,1500.355,1463.789,1395.748,1364.055,1538.792
Retention time (min):	20.38
Expected Mass:	59973.780
Area (count*min):	-
Intensity (counts):	-
Amount:	-

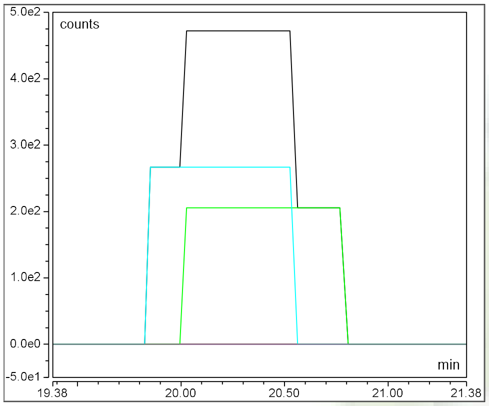

© 2009-2020 Thermo Fisher Scientific Inc. All rights reserved.
Chromeleon 7.3.0.60919

Component Name:	AAV9_VP1
Quantitation ions:	1378.820,1333.651,1355.868,1402.584,1534.808,1452.636
Retention time (min):	23.16
Expected Mass:	81290.340
Area (count*min):	-
Intensity (counts):	-
Amount:	-

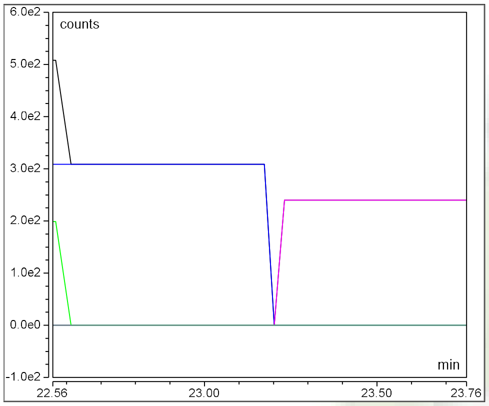

© 2009-2020 Thermo Fisher Scientific Inc. All rights reserved.
Chromeleon 7.3.0.60919

Component Name:	AAV8_VP1
Quantitation ions:	1409.080,1459.382,1385.209,1513.388,1184.605,1339.828
Retention time (min):	23.33
Expected Mass:	81666.710
Area (count*min):	-
Intensity (counts):	-
Amount:	-

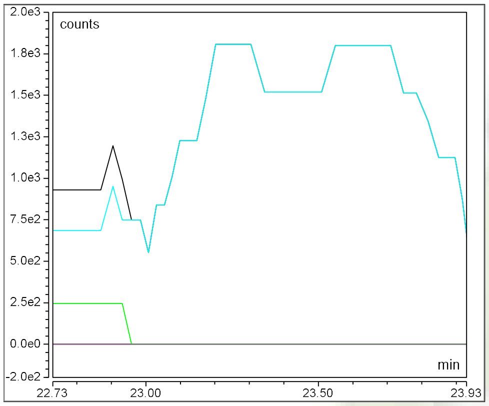

© 2009-2020 Thermo Fisher Scientific Inc. All rights reserved.
Chromeleon 7.3.0.60919

Component Name: AAV5_VP1

Quantitation ions:	1362.628,1410.415,1435.577,1317.996,1339.949,1488.697
Retention time (min):	24.09
Expected Mass:	80335.080
Area (count*min):	40081
Intensity (counts):	87360
Amount:	-

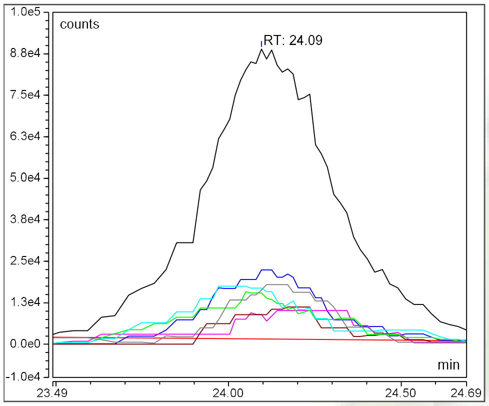

© 2009-2020 Thermo Fisher Scientific Inc. All rights reserved.
Chromeleon 7.3.0.60919

Component Name: AAV9_VP2

Quantitation ions:	1409.730,1162.589,1472.334,1274.287,1380.371,1352.231
Retention time (min):	24.10
Expected Mass:	66209.760
Area (count*min):	-
Intensity (counts):	-
Amount:	-

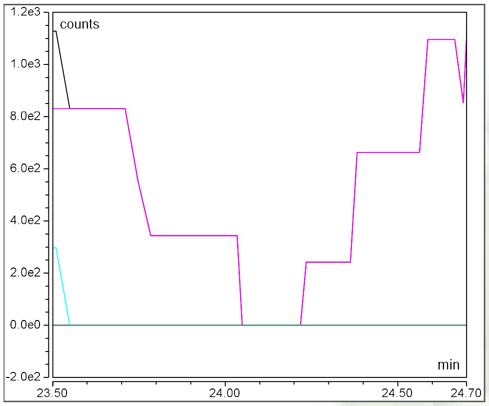

© 2009-2020 Thermo Fisher Scientific Inc. All rights reserved.
Chromeleon 7.3.0.60919

Component Name: AAV8_VP2	
Quantitation ions:	1386.809,1358.524,1331.393,1305.308,1416.294,1623.397
Retention time (min):	24.50
Expected Mass:	66518.100
Area (count*min):	-
Intensity (counts):	-
Amount:	-

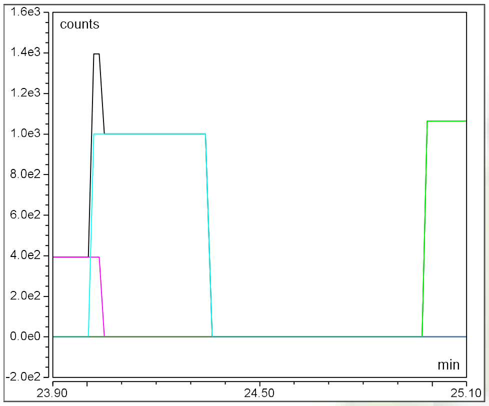

© 2009-2020 Thermo Fisher Scientific Inc. All rights reserved.
Chromeleon 7.3.0.60919

Component Name: AAV5_VP2

Quantitation ions:	1389.997,1519.197,1484.692,1420.196,1555.348,1451.727
Retention time (min):	25.16
Expected Mass:	65282.690
Area (count*min):	86267
Intensity (counts):	213000
Amount:	-

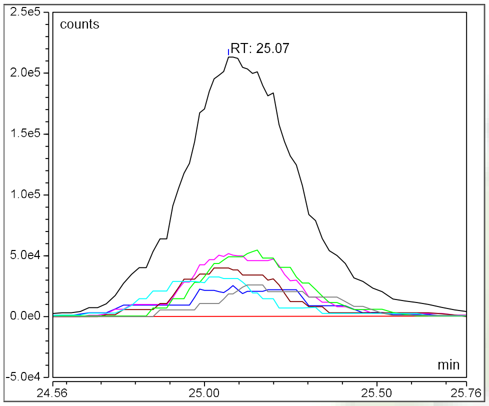

© 2009-2020 Thermo Fisher Scientific Inc. All rights reserved.
Chromeleon 7.3.0.60919

Component Name:	AAV2_VP1
Quantitation ions:	1388.434,1545.445,1489.336,1342.917,1365.283,1412.362
Retention time (min):	25.18
Expected Mass:	81854.900
Area (count*min):	-
Intensity (counts):	-
Amount:	-

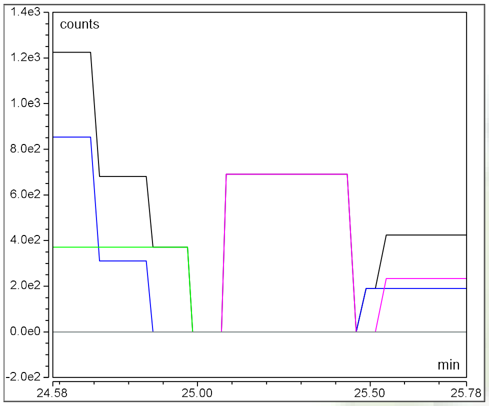

© 2009-2020 Thermo Fisher Scientific Inc. All rights reserved.
Chromeleon 7.3.0.60919

Component Name:	AAV2_VP2
Quantitation ions:	1415.636,1446.415,1255.530,1478.506,1279.609,1304.686
Retention time (min):	26.20
Expected Mass:	66487.900
Area (count*min):	-
Intensity (counts):	-
Amount:	-

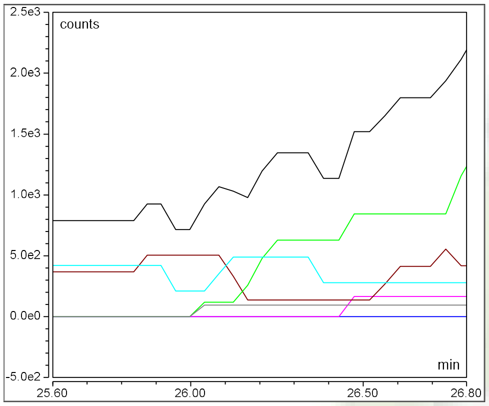

© 2009-2020 Thermo Fisher Scientific Inc. All rights reserved.
Chromeleon 7.3.0.60919

Chromatogram and SST Results

Injection Details

Injection Name:	**20211011_AAV5 E_45C_65-59B_Mod1_1ug_2**	*Run Time (min):*	**44.98**
Vial Number:	**R:A7**	*Injection Volume:*	**3.17**
Injection Type:	**Unknown**		
Calibration Level:			
Instrument Method:	**AAV Cap_Prot_Sep_BEH Glycoprotein Amide HILIC_1x150mm_FLR + MS_CT45C_65-59_S**		
Processing Method:	**MS Quantitative_45C_65-59B**	*Dilution Factor:*	**1.0000**
Injection Date/Time:	**13/Oct/21 06:12**	*Sample Weight:*	**1.0000**

Chromatogram

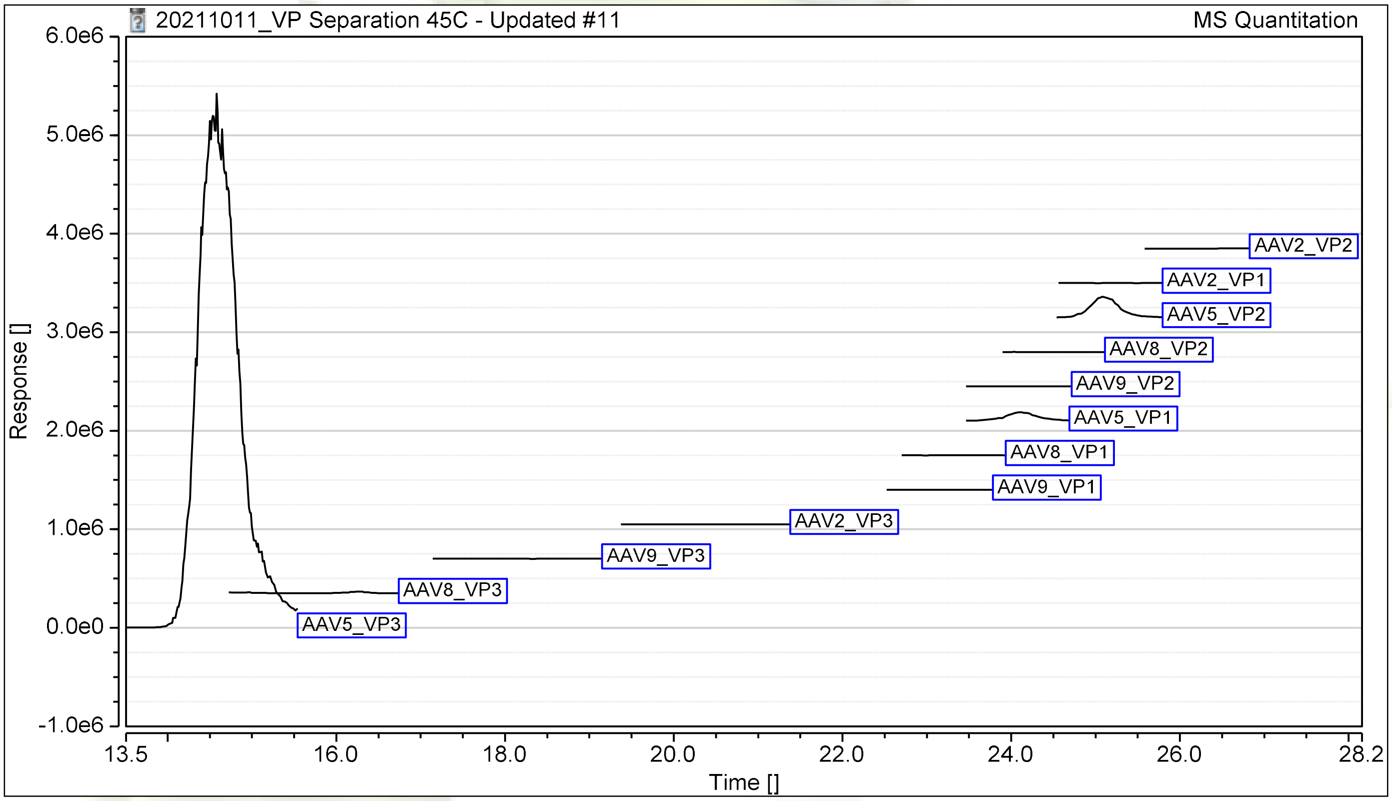

SST Results

No.	Name	Inj.Condition	Peak	Test Result	Injection
**Number of executed test cases:**	**n.a.**		**Total Result:**	**Passed**	

© 2009-2020 Thermo Fisher Scientific Inc. All rights reserved.
Chromeleon 7.3.0.60919

Protein Deconvolution Summary

Injection Details

Injection Name:	**20211011_AAV5 E_45C_65-59B_Mod1_1ug_3**	*Injection Volume:*	**3.17**
Vial Number:	**R:A7**	*Instrument Method:*	**AAV Cap_Prot_Sep_BEH Glycoprotein Amide H**
Injection Type:	**Unknown**	*Processing Method:*	**MS Quantitative_45C_65-59B**
Run Time (min):	**45.00**	*Injection Date/Time:*	**13/Oct/21 06:58**

Chromatogram

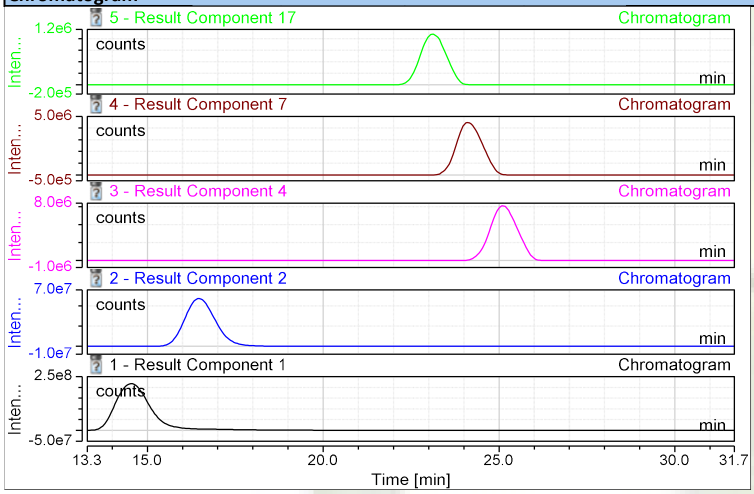

Source Spectrum

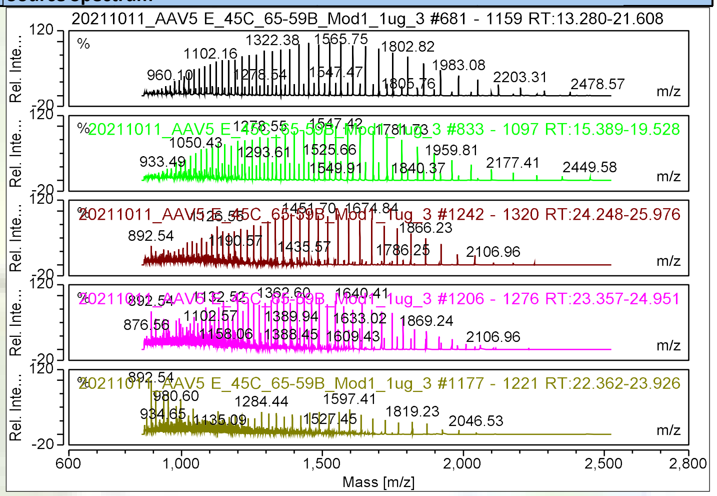

Deconvoluted Spectrum

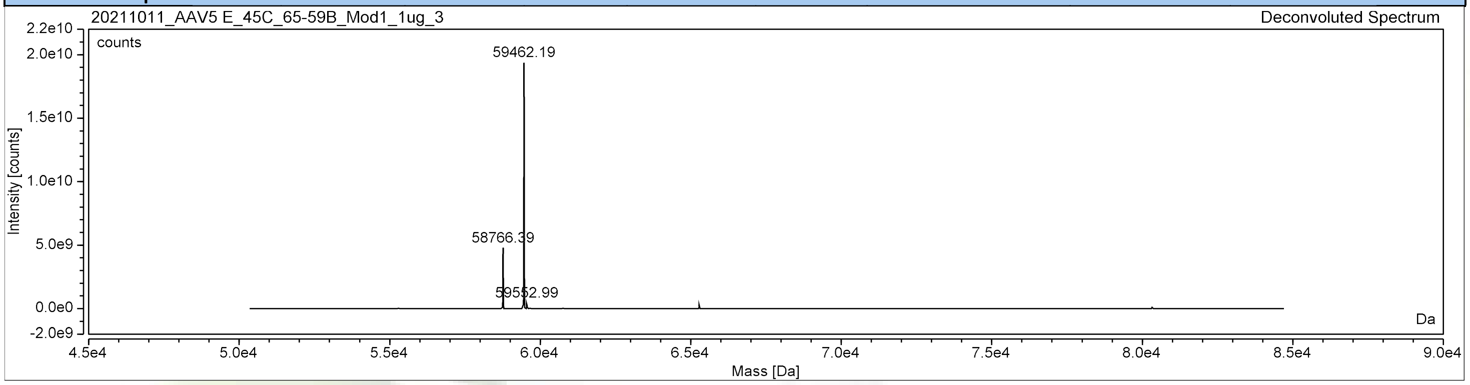

Deconvolution Results

Result Component	RT min	Mass Da	Std. Dev. Da	Std. Dev. ppm	Delta Mass Da	Intensity counts	Fractional Abundance counts	Rel. Abundance %	Number of Charge States	Score
Result Component 1	18.11	59733.033	0.40	6.68	0.000	27034264760	80.1	100.00	45	238.14
Result Component 2	18.22	59820.302	0.95	15.91	87.269	1527586438	4.5	5.65	40	170.79
Result Component 3	23.18	81291.415	0.91	11.13	21558.382	643910272	1.9	2.38	56	291.15
Result Component 4	19.20	59813.944	0.85	14.29	80.911	471832605	1.4	1.75	43	234.57
Result Component 5	18.27	59686.960	1.22	20.37	-46.073	390164875	1.2	1.44	32	176.14
Result Component 7	24.10	66210.268	0.85	12.85	6477.235	340089971	1.0	1.26	44	239.41
Result Component 8	21.47	59731.938	0.90	15.10	-1.095	300593658	0.9	1.11	41	217.18
Result Component 9	13.31	59733.070	0.64	10.75	0.037	298107673	0.9	1.10	42	257.50
Result Component 11	19.18	59933.627	1.10	18.41	200.594	200302659	0.6	0.74	40	199.85
Result Component 13	17.92	59687.388	1.55	25.99	-45.645	162773683	0.5	0.60	31	170.85
Result Component 15	21.21	59731.785	0.70	11.70	-1.249	129722977	0.4	0.48	41	225.58
Result Component 17	19.13	59766.513	1.08	18.11	33.479	91587642	0.3	0.34	38	197.64
Result Component 18	23.54	81372.238	1.92	23.54	21639.205	84227912	0.2	0.31	50	158.32
Result Component 19	18.85	59816.211	1.65	27.63	83.178	73795145	0.2	0.27	39	170.82
Result Component 20	19.45	59690.811	1.05	17.65	-42.222	72092544	0.2	0.27	37	195.30
Result Component 21	24.66	66290.558	1.28	19.26	6557.525	43412692	0.1	0.16	41	172.17

© 2009-2020 Thermo Fisher Scientific Inc. All rights reserved.
Chromeleon 7.3.0.60919

Component Summary

Injection Details

Injection Name:	**20211011_AAV5 E_45C_65-59B_Mod1_1ug**	*Injection Volume:*	**3.17**
Vial Number:	**R:A7**	*Instrument Method:*	**AAV Cap_Prot_Sep_BEH Glycoprotein Amide HILIC_1x150mm**
Injection Type:	**Unknown**	*Processing Method:*	**MS Quantitative_45C_65-59B**
Run Time (min):	**45.00**	*Injection Date/Time:*	**13/Oct/21 06:58**

Peak Name	Retention Time min	Area counts*min	Relative Area %	Quantitation Ions m/z	Mol.Mass	Mass Confirmation Pass/Fail
AAV5_VP3	14.478	3383440.026	91.46	1525.669,1565.784,1608.079,1451.304,1487.553,1416.767	59462.370	**PASS**
AAV5_VP1	24.186	32188.637	0.87	1362.628,1410.415,1435.577,1317.996,1339.949,1488.697	80335.080	**PASS**
AAV5_VP2	25.076	67494.962	1.82	1389.997,1519.197,1484.692,1420.196,1555.348,1451.727	65282.690	**PASS**
			**94.15**			

Component Name: AAV5_VP3

Quantitation ions:	1525.669,1565.784,1608.079,1451.304,1487.553,1416.767
Retention time (min):	14.53
Expected Mass:	59462.370
Area (count*min):	3383440
Intensity (counts):	5752788
Amount:	-

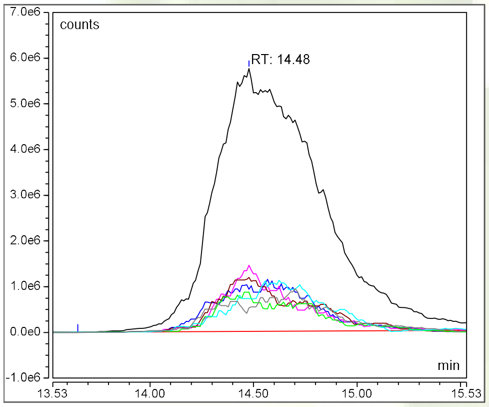

© 2009-2020 Thermo Fisher Scientific Inc. All rights reserved.
Chromeleon 7.3.0.60919

Component Name: AAV8_VP3

Quantitation ions:	1574.800,1496.109,1534.442,1459.645,1273.445,1391.802
Retention time (min):	15.73
Expected Mass:	59804.680
Area (count*min):	-
Intensity (counts):	-
Amount:	-

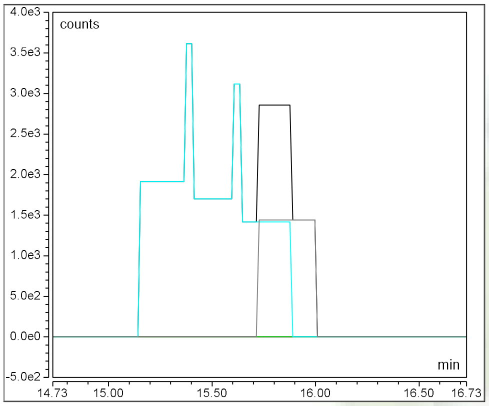

© 2009-2020 Thermo Fisher Scientific Inc. All rights reserved.
Chromeleon 7.3.0.60919

Component Name: AAV9_VP3

Quantitation ions:	1358.581,1390.150,1299.556,1328.415,1271.930,1457.907
Retention time (min):	18.15
Expected Mass:	59732.620
Area (count*min):	-
Intensity (counts):	-
Amount:	-

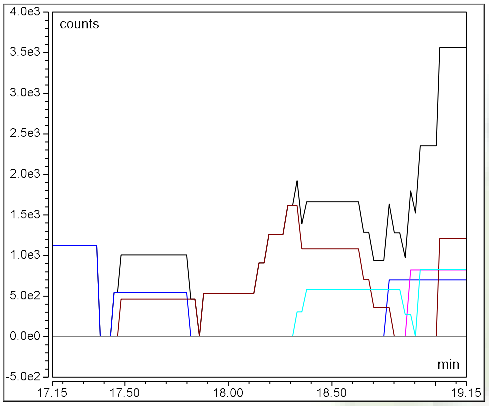

© 2009-2020 Thermo Fisher Scientific Inc. All rights reserved.
Chromeleon 7.3.0.60919

Component Name:	AAV2_VP3
Quantitation ions:	1428.955,1500.355,1463.789,1395.748,1364.055,1538.792
Retention time (min):	20.38
Expected Mass:	59973.780
Area (count*min):	-
Intensity (counts):	-
Amount:	-

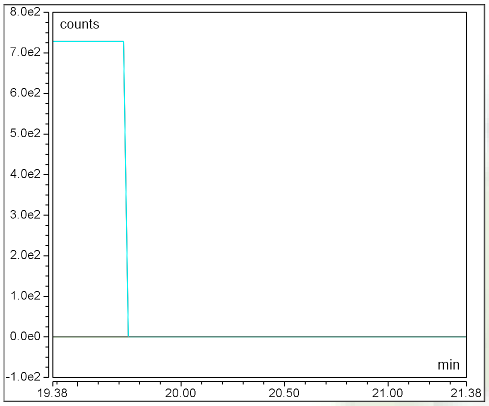

© 2009-2020 Thermo Fisher Scientific Inc. All rights reserved.
Chromeleon 7.3.0.60919

Component Name: AAV9_VP1

Quantitation ions:	1378.820,1333.651,1355.868,1402.584,1534.808,1452.636
Retention time (min):	23.16
Expected Mass:	81290.340
Area (count*min):	-
Intensity (counts):	-
Amount:	-

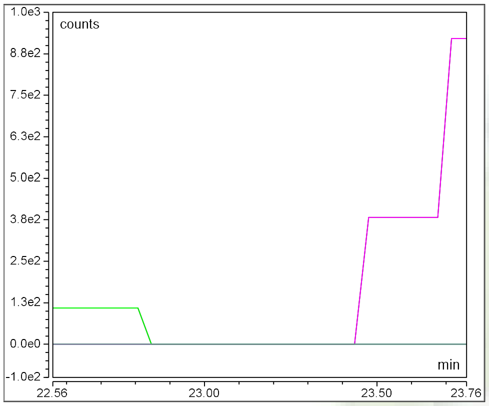

© 2009-2020 Thermo Fisher Scientific Inc. All rights reserved.
Chromeleon 7.3.0.60919

Component Name:	AAV8_VP1
Quantitation ions:	1409.080,1459.382,1385.209,1513.388,1184.605,1339.828
Retention time (min):	23.33
Expected Mass:	81666.710
Area (count*min):	-
Intensity (counts):	-
Amount:	-

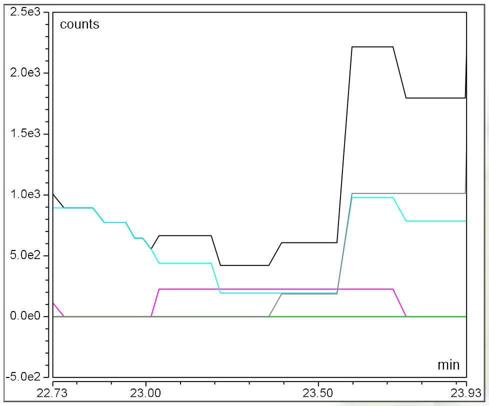

© 2009-2020 Thermo Fisher Scientific Inc. All rights reserved.
Chromeleon 7.3.0.60919

Component Name:	AAV5_VP1
Quantitation ions:	1362.628,1410.415,1435.577,1317.996,1339.949,1488.697
Retention time (min):	24.09
Expected Mass:	80335.080
Area (count*min):	32189
Intensity (counts):	83792
Amount:	-

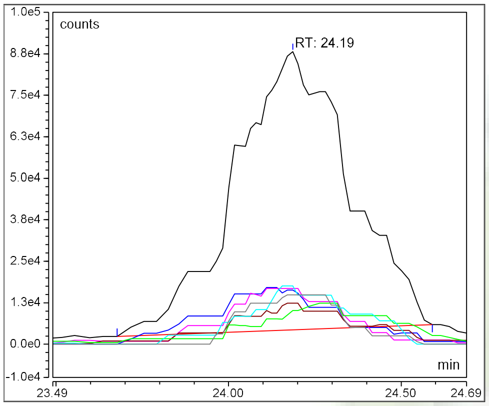

© 2009-2020 Thermo Fisher Scientific Inc. All rights reserved.
Chromeleon 7.3.0.60919

Component Name: AAV9_VP2

Quantitation ions:	1409.730,1162.589,1472.334,1274.287,1380.371,1352.231
Retention time (min):	24.10
Expected Mass:	66209.760
Area (count*min):	-
Intensity (counts):	-
Amount:	-

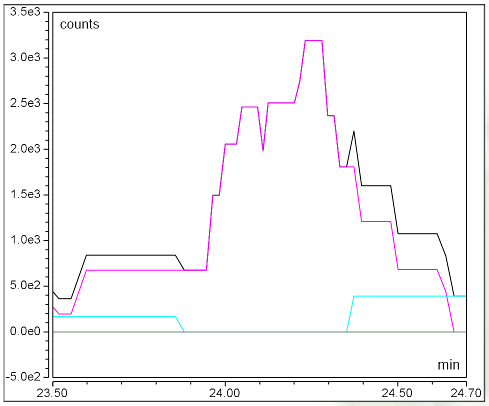

© 2009-2020 Thermo Fisher Scientific Inc. All rights reserved.
Chromeleon 7.3.0.60919

Component Name:	AAV8_VP2
Quantitation ions:	1386.809,1358.524,1331.393,1305.308,1416.294,1623.397
Retention time (min):	24.50
Expected Mass:	66518.100
Area (count*min):	-
Intensity (counts):	-
Amount:	-

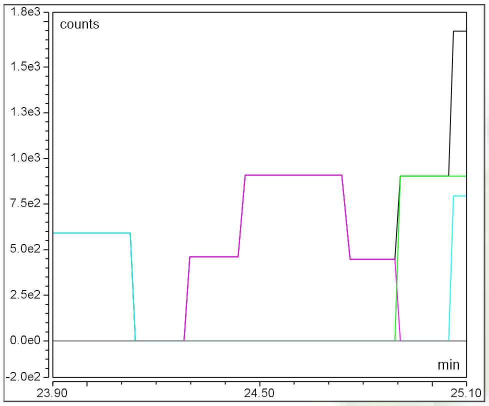

© 2009-2020 Thermo Fisher Scientific Inc. All rights reserved.
Chromeleon 7.3.0.60919

Component Name:	AAV5_VP2
Quantitation ions:	1389.997,1519.197,1484.692,1420.196,1555.348,1451.727
Retention time (min):	25.16
Expected Mass:	65282.690
Area (count*min):	67495
Intensity (counts):	155649
Amount:	-

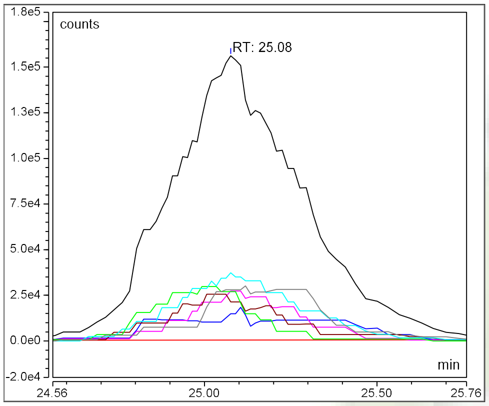

© 2009-2020 Thermo Fisher Scientific Inc. All rights reserved.
Chromeleon 7.3.0.60919

Component Name:	AAV2_VP1
Quantitation ions:	1388.434,1545.445,1489.336,1342.917,1365.283,1412.362
Retention time (min):	25.18
Expected Mass:	81854.900
Area (count*min):	-
Intensity (counts):	-
Amount:	-

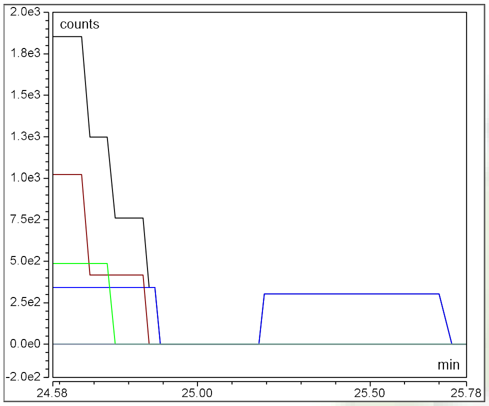

© 2009-2020 Thermo Fisher Scientific Inc. All rights reserved.
Chromeleon 7.3.0.60919

Component Name:	AAV2_VP2
Quantitation ions:	1415.636,1446.415,1255.530,1478.506,1279.609,1304.686
Retention time (min):	26.20
Expected Mass:	66487.900
Area (count*min):	-
Intensity (counts):	-
Amount:	-

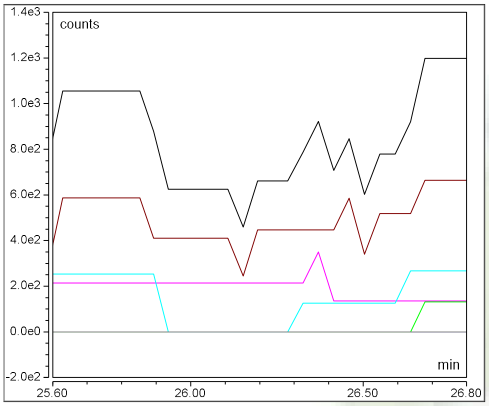

© 2009-2020 Thermo Fisher Scientific Inc. All rights reserved.
Chromeleon 7.3.0.60919

Chromatogram and SST Results

Injection Details

Injection Name:	**20211011_AAV5 E_45C_65-59B_Mod1_1ug_3**	*Run Time (min):*	**45.00**
Vial Number:	**R:A7**	*Injection Volume:*	**3.17**
Injection Type:	**Unknown**		
Calibration Level:			
Instrument Method:	**AAV Cap_Prot_Sep_BEH Glycoprotein Amide HILIC_1x150mm_FLR + MS_CT45C_65-59_S**		
Processing Method:	**MS Quantitative_45C_65-59B**	*Dilution Factor:*	**1.0000**
Injection Date/Time:	**13/Oct/21 06:58**	*Sample Weight:*	**1.0000**

Chromatogram

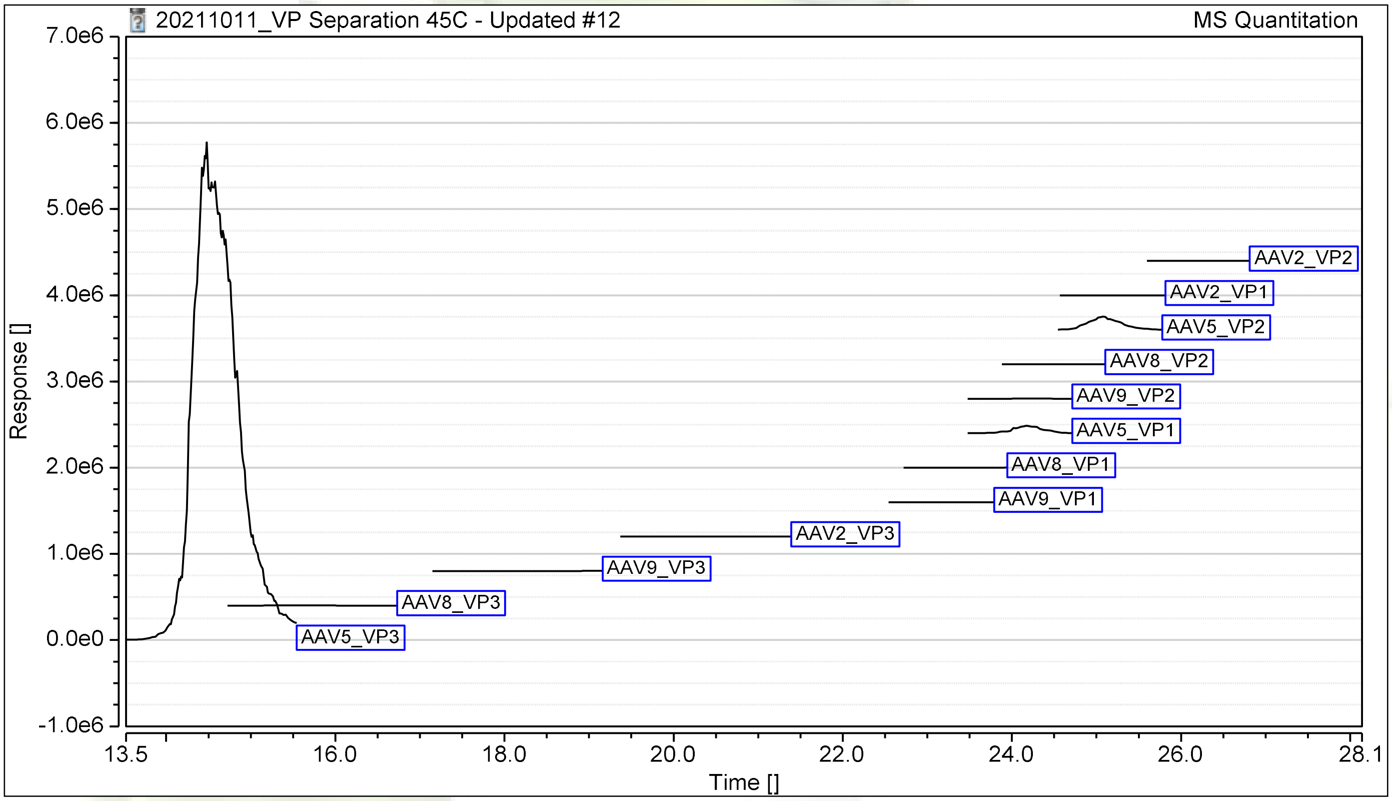

SST Results

No.	Name	Inj.Condition	Peak	Test Result	Injection

Number of executed test cases: **n.a.** **Total Result:** **Passed**

© 2009-2020 Thermo Fisher Scientific Inc. All rights reserved.
Chromeleon 7.3.0.60919

Protein Deconvolution Summary

Injection Details

Injection Name:	**20211011_AAV2 Mixed_45C_65-59B_Mod1_1ug_1**	*Injection Volume:*	**8.45**
Vial Number:	**R:A9**	*Instrument Method:*	**AAV Cap_Prot_Sep_BEH Glycoprotein Amide H**
Injection Type:	**Unknown**	*Processing Method:*	**MS Quantitative_45C_65-59B**
Run Time (min):	**45.00**	*Injection Date/Time:*	**12/Oct/21 18:13**

Chromatogram

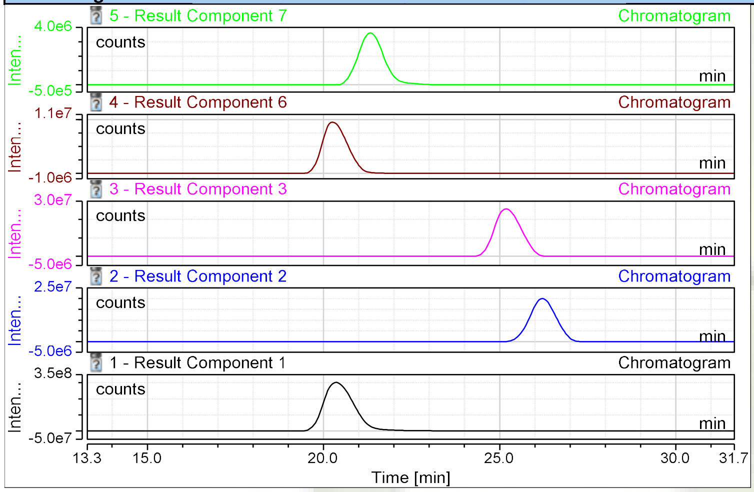

Source Spectrum

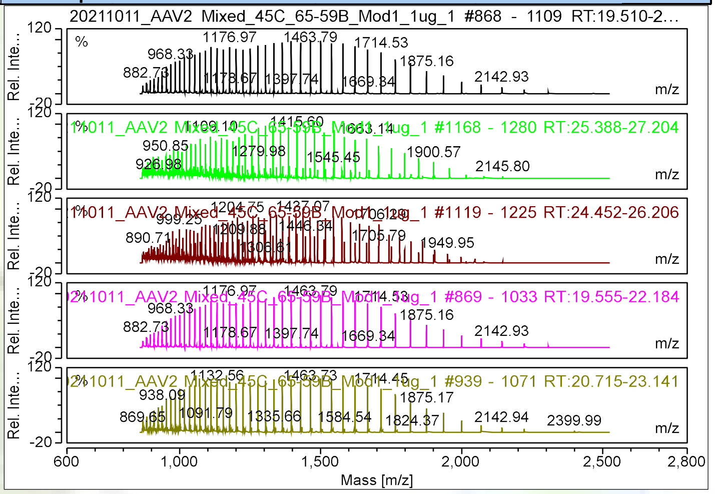

Deconvoluted Spectrum

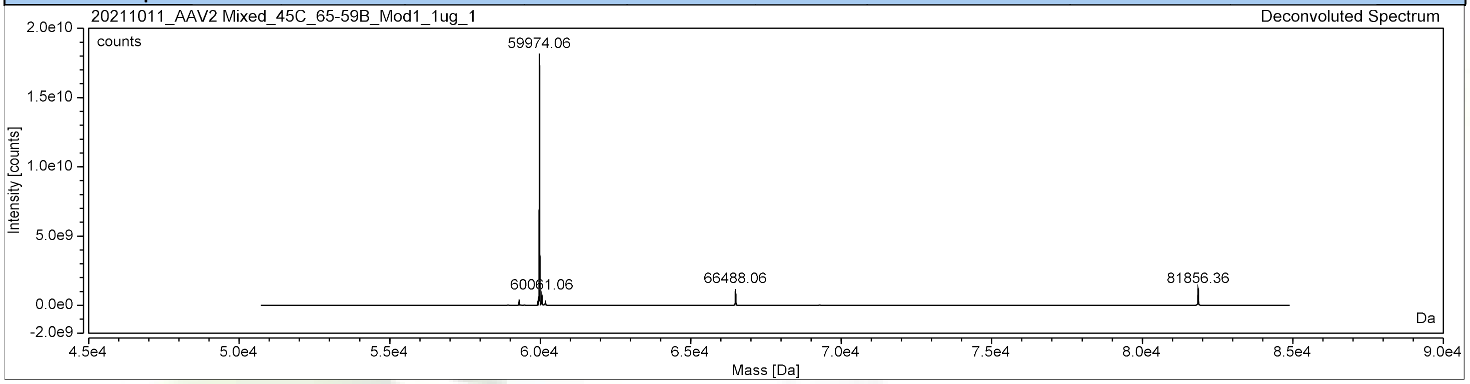

Deconvolution Results

Result Component	RT min	Mass Da	Std. Dev. Da	Std. Dev. ppm	Delta Mass Da	Intensity counts	Fractional Abundance counts	Rel. Abundance %	Number of Charge States	Score
Result Component 1	18.11	59733.033	0.40	6.68	0.000	27034264760	80.1	100.00	45	238.14
Result Component 2	18.22	59820.302	0.95	15.91	87.269	1527586438	4.5	5.65	40	170.79
Result Component 3	23.18	81291.415	0.91	11.13	21558.382	643910272	1.9	2.38	56	291.15
Result Component 4	19.20	59813.944	0.85	14.29	80.911	471832605	1.4	1.75	43	234.57
Result Component 5	18.27	59686.960	1.22	20.37	-46.073	390164875	1.2	1.44	32	176.14
Result Component 7	24.10	66210.268	0.85	12.85	6477.235	340089971	1.0	1.26	44	239.41
Result Component 8	21.47	59731.938	0.90	15.10	-1.095	300593658	0.9	1.11	41	217.18
Result Component 9	13.31	59733.070	0.64	10.75	0.037	298107673	0.9	1.10	42	257.50
Result Component 11	19.18	59933.627	1.10	18.41	200.594	200302659	0.6	0.74	40	199.85
Result Component 13	17.92	59687.388	1.55	25.99	-45.645	162773683	0.5	0.60	31	170.85
Result Component 15	21.21	59731.785	0.70	11.70	-1.249	129722977	0.4	0.48	41	225.58
Result Component 17	19.13	59766.513	1.08	18.11	33.479	91587642	0.3	0.34	38	197.64
Result Component 18	23.54	81372.238	1.92	23.54	21639.205	84227912	0.2	0.31	50	158.32
Result Component 19	18.85	59816.211	1.65	27.63	83.178	73795145	0.2	0.27	39	170.82
Result Component 20	19.45	59690.811	1.05	17.65	-42.222	72092544	0.2	0.27	37	195.30
Result Component 21	24.66	66290.558	1.28	19.26	6557.525	43412692	0.1	0.16	41	172.17

© 2009-2020 Thermo Fisher Scientific Inc. All rights reserved.
Chromeleon 7.3.0.60919

Component Summary

Injection Details

Injection Name:	**20211011_AAV2 Mixed_45C_65-59B_Mod1**	*Injection Volume:*	**8.45**
Vial Number:	**R:A9**	*Instrument Method:*	**AAV Cap_Prot_Sep_BEH Glycoprotein Amide HILIC_1x150mm**
Injection Type:	**Unknown**	*Processing Method:*	**MS Quantitative_45C_65-59B**
Run Time (min):	**45.00**	*Injection Date/Time:*	**12/Oct/21 18:13**

Peak Name	Retention Time min	Area counts*min	Relative Area %	Quantitation Ions m/z	Mol.Mass	Mass Confirmation Pass/Fail
AAV2_VP3	20.399	3830196.738	79.17	1428.955,1500.355,1463.789,1395.748,1364.055,1538.792	59973.780	**PASS**
AAV2_VP1	25.230	145835.140	3.01	1388.434,1545.445,1489.336,1342.917,1365.283,1412.362	81854.900	**PASS**
AAV2_VP2	26.206	179370.301	3.71	1415.636,1446.415,1255.530,1478.506,1279.609,1304.686	66487.900	**PASS**
			**85.89**			

Component Name: AAV5_VP3

Quantitation ions:	1525.669,1565.784,1608.079,1451.304,1487.553,1416.767
Retention time (min):	14.53
Expected Mass:	59462.370
Area (count*min):	-
Intensity (counts):	-
Amount:	-

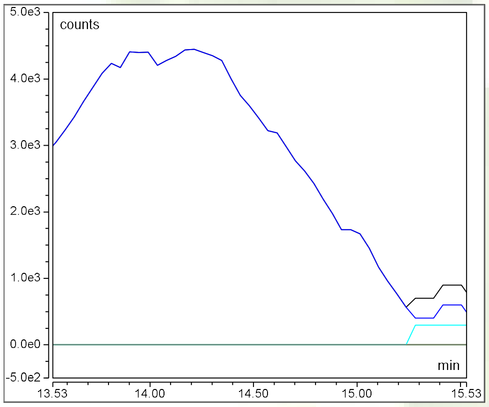

© 2009-2020 Thermo Fisher Scientific Inc. All rights reserved.
Chromeleon 7.3.0.60919

Component Name:	AAV8_VP3
Quantitation ions:	1574.800,1496.109,1534.442,1459.645,1273.445,1391.802
Retention time (min):	15.73
Expected Mass:	59804.680
Area (count*min):	-
Intensity (counts):	-
Amount:	-

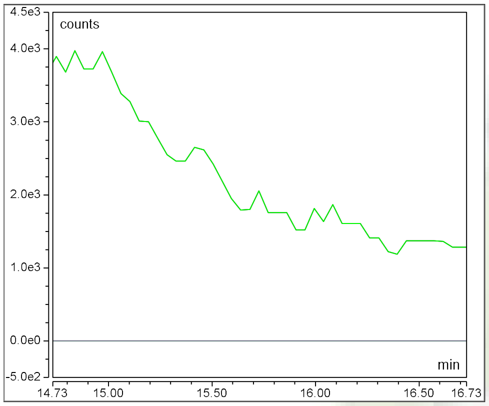

© 2009-2020 Thermo Fisher Scientific Inc. All rights reserved.
Chromeleon 7.3.0.60919

Component Name: AAV9_VP3

Quantitation ions:	1358.581,1390.150,1299.556,1328.415,1271.930,1457.907
Retention time (min):	18.15
Expected Mass:	59732.620
Area (count*min):	-
Intensity (counts):	-
Amount:	-

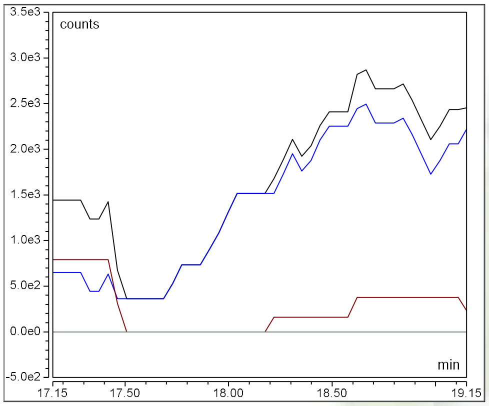

© 2009-2020 Thermo Fisher Scientific Inc. All rights reserved.
Chromeleon 7.3.0.60919

Component Name: AAV2_VP3

Quantitation ions:	1428.955,1500.355,1463.789,1395.748,1364.055,1538.792
Retention time (min):	20.38
Expected Mass:	59973.780
Area (count*min):	3830197
Intensity (counts):	8641216
Amount:	-

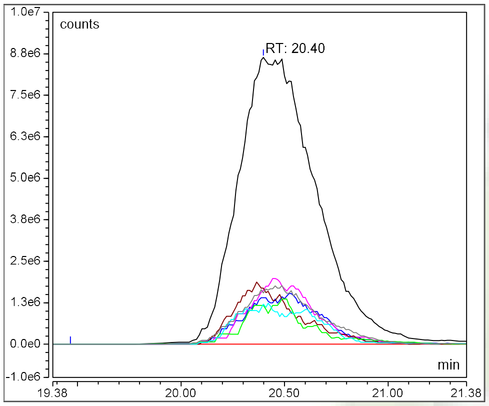

© 2009-2020 Thermo Fisher Scientific Inc. All rights reserved.
Chromeleon 7.3.0.60919

Component Name: AAV9_VP1

Quantitation ions:	1378.820,1333.651,1355.868,1402.584,1534.808,1452.636
Retention time (min):	23.16
Expected Mass:	81290.340
Area (count*min):	-
Intensity (counts):	-
Amount:	-

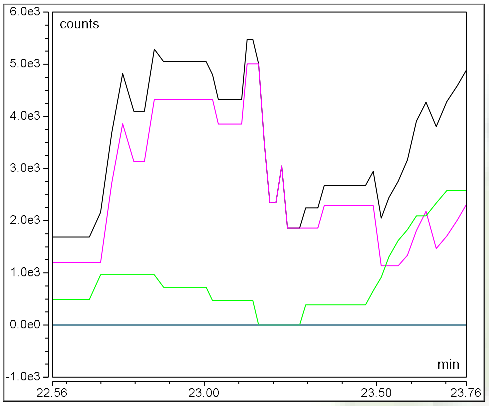

© 2009-2020 Thermo Fisher Scientific Inc. All rights reserved.
Chromeleon 7.3.0.60919

Component Name:	AAV8_VP1
Quantitation ions:	1409.080,1459.382,1385.209,1513.388,1184.605,1339.828
Retention time (min):	23.33
Expected Mass:	81666.710
Area (count*min):	-
Intensity (counts):	-
Amount:	-

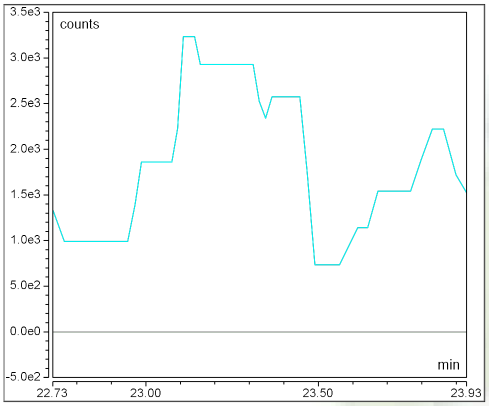

© 2009-2020 Thermo Fisher Scientific Inc. All rights reserved.
Chromeleon 7.3.0.60919

Component Name:	AAV5_VP1
Quantitation ions:	1362.628,1410.415,1435.577,1317.996,1339.949,1488.697
Retention time (min):	24.09
Expected Mass:	80335.080
Area (count*min):	-
Intensity (counts):	-
Amount:	-

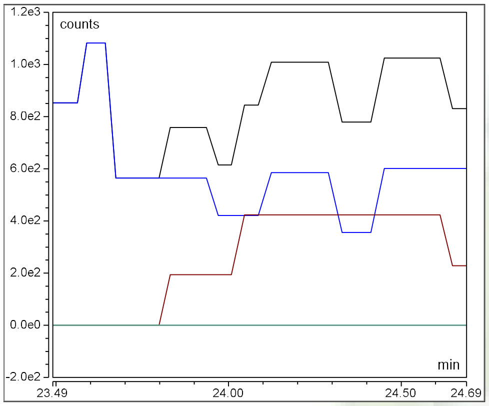

© 2009-2020 Thermo Fisher Scientific Inc. All rights reserved.
Chromeleon 7.3.0.60919

Component Name:	AAV9_VP2
Quantitation ions:	1409.730,1162.589,1472.334,1274.287,1380.371,1352.231
Retention time (min):	24.10
Expected Mass:	66209.760
Area (count*min):	-
Intensity (counts):	-
Amount:	-

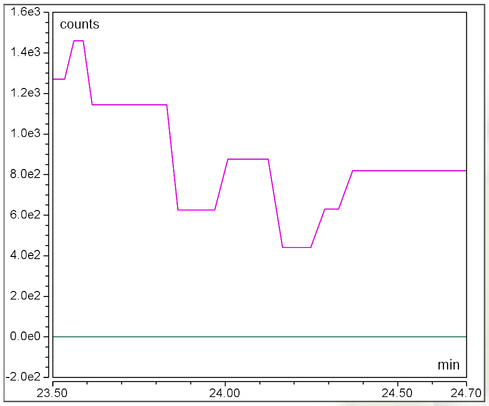

© 2009-2020 Thermo Fisher Scientific Inc. All rights reserved.
Chromeleon 7.3.0.60919

Component Name:	AAV8_VP2
Quantitation ions:	1386.809,1358.524,1331.393,1305.308,1416.294,1623.397
Retention time (min):	24.50
Expected Mass:	66518.100
Area (count*min):	-
Intensity (counts):	-
Amount:	-

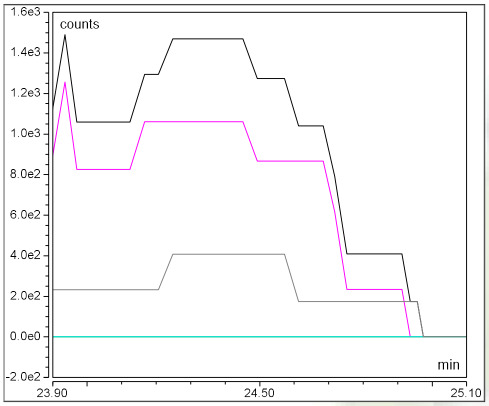

© 2009-2020 Thermo Fisher Scientific Inc. All rights reserved.
Chromeleon 7.3.0.60919

Component Name: AAV5_VP2

Quantitation ions:	1389.997,1519.197,1484.692,1420.196,1555.348,1451.727
Retention time (min):	25.16
Expected Mass:	65282.690
Area (count*min):	-
Intensity (counts):	-
Amount:	-

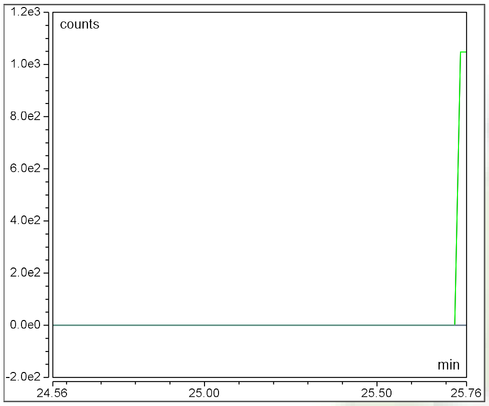

© 2009-2020 Thermo Fisher Scientific Inc. All rights reserved.
Chromeleon 7.3.0.60919

Component Name: AAV2_VP1	
Quantitation ions:	1388.434,1545.445,1489.336,1342.917,1365.283,1412.362
Retention time (min):	25.18
Expected Mass:	81854.900
Area (count*min):	145835
Intensity (counts):	349122
Amount:	-

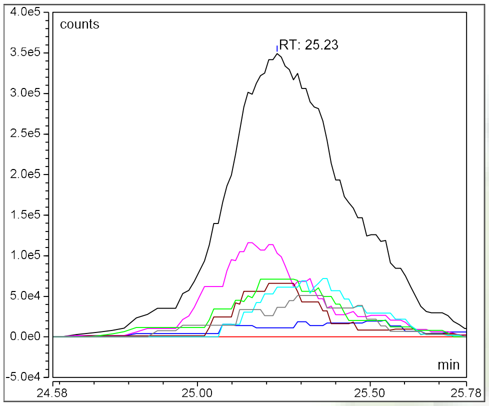

© 2009-2020 Thermo Fisher Scientific Inc. All rights reserved.
Chromeleon 7.3.0.60919

Component Name:	AAV2_VP2
Quantitation ions:	1415.636,1446.415,1255.530,1478.506,1279.609,1304.686
Retention time (min):	26.20
Expected Mass:	66487.900
Area (count*min):	179370
Intensity (counts):	455688
Amount:	-

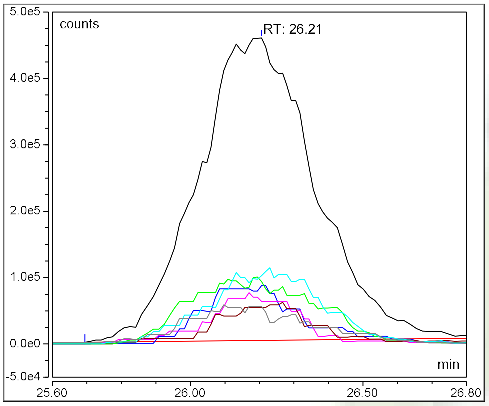

© 2009-2020 Thermo Fisher Scientific Inc. All rights reserved.
Chromeleon 7.3.0.60919

Chromatogram and SST Results

Injection Details

Injection Name:	**20211011_AAV2 Mixed_45C_65-59B_Mod1_1ug_1**	*Run Time (min):*	**45.00**
Vial Number:	**R:A9**	*Injection Volume:*	**8.45**
Injection Type:	**Unknown**		
Calibration Level:			
Instrument Method:	**AAV Cap_Prot_Sep_BEH Glycoprotein Amide HILIC_1x150mm_FLR + MS_CT45C_65-59_S**		
Processing Method:	**MS Quantitative_45C_65-59B**	*Dilution Factor:*	**1.0000**
Injection Date/Time:	**12/Oct/21 18:13**	*Sample Weight:*	**1.0000**

Chromatogram

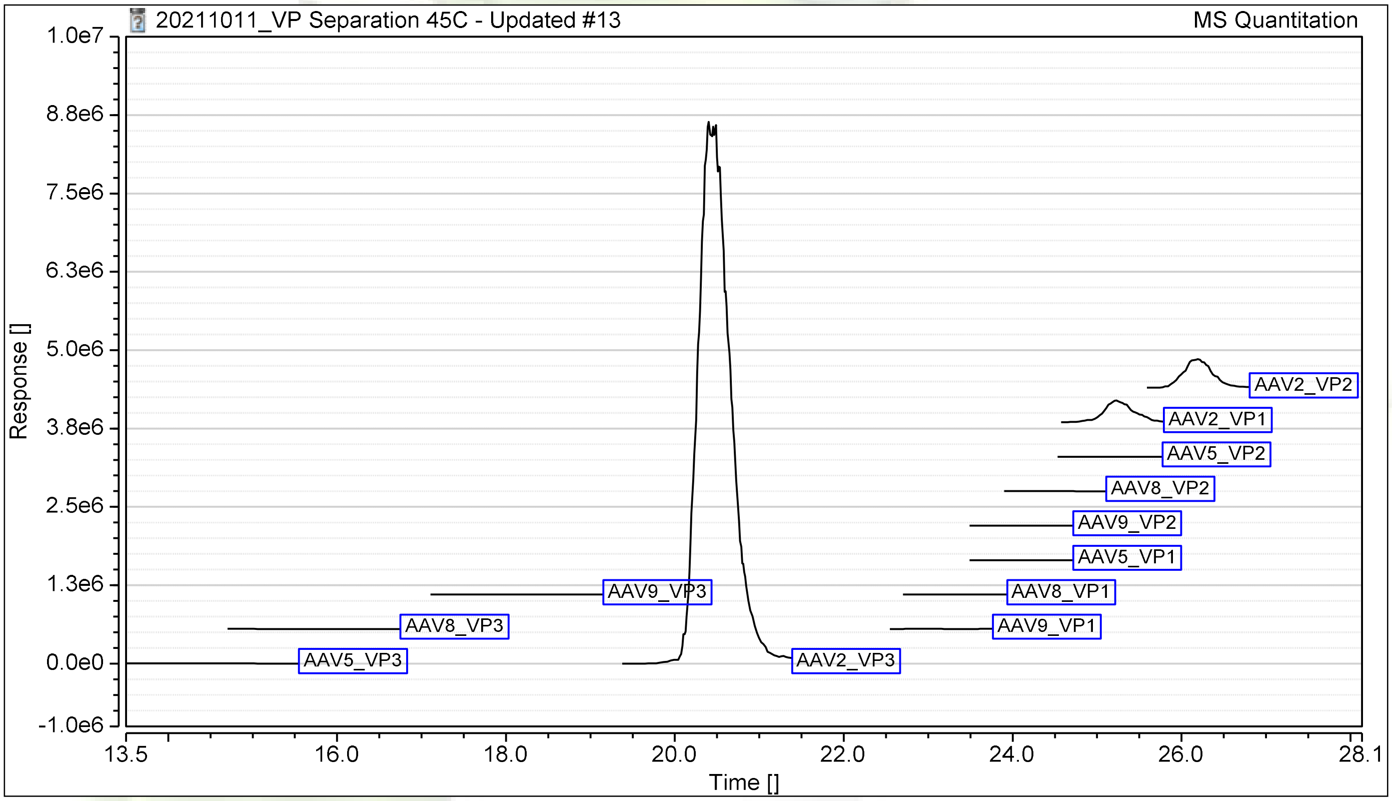

SST Results

No.	Name	Inj.Condition	Peak	Test Result	Injection
**Number of executed test cases:**	**n.a.**		**Total Result:**	**Passed**	

© 2009-2020 Thermo Fisher Scientific Inc. All rights reserved.
Chromeleon 7.3.0.60919

Protein Deconvolution Summary

Injection Details

Injection Name:	**20211011_AAV2 Mixed_45C_65-59B_Mod1_1ug_2**	*Injection Volume:*	**8.45**
Vial Number:	**R:A9**	*Instrument Method:*	**AAV Cap_Prot_Sep_BEH Glycoprotein Amide H**
Injection Type:	**Unknown**	*Processing Method:*	**MS Quantitative_45C_65-59B**
Run Time (min):	**44.98**	*Injection Date/Time:*	**12/Oct/21 18:59**

Chromatogram / Source Spectrum

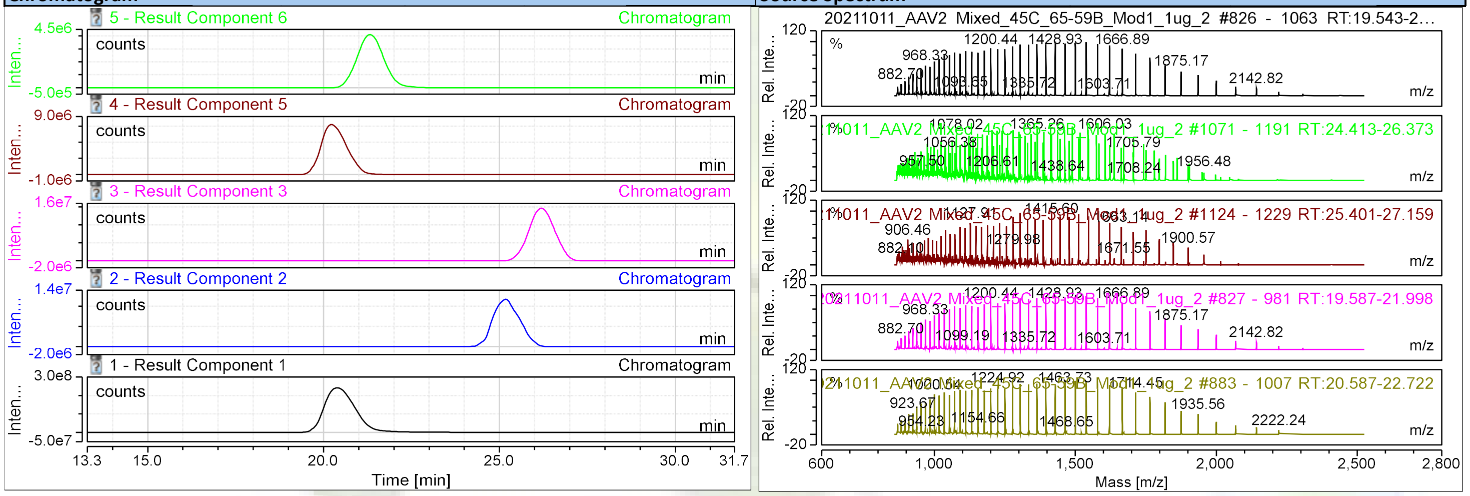

Deconvoluted Spectrum

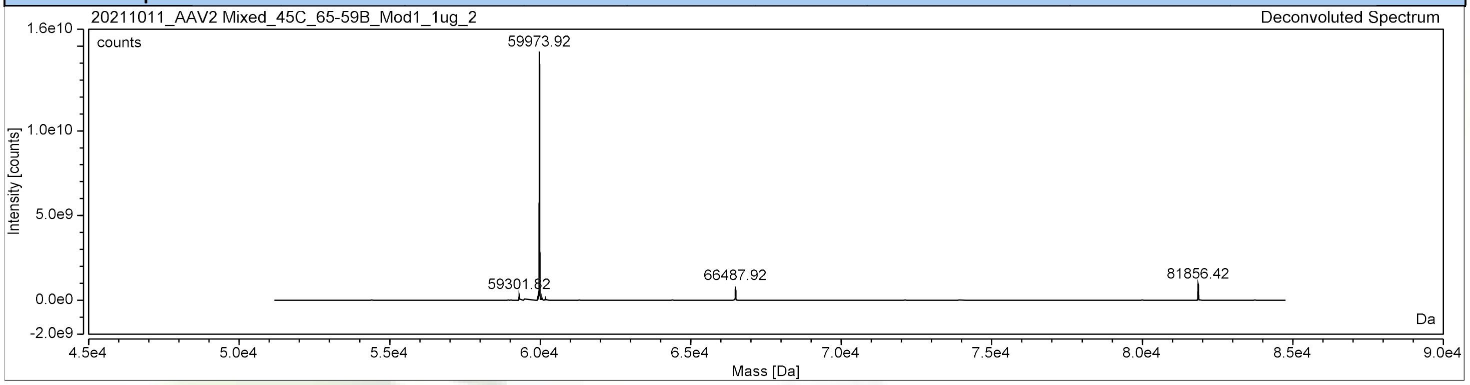

Deconvolution Results

Result Component	RT min	Mass Da	Std. Dev. Da	Std. Dev. ppm	Delta Mass Da	Intensity counts	Fractional Abundance counts	Rel. Abundance %	Number of Charge States	Score
Result Component 1	18.11	59733.033	0.40	6.68	0.000	27034264760	80.1	100.00	45	238.14
Result Component 2	18.22	59820.302	0.95	15.91	87.269	1527586438	4.5	5.65	40	170.79
Result Component 3	23.18	81291.415	0.91	11.13	21558.382	643910272	1.9	2.38	56	291.15
Result Component 4	19.20	59813.944	0.85	14.29	80.911	471832605	1.4	1.75	43	234.57
Result Component 5	18.27	59686.960	1.22	20.37	-46.073	390164875	1.2	1.44	32	176.14
Result Component 7	24.10	66210.268	0.85	12.85	6477.235	340089971	1.0	1.26	44	239.41
Result Component 8	21.47	59731.938	0.90	15.10	-1.095	300593658	0.9	1.11	41	217.18
Result Component 9	13.31	59733.070	0.64	10.75	0.037	298107673	0.9	1.10	42	257.50
Result Component 11	19.18	59933.627	1.10	18.41	200.594	200302659	0.6	0.74	40	199.85
Result Component 13	17.92	59687.388	1.55	25.99	-45.645	162773683	0.5	0.60	31	170.85
Result Component 15	21.21	59731.785	0.70	11.70	-1.249	129722977	0.4	0.48	41	225.58
Result Component 17	19.13	59766.513	1.08	18.11	33.479	91587642	0.3	0.34	38	197.64
Result Component 18	23.54	81372.238	1.92	23.54	21639.205	84227912	0.2	0.31	50	158.32
Result Component 19	18.85	59816.211	1.65	27.63	83.178	73795145	0.2	0.27	39	170.82
Result Component 20	19.45	59690.811	1.05	17.65	-42.222	72092544	0.2	0.27	37	195.30
Result Component 21	24.66	66290.558	1.28	19.26	6557.525	43412692	0.1	0.16	41	172.17

© 2009-2020 Thermo Fisher Scientific Inc. All rights reserved.
Chromeleon 7.3.0.60919

Component Summary

Injection Details

Injection Name:	**20211011_AAV2 Mixed_45C_65-59B_Mod1**	*Injection Volume:*	**8.45**
Vial Number:	**R:A9**	*Instrument Method:*	**AAV Cap_Prot_Sep_BEH Glycoprotein Amide HILIC_1x150mm**
Injection Type:	**Unknown**	*Processing Method:*	**MS Quantitative_45C_65-59B**
Run Time (min):	**44.98**	*Injection Date/Time:*	**12/Oct/21 18:59**

Peak Name	Retention Time min	Area counts*min	Relative Area %	Quantitation Ions m/z	Mol.Mass	Mass Confirmation Pass/Fail
AAV2_VP3	20.453	2637005.867	75.85	1428.955,1500.355,1463.789,1395.748,1364.055,1538.792	59973.780	**PASS**
AAV2_VP1	25.142	91155.345	2.62	1388.434,1545.445,1489.336,1342.917,1365.283,1412.362	81854.900	**PASS**
AAV2_VP2	26.152	124930.221	3.59	1415.636,1446.415,1255.530,1478.506,1279.609,1304.686	66487.900	**PASS**
			**82.07**			

Component Name: AAV5_VP3

Quantitation ions:	1525.669,1565.784,1608.079,1451.304,1487.553,1416.767
Retention time (min):	14.53
Expected Mass:	59462.370
Area (count*min):	-
Intensity (counts):	-
Amount:	-

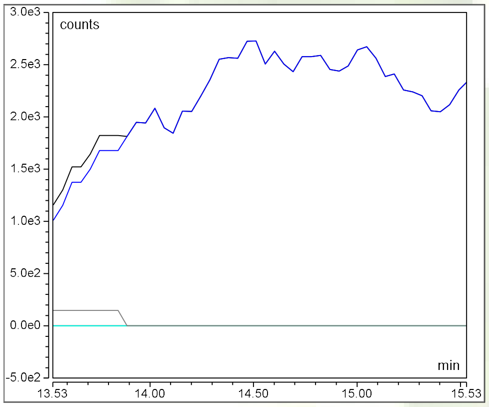

© 2009-2020 Thermo Fisher Scientific Inc. All rights reserved.
Chromeleon 7.3.0.60919

Component Name: AAV8_VP3

Quantitation ions:	1574.800,1496.109,1534.442,1459.645,1273.445,1391.802
Retention time (min):	15.73
Expected Mass:	59804.680
Area (count*min):	-
Intensity (counts):	-
Amount:	-

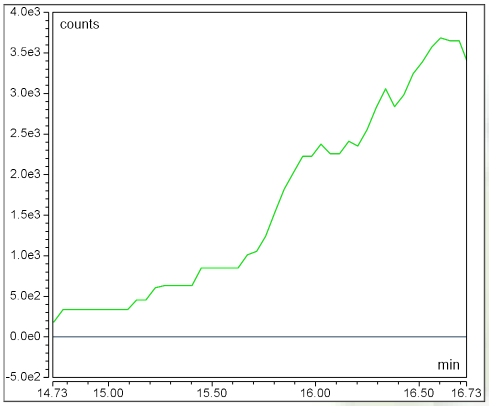

© 2009-2020 Thermo Fisher Scientific Inc. All rights reserved.
Chromeleon 7.3.0.60919

Component Name: AAV9_VP3

Quantitation ions:	1358.581,1390.150,1299.556,1328.415,1271.930,1457.907
Retention time (min):	18.15
Expected Mass:	59732.620
Area (count*min):	-
Intensity (counts):	-
Amount:	-

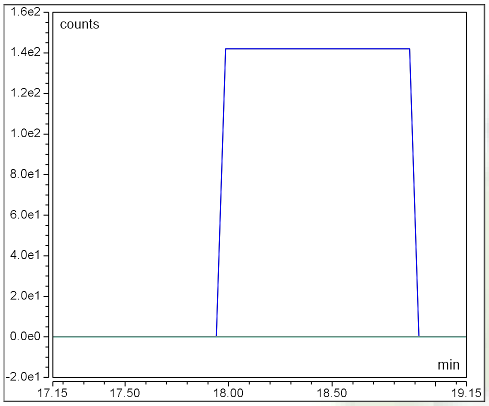

© 2009-2020 Thermo Fisher Scientific Inc. All rights reserved.
Chromeleon 7.3.0.60919

Component Name:	AAV2_VP3
Quantitation ions:	1428.955,1500.355,1463.789,1395.748,1364.055,1538.792
Retention time (min):	20.38
Expected Mass:	59973.780
Area (count*min):	2637006
Intensity (counts):	6113568
Amount:	-

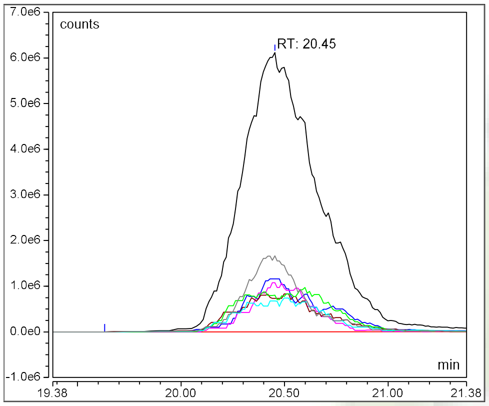

© 2009-2020 Thermo Fisher Scientific Inc. All rights reserved.
Chromeleon 7.3.0.60919

Component Name:	AAV9_VP1
Quantitation ions:	1378.820,1333.651,1355.868,1402.584,1534.808,1452.636
Retention time (min):	23.16
Expected Mass:	81290.340
Area (count*min):	-
Intensity (counts):	-
Amount:	-

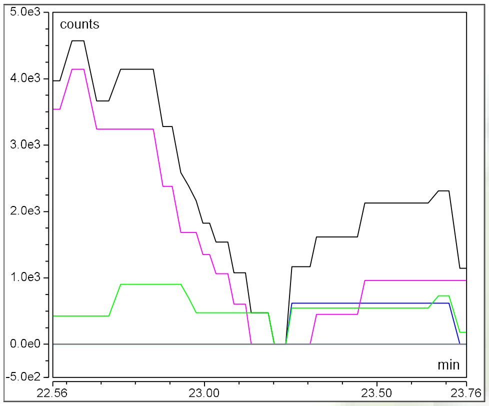

© 2009-2020 Thermo Fisher Scientific Inc. All rights reserved.
Chromeleon 7.3.0.60919

Component Name: AAV8_VP1

Quantitation ions:	1409.080,1459.382,1385.209,1513.388,1184.605,1339.828
Retention time (min):	23.33
Expected Mass:	81666.710
Area (count*min):	-
Intensity (counts):	-
Amount:	-

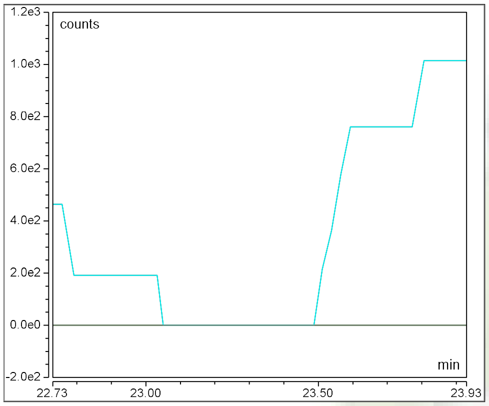

© 2009-2020 Thermo Fisher Scientific Inc. All rights reserved.
Chromeleon 7.3.0.60919

Component Name:	AAV5_VP1
Quantitation ions:	1362.628,1410.415,1435.577,1317.996,1339.949,1488.697
Retention time (min):	24.09
Expected Mass:	80335.080
Area (count*min):	-
Intensity (counts):	-
Amount:	-

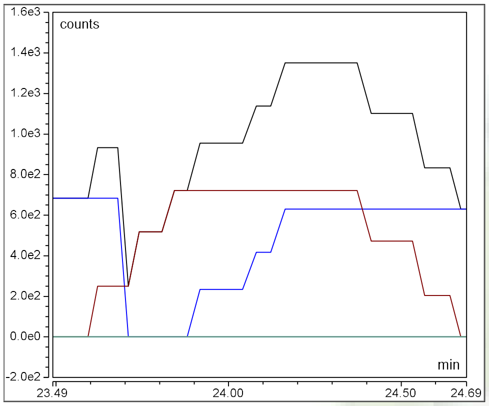

© 2009-2020 Thermo Fisher Scientific Inc. All rights reserved.
Chromeleon 7.3.0.60919

Component Name:	AAV9_VP2
Quantitation ions:	1409.730,1162.589,1472.334,1274.287,1380.371,1352.231
Retention time (min):	24.10
Expected Mass:	66209.760
Area (count*min):	-
Intensity (counts):	-
Amount:	-

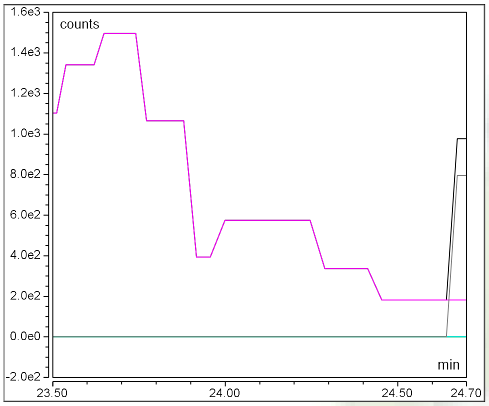

© 2009-2020 Thermo Fisher Scientific Inc. All rights reserved.
Chromeleon 7.3.0.60919

Component Name: AAV8_VP2

Quantitation ions:	1386.809,1358.524,1331.393,1305.308,1416.294,1623.397
Retention time (min):	24.50
Expected Mass:	66518.100
Area (count*min):	-
Intensity (counts):	-
Amount:	-

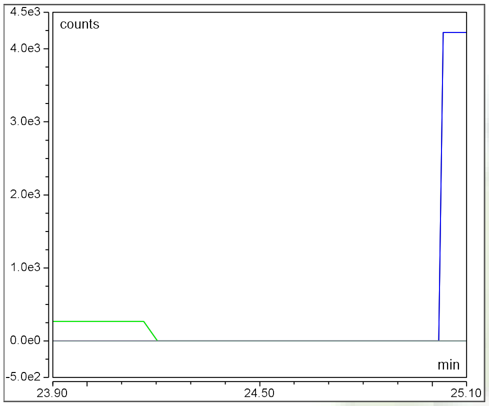

© 2009-2020 Thermo Fisher Scientific Inc. All rights reserved.
Chromeleon 7.3.0.60919

Component Name: AAV5_VP2

Quantitation ions:	1389.997,1519.197,1484.692,1420.196,1555.348,1451.727
Retention time (min):	25.16
Expected Mass:	65282.690
Area (count*min):	-
Intensity (counts):	-
Amount:	-

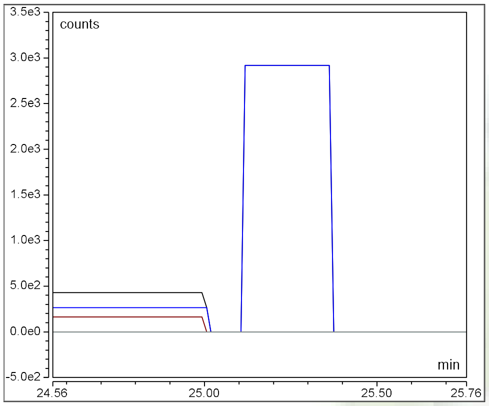

© 2009-2020 Thermo Fisher Scientific Inc. All rights reserved.
Chromeleon 7.3.0.60919

Component Name:	AAV2_VP1
Quantitation ions:	1388.434,1545.445,1489.336,1342.917,1365.283,1412.362
Retention time (min):	25.18
Expected Mass:	81854.900
Area (count*min):	91155
Intensity (counts):	235208
Amount:	-

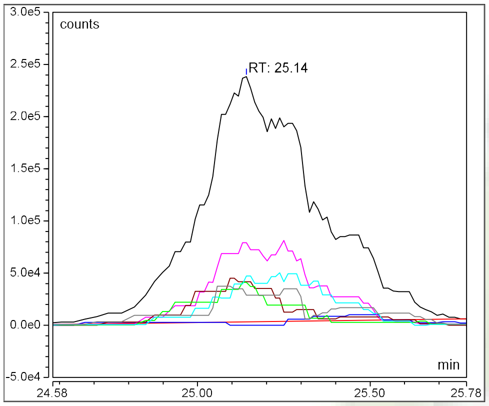

© 2009-2020 Thermo Fisher Scientific Inc. All rights reserved.
Chromeleon 7.3.0.60919

Component Name: AAV2_VP2	
Quantitation ions:	1415.636,1446.415,1255.530,1478.506,1279.609,1304.686
Retention time (min):	26.20
Expected Mass:	66487.900
Area (count*min):	124930
Intensity (counts):	318436
Amount:	-

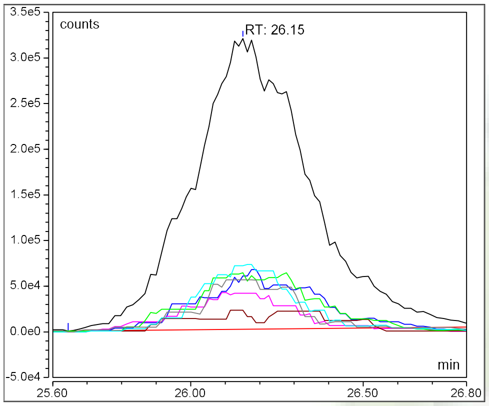

© 2009-2020 Thermo Fisher Scientific Inc. All rights reserved.
Chromeleon 7.3.0.60919

Chromatogram and SST Results

Injection Details

Injection Name:	**20211011_AAV2 Mixed_45C_65-59B_Mod1_1ug_2**	*Run Time (min):*	**44.98**
Vial Number:	**R:A9**	*Injection Volume:*	**8.45**
Injection Type:	**Unknown**		
Calibration Level:			
Instrument Method:	**AAV Cap_Prot_Sep_BEH Glycoprotein Amide HILIC_1x150mm_FLR + MS_CT45C_65-59_S**		
Processing Method:	**MS Quantitative_45C_65-59B**	*Dilution Factor:*	**1.0000**
Injection Date/Time:	**12/Oct/21 18:59**	*Sample Weight:*	**1.0000**

Chromatogram

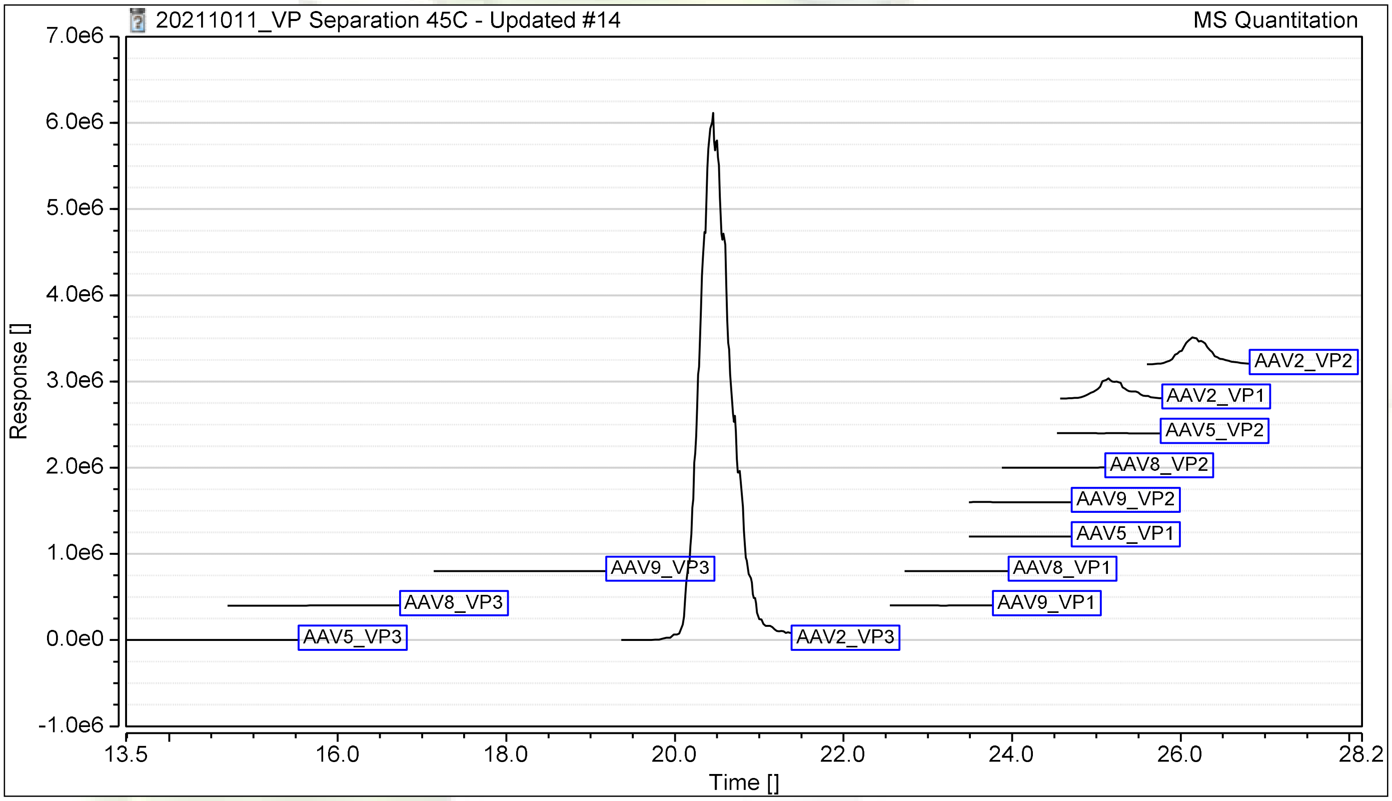

SST Results

No.	Name	Inj.Condition	Peak	Test Result	Injection
**Number of executed test cases:**	**n.a.**		**Total Result:**	**Passed**	

© 2009-2020 Thermo Fisher Scientific Inc. All rights reserved.

Protein Deconvolution Summary

Injection Details

Injection Name:	**20211011_AAV2 Mixed_45C_65-59B_Mod1_1ug_3**	*Injection Volume:*	**8.45**
Vial Number:	**R:A9**	*Instrument Method:*	**AAV Cap_Prot_Sep_BEH Glycoprotein Amide H**
Injection Type:	**Unknown**	*Processing Method:*	**MS Quantitative_45C_65-59B**
Run Time (min):	**44.99**	*Injection Date/Time:*	**12/Oct/21 19:45**

Chromatogram

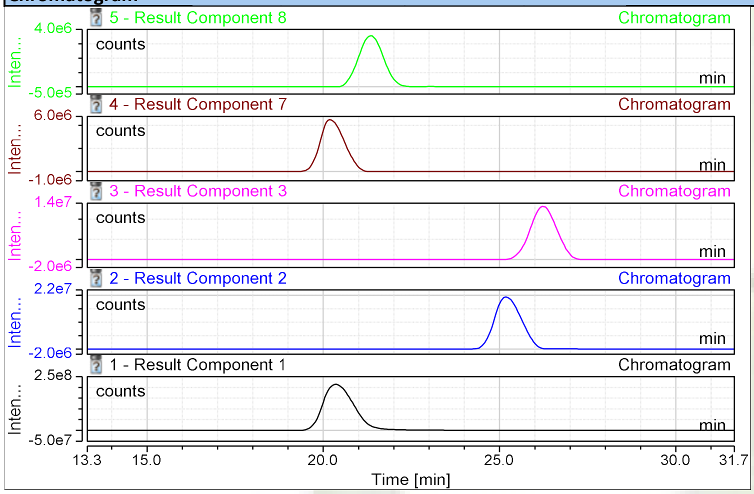

Source Spectrum

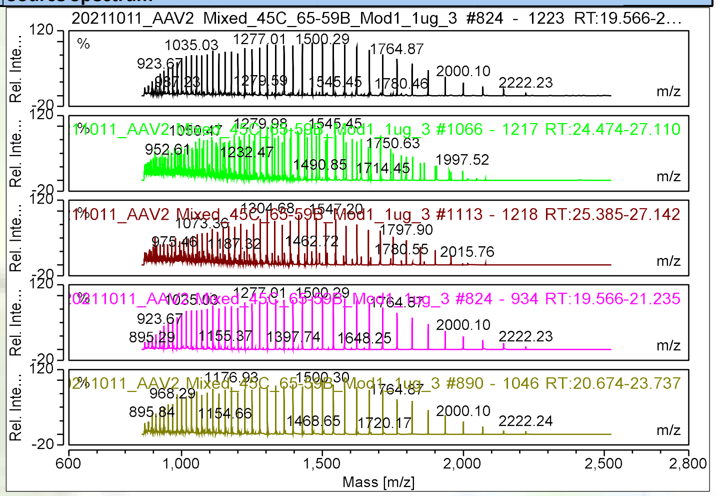

Deconvoluted Spectrum

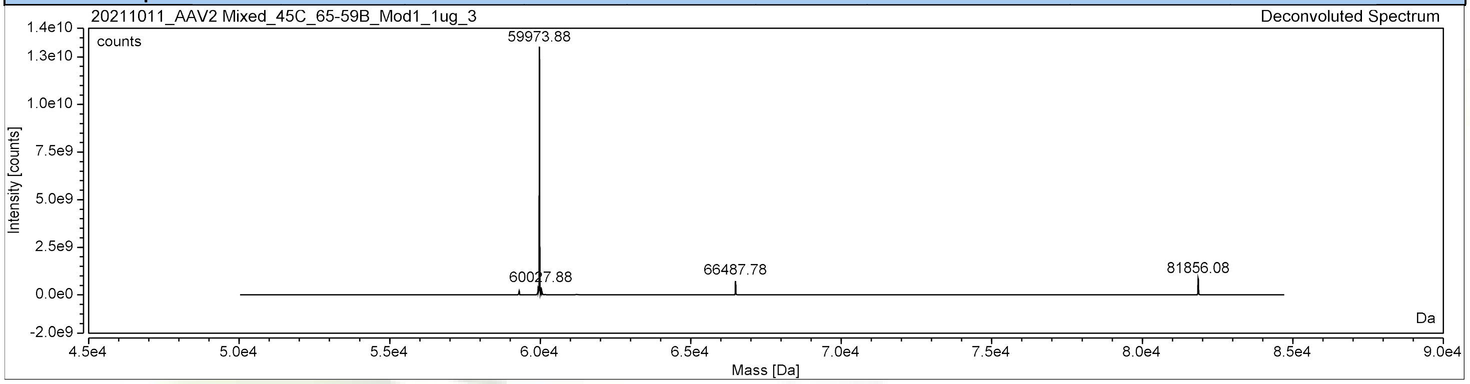

Deconvolution Results

Result Component	RT min	Mass Da	Std. Dev. Da	Std. Dev. ppm	Delta Mass Da	Intensity counts	Fractional Abundance counts	Rel. Abundance %	Number of Charge States	Score
Result Component 1	18.11	59733.033	0.40	6.68	0.000	27034264760	80.1	100.00	45	238.14
Result Component 2	18.22	59820.302	0.95	15.91	87.269	1527586438	4.5	5.65	40	170.79
Result Component 3	23.18	81291.415	0.91	11.13	21558.382	643910272	1.9	2.38	56	291.15
Result Component 4	19.20	59813.944	0.85	14.29	80.911	471832605	1.4	1.75	43	234.57
Result Component 5	18.27	59686.960	1.22	20.37	-46.073	390164875	1.2	1.44	32	176.14
Result Component 7	24.10	66210.268	0.85	12.85	6477.235	340089971	1.0	1.26	44	239.41
Result Component 8	21.47	59731.938	0.90	15.10	-1.095	300593658	0.9	1.11	41	217.18
Result Component 9	13.31	59733.070	0.64	10.75	0.037	298107673	0.9	1.10	42	257.50
Result Component 11	19.18	59933.627	1.10	18.41	200.594	200302659	0.6	0.74	40	199.85
Result Component 13	17.92	59687.388	1.55	25.99	-45.645	162773683	0.5	0.60	31	170.85
Result Component 15	21.21	59731.785	0.70	11.70	-1.249	129722977	0.4	0.48	41	225.58
Result Component 17	19.13	59766.513	1.08	18.11	33.479	91587642	0.3	0.34	38	197.64
Result Component 18	23.54	81372.238	1.92	23.54	21639.205	84227912	0.2	0.31	50	158.32
Result Component 19	18.85	59816.211	1.65	27.63	83.178	73795145	0.2	0.27	39	170.82
Result Component 20	19.45	59690.811	1.05	17.65	-42.222	72092544	0.2	0.27	37	195.30
Result Component 21	24.66	66290.558	1.28	19.26	6557.525	43412692	0.1	0.16	41	172.17

© 2009-2020 Thermo Fisher Scientific Inc. All rights reserved.
Chromeleon 7.3.0.60919

Component Summary

Injection Details

Injection Name:	**20211011_AAV2 Mixed_45C_65-59B_Mod1**	*Injection Volume:*	**8.45**
Vial Number:	**R:A9**	*Instrument Method:*	**AAV Cap_Prot_Sep_BEH Glycoprotein Amide HILIC_1x150mm**
Injection Type:	**Unknown**	*Processing Method:*	**MS Quantitative_45C_65-59B**
Run Time (min):	**44.99**	*Injection Date/Time:*	**12/Oct/21 19:45**

Peak Name	Retention Time min	Area counts*min	Relative Area %	Quantitation Ions m/z	Mol.Mass	Mass Confirmation Pass/Fail
AAV2_VP3	20.403	2673211.186	78.52	1428.955,1500.355,1463.789,1395.748,1364.055,1538.792	59973.780	**PASS**
AAV2_VP1	25.250	107448.559	3.16	1388.434,1545.445,1489.336,1342.917,1365.283,1412.362	81854.900	**PASS**
AAV2_VP2	26.172	104060.303	3.06	1415.636,1446.415,1255.530,1478.506,1279.609,1304.686	66487.900	**PASS**
			**84.73**			

Component Name: AAV5_VP3

Quantitation ions:	1525.669,1565.784,1608.079,1451.304,1487.553,1416.767
Retention time (min):	14.53
Expected Mass:	59462.370
Area (count*min):	-
Intensity (counts):	-
Amount:	-

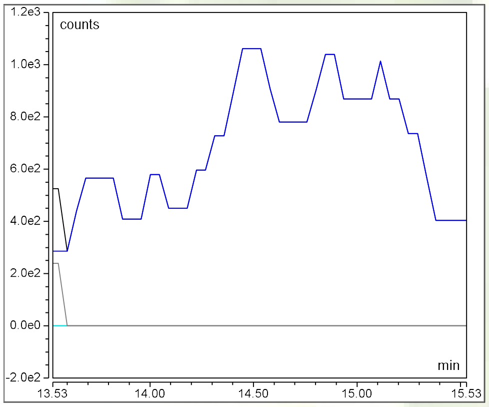

© 2009-2020 Thermo Fisher Scientific Inc. All rights reserved.
Chromeleon 7.3.0.60919

Component Name:	AAV8_VP3
Quantitation ions:	1574.800,1496.109,1534.442,1459.645,1273.445,1391.802
Retention time (min):	15.73
Expected Mass:	59804.680
Area (count*min):	-
Intensity (counts):	-
Amount:	-

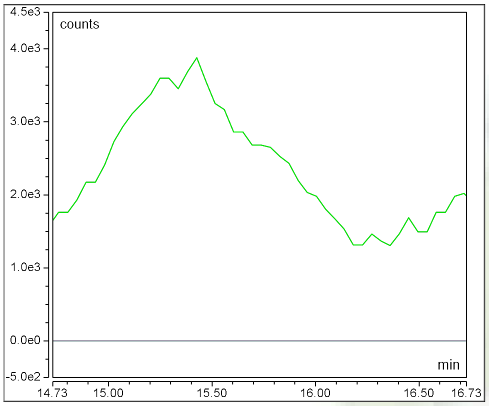

© 2009-2020 Thermo Fisher Scientific Inc. All rights reserved.
Chromeleon 7.3.0.60919

Component Name:	AAV9_VP3
Quantitation ions:	1358.581,1390.150,1299.556,1328.415,1271.930,1457.907
Retention time (min):	18.15
Expected Mass:	59732.620
Area (count*min):	-
Intensity (counts):	-
Amount:	-

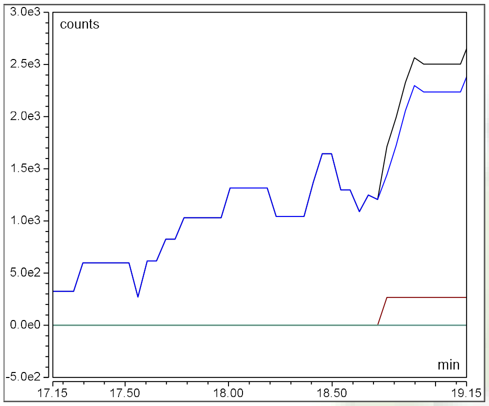

© 2009-2020 Thermo Fisher Scientific Inc. All rights reserved.
Chromeleon 7.3.0.60919

Component Name:	AAV2_VP3
Quantitation ions:	1428.955,1500.355,1463.789,1395.748,1364.055,1538.792
Retention time (min):	20.38
Expected Mass:	59973.780
Area (count*min):	2673211
Intensity (counts):	5948923
Amount:	-

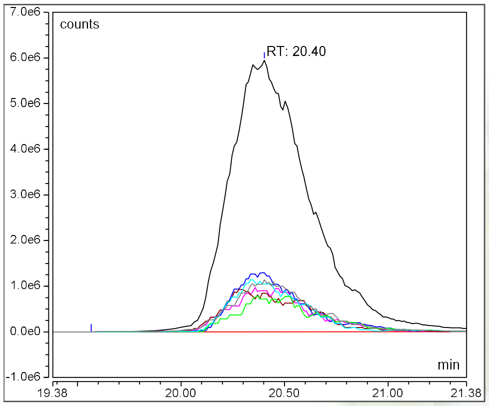

© 2009-2020 Thermo Fisher Scientific Inc. All rights reserved.
Chromeleon 7.3.0.60919

Component Name:	AAV9_VP1
Quantitation ions:	1378.820,1333.651,1355.868,1402.584,1534.808,1452.636
Retention time (min):	23.16
Expected Mass:	81290.340
Area (count*min):	-
Intensity (counts):	-
Amount:	-

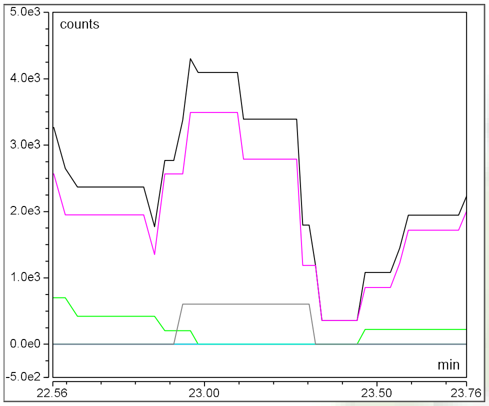

© 2009-2020 Thermo Fisher Scientific Inc. All rights reserved.
Chromeleon 7.3.0.60919

Component Name:	AAV8_VP1
Quantitation ions:	1409.080,1459.382,1385.209,1513.388,1184.605,1339.828
Retention time (min):	23.33
Expected Mass:	81666.710
Area (count*min):	-
Intensity (counts):	-
Amount:	-

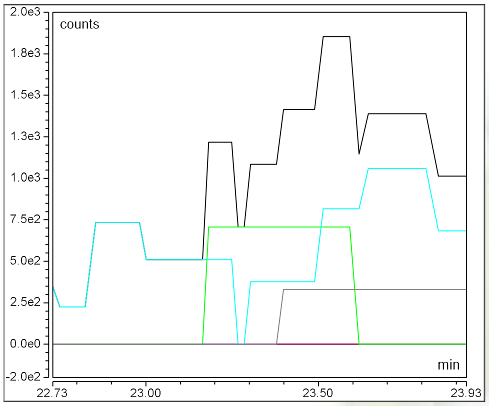

© 2009-2020 Thermo Fisher Scientific Inc. All rights reserved.
Chromeleon 7.3.0.60919

Component Name:	AAV5_VP1
Quantitation ions:	1362.628,1410.415,1435.577,1317.996,1339.949,1488.697
Retention time (min):	24.09
Expected Mass:	80335.080
Area (count*min):	-
Intensity (counts):	-
Amount:	-

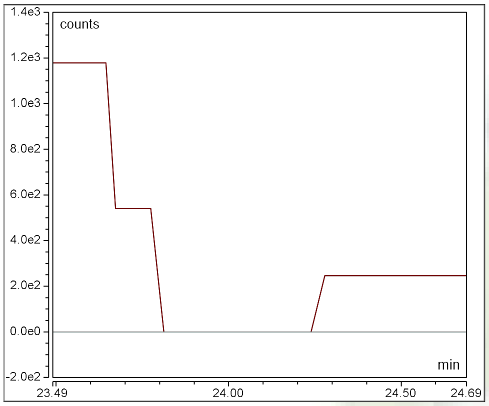

© 2009-2020 Thermo Fisher Scientific Inc. All rights reserved.
Chromeleon 7.3.0.60919

Component Name:	AAV9_VP2
Quantitation ions:	1409.730,1162.589,1472.334,1274.287,1380.371,1352.231
Retention time (min):	24.10
Expected Mass:	66209.760
Area (count*min):	-
Intensity (counts):	-
Amount:	-

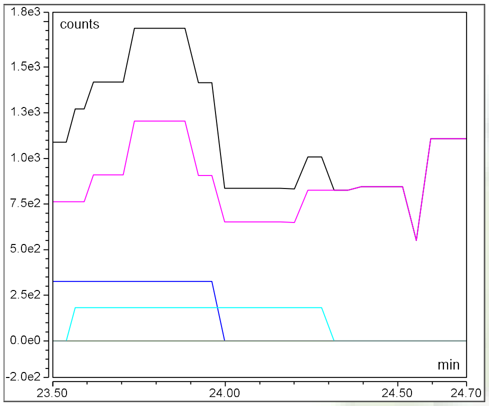

© 2009-2020 Thermo Fisher Scientific Inc. All rights reserved.
Chromeleon 7.3.0.60919

Component Name: AAV8_VP2

Quantitation ions:	1386.809,1358.524,1331.393,1305.308,1416.294,1623.397
Retention time (min):	24.50
Expected Mass:	66518.100
Area (count*min):	-
Intensity (counts):	-
Amount:	-

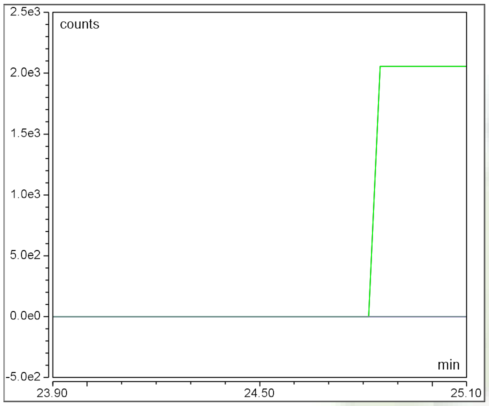

© 2009-2020 Thermo Fisher Scientific Inc. All rights reserved.
Chromeleon 7.3.0.60919

Component Name:	AAV5_VP2
Quantitation ions:	1389.997,1519.197,1484.692,1420.196,1555.348,1451.727
Retention time (min):	25.16
Expected Mass:	65282.690
Area (count*min):	-
Intensity (counts):	-
Amount:	-

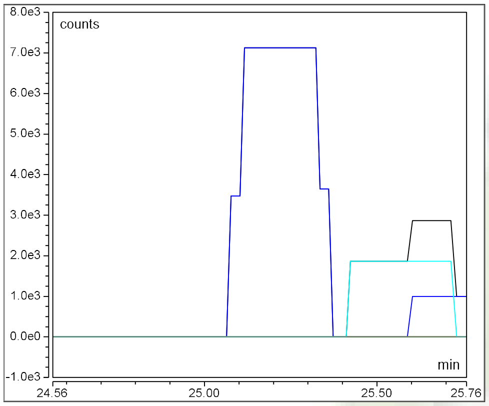

© 2009-2020 Thermo Fisher Scientific Inc. All rights reserved.
Chromeleon 7.3.0.60919

Component Name:	AAV2_VP1
Quantitation ions:	1388.434,1545.445,1489.336,1342.917,1365.283,1412.362
Retention time (min):	25.18
Expected Mass:	81854.900
Area (count*min):	107449
Intensity (counts):	272716
Amount:	-

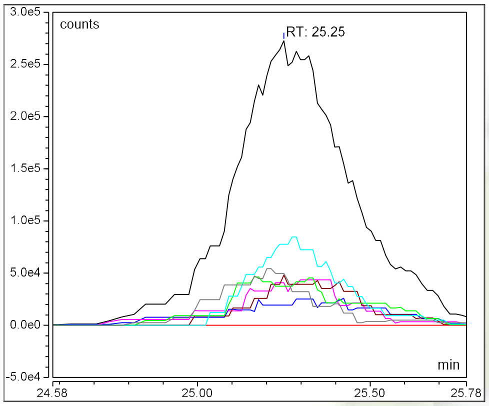

© 2009-2020 Thermo Fisher Scientific Inc. All rights reserved.
Chromeleon 7.3.0.60919

Component Name: AAV2_VP2

Quantitation ions:	1415.636,1446.415,1255.530,1478.506,1279.609,1304.686
Retention time (min):	26.20
Expected Mass:	66487.900
Area (count*min):	104060
Intensity (counts):	217576
Amount:	-

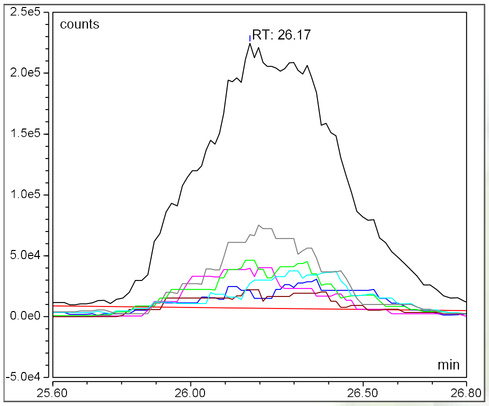

© 2009-2020 Thermo Fisher Scientific Inc. All rights reserved.
Chromeleon 7.3.0.60919

Chromatogram and SST Results

Injection Details

Injection Name:	**20211011_AAV2 Mixed_45C_65-59B_Mod1_1ug_3**	*Run Time (min):*	**44.99**
Vial Number:	**R:A9**	*Injection Volume:*	**8.45**
Injection Type:	**Unknown**		
Calibration Level:			
Instrument Method:	**AAV Cap_Prot_Sep_BEH Glycoprotein Amide HILIC_1x150mm_FLR + MS_CT45C_65-59_S**		
Processing Method:	**MS Quantitative_45C_65-59B**	*Dilution Factor:*	**1.0000**
Injection Date/Time:	**12/Oct/21 19:45**	*Sample Weight:*	**1.0000**

Chromatogram

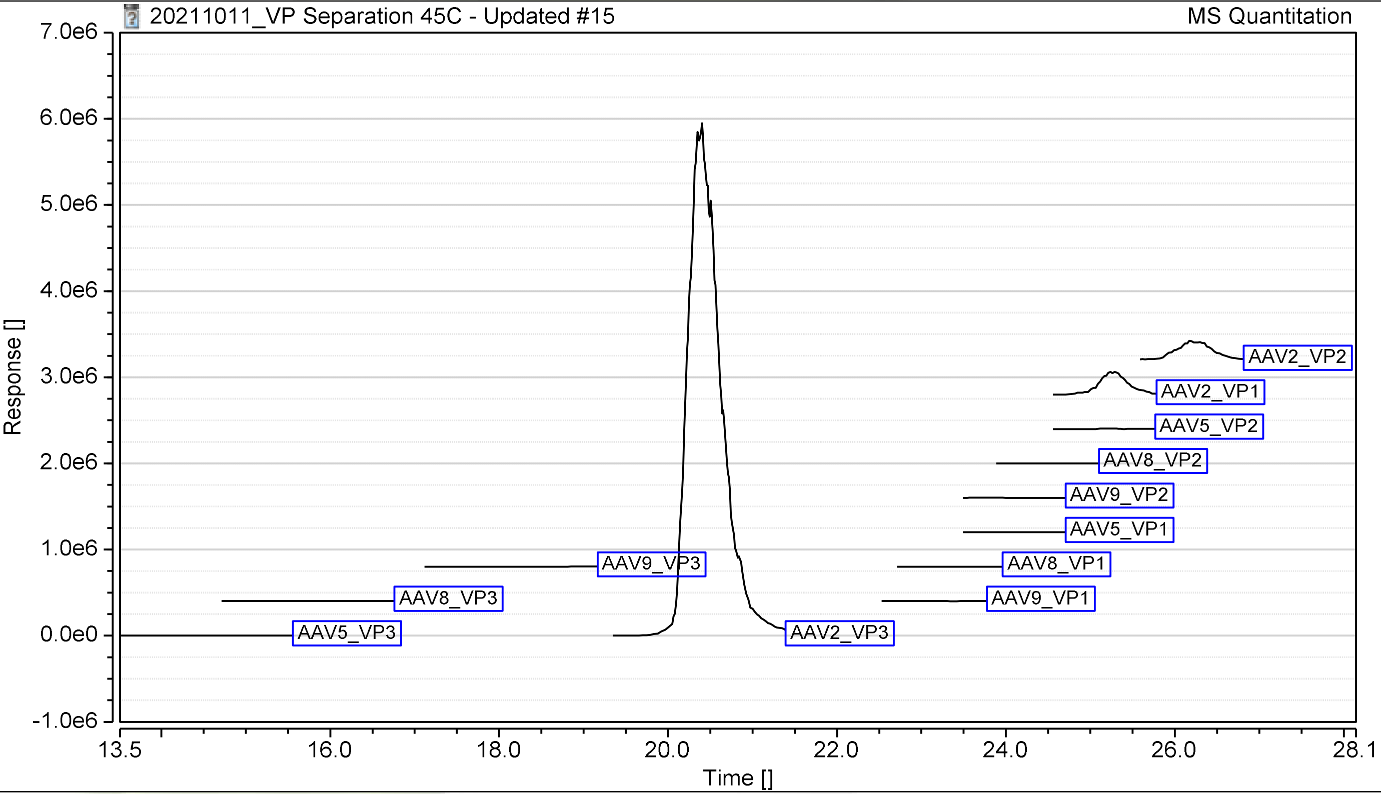

SST Results

No.	Name	Inj.Condition	Peak	Test Result	Injection
**Number of executed test cases:**	**n.a.**		**Total Result:**	**Passed**	

© 2009-2020 Thermo Fisher Scientific Inc. All rights reserved.
Chromeleon 7.3.0.60919

Supplementary Report 2:

Report of PTM monitoring method applied to AAV2 generated in Chromeleon

Protein Deconvolution Summary

Injection Details

Injection Name:	**20220120_AAV2_HEK_VP Sep_25C_64.5-58.5B_45K Res_1ug_1**	*Injection Volume:*	**8.56**
Vial Number:	**R:A1**	*Instrument Method:*	**AAV Cap_Prot_Sep_BEH Glycoprotein Amide H**
Injection Type:	**Unknown**	*Processing Method:*	**AAV2 MS Quantitative In-depth Characterizatior**
Run Time (min):	**45.00**	*Injection Date/Time:*	**21/Jan/22 07:32**

Chromatogram Component 1

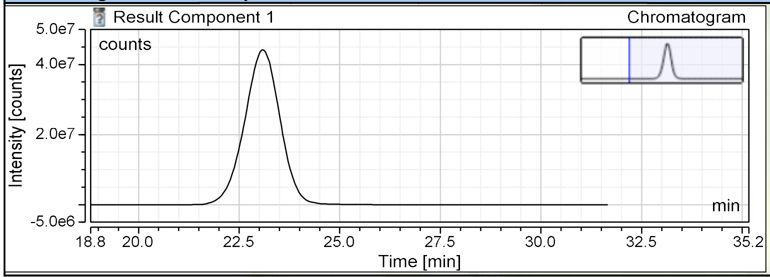

Source Spectrum Component 1

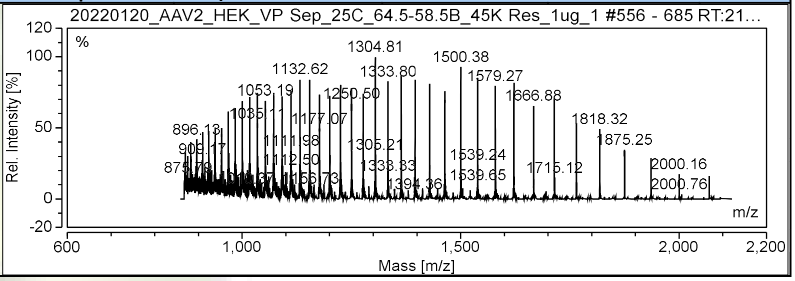

Chromatogram Component 2

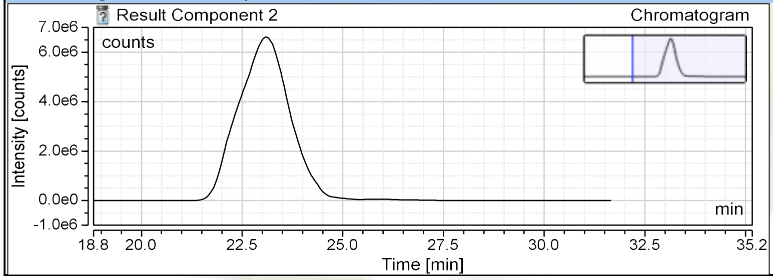

Source Spectrum Component 2

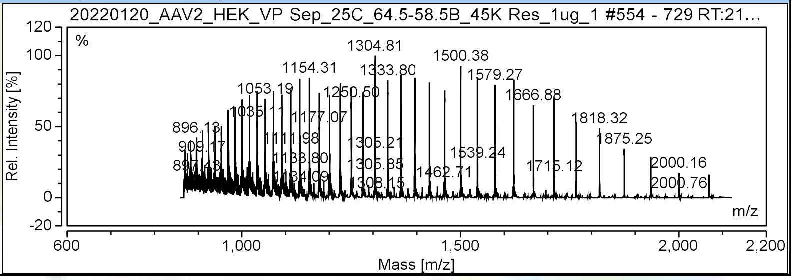

Chromatogram Component 3

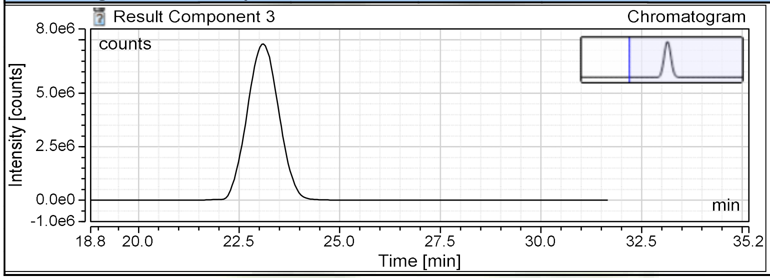

Source Spectrum Component 3

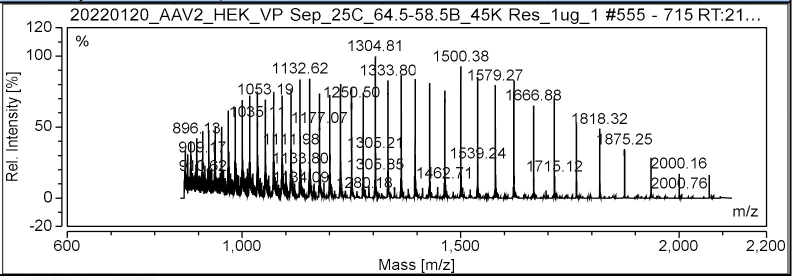

Chromatogram Component 4

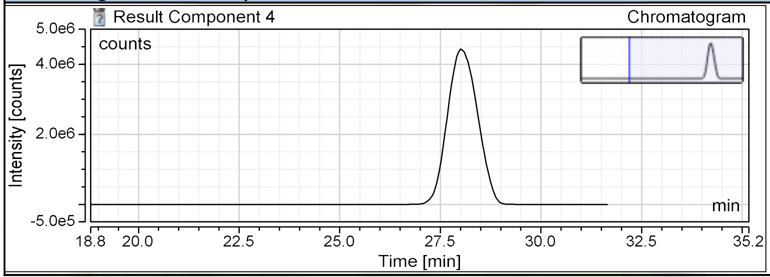

Source Spectrum Component 4

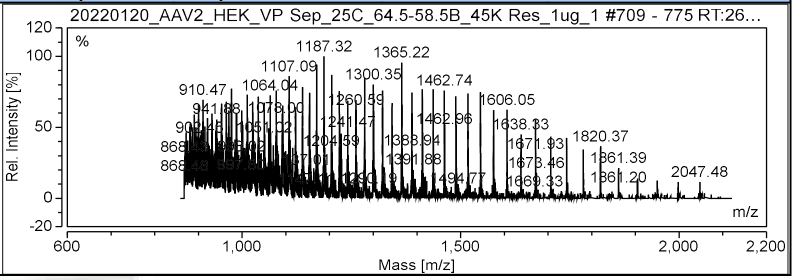

Chromatogram Component 5

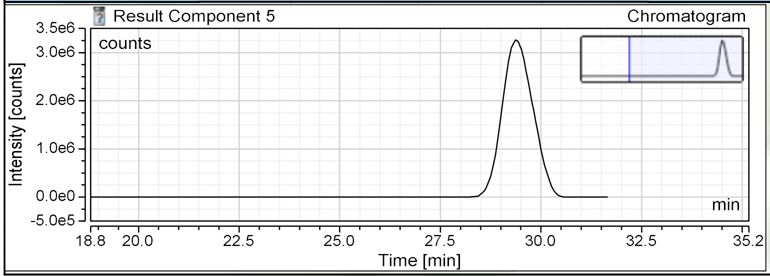

Source Spectrum Component 5

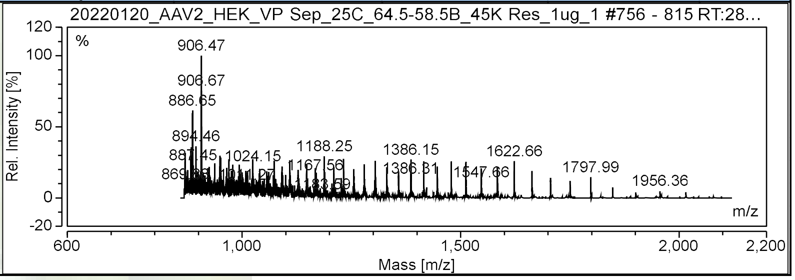

© 2009-2020 Thermo Fisher Scientific Inc. All rights reserved.
Chromeleon 7.3.0.60919

Chromatogram Component 7

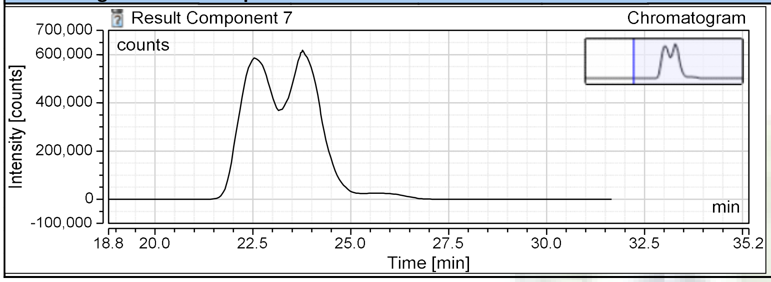

Source Spectrum Component 7

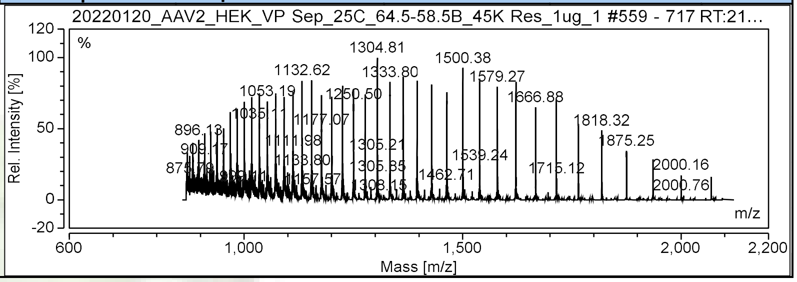

Chromatogram Component 9

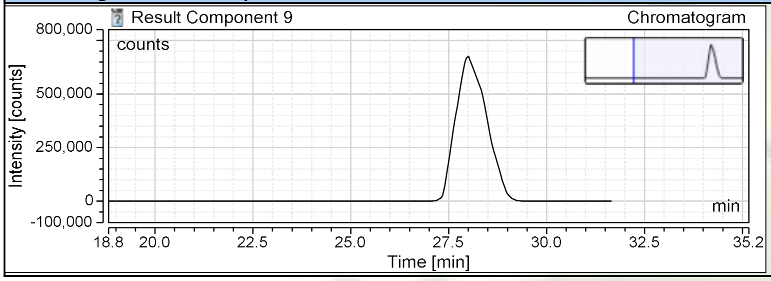

Source Spectrum Component 9

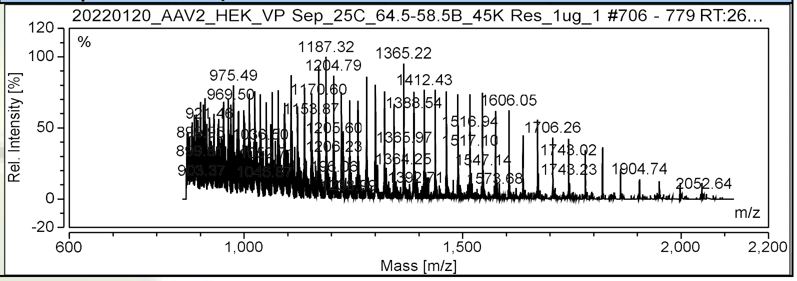

Chromatogram Component 12

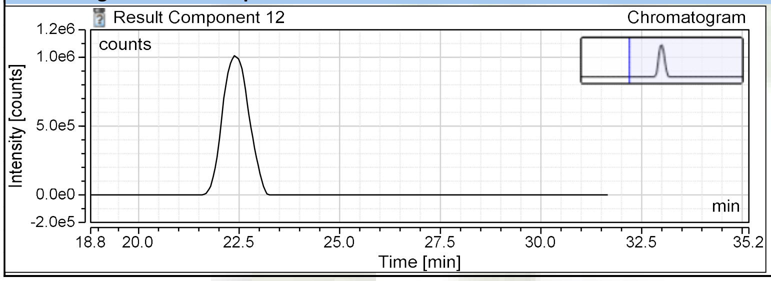

Source Spectrum Component 12

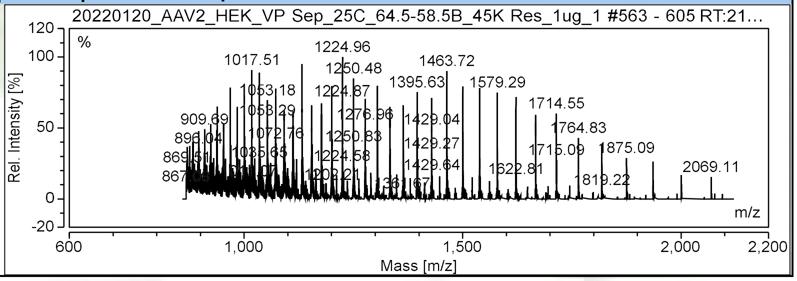

Chromatogram Component 20

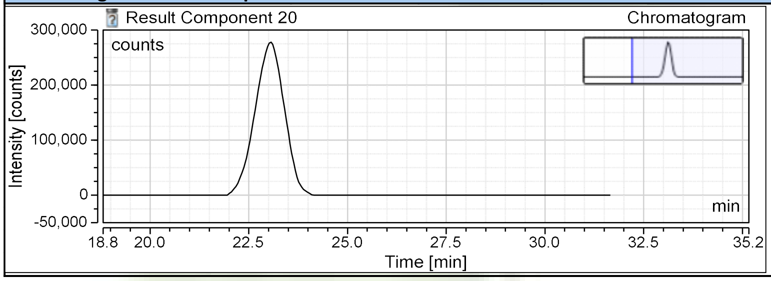

Source Spectrum Component 20

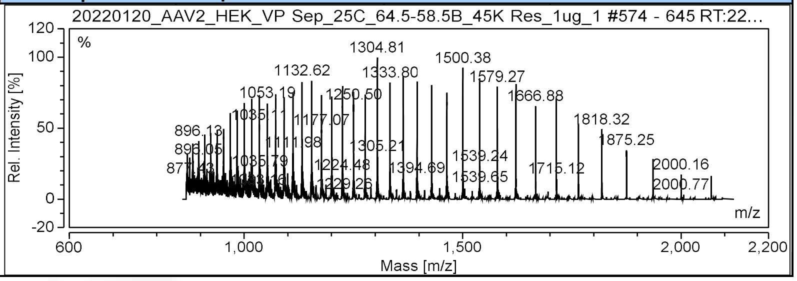

Deconvoluted Spectrum

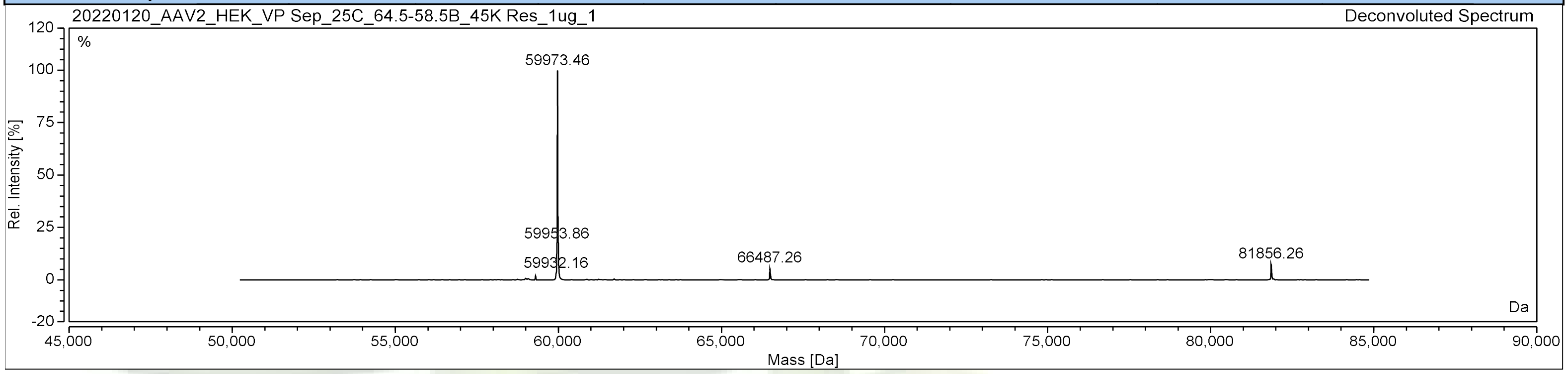

Deconvolution Results

Result Component	RT min	Mass Da	Std. Dev. Da	Std. Dev. ppm	Delta Mass Da	Intensity counts	Fractional Abundance counts	Rel. Abundance %	Number of Charge States	Score
Result Component 1	23.08	59973.436	0.84	13.99	0.000	1959279259	46.2	100.00	41	242.04
Result Component 2	23.11	59991.819	1.81	30.24	18.383	430877006	10.2	21.99	41	161.22
Result Component 3	23.08	59953.840	1.53	25.59	-19.596	302960887	7.1	15.46	34	154.28
Result Component 4	28.00	81855.271	1.70	20.79	21881.835	149335479	3.5	7.62	55	180.65
Result Component 5	29.37	66487.295	1.24	18.66	6513.860	115071618	2.7	5.87	42	195.77
Result Component 6	23.08	60018.234	1.74	28.98	44.799	61338748	1.4	3.13	25	68.82
Result Component 7	23.76	60006.121	1.86	30.94	32.686	59932437	1.4	3.06	39	79.35
Result Component 8	29.37	66504.638	1.95	29.34	6531.202	43476745	1.0	2.22	41	102.72
Result Component 9	27.98	81872.829	2.11	25.73	21899.393	38271649	0.9	1.95	44	90.47

Result Component 11	22.91	59299.871	1.48	25.01	-673.565	28850826	0.7	1.47	30	69.26
Result Component 12	22.40	59954.253	1.72	28.73	-19.183	27440115	0.6	1.40	33	139.46
Result Component 19	27.67	81874.453	2.73	33.39	21901.017	10900834	0.3	0.56	50	109.08
Result Component 77	24.00	60020.804	1.54	25.71	47.368	3356483	0.1	0.17	33	78.05
Result Component 152	29.15	66467.903	1.85	27.80	6494.467	1942465	0.0	0.10	19	65.67

© 2009-2020 Thermo Fisher Scientific Inc. All rights reserved.
Chromeleon 7.3.0.60919

Component Summary

Injection Details

Injection Name:	**20220120_AAV2_HEK_VP Sep_25C_64.5-5**	*Injection Volume:*	**8.56**
Vial Number:	**R:A1**	*Instrument Method:*	**AAV Cap_Prot_Sep_BEH Glycoprotein**
Injection Type:	**Unknown**	*Processing Method:*	**AAV2 MS Quantitative In-depth Charact**
Run Time (min):	**45.00**	*Injection Date/Time:*	**21/Jan/22 07:32**

Peak Name	Retention Time min	Area counts*min	Relative Area %	Quantitation Ions m/z	Mol.Mass	Mass Confirmation Pass/Fail
AAV2_VP3 Variant	22.934	44616.104	6.75	1447.374,957.488,1380.090,1163.736,1099.120,973.145	59301.030	PASS
AAV2_VP3[2xOxidation (MW)]	22.934	89046.902	13.47	1133.193,1092.027,1112.217,1072.520,1539.665,1464.606	60005.780	PASS
AAV2_VP3[1xSuccinimide D]	22.955	133805.141	20.24	952.674,923.374,968.020,937.789,1017.182,1000.250	59955.770	PASS
AAV2_VP3[1xOxidation (MW)]	23.079	125455.836	18.97	1277.428,1396.179,1464.229,1250.823,1305.183,1225.324	59989.780	PASS
AAV2_VP3	23.100	122079.437	18.46	1364.311,1500.570,1333.966,1395.925,1463.892,1225.374	59973.780	PASS
AAV2_VP1[1xOxidation (MW)]	28.073	34246.303	5.18	1343.191,1154.150,1388.700,1187.572,1412.610,1170.616	81870.890	PASS
AAV2_VP1	28.117	52640.275	7.96	1342.896,1388.388,1365.271,1300.301,1187.321,1279.998	81854.900	PASS
AAV2_VP2[1xOxidation (MW)]	29.331	24845.730	3.76	1446.760,1305.022,1167.738,1358.263,1147.628,1331.107	66503.900	PASS
AAV2_VP2	29.400	34429.457	5.21	1446.392,1415.628,1304.683,1478.503,1209.871,1386.167	66487.900	PASS
			**100.00**			

Component Name: AAV2_VP3 Variant

Quantitation ions:	1447.374,957.488,1380.090,1163.736,1099.120,973.145
Retention time (min):	22.89
Expected Mass:	59301.030
Area (count*min):	44616
Intensity (counts):	90177
Amount:	-

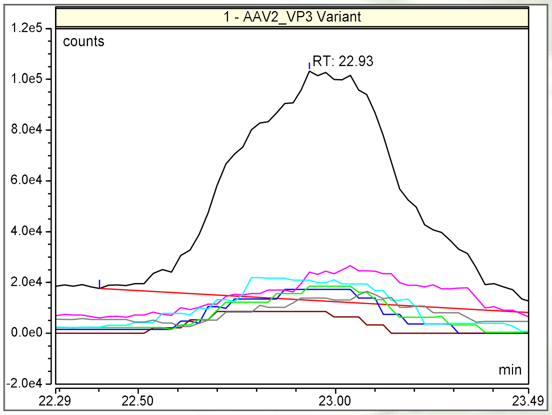

© 2009-2020 Thermo Fisher Scientific Inc. All rights reserved.
Chromeleon 7.3.0.60919

Component Name:	AAV2_VP3[1xSuccinimide D]
Quantitation ions:	952.674,923.374,968.020,937.789,1017.182,1000.250
Retention time (min):	23.09
Expected Mass:	59955.770
Area (count*min):	133805
Intensity (counts):	248069
Amount:	-

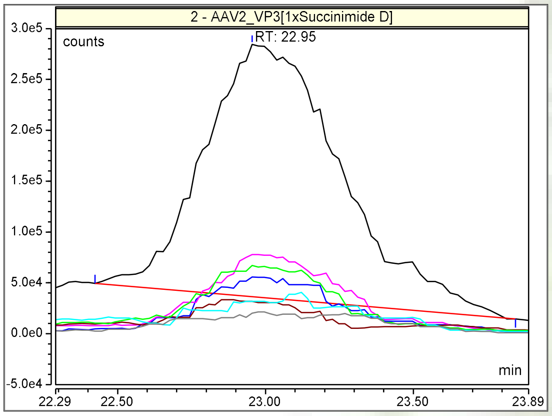

© 2009-2020 Thermo Fisher Scientific Inc. All rights reserved.
Chromeleon 7.3.0.60919

Component Name: AAV2_VP3	
Quantitation ions:	1364.311,1500.570,1333.966,1395.925,1463.892,1225.374
Retention time (min):	23.10
Expected Mass:	59973.780
Area (count*min):	122079
Intensity (counts):	183519
Amount:	-

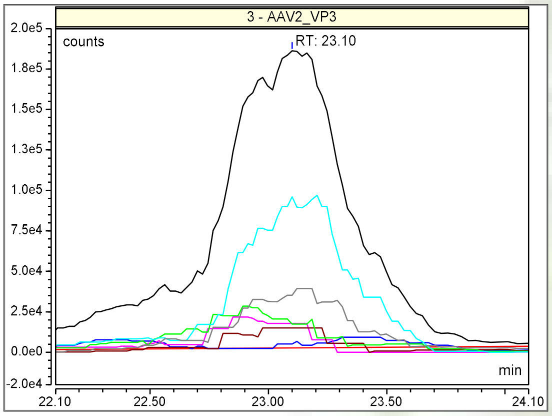

© 2009-2020 Thermo Fisher Scientific Inc. All rights reserved.
Chromeleon 7.3.0.60919

Component Name:	AAV2_VP3[2xOxidation (MW)]
Quantitation ions:	1133.193,1092.027,1112.217,1072.520,1539.665,1464.606
Retention time (min):	23.33
Expected Mass:	60005.780
Area (count*min):	89047
Intensity (counts):	85510
Amount:	-

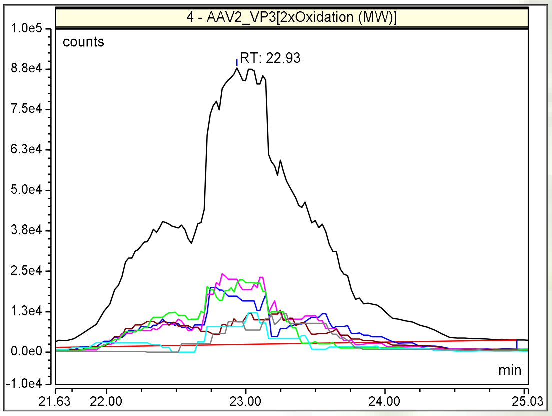

© 2009-2020 Thermo Fisher Scientific Inc. All rights reserved.
Chromeleon 7.3.0.60919

Component Name:	AAV2_VP3[1xOxidation (MW)]
Quantitation ions:	1277.428,1396.179,1464.229,1250.823,1305.183,1225.324
Retention time (min):	23.37
Expected Mass:	59989.780
Area (count*min):	125456
Intensity (counts):	139774
Amount:	-

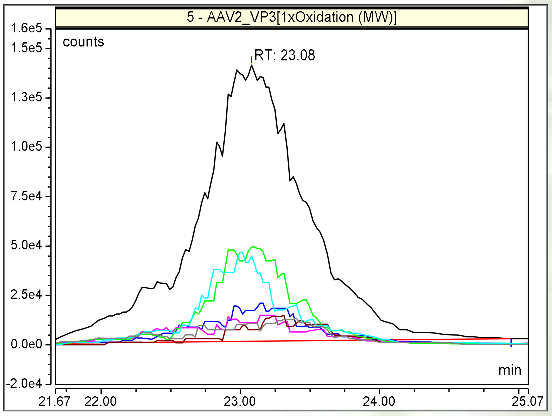

© 2009-2020 Thermo Fisher Scientific Inc. All rights reserved.
Chromeleon 7.3.0.60919

Component Name:	AAV2_VP1
Quantitation ions:	1342.896,1388.388,1365.271,1300.301,1187.321,1279.998
Retention time (min):	28.03
Expected Mass:	81854.900
Area (count*min):	52640
Intensity (counts):	87048
Amount:	-

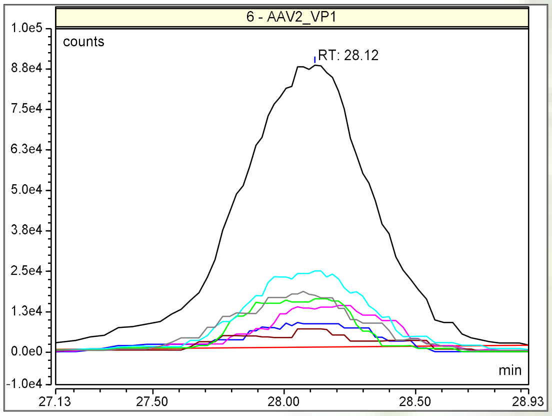

© 2009-2020 Thermo Fisher Scientific Inc. All rights reserved.
Chromeleon 7.3.0.60919

Component Name:	AAV2_VP1[1xOxidation (MW)]
Quantitation ions:	1343.191,1154.150,1388.700,1187.572,1412.610,1170.616
Retention time (min):	28.03
Expected Mass:	81870.890
Area (count*min):	34246
Intensity (counts):	46943
Amount:	-

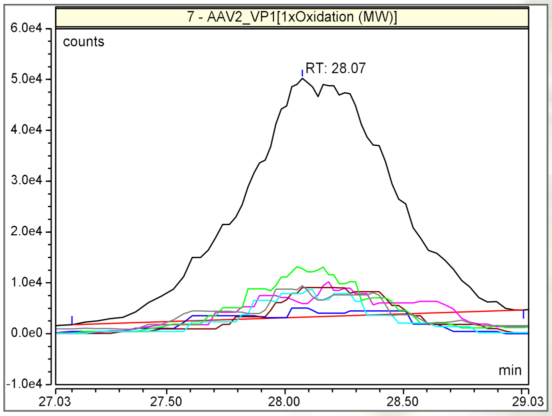

© 2009-2020 Thermo Fisher Scientific Inc. All rights reserved.
Chromeleon 7.3.0.60919

Component Name:	AAV2_VP2
Quantitation ions:	1446.392,1415.628,1304.683,1478.503,1209.871,1386.167
Retention time (min):	29.34
Expected Mass:	66487.900
Area (count*min):	34429
Intensity (counts):	58675
Amount:	-

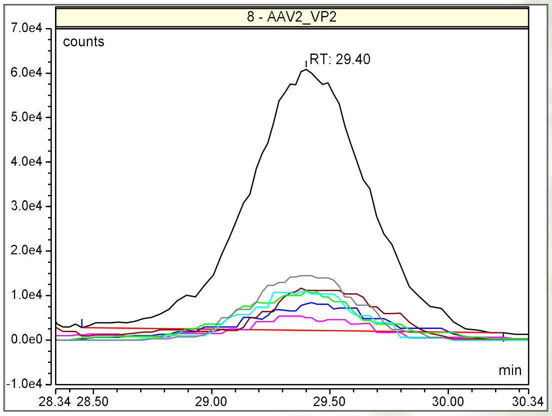

© 2009-2020 Thermo Fisher Scientific Inc. All rights reserved.
Chromeleon 7.3.0.60919

Component Name:	AAV2_VP2[1xOxidation (MW)]
Quantitation ions:	1446.760,1305.022,1167.738,1358.263,1147.628,1331.107
Retention time (min):	29.38
Expected Mass:	66503.900
Area (count*min):	24846
Intensity (counts):	33271
Amount:	-

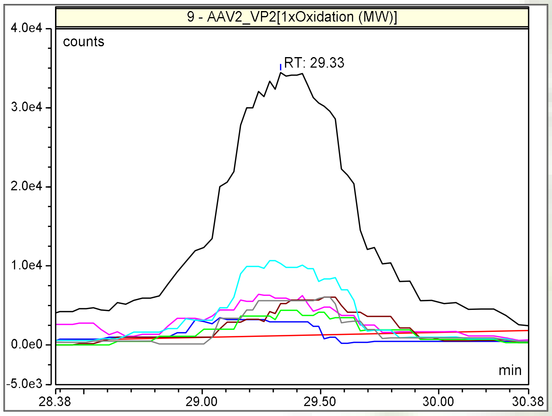

© 2009-2020 Thermo Fisher Scientific Inc. All rights reserved.
Chromeleon 7.3.0.60919

Chromatogram and SST Results

Injection Details

Injection Name:	**20220120_AAV2_HEK_VP Sep_25C_64.5-58.5B_45K R**	*Run Time (min):*	**45.00**
Vial Number:	**R:A1**	*Injection Volume:*	**8.56**
Injection Type:	**Unknown**		
Calibration Level:			
Instrument Method:	**AAV Cap_Prot_Sep_BEH Glycoprotein Amide HILIC_1x150mm_FLR + MS_45K Res_CT25C_**		
Processing Method:	**AAV2 MS Quantitative In-depth Characterization**	*Dilution Factor:*	**1.0000**
Injection Date/Time:	**21/Jan/22 07:32**	*Sample Weight:*	**1.0000**

Chromatogram

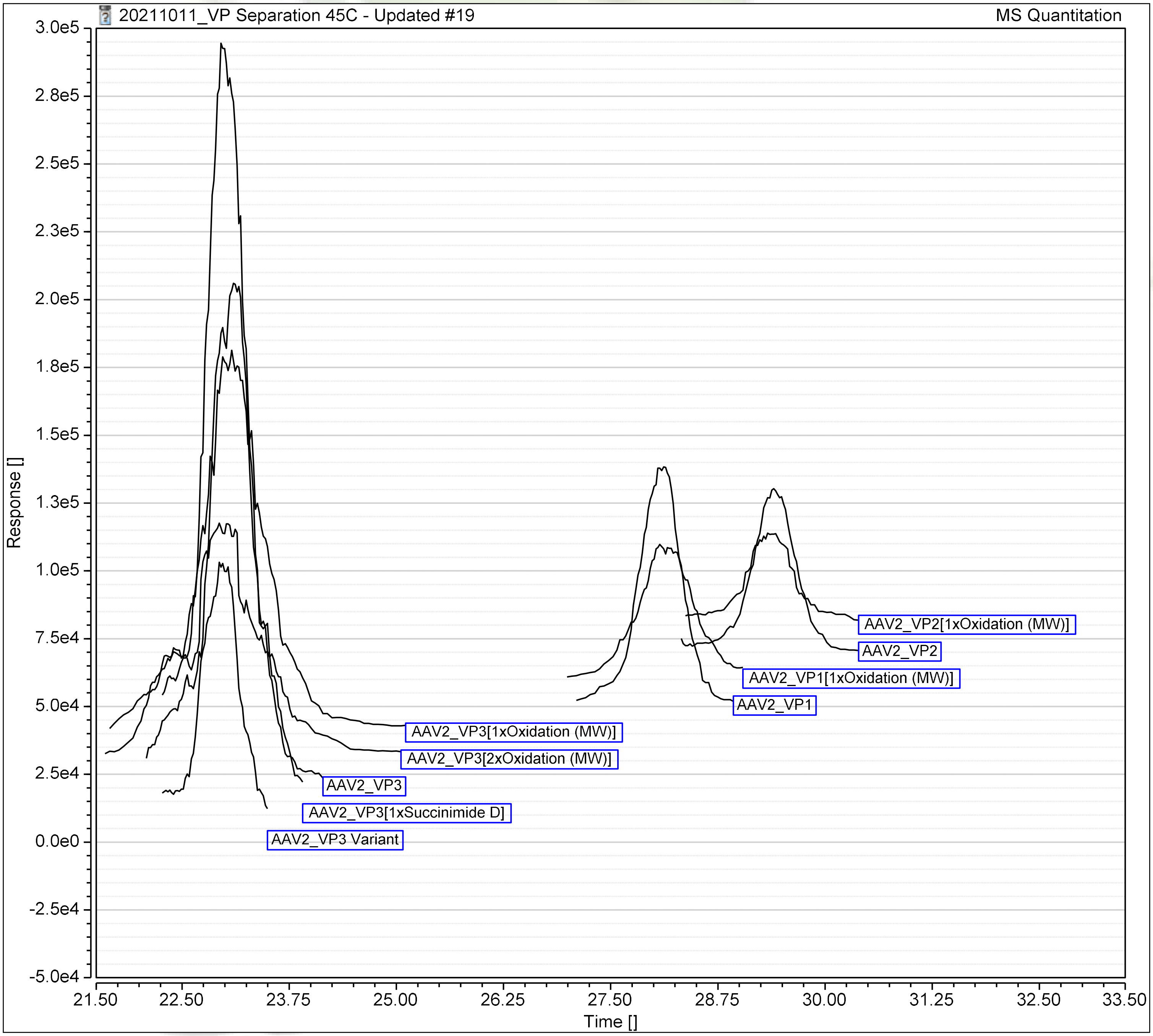

© 2009-2020 Thermo Fisher Scientific Inc. All rights reserved.
Chromeleon 7.3.0.60919

SST Results

No.	Name	Inj.Condition	Peak	Test Result	Injection
**Number of executed test cases:**		**n.a.**	**Total Result:**	**Passed**	

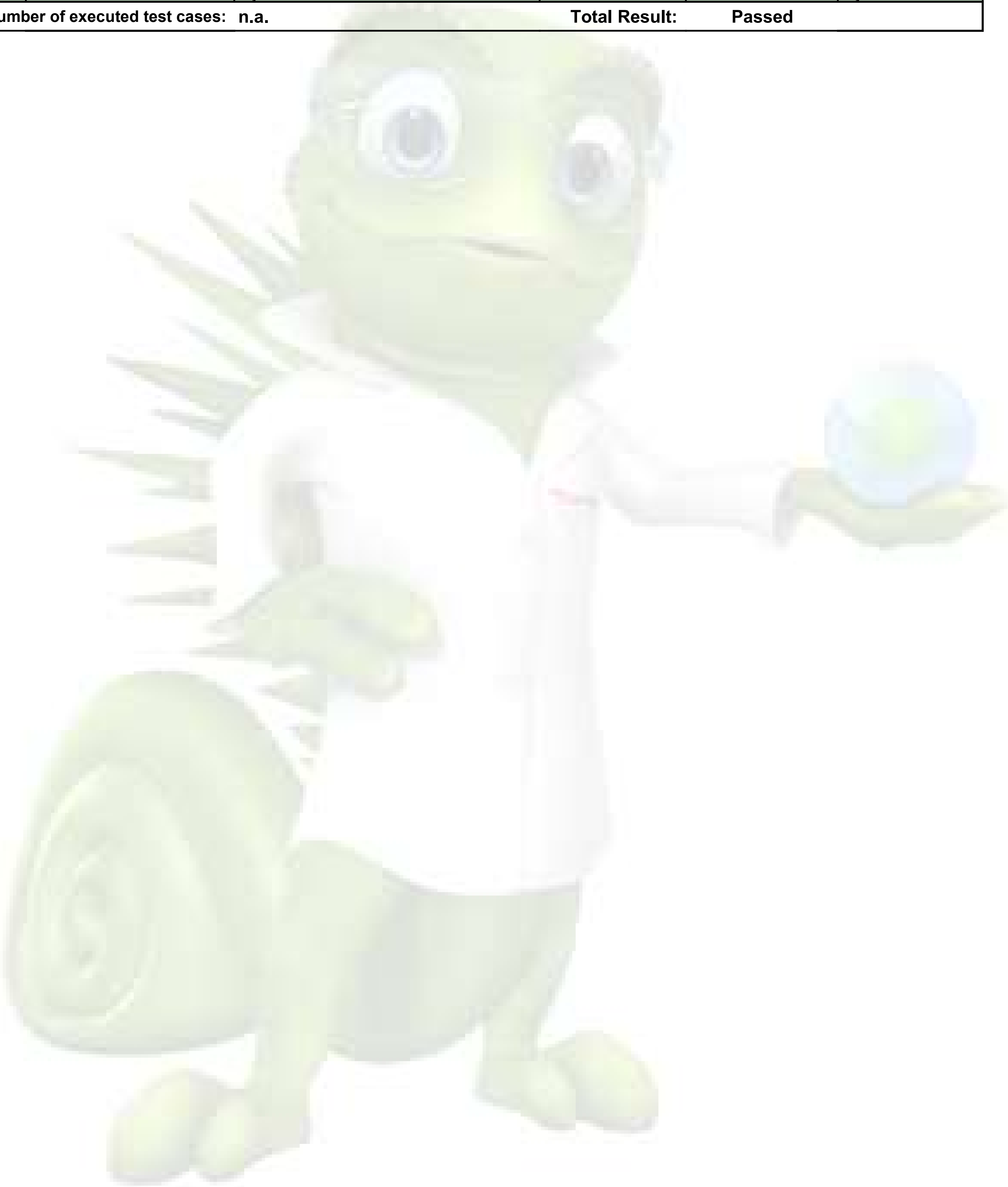

© 2009-2020 Thermo Fisher Scientific Inc. All rights reserved.

Protein Deconvolution Summary

Injection Details

Injection Name:	**20220120_AAV2_HEK_VP Sep_25C_64.5-58.5B_45K Res_1ug_2**	*Injection Volume:*	**8.56**
Vial Number:	**R:A1**	*Instrument Method:*	**AAV Cap_Prot_Sep_BEH Glycoprotein Amide H**
Injection Type:	**Unknown**	*Processing Method:*	**AAV2 MS Quantitative In-depth Characterizatior**
Run Time (min):	**44.99**	*Injection Date/Time:*	**21/Jan/22 08:18**

Chromatogram Component 1

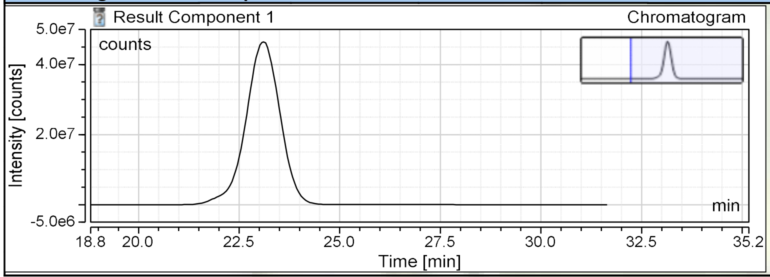

Source Spectrum Component 1

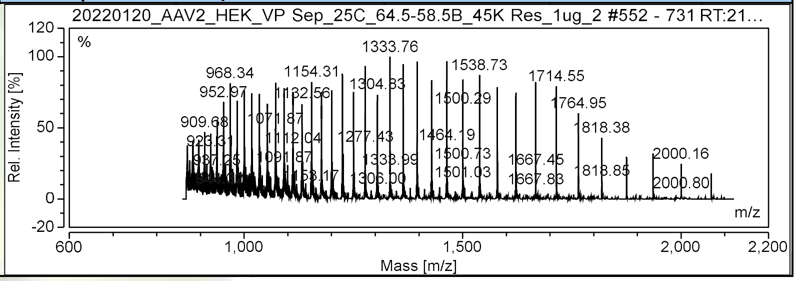

Chromatogram Component 2

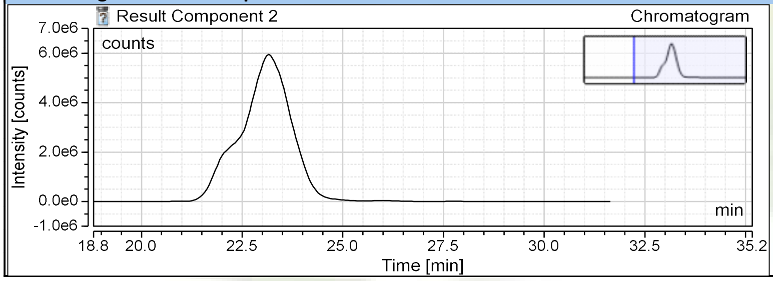

Source Spectrum Component 2

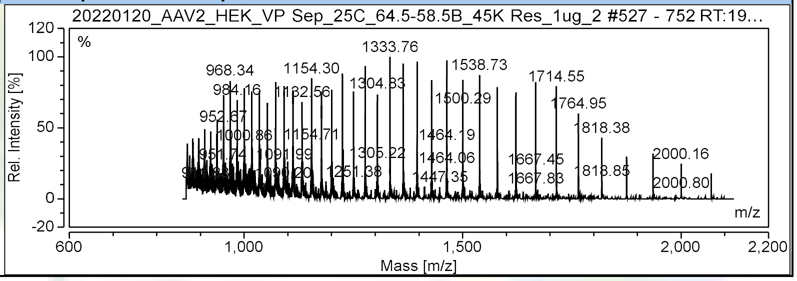

Chromatogram Component 3

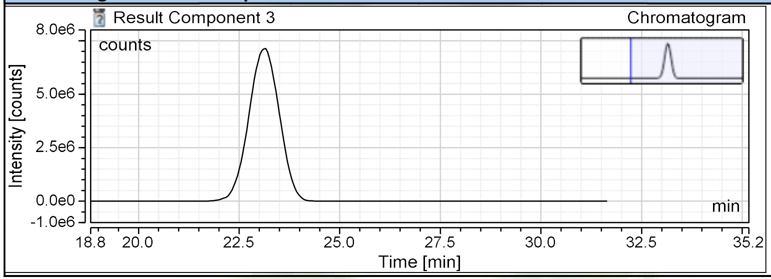

Source Spectrum Component 3

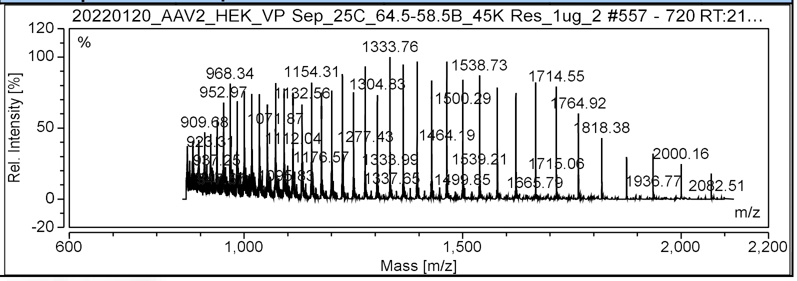

Chromatogram Component 4

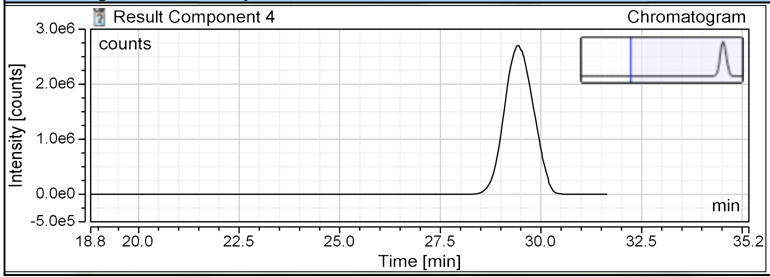

Source Spectrum Component 4

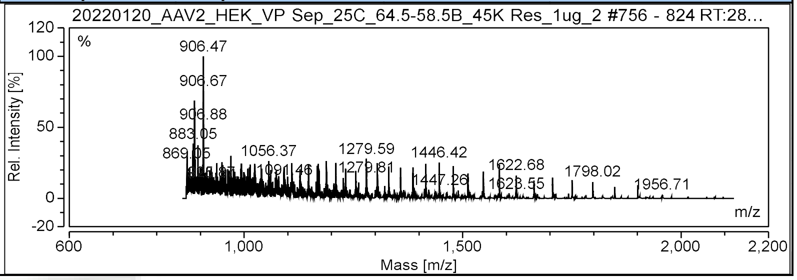

Chromatogram Component 5

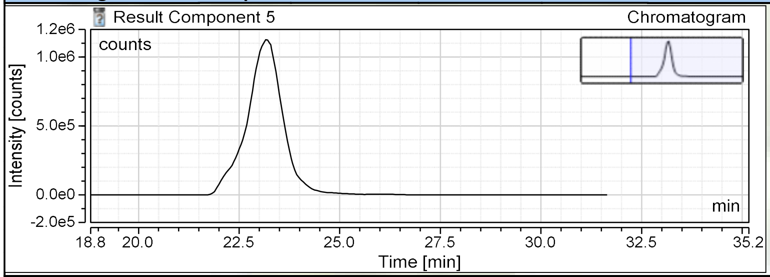

Source Spectrum Component 5

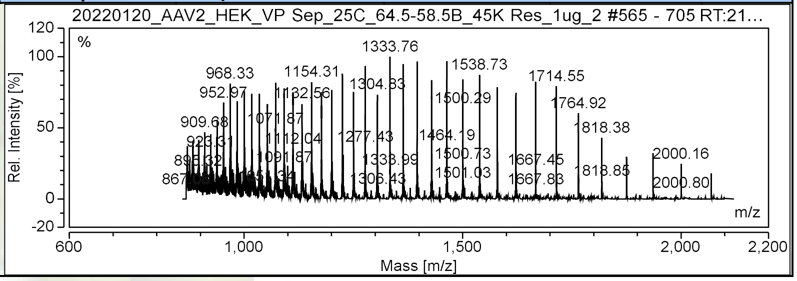

© 2009-2020 Thermo Fisher Scientific Inc. All rights reserved.
Chromeleon 7.3.0.60919

Chromatogram Component 7

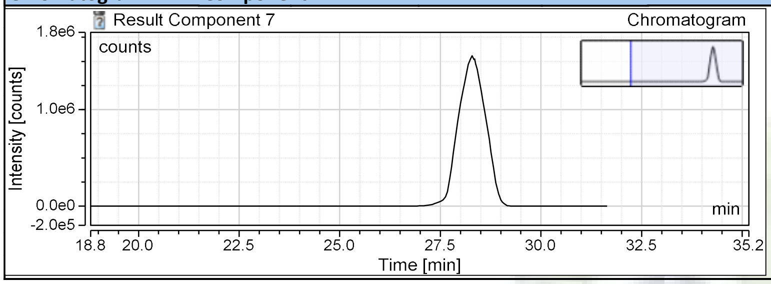

Source Spectrum Component 7

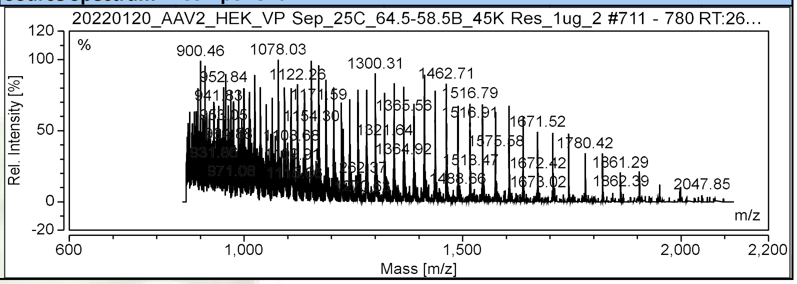

Chromatogram Component 9

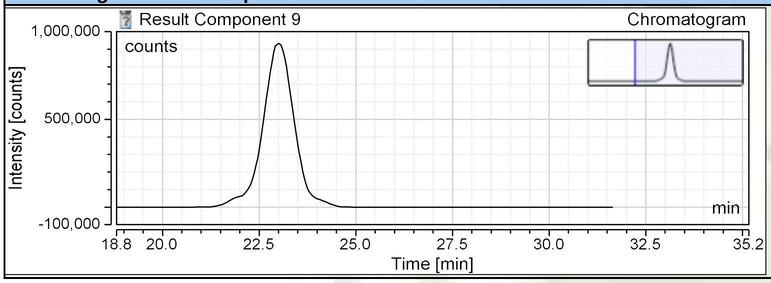

Source Spectrum Component 9

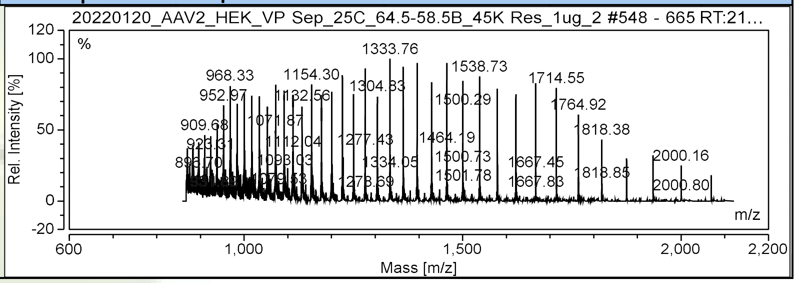

Chromatogram Component 12

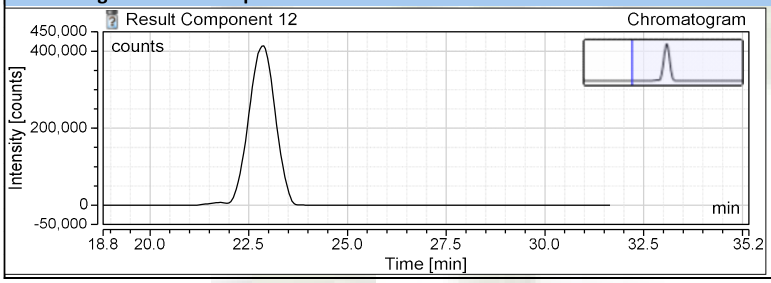

Source Spectrum Component 12

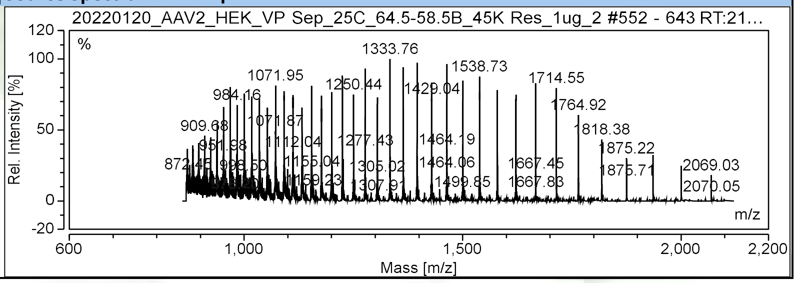

Chromatogram Component 20

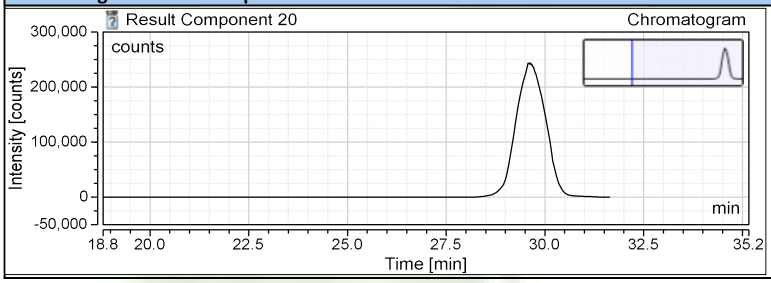

Source Spectrum Component 20

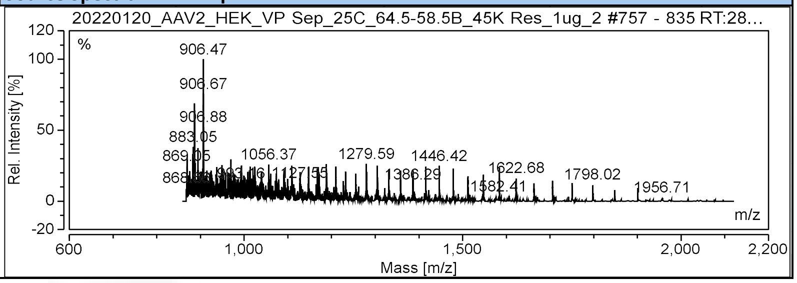

Deconvoluted Spectrum

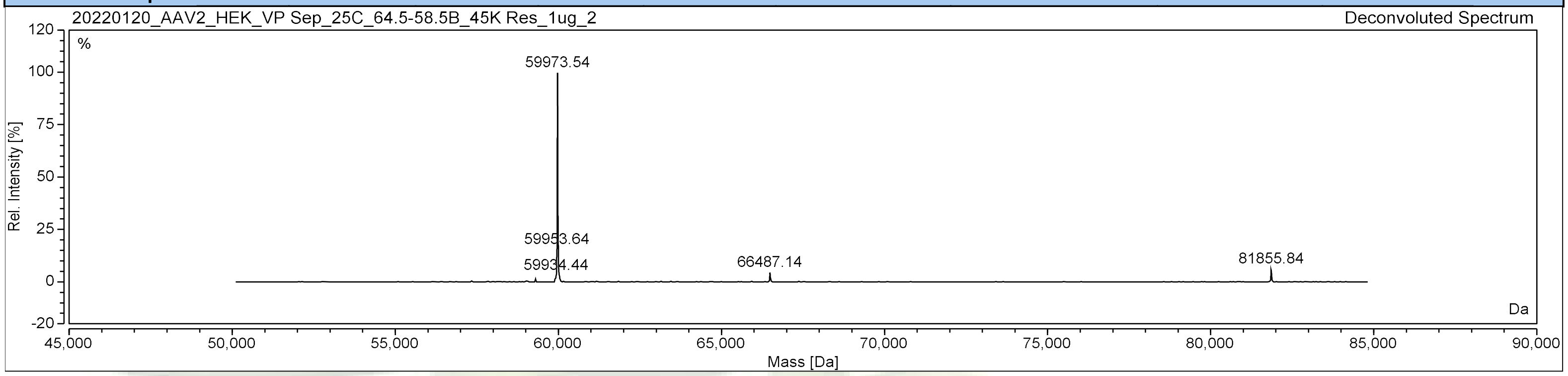

Deconvolution Results

Result Component	RT min	Mass Da	Std. Dev. Da	Std. Dev. ppm	Delta Mass Da	Intensity counts	Fractional Abundance counts	Rel. Abundance %	Number of Charge States	Score
Result Component 1	23.08	59973.436	0.84	13.99	0.000	1959279259	46.2	100.00	41	242.04
Result Component 2	23.11	59991.819	1.81	30.24	18.383	430877006	10.2	21.99	41	161.22
Result Component 3	23.08	59953.840	1.53	25.59	-19.596	302960887	7.1	15.46	34	154.28
Result Component 4	28.00	81855.271	1.70	20.79	21881.835	149335479	3.5	7.62	55	180.65
Result Component 5	29.37	66487.295	1.24	18.66	6513.860	115071618	2.7	5.87	42	195.77
Result Component 6	23.08	60018.234	1.74	28.98	44.799	61338748	1.4	3.13	25	68.82
Result Component 7	23.76	60006.121	1.86	30.94	32.686	59932437	1.4	3.06	39	79.35
Result Component 8	29.37	66504.638	1.95	29.34	6531.202	43476745	1.0	2.22	41	102.72
Result Component 9	27.98	81872.829	2.11	25.73	21899.393	38271649	0.9	1.95	44	90.47

Result Component 11	22.91	59299.871	1.48	25.01	-673.565	28850826	0.7	1.47	30	69.26
Result Component 12	22.40	59954.253	1.72	28.73	-19.183	27440115	0.6	1.40	33	139.46
Result Component 19	27.67	81874.453	2.73	33.39	21901.017	10900834	0.3	0.56	50	109.08
Result Component 77	24.00	60020.804	1.54	25.71	47.368	3356483	0.1	0.17	33	78.05
Result Component 152	29.15	66467.903	1.85	27.80	6494.467	1942465	0.0	0.10	19	65.67

© 2009-2020 Thermo Fisher Scientific Inc. All rights reserved.
Chromeleon 7.3.0.60919

Component Summary

Injection Details

Injection Name:	**20220120_AAV2_HEK_VP Sep_25C_64.5-5**	*Injection Volume:*	**8.56**
Vial Number:	**R:A1**	*Instrument Method:*	**AAV Cap_Prot_Sep_BEH Glycoprotein**
Injection Type:	**Unknown**	*Processing Method:*	**AAV2 MS Quantitative In-depth Charact**
Run Time (min):	**44.99**	*Injection Date/Time:*	**21/Jan/22 08:18**

Peak Name	Retention Time min	Area counts*min	Relative Area %	Quantitation Ions m/z	Mol.Mass	Mass Confirmation Pass/Fail
AAV2_VP3[1xOxidation (MW)]	22.988	172743.238	23.92	1277.428,1396.179,1464.229,1250.823,1305.183,1225.324	59989.780	PASS
AAV2_VP3 Variant	23.050	55540.588	7.69	1447.374,957.488,1380.090,1163.736,1099.120,973.145	59301.030	PASS
AAV2_VP3[1xSuccinimide D]	23.071	196617.847	27.23	952.674,923.374,968.020,937.789,1017.182,1000.250	59955.770	PASS
AAV2_VP3	23.071	92029.695	12.74	1364.311,1500.570,1333.966,1395.925,1463.892,1225.374	59973.780	PASS
AAV2_VP3[2xOxidation (MW)]	23.092	75537.982	10.46	1133.193,1092.027,1112.217,1072.520,1539.665,1464.606	60005.780	PASS
AAV2_VP1[1xOxidation (MW)]	28.146	38902.016	5.39	1343.191,1154.150,1388.700,1187.572,1412.610,1170.616	81870.890	PASS
AAV2_VP1	28.168	40665.176	5.63	1342.896,1388.388,1365.271,1300.301,1187.321,1279.998	81854.900	PASS
AAV2_VP2	29.391	30432.656	4.21	1446.392,1415.628,1304.683,1478.503,1209.871,1386.167	66487.900	PASS
AAV2_VP2[1xOxidation (MW)]	29.437	19629.625	2.72	1446.760,1305.022,1167.738,1358.263,1147.628,1331.107	66503.900	PASS
			**100.00**			

Component Name: AAV2_VP3 Variant

Quantitation ions: 1447.374,957.488,1380.090,1163.736,1099.120,973.145

Retention time (min):	22.89
Expected Mass:	59301.030
Area (count*min):	55541
Intensity (counts):	109089
Amount:	-

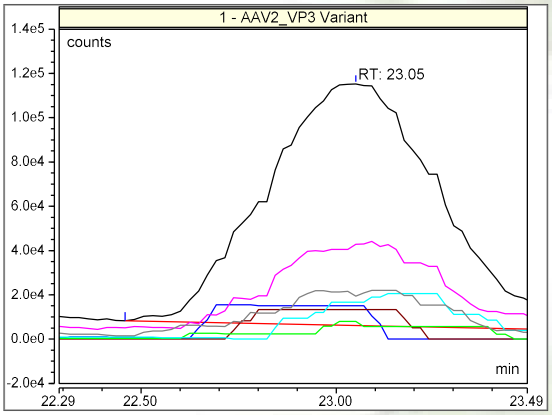

© 2009-2020 Thermo Fisher Scientific Inc. All rights reserved.
Chromeleon 7.3.0.60919

Component Name:	AAV2_VP3[1xSuccinimide D]
Quantitation ions:	952.674,923.374,968.020,937.789,1017.182,1000.250
Retention time (min):	23.09
Expected Mass:	59955.770
Area (count*min):	196618
Intensity (counts):	370451
Amount:	-

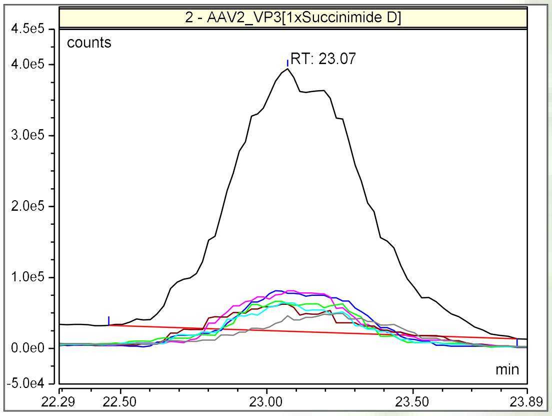

© 2009-2020 Thermo Fisher Scientific Inc. All rights reserved.
Chromeleon 7.3.0.60919

Component Name:	AAV2_VP3
Quantitation ions:	1364.311,1500.570,1333.966,1395.925,1463.892,1225.374
Retention time (min):	23.10
Expected Mass:	59973.780
Area (count*min):	92030
Intensity (counts):	160211
Amount:	-

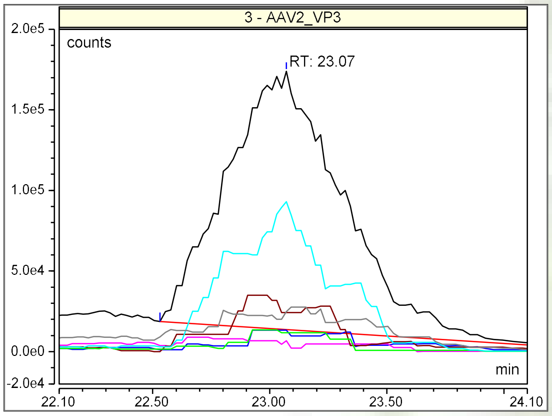

© 2009-2020 Thermo Fisher Scientific Inc. All rights reserved.
Chromeleon 7.3.0.60919

Component Name:	AAV2_VP3[2xOxidation (MW)]
Quantitation ions:	1133.193,1092.027,1112.217,1072.520,1539.665,1464.606
Retention time (min):	23.33
Expected Mass:	60005.780
Area (count*min):	75538
Intensity (counts):	71338
Amount:	-

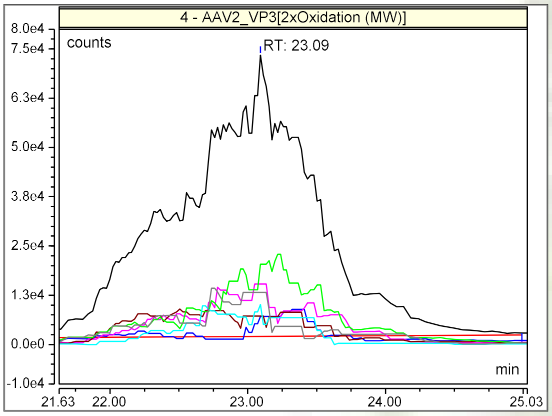

© 2009-2020 Thermo Fisher Scientific Inc. All rights reserved.
Chromeleon 7.3.0.60919

Component Name:	AAV2_VP3[1xOxidation (MW)]
Quantitation ions:	1277.428,1396.179,1464.229,1250.823,1305.183,1225.324
Retention time (min):	23.37
Expected Mass:	59989.780
Area (count*min):	172743
Intensity (counts):	256693
Amount:	-

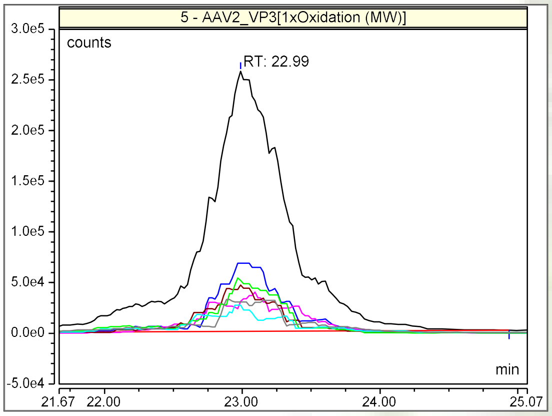

© 2009-2020 Thermo Fisher Scientific Inc. All rights reserved.
Chromeleon 7.3.0.60919

Component Name:	AAV2_VP1
Quantitation ions:	1342.896,1388.388,1365.271,1300.301,1187.321,1279.998
Retention time (min):	28.03
Expected Mass:	81854.900
Area (count*min):	40665
Intensity (counts):	68019
Amount:	-

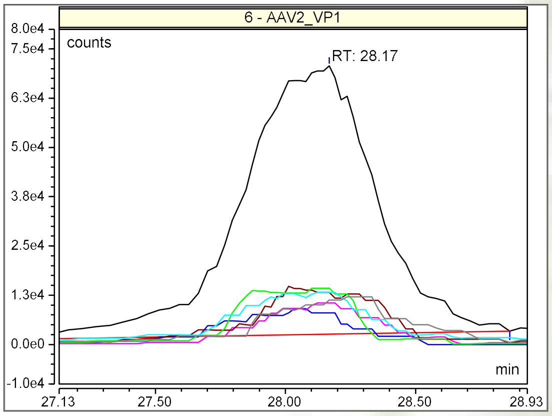

© 2009-2020 Thermo Fisher Scientific Inc. All rights reserved.
Chromeleon 7.3.0.60919

Component Name:	AAV2_VP1[1xOxidation (MW)]
Quantitation ions:	1343.191,1154.150,1388.700,1187.572,1412.610,1170.616
Retention time (min):	28.03
Expected Mass:	81870.890
Area (count*min):	38902
Intensity (counts):	62034
Amount:	-

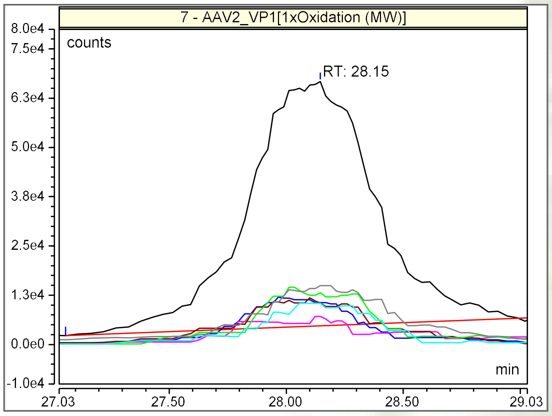

© 2009-2020 Thermo Fisher Scientific Inc. All rights reserved.
Chromeleon 7.3.0.60919

Component Name:	AAV2_VP2
Quantitation ions:	1446.392,1415.628,1304.683,1478.503,1209.871,1386.167
Retention time (min):	29.34
Expected Mass:	66487.900
Area (count*min):	30433
Intensity (counts):	47609
Amount:	-

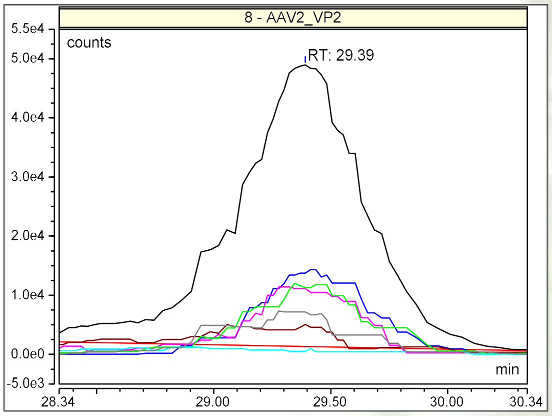

© 2009-2020 Thermo Fisher Scientific Inc. All rights reserved.
Chromeleon 7.3.0.60919

Component Name: AAV2_VP2[1xOxidation (MW)]

Quantitation ions:	1446.760,1305.022,1167.738,1358.263,1147.628,1331.107
Retention time (min):	29.38
Expected Mass:	66503.900
Area (count*min):	19630
Intensity (counts):	30364
Amount:	-

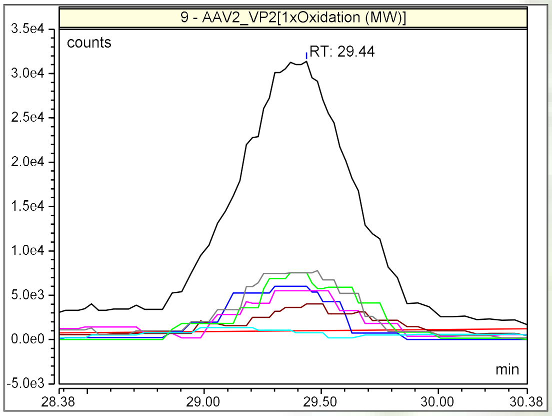

© 2009-2020 Thermo Fisher Scientific Inc. All rights reserved.
Chromeleon 7.3.0.60919

Chromatogram and SST Results

Injection Details

Injection Name:	**20220120_AAV2_HEK_VP Sep_25C_64.5-58.5B_45K F**	*Run Time (min):*	**44.99**
Vial Number:	**R:A1**	*Injection Volume:*	**8.56**
Injection Type:	**Unknown**		
Calibration Level:			
Instrument Method:	**AAV Cap_Prot_Sep_BEH Glycoprotein Amide HILIC_1x150mm_FLR + MS_45K Res_CT25C_**		
Processing Method:	**AAV2 MS Quantitative In-depth Characterization**	*Dilution Factor:*	**1.0000**
Injection Date/Time:	**21/Jan/22 08:18**	*Sample Weight:*	**1.0000**

Chromatogram

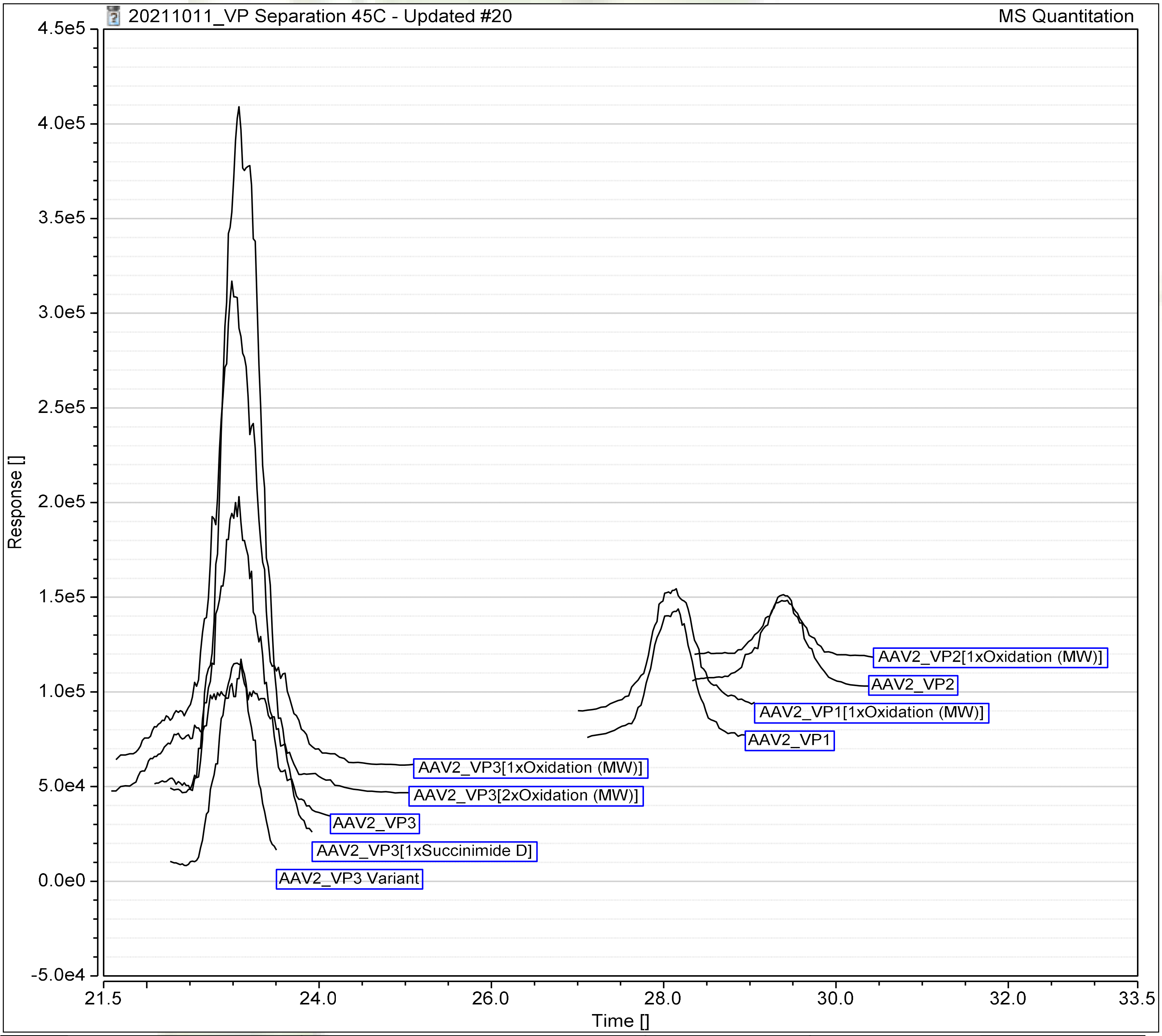

SST Results

© 2009-2020 Thermo Fisher Scientific Inc. All rights reserved.
Chromeleon 7.3.0.60919

No.	Name	Inj.Condition	Peak	Test Result	Injection
**Number of executed test cases:**		**n.a.**	**Total Result:**	**Passed**	

© 2009-2020 Thermo Fisher Scientific Inc. All rights reserved.

Protein Deconvolution Summary

Injection Details

Injection Name:	**20220120_AAV2_HEK_VP Sep_25C_64.5-58.5B_45K Res_1ug_3**	*Injection Volume:*	**8.56**
Vial Number:	**R:A1**	*Instrument Method:*	**AAV Cap_Prot_Sep_BEH Glycoprotein Amide H**
Injection Type:	**Unknown**	*Processing Method:*	**AAV2 MS Quantitative In-depth Characterizatior**
Run Time (min):	**45.00**	*Injection Date/Time:*	**21/Jan/22 09:04**

Chromatogram **Component 1**

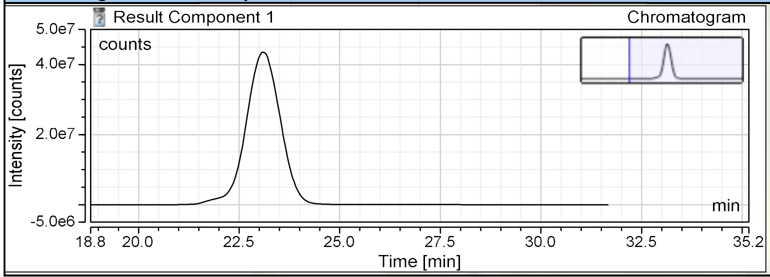

Source Spectrum **Component 1**

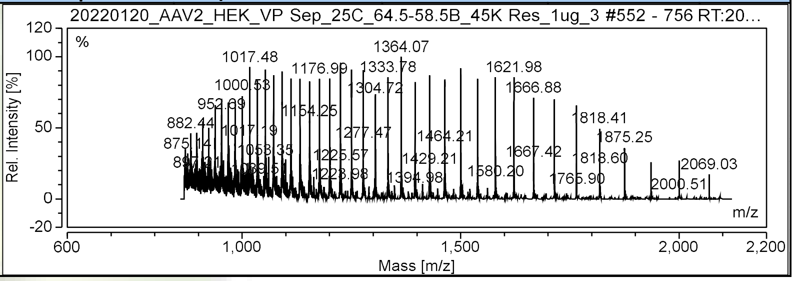

Chromatogram **Component 2**

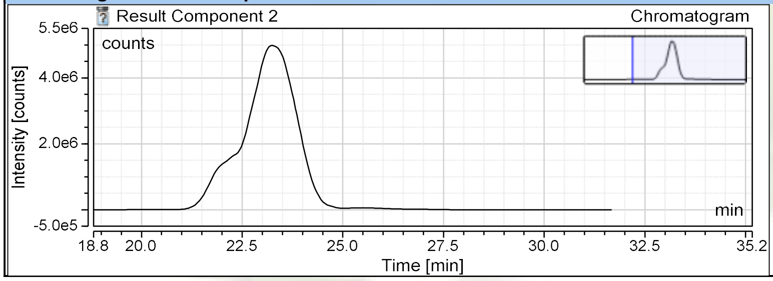

Source Spectrum **Component 2**

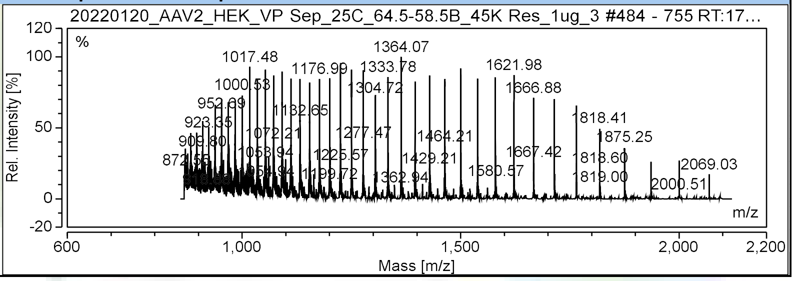

Chromatogram **Component 3**

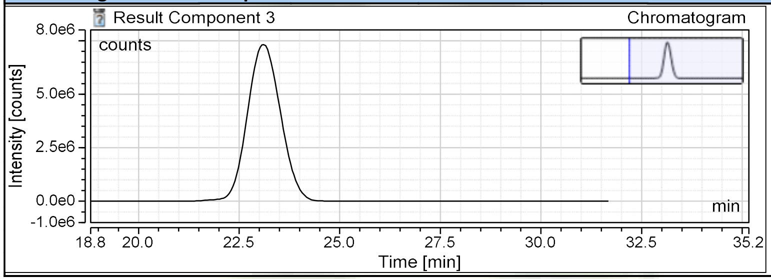

Source Spectrum **Component 3**

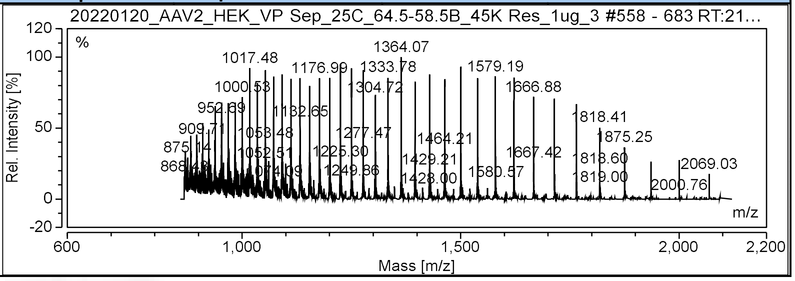

Chromatogram **Component 4**

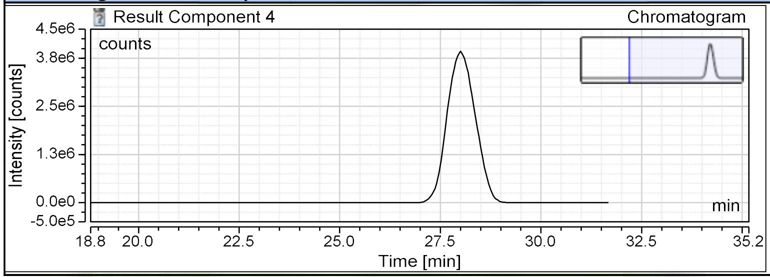

Source Spectrum **Component 4**

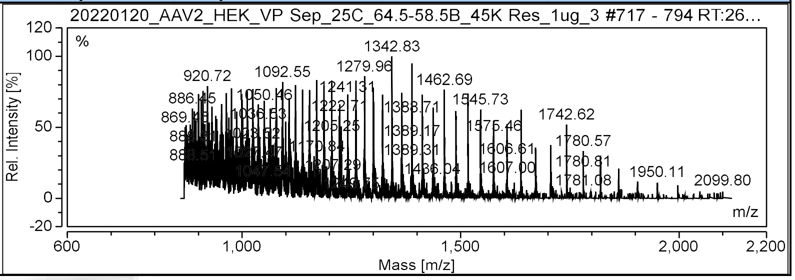

Chromatogram **Component 5**

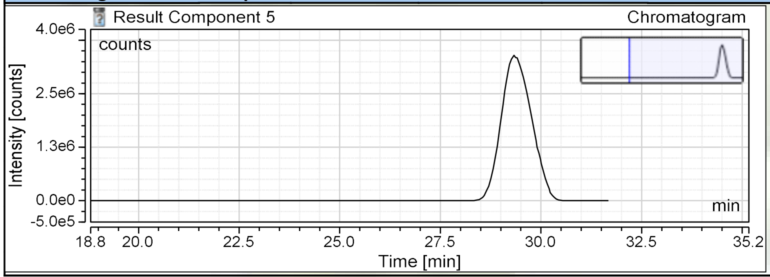

Source Spectrum **Component 5**

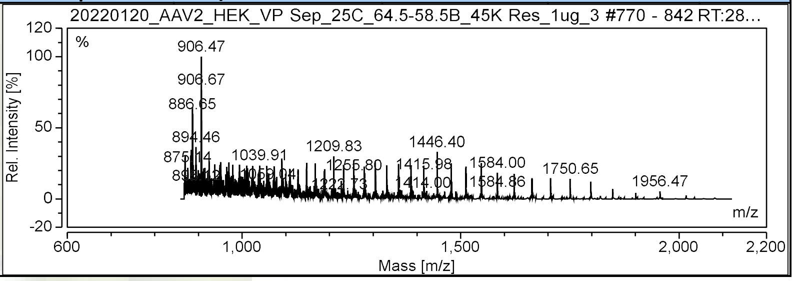

© 2009-2020 Thermo Fisher Scientific Inc. All rights reserved.
Chromeleon 7.3.0.60919

Chromatogram Component 7

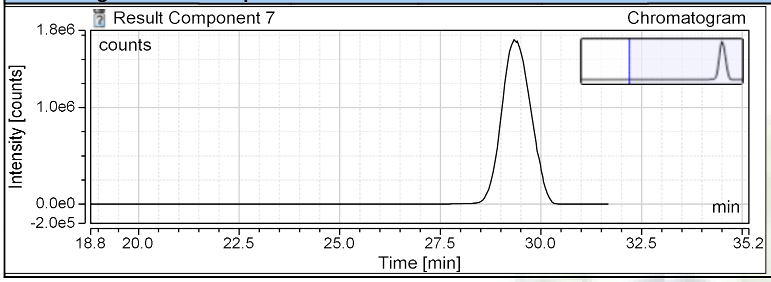

Source Spectrum Component 7

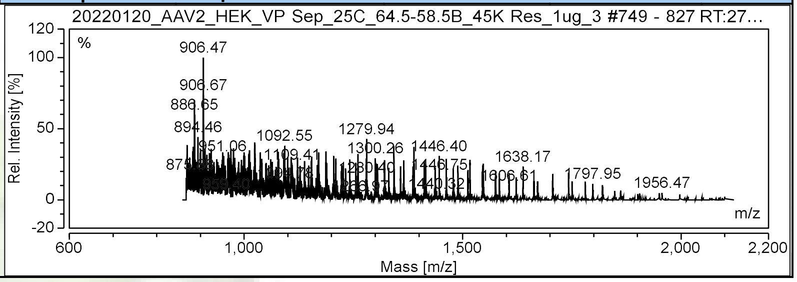

Chromatogram Component 9

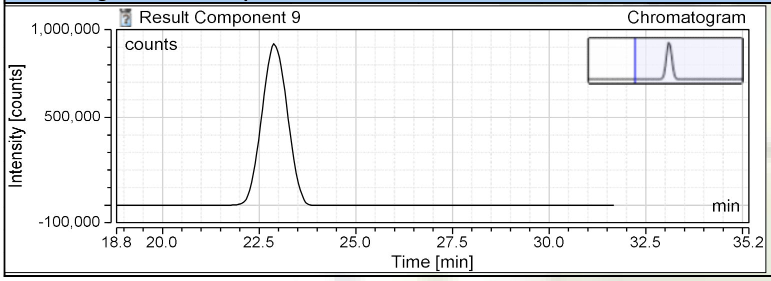

Source Spectrum Component 9

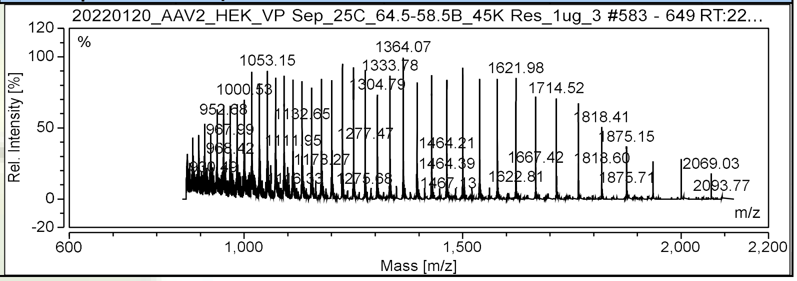

Chromatogram Component 12

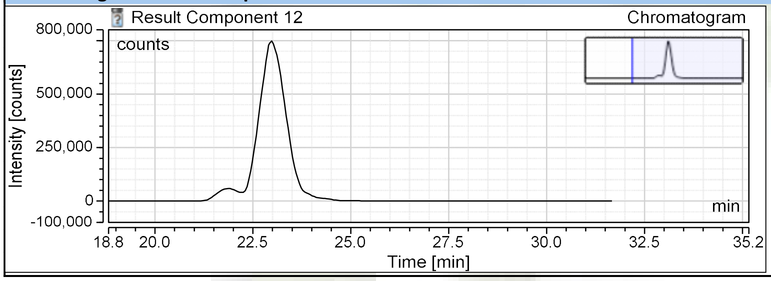

Source Spectrum Component 12

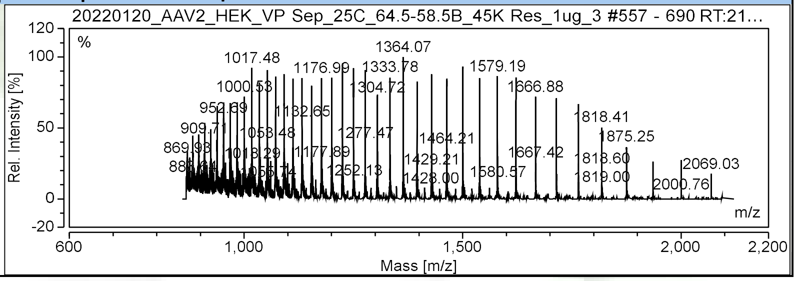

Chromatogram Component 20

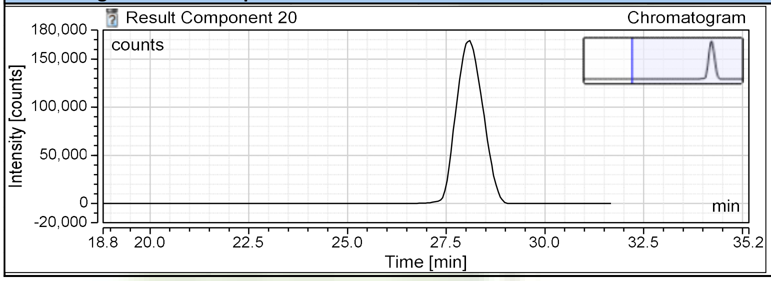

Source Spectrum Component 20

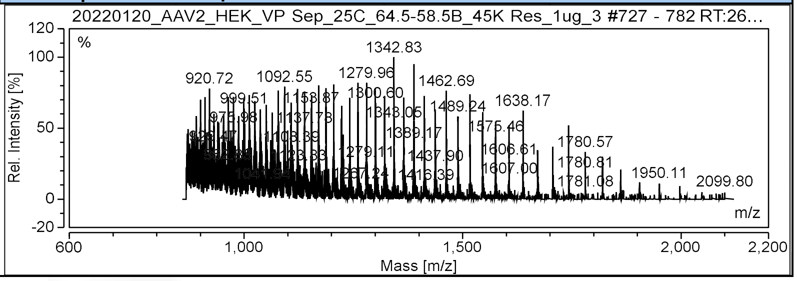

Deconvoluted Spectrum

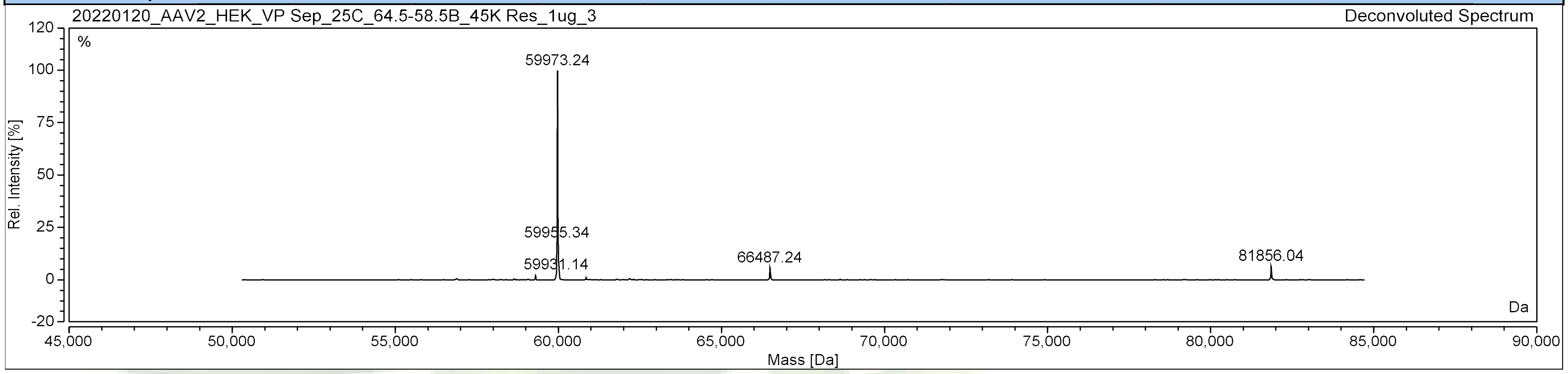

Deconvolution Results

Result Component	RT min	Mass Da	Std. Dev. Da	Std. Dev. ppm	Delta Mass Da	Intensity counts	Fractional Abundance counts	Rel. Abundance %	Number of Charge States	Score
Result Component 1	23.08	59973.436	0.84	13.99	0.000	1959279259	46.2	100.00	41	242.04
Result Component 2	23.11	59991.819	1.81	30.24	18.383	430877006	10.2	21.99	41	161.22
Result Component 3	23.08	59953.840	1.53	25.59	-19.596	302960887	7.1	15.46	34	154.28
Result Component 4	28.00	81855.271	1.70	20.79	21881.835	149335479	3.5	7.62	55	180.65
Result Component 5	29.37	66487.295	1.24	18.66	6513.860	115071618	2.7	5.87	42	195.77
Result Component 6	23.08	60018.234	1.74	28.98	44.799	61338748	1.4	3.13	25	68.82
Result Component 7	23.76	60006.121	1.86	30.94	32.686	59932437	1.4	3.06	39	79.35
Result Component 8	29.37	66504.638	1.95	29.34	6531.202	43476745	1.0	2.22	41	102.72
Result Component 9	27.98	81872.829	2.11	25.73	21899.393	38271649	0.9	1.95	44	90.47

Result Component 11	22.91	59299.871	1.48	25.01	-673.565	28850826	0.7	1.47	30	69.26
Result Component 12	22.40	59954.253	1.72	28.73	-19.183	27440115	0.6	1.40	33	139.46
Result Component 19	27.67	81874.453	2.73	33.39	21901.017	10900834	0.3	0.56	50	109.08
Result Component 77	24.00	60020.804	1.54	25.71	47.368	3356483	0.1	0.17	33	78.05
Result Component 152	29.15	66467.903	1.85	27.80	6494.467	1942465	0.0	0.10	19	65.67

© 2009-2020 Thermo Fisher Scientific Inc. All rights reserved.
Chromeleon 7.3.0.60919

Component Summary

Injection Details

Injection Name:	**20220120_AAV2_HEK_VP Sep_25C_64.5-5**	*Injection Volume:*	**8.56**
Vial Number:	**R:A1**	*Instrument Method:*	**AAV Cap_Prot_Sep_BEH Glycoprotein**
Injection Type:	**Unknown**	*Processing Method:*	**AAV2 MS Quantitative In-depth Charact**
Run Time (min):	**45.00**	*Injection Date/Time:*	**21/Jan/22 09:04**

Peak Name	Retention Time min	Area counts*min	Relative Area %	Quantitation Ions m/z	Mol.Mass	Mass Confirmation Pass/Fail
AAV2_VP3 Variant	22.969	67967.176	8.71	1447.374,957.488,1380.090,1163.736,1099.120,973.145	59301.030	PASS
AAV2_VP3[1xOxidation (MW)]	23.010	139936.624	17.94	1277.428,1396.179,1464.229,1250.823,1305.183,1225.324	59989.780	PASS
AAV2_VP3[2xOxidation (MW)]	23.010	129323.456	16.58	1133.193,1092.027,1112.217,1072.520,1539.665,1464.606	60005.780	PASS
AAV2_VP3	23.052	130658.262	16.75	1364.311,1500.570,1333.966,1395.925,1463.892,1225.374	59973.780	PASS
AAV2_VP3[1xSuccinimide D]	23.135	171338.149	21.97	952.674,923.374,968.020,937.789,1017.182,1000.250	59955.770	PASS
AAV2_VP1[1xOxidation (MW)]	28.028	38381.637	4.92	1343.191,1154.150,1388.700,1187.572,1412.610,1170.616	81870.890	PASS
AAV2_VP1	28.050	39880.541	5.11	1342.896,1388.388,1365.271,1300.301,1187.321,1279.998	81854.900	PASS
AAV2_VP2[1xOxidation (MW)]	29.333	30324.198	3.89	1446.760,1305.022,1167.738,1358.263,1147.628,1331.107	66503.900	PASS
AAV2_VP2	29.355	32159.860	4.12	1446.392,1415.628,1304.683,1478.503,1209.871,1386.167	66487.900	PASS
			**100.00**			

Component Name: AAV2_VP3 Variant

Quantitation ions:	1447.374,957.488,1380.090,1163.736,1099.120,973.145
Retention time (min):	22.89
Expected Mass:	59301.030
Area (count*min):	67967
Intensity (counts):	133312
Amount:	-

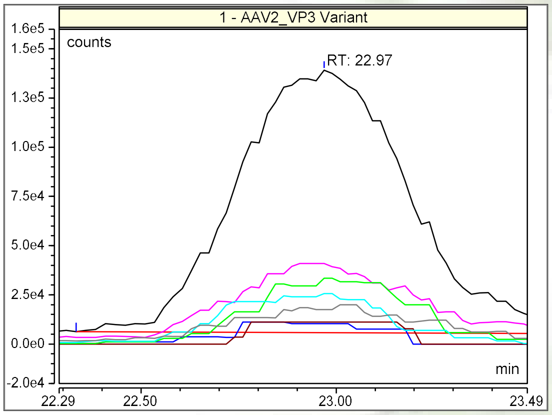

© 2009-2020 Thermo Fisher Scientific Inc. All rights reserved.
Chromeleon 7.3.0.60919

Component Name:	AAV2_VP3[1xSuccinimide D]
Quantitation ions:	952.674,923.374,968.020,937.789,1017.182,1000.250
Retention time (min):	23.09
Expected Mass:	59955.770
Area (count*min):	171338
Intensity (counts):	292135
Amount:	-

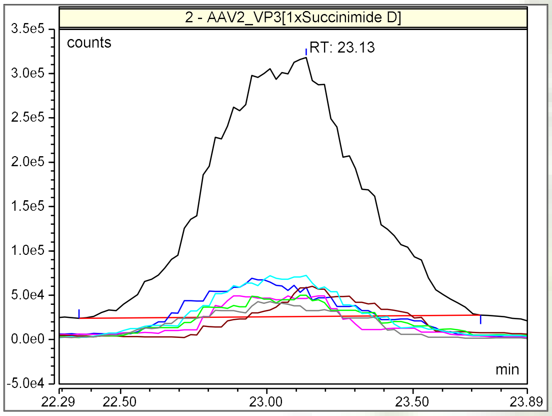

© 2009-2020 Thermo Fisher Scientific Inc. All rights reserved.
Chromeleon 7.3.0.60919

Component Name:	AAV2_VP3
Quantitation ions:	1364.311,1500.570,1333.966,1395.925,1463.892,1225.374
Retention time (min):	23.10
Expected Mass:	59973.780
Area (count*min):	130658
Intensity (counts):	212787
Amount:	-

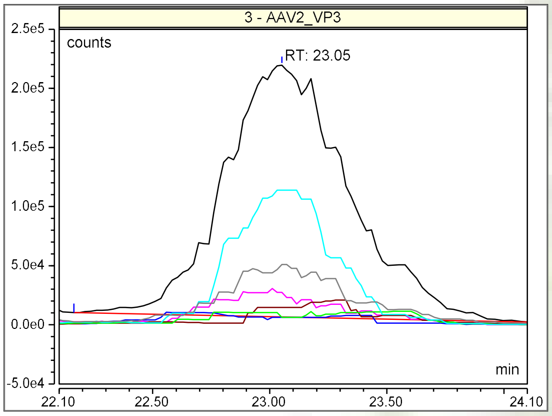

© 2009-2020 Thermo Fisher Scientific Inc. All rights reserved.
Chromeleon 7.3.0.60919

Component Name:	AAV2_VP3[2xOxidation (MW)]
Quantitation ions:	1133.193,1092.027,1112.217,1072.520,1539.665,1464.606
Retention time (min):	23.33
Expected Mass:	60005.780
Area (count*min):	129323
Intensity (counts):	152181
Amount:	-

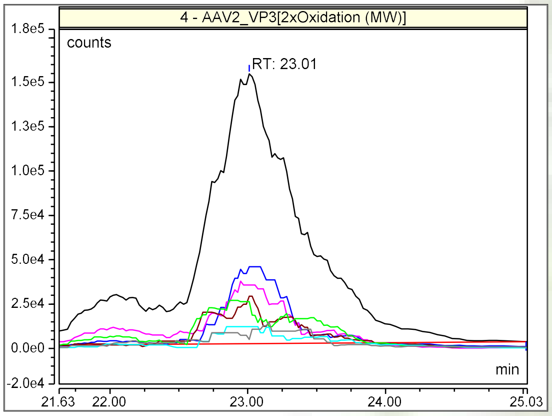

© 2009-2020 Thermo Fisher Scientific Inc. All rights reserved.
Chromeleon 7.3.0.60919

Component Name:	AAV2_VP3[1xOxidation (MW)]
Quantitation ions:	1277.428,1396.179,1464.229,1250.823,1305.183,1225.324
Retention time (min):	23.37
Expected Mass:	59989.780
Area (count*min):	139937
Intensity (counts):	170816
Amount:	-

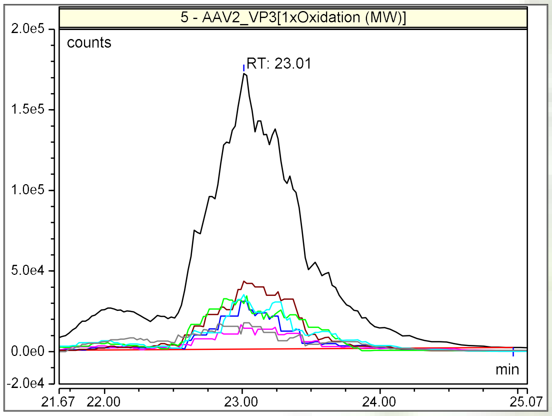

© 2009-2020 Thermo Fisher Scientific Inc. All rights reserved.
Chromeleon 7.3.0.60919

Component Name:	AAV2_VP1
Quantitation ions:	1342.896,1388.388,1365.271,1300.301,1187.321,1279.998
Retention time (min):	28.03
Expected Mass:	81854.900
Area (count*min):	39881
Intensity (counts):	69482
Amount:	-

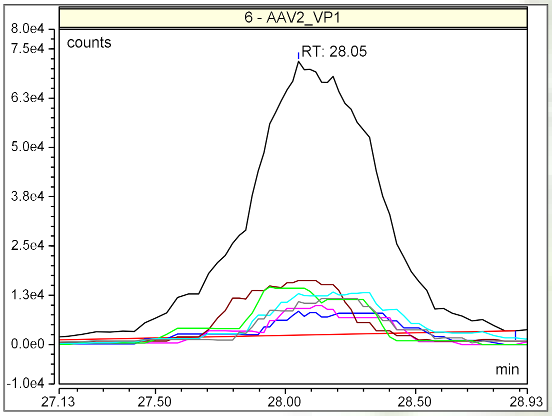

© 2009-2020 Thermo Fisher Scientific Inc. All rights reserved.
Chromeleon 7.3.0.60919

Component Name:	AAV2_VP1[1xOxidation (MW)]
Quantitation ions:	1343.191,1154.150,1388.700,1187.572,1412.610,1170.616
Retention time (min):	28.03
Expected Mass:	81870.890
Area (count*min):	38382
Intensity (counts):	57736
Amount:	-

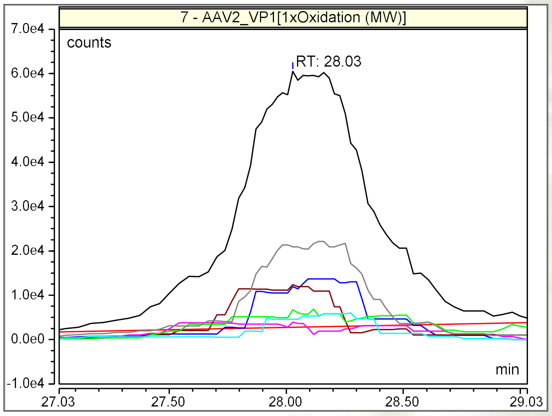

© 2009-2020 Thermo Fisher Scientific Inc. All rights reserved.
Chromeleon 7.3.0.60919

Component Name:	AAV2_VP2
Quantitation ions:	1446.392,1415.628,1304.683,1478.503,1209.871,1386.167
Retention time (min):	29.34
Expected Mass:	66487.900
Area (count*min):	32160
Intensity (counts):	54720
Amount:	-

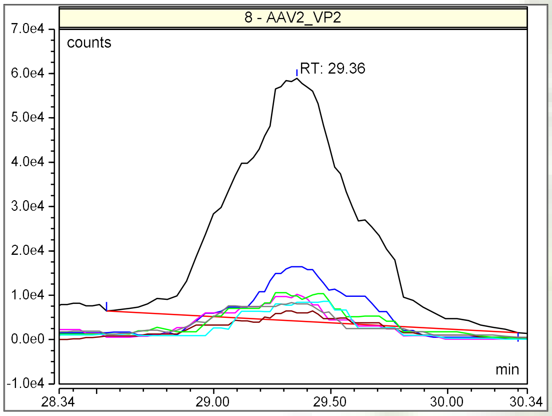

© 2009-2020 Thermo Fisher Scientific Inc. All rights reserved.
Chromeleon 7.3.0.60919

Component Name:	AAV2_VP2[1xOxidation (MW)]
Quantitation ions:	1446.760,1305.022,1167.738,1358.263,1147.628,1331.107
Retention time (min):	29.38
Expected Mass:	66503.900
Area (count*min):	30324
Intensity (counts):	48211
Amount:	-

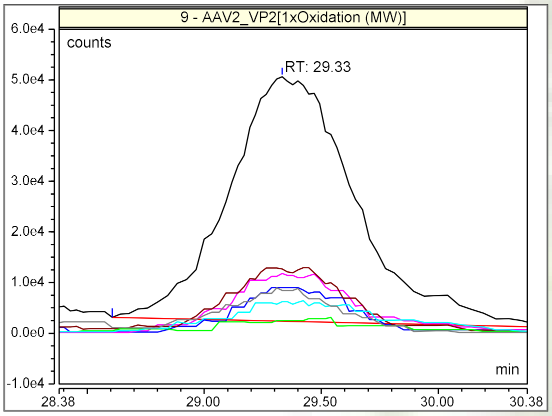

© 2009-2020 Thermo Fisher Scientific Inc. All rights reserved.
Chromeleon 7.3.0.60919

Chromatogram and SST Results

Injection Details

Injection Name:	**20220120_AAV2_HEK_VP Sep_25C_64.5-58.5B_45K R**	*Run Time (min):*	**45.00**
Vial Number:	**R:A1**	*Injection Volume:*	**8.56**
Injection Type:	**Unknown**		
Calibration Level:			
Instrument Method:	**AAV Cap_Prot_Sep_BEH Glycoprotein Amide HILIC_1x150mm_FLR + MS_45K Res_CT25C_**		
Processing Method:	**AAV2 MS Quantitative In-depth Characterization**	*Dilution Factor:*	**1.0000**
Injection Date/Time:	**21/Jan/22 09:04**	*Sample Weight:*	**1.0000**

Chromatogram

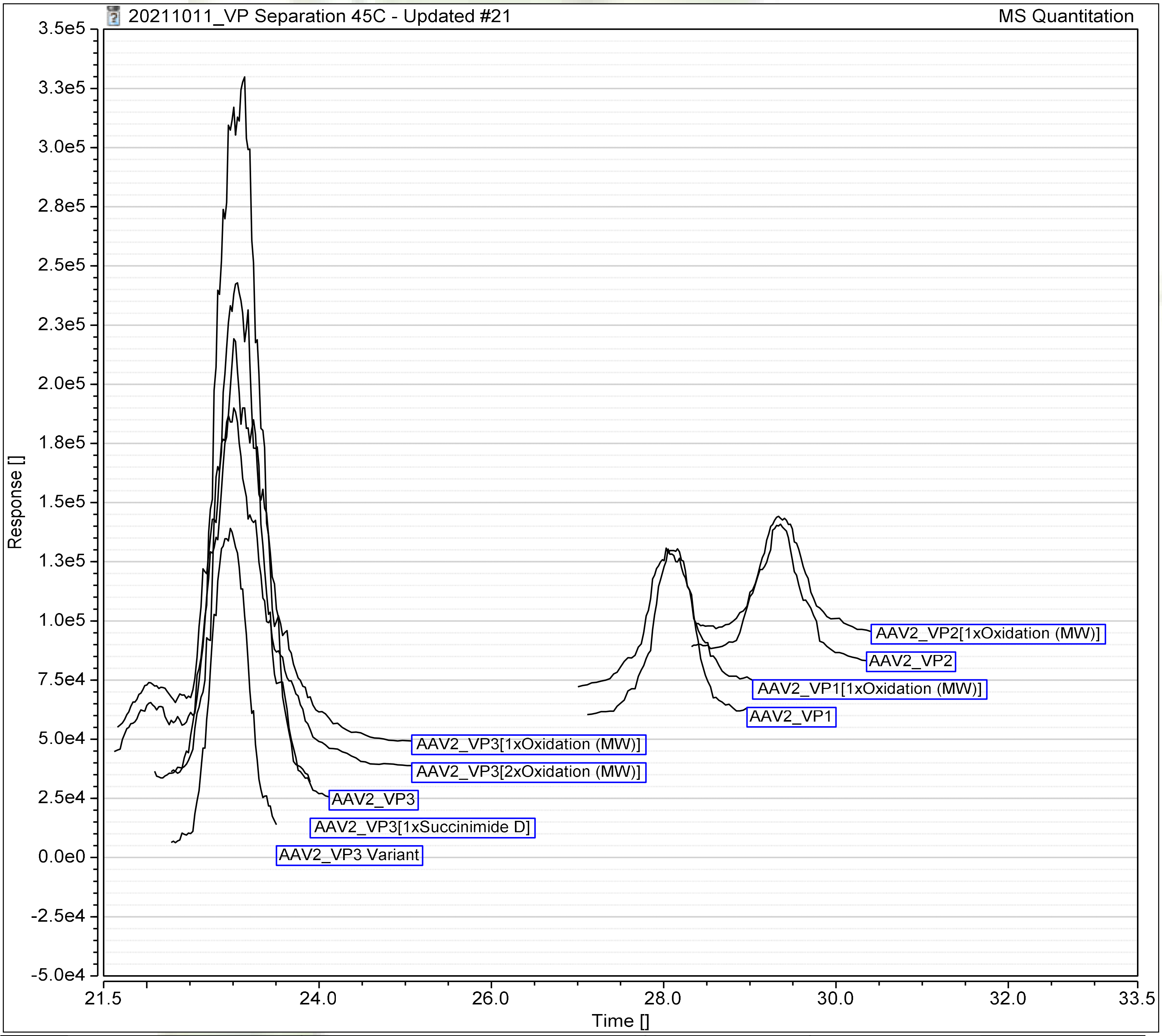

SST Results

© 2009-2020 Thermo Fisher Scientific Inc. All rights reserved.
Chromeleon 7.3.0.60919

No.	Name	Inj.Condition	Peak	Test Result	Injection
**Number of executed test cases:**		**n.a.**	**Total Result:**	**Passed**	

© 2009-2020 Thermo Fisher Scientific Inc. All rights reserved.
